# Supplementary material for: Copper(I)-catalyzed diastereo- and enantio-selective construction of optically pure exocyclic allenes
Source: Nat Commun. 2020 Aug 27;11:4293. doi: 10.1038/s41467-020-18136-x (PMC7453021; doi:10.1038/s41467-020-18136-x)
Supplement: Supplementary file 1 — Supplementary Information [file 41467_2020_18136_MOESM1_ESM.pdf]

Supplementary Information for

Copper(I)-catalyzed diastereo- and enantio-selective construction of optically pure exocyclic allenes

He et al.

Supplementary Methods

General information

All solvents were dried before use following the standard procedures. Unless otherwise indicated, all starting materials purchased from commercial suppliers were used without further purification. ^{1}H and ^{13}C NMR spectra were recorded on a Brüker Advance 400 spectrometer (^{1}H: 400MHz, ^{13}C: 100 MHz). Chemical shifts (δ) for ^{1}H and ^{13}C NMR spectra are given in ppm relative to TMS, The residual solvent signals were used as references for ^{1}H and ^{13}C NMR spectra and the chemical shifts converted to the TMS scale ($CDCl_3$: 7.26 ppm for ^{1}H NMR and 77.16 ppm for ^{13}C NMR). Data are reported as follows: chemical shift, integration, multiplicity (s = singlet, d = doublet, t = triplet, q = quartet, br = broad, m = multiplet), and coupling constant (Hz). Optical rotations were measured on a JASCOP-1030 polarimeter. Infrared (IR) spectra were recorded on Nicolet iN 10 MX. High resolution mass spectrometry were recorded on Agilent 1200/G6100A.

Substrate preparation

General procedures for the preparation of 1,3-enynes (1a-1r).

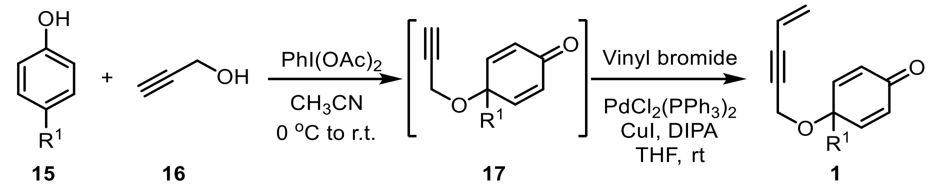

A well-stirred solution of 4-substituted phenol **15** (40.0 mmol) in propargyl alcohol **16** (16.3 mL, 240.0 mmol, 6 equiv) and acetonitrile (5 ml) was cooled to 0 °C and treated with phenyliodine diacetate (PIDA, 19.32 g, 60 mmol, 1.5 equiv) in several portions. The resulting mixture was warmed to room temperature and stirred for 30 mins. The reaction mixture was diluted with water (300 mL) and extracted with ethyl acetate (100 mL×3). The combined organic phases were washed with brine (200 mL), dried over anhydrous Na_2SO_4 and concentrated under reduced pressure. The residue was purified roughly by flash column chromatography using petroleum ether/ethyl acetate eluent to afford the crude product **17**.

A dried Schlenk flask was charged with bis(triphenylphosphine)palladium(II) dichloride (280 mg, 0.4 mmol, 0.01 equiv) and copper(I) iodide (150 mg, 0.8 mmol, 0.02 equiv), backfilled with argon for 3 times, then a mixture of crude product **17** in anhydrous THF (20 ml), diisopropylamine (11 ml, 80 mmol, 2.0 equiv) and vinyl bromide (1.0 M, 60 ml, 60 mmol, 1.5 equiv) was added. The reaction was monitored by TLC. Upon completion, the reaction was filtered through Celite® and the filtrate was evaporated under reduced

pressure and purified by flash column chromatography using petroleum ether/ethyl acetate eluent to afford the desired product **1**.

4-Methyl-4-(pent-4-en-2-yn-1-yloxy)cyclohexa-2,5-dien-1-one (1a)

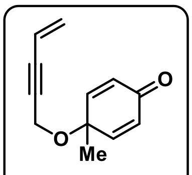

1a

yellow oil, 15% yield. **^{1}H NMR** (400 MHz, $CDCl_3$) δ (ppm) 6.83 (d, *J* = 10.2 Hz, 2H), 6.30 (d, *J* = 10.2 Hz, 2H), 5.78 (ddt, *J* = 17.6, 11.0, 1.9 Hz, 1H), 5.63 (dd, *J* = 17.6, 2.1 Hz, 1H), 5.49 (dd, *J* = 11.0, 2.1 Hz, 1H), 4.10 (d, *J* = 1.8 Hz, 2H), 1.47 (s, 3H). **^{13}C NMR** (100 MHz, $CDCl_3$) δ (ppm) 185.0, 150.9, 130.5, 127.8, 116.5, 86.3, 85.5, 73.2, 54.5, 26.4. **HRMS (DART)**: $[M+H]^{\oplus}$ calcd for $C_{12}H_{13}O_2^{\oplus}$ 189.0910, found 189.0910. **IR (KBr)** *ν* (cm^{-1}) 2980, 2929, 2856, 1706, 1674, 1631, 1605, 1391, 1382, 1301, 1181, 1080, 1045, 1021, 861, 705.

4-Ethyl-4-(pent-4-en-2-yn-1-yloxy)cyclohexa-2,5-dien-1-one (1b)

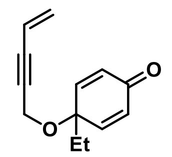

1b

yellow oil, 17% yield. **^{1}H NMR** (400 MHz, $CDCl_3$) δ (ppm) 6.76 (d, *J* = 10.2 Hz, 2H), 6.36 (d, *J* = 10.2 Hz, 2H), 5.78 (ddt, *J* = 17.5, 11.0, 1.8 Hz, 1H), 5.62 (dd, *J* = 17.6, 2.0 Hz, 1H), 5.47 (dd, *J* = 11.0, 2.1 Hz, 1H), 4.12 (d, *J* = 1.7 Hz, 2H), 1.81 (q, *J* = 7.6 Hz, 2H), 0.82 (t, *J* = 7.6 Hz, 3H). **^{13}C NMR** (100 MHz, $CDCl_3$) δ (ppm) 185.5, 150.2, 131.8, 127.9, 116.6, 86.5, 85.5, 77.1, 54.4, 32.4, 8.0. **HRMS (EI)**: $[M]^{\oplus}$ calcd for $C_{13}H_{14}O_2^{\oplus}$ 202.0994, found 202.0999. **IR (KBr)** *ν* (cm^{-1}) 2971, 2936, 2880, 1668, 1633, 1605, 1457, 1392, 1379, 1256, 1173, 1096, 1055, 1027, 974, 921, 856, 683.

4-Isopropyl-4-(pent-4-en-2-yn-1-yloxy)cyclohexa-2,5-dien-1-one (1c)

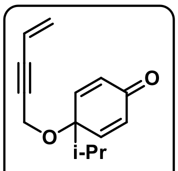

1c

yellow oil, 20% yield. **^{1}H NMR** (400 MHz, $CDCl_3$) δ (ppm) 6.78 (d, *J* = 10.3 Hz, 2H), 6.39 (d, *J* = 10.3 Hz, 2H), 5.78 (ddt, *J* = 17.5, 11.0, 1.9 Hz, 1H), 5.62 (dd, *J* = 17.6, 2.1 Hz, 1H), 5.48 (dd, *J* = 11.0, 2.1 Hz, 1H), 4.11 (d, *J* = 1.8 Hz, 2H), 2.09 – 1.98 (m, 1H), 0.93 (d, *J* = 6.9 Hz, 6H). **^{13}C NMR** (100 MHz, $CDCl_3$) δ (ppm) 185.6, 149.4, 132.3, 127.8, 116.7, 86.8, 85.3, 79.0, 54.3, 36.7, 17.2. **HRMS (DART)**: $[M+H]^{\oplus}$ calcd for $C_{14}H_{17}O_2^{\oplus}$ 217.1223, found 217.1223. **IR (KBr)** *ν* (cm^{-1}) 2966, 1669, 1630, 1605, 1385, 1270, 1167, 1050, 974, 922, 854.

4-(*tert*-Butyl)-4-(pent-4-en-2-yn-1-yloxy)cyclohexa-2,5-dien-1-one (1d)

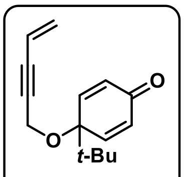

1d

white solid, 10% yield. mp 60 °C. **^{1}H NMR** (400 MHz, $CDCl_3$) δ (ppm) 6.98 – 6.91 (m, 2H), 6.43 – 6.35 (m, 2H), 5.79 (ddt, *J* = 17.5, 11.0, 1.8 Hz, 1H), 5.62 (dd, *J* = 17.6, 2.0 Hz, 1H), 5.48 (dd, *J* = 11.0, 2.1 Hz, 1H), 4.10 (d, *J* = 1.7 Hz, 2H), 1.02 (s, 9H). **^{13}C NMR** (100 MHz, $CDCl_3$) δ (ppm) 185.1, 149.8, 132.3, 127.6, 116.8, 87.2, 85.1, 80.4, 54.5, 39.7, 25.8. **HRMS**

(ESI): [2M+Na]$^{\oplus}$ calcd for $C_{30}H_{36}O_4Na^{\oplus}$ 483.2506, found 483.2506. **IR (KBr)** *ν* (cm^{-1}) 2967, 2360, 2342, 1670, 1628, 1381, 1176, 1045, 923, 861.

1-(Pent-4-en-2-yn-1-yloxy)-[1,1'-bi(cyclohexane)]-2,5-dien-4-one (1e)

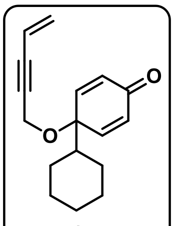

1e

yellow solid, 22% yield. mp 74 ºC. **^{1}H NMR** (400 MHz, $CDCl_3$) δ (ppm) 6.79 (d, *J* = 10.3 Hz, 2H), 6.37 (d, *J* = 10.3 Hz, 2H), 5.79 (ddt, *J* = 17.6, 11.0, 1.9 Hz, 1H), 5.63 (dd, *J* = 17.6, 2.2 Hz, 1H), 5.48 (dd, *J* = 11.0, 2.2 Hz, 1H), 4.10 (d, *J* = 1.9 Hz, 2H), 1.89 (d, *J* = 11.9 Hz, 2H), 1.81 – 1.61 (m, 4H), 1.28 – 1.00 (m, 3H), 0.92 (qd, *J* = 12.5, 3.0 Hz, 2H). **^{13}C NMR** (100 MHz, $CDCl_3$) δ (ppm) 185.8, 149.8, 132.1, 127.8, 116.7, 86.8, 85.3, 78.7, 54.1, 46.7, 27.5, 26.5. **HRMS (ESI)**: [2M+Na]$^{\oplus}$ calcd for $C_{34}H_{40}O_4Na^{\oplus}$ 535.2819, found 535.2808. **IR (KBr)** *ν* (cm^{-1}) 2929, 2854, 1670, 1630, 1051, 1043, 865.

4-(Adamantan-1-yl)-4-(pent-4-en-2-yn-1-yloxy)cyclohexa-2,5-dien-1-one (1f)

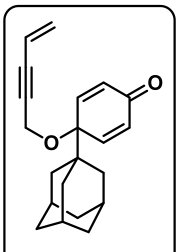

1f

yellow solid, 16% yield. mp 113 ºC. **^{1}H NMR** (400 MHz, $CDCl_3$) δ (ppm) 6.98 (d, *J* = 10.3 Hz, 2H), 6.39 (d, *J* = 10.3 Hz, 2H), 5.86 – 5.73 (m, 1H), 5.63 (dd, *J* = 17.6, 1.8 Hz, 1H), 5.48 (dd, *J* = 11.0, 1.9 Hz, 1H), 4.09 (d, *J* = 1.5 Hz, 2H), 1.99 (s, 3H), 1.74 (d, *J* = 2.1 Hz, 6H), 1.64 (dd, *J* = 21.2, 10.2 Hz, 6H). **^{13}C NMR** (100 MHz, $CDCl_3$) δ (ppm) 185.2, 149.9, 132.3, 127.6, 116.8, 87.3, 85.1, 80.7, 54.2, 42.3, 37.0, 37.0, 28.9. **HRMS (EI)**: [M]$^{\oplus}$ calcd for $C_{21}H_{24}O_2^{\oplus}$ 308.1776, found 308.1780. **IR (KBr)** *ν* (cm^{-1}) 2954, 2921, 2901, 2884, 2845, 1665, 1624, 1447, 1390, 1372, 1342, 1290, 1186, 1058, 1029, 970, 921, 873, 816.

4-(Pent-4-en-2-yn-1-yloxy)-4-vinylcyclohexa-2,5-dien-1-one (1g)

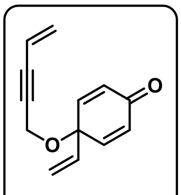

1g

yellow oil, 12% yield. **^{1}H NMR** (400 MHz, $CDCl_3$) δ (ppm) 6.81 (d, *J* = 10.2 Hz, 2H), 6.35 (t, *J* = 6.6 Hz, 2H), 5.84 – 5.70 (m, 2H), 5.64 (dd, *J* = 17.6, 2.1 Hz, 1H), 5.53 – 5.39 (m, 2H), 5.27 (d, *J* = 10.6 Hz, 1H), 4.22 (d, *J* = 1.7 Hz, 2H). **^{13}C NMR** (100 MHz, $CDCl_3$) δ (ppm) 185.3, 148.6, 135.8, 130.6, 128.0, 117.6, 116.6, 86.3, 85.8, 76.0, 54.2. **HRMS (EI)**: [M]$^{\oplus}$ calcd for $C_{13}H_{12}O_2^{\oplus}$ 200.0837, found 200.0832. **IR (KBr)** *ν* (cm^{-1}) 1670, 1632, 1389, 1377, 1169, 1125, 1054, 985, 925, 859.

4-Benzyl-4-(pent-4-en-2-yn-1-yloxy)cyclohexa-2,5-dien-1-one (1h)

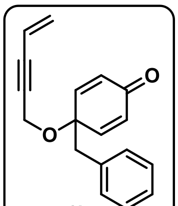

1h

yellow oil, 21% yield. **^{1}H NMR** (400 MHz, $CDCl_3$) δ (ppm) 7.23 (t, *J* = 6.0 Hz, 2H), 7.17 – 7.13 (m, 2H), 6.84 – 6.76 (m, 2H), 6.31 – 6.24 (m, 2H), 5.80 (ddt, *J* = 17.6, 11.0, 1.9 Hz, 1H), 5.64 (dd, *J* = 17.6, 2.1 Hz, 1H), 5.50 (dd, *J* = 11.0, 2.1 Hz, 1H), 4.13 (d, *J* = 1.8 Hz, 2H), 3.07 (s, 2H). **^{13}C NMR** (100 MHz, $CDCl_3$) δ (ppm) 185.2, 149.8, 134.5, 131.4, 130.8,

128.2, 127.9, 127.3, 116.6, 86.6, 85.6, 76.4, 54.6, 46.4. **HRMS (EI)**: [M]$^{\oplus}$ calcd for $C_{18}H_{16}O_2^{\oplus}$ 264.1150, found 264.1153. **IR (KBr)** *v* (cm^{-1}) 3061, 3030, 2921, 2858, 1671, 1630, 1603, 1495, 1454, 1383, , 1242, 1196, 1162, 1093, 1053, 929, 861, 764, 704.

1-(Pent-4-en-2-yn-1-yloxy)-[1,1'-biphenyl]-4(1*H*)-one (1i)

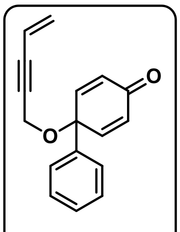
1i

yellow oil, 17% yield. **^{1}H NMR** (400 MHz, $CDCl_3$) δ (ppm) 7.48 (dd, *J* = 8.3, 1.3 Hz, 2H), 7.40 – 7.29 (m, 3H), 6.89 (d, *J* = 10.2 Hz, 2H), 6.40 (d, *J* = 10.1 Hz, 2H), 5.82 (ddt, *J* = 17.6, 11.0, 1.9 Hz, 1H), 5.66 (dd, *J* = 17.6, 2.1 Hz, 1H), 5.51 (dd, *J* = 11.0, 2.1 Hz, 1H), 4.36 (d, *J* = 1.8 Hz, 2H). **^{13}C NMR** (100 MHz, $CDCl_3$) δ (ppm) 185.6, 149.8, 137.8, 130.0, 129.0, 128.6, 128.0, 125.9, 116.6, 86.5, 85.7, 77.0, 54.3. **HRMS (DART)**: [M+H]$^{\oplus}$ calcd for $C_{17}H_{15}O_2^{\oplus}$ 251.1067, found 251.1066. **IR (KBr)** *v* (cm^{-1}) 1669, 1629, 1602, 1489, 1448, 1377, 1274, 1164, 1085, 1060, 1021, 925, 855, 753, 698.

4'-Bromo-1-(pent-4-en-2-yn-1-yloxy)-[1,1'-biphenyl]-4(1*H*)-one (1j)

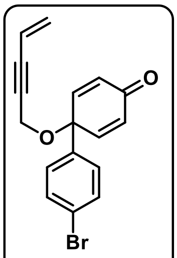
1j

yellow oil, 8% yield. **^{1}H NMR** (400 MHz, $CDCl_3$) δ (ppm) 7.49 (d, *J* = 8.6 Hz, 2H), 7.38 – 7.33 (m, 2H), 6.87 – 6.80 (m, 2H), 6.45 – 6.37 (m, 2H), 5.80 (ddd, *J* = 11.0, 9.7, 6.4 Hz, 1H), 5.66 (dd, *J* = 17.6, 2.1 Hz, 1H), 5.52 (dd, *J* = 11.0, 2.1 Hz, 1H), 4.35 (d, *J* = 1.7 Hz, 2H). **^{13}C NMR** (100 MHz, $CDCl_3$) δ (ppm) 185.3, 149.2, 137.0, 132.1, 130.3, 128.1, 127.7, 122.8, 116.6, 86.3, 85.9, 76.7, 54.4. **HRMS (EI)**: [M]$^{\oplus}$ calcd for $C_{17}H_{13}O_2Br^{\oplus}$ 328.0099, found 328.0101. **IR (KBr)** *v* (cm^{-1}) 2919, 1669, 1629, 1487, 1387, 1164, 1061, 1026, 1008, 924, 855, 825, 530.

4'-Nitro-1-(pent-4-en-2-yn-1-yloxy)-[1,1'-biphenyl]-4(1*H*)-one (1k)

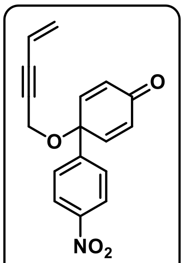
1k

yellow oil, 9% yield. **^{1}H NMR** (400 MHz, $CDCl_3$) δ (ppm) 8.28 – 8.16 (m, 2H), 7.72 – 7.61 (m, 2H), 6.87 – 6.76 (m, 2H), 6.56 – 6.42 (m, 2H), 5.82 (ddt, *J* = 17.6, 11.0, 1.9 Hz, 1H), 5.68 (dd, *J* = 17.6, 2.1 Hz, 1H), 5.54 (dd, *J* = 11.0, 2.2 Hz, 1H), 4.39 (d, *J* = 1.8 Hz, 2H). **^{13}C NMR** (100 MHz, $CDCl_3$) δ (ppm) 184.9, 148.3, 148.0, 145.0, 131.0, 128.4, 127.1, 124.1, 116.4, 86.3, 85.9, 76.8, 54.6. **HRMS (EI)**: [M]$^{\oplus}$ calcd for $C_{17}H_{13}NO_4^{\oplus}$ 295.0845, found 295.0843. **IR (KBr)** *v* (cm^{-1}) 1670, 1630, 1595, 1521, 1349, 1165, 1063, 1026, 1012, 857, 749, 695.

4'-Oxo-1'-(pent-4-en-2-yn-1-yloxy)-1',4'-dihydro-[1,1'-biphenyl]-4-carbonitrile (1l)

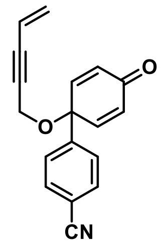
1l

yellow oil, 7% yield. **^{1}H NMR** (400 MHz, $CDCl_3$) δ (ppm) 7.66 (d, *J* = 8.5 Hz, 2H), 7.60 (d, *J* = 8.5 Hz, 2H), 6.81 (d, *J* = 10.1 Hz, 2H), 6.45 (d, *J* = 10.1 Hz, 2H), 5.86 – 5.75 (m, 1H), 5.66 (dd, *J* = 17.6, 2.0 Hz, 1H), 5.53 (dd, *J* = 11.0, 2.1 Hz, 1H), 4.37 (d, *J* = 1.6 Hz, 2H). **^{13}C NMR** (100 MHz, $CDCl_3$) δ (ppm) 184.9, 148.4, 143.1, 132.7, 130.9, 128.3, 126.8,

118.5, 116.4, 112.5, 86.2, 86.0, 76.7, 54.5. **HRMS (EI)**: $[M]^{\oplus}$ calcd for $C_{18}H_{13}NO_2^{\oplus}$ 275.0946, found 275.0944. **IR (KBr)** *v* (cm^{-1}) 2229, 1684, 1670, 1631, 1605, 1499, 1388, 1164, 1063, 1028, 1017, 856, 841, 562.

4-(Pent-4-en-2-yn-1-yloxy)-4-(pyridin-2-yl)cyclohexa-2,5-dien-1-one (1m)

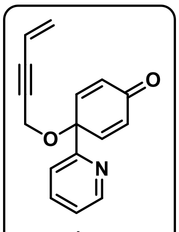
1m

yellow oil, 6% yield. **^{1}H NMR** (400 MHz, $CDCl_3$) δ (ppm) 8.53 (d, *J* = 4.7 Hz, 1H), 7.76 (dd, *J* = 5.0, 1.4 Hz, 2H), 7.25 – 7.21 (m, 1H), 6.98 – 6.85 (m, 2H), 6.46 (d, *J* = 10.2 Hz, 2H), 5.81 (ddt, *J* = 17.6, 11.0, 1.9 Hz, 1H), 5.65 (dd, *J* = 17.6, 2.1 Hz, 1H), 5.51 (dd, *J* = 11.0, 2.1 Hz, 1H), 4.37 (d, *J* = 1.8 Hz, 2H). **^{13}C NMR** (100 MHz, $CDCl_3$) δ (ppm) 185.6, 158.3, 149.4, 148.2, 137.5, 131.2, 128.0, 123.6, 121.5, 116.6, 86.4, 85.8, 78.0, 54.2. **HRMS (DART)**: $[M+H]^{\oplus}$ calcd for $C_{16}H_{14}NO_2^{\oplus}$ 252.1019, found 252.1019. **IR (KBr)** *v* (cm^{-1}) 1745, 1670, 1630, 1606, 1585, 1464, 1432, 1389, 1377, 1276, 1227, 1167, 1099, 1063, 1027, 924, 854, 791, 758, 712.

1-(Pent-4-en-2-yn-1-yloxy)-[1,1'-bi(cyclohexane)]-2,5-diene-4,4'-dione (1n)

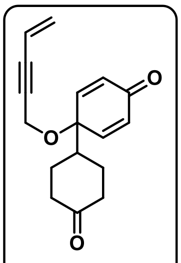
1n

yellow oil, 12% yield. **^{1}H NMR** (400 MHz, $CDCl_3$) δ (ppm) 6.84 – 6.76 (m, 2H), 6.47 – 6.38 (m, 2H), 5.79 (ddt, *J* = 17.5, 11.0, 1.9 Hz, 1H), 5.64 (dd, *J* = 17.6, 2.1 Hz, 1H), 5.50 (dd, *J* = 11.0, 2.1 Hz, 1H), 4.14 (d, *J* = 1.8 Hz, 2H), 2.46 – 2.11 (m, 8H), 1.51 – 1.36 (m, 1H). **^{13}C NMR** (100 MHz, $CDCl_3$) δ (ppm) 210.5, 185.1, 148.4, 132.7, 128.0, 116.6, 86.3, 85.6, 77.8, 54.3, 44.8, 40.6, 26.9. **HRMS (DART)**: $[M+H]^{\oplus}$ calcd for $C_{17}H_{19}O_3^{\oplus}$ 271.1329, found 271.1329. **IR (KBr)** *v* (cm^{-1}) 2954, 2874, 1715, 1669, 1628, 1373, 1328, 1214, 1192, 1170, 1063, 1048, 922, 872, 854.

Methyl 3-(4-oxo-1-(pent-4-en-2-yn-1-yloxy)cyclohexa-2,5-dien-1-yl)propanoate (1o)

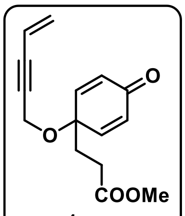
1o

colorless oil, 12% yield. **^{1}H NMR** (400 MHz, $CDCl_3$) δ (ppm) 6.80 (d, *J* = 10.2 Hz, 2H), 6.37 (d, *J* = 10.2 Hz, 2H), 5.79 (ddt, *J* = 17.5, 10.9, 1.9 Hz, 1H), 5.64 (dd, *J* = 17.6, 2.1 Hz, 1H), 5.51 (dd, *J* = 11.0, 2.1 Hz, 1H), 4.13 (d, *J* = 1.8 Hz, 2H), 3.65 (s, 3H), 2.33 (t, *J* = 7.8 Hz, 2H), 2.14 (t, *J* = 7.8 Hz, 2H). **^{13}C NMR** (100 MHz, $CDCl_3$) δ (ppm) 185.0, 173.0, 149.3, 131.9, 128.0, 116.6, 77.4, 54.5, 52.0, 34.3, 29.9, 29.5, 28.6. **HRMS (EI)**: $[M]^{\oplus}$ calcd for $C_{15}H_{16}O_4^{\oplus}$ 260.1049, found 260.1042. **IR (KBr)** *v* (cm^{-1}) 2951, 2922, 1736, 1671, 1631, 1438, 1382, 1200, 1172, 1062, 862.

4-(3-((*tert*-Butyldimethylsilyl)oxy)propyl)-4-(pent-4-en-2-yn-1-yloxy)cyclohexa-2,5-dien-1-one (1p)

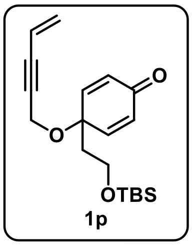

yellow oil, 17% yield. **^{1}H NMR** (400 MHz, $CDCl_3$) δ (ppm) 6.88 (d, *J* = 10.2 Hz, 2H), 6.32 (d, *J* = 10.2 Hz, 2H), 5.79 (ddt, *J* = 17.6, 11.0, 1.8 Hz, 1H), 5.64 (dd, *J* = 17.6, 2.0 Hz, 1H), 5.49 (dd, *J* = 11.0, 2.1 Hz, 1H), 4.11 (d, *J* = 1.7 Hz, 2H), 3.69 (t, *J* = 6.1 Hz, 2H), 2.00 (t, *J* = 6.1 Hz, 2H), 0.86 (s, 9H), 0.01 (s, 6H). **^{13}C NMR** (100 MHz, $CDCl_3$) δ (ppm) 185.6, 150.4, 130.8, 127.9, 116.6, 86.5, 85.5, 75.1, 58.0, 54.1, 43.0, 26.0, 18.3, -5.3. **HRMS (DART)**: $[M+H]^{\oplus}$ calcd for $C_{19}H_{29}O_3Si^{\oplus}$ 333.1880, found 333.1879. **IR (KBr)** *ν* (cm^{-1}) 2954, 2885, 2857, 1672, 1633, 1472, 1392, 1253, 1087, 1053, 923, 811, 777.

4-(3-Chloropropyl)-4-(pent-4-en-2-yn-1-yloxy)cyclohexa-2,5-dien-1-one (1q)

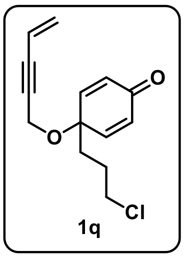

yellow oil, 14% yield. **^{1}H NMR** (400 MHz, $CDCl_3$) δ (ppm) 6.88 – 6.72 (m, 2H), 6.48 – 6.30 (m, 2H), 5.79 (ddt, *J* = 17.5, 11.0, 1.9 Hz, 1H), 5.64 (dd, *J* = 17.6, 2.1 Hz, 1H), 5.50 (dd, *J* = 11.0, 2.1 Hz, 1H), 4.13 (d, *J* = 1.8 Hz, 2H), 3.51 (t, *J* = 6.4 Hz, 2H), 1.99 – 1.88 (m, 2H), 1.79 – 1.75 (m, 2H). **^{13}C NMR** (100 MHz, $CDCl_3$) δ (ppm) 185.1, 149.7, 131.8, 128.0, 116.6, 86.3, 85.7, 75.8, 54.4, 44.7, 37.0, 26.9. **HRMS (DART)**: $[M+H]^{\oplus}$ calcd for $C_{14}H_{16}O_2Cl^{\oplus}$ 251.0833, found 251.0834. **IR (KBr)** *ν* (cm^{-1}) 2958, 2925, 2857, 1671, 1631, 1605, 1443, 1383, 1307, 1243, 1171, 1054, 922, 867, 651.

4-(3-Bromopropyl)-4-(pent-4-en-2-yn-1-yloxy)cyclohexa-2,5-dien-1-one (1r)

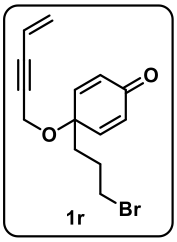

yellow oil, 17% yield. **^{1}H NMR** (400 MHz, $CDCl_3$) δ (ppm) 6.80 (d, *J* = 10.2 Hz, 2H), 6.36 (d, *J* = 10.2 Hz, 2H), 5.78 (ddt, *J* = 17.5, 11.0, 1.8 Hz, 1H), 5.63 (dd, *J* = 17.6, 2.1 Hz, 1H), 5.49 (dd, *J* = 11.0, 2.1 Hz, 1H), 4.12 (d, *J* = 1.7 Hz, 2H), 3.36 (t, *J* = 6.3 Hz, 2H), 1.92 (ddt, *J* = 8.4, 5.3, 2.7 Hz, 2H), 1.88 – 1.80 (m, 2H). **^{13}C NMR** (100 MHz, $CDCl_3$) δ (ppm) 185.0, 149.6, 131.8, 127.9, 116.5, 86.3, 85.6, 75.7, 54.4, 38.2, 33.1, 26.9. **HRMS (EI)**: $[M]^{\oplus}$ calcd for $C_{14}H_{15}O_2Br^{\oplus}$ 294.0255, found 294.0261. **IR (KBr)** *ν* (cm^{-1}) 2924, 2856, 1671, 1631, 1605, 1439, 1382, 1275, 1257, 1233, 1169, 1059, 921, 864.

Preparation of 1,3-enynes (1s-1w).

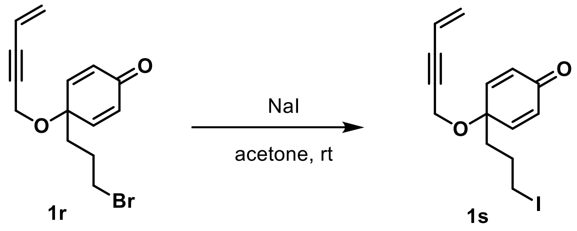

4-(3-Iodopropyl)-4-(pent-4-en-2-yn-1-yloxy)cyclohexa-2,5-dien-1-one (1s) To a solution of **1r** (0.59 g, 2 mmol) in acetone (10 ml) was added NaI (3.0 g, 20 mmol, 10.0 equiv), and the resulting mixture was stirred at room temperature. The reaction was monitored by crude ^{1}H-NMR analysis. Upon completion, the reaction was filtered through Celite® and the filtrate was evaporated under reduced pressure and purified by flash column chromatography (PE/EA = 50/1) to afford the desired product **1s** (0.53 g) as a yellow oil.

78% yield. **^{1}H NMR** (400 MHz, $CDCl_3$) δ (ppm) 6.79 (d, *J* = 10.2 Hz, 2H), 6.36 (t, *J* = 6.6 Hz, 2H), 5.78 (ddt, *J* = 17.5, 11.0, 1.8 Hz, 1H), 5.63 (dd, *J* = 17.6, 2.1 Hz, 1H), 5.49 (dd, *J* = 11.0, 2.1 Hz, 1H), 4.11 (d, *J* = 1.7 Hz, 2H), 3.13 (t, *J* = 6.6 Hz, 2H), 1.92 – 1.84 (m, 2H), 1.84 – 1.74 (m, 2H). **^{13}C NMR** (100 MHz, $CDCl_3$) δ (ppm) 185.0, 149.6, 131.8, 128.0, 116.6, 86.3, 85.7, 75.6, 54.4, 40.4, 27.6, 5.7. **HRMS (EI)**: $[M]^{\oplus}$ calcd for $C_{14}H_{15}O_2I^{\oplus}$ 342.0117, found 342.0111. **IR (KBr)** *ν* (cm^{-1}) 3361, 2923, 2852, 1668, 1631, 1443, 1427, 1384, 1263, 1216, 1053, 863, 821, 736, 701.

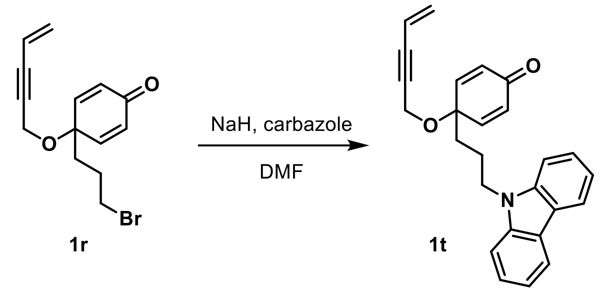

4-(3-(9*H*-carbazol-9-yl)propyl)-4-(pent-4-en-2-yn-1-yloxy)cyclohexa-2,5-dien-1-one (1t) A solution of carbazole (418 mg, 2.5 mmol, 1.25 equiv) in DMF (5 ml) was cooled to 0 °C and treated with NaH (120 mg, 3 mmol, 1.5 equiv) in several portions. After the reaction was further stirred at 0 °C for 30 mins, a mixture of **1r** in DMF (5 ml) was added, and the resulting mixture was stirred at room temperature for 12 h. Upon completion, the reaction was quenched with H_2O (20 ml), filtered through Celite® and the filtrate was extracted with DCM (20 mL x 3). The combined organic phases were washed with saturated lithium chloride solution for 3 times, dried over anhydrous Na_2SO_4, filtered and concentrated in vacuo. The residue was purified by flash column chromatography (PE/EA = 5/1) to afford **1t** (420 mg) as a yellow solid.

55% yield. mp 88 °C. **^{1}H NMR** (400 MHz, $CDCl_3$) δ (ppm) 8.10 (d, *J* = 7.7 Hz, 2H), 7.46 (t, *J* = 7.7 Hz, 2H), 7.36 (d, *J* = 8.2 Hz, 2H), 7.23 (d, *J* = 7.2 Hz, 2H), 6.71 (d, *J* = 10.2 Hz, 2H), 6.31 (d, *J* = 10.2 Hz, 2H), 5.77 (dd, *J* = 17.6, 11.0 Hz, 1H), 5.62 (dd, *J* = 17.6, 2.0 Hz, 1H), 5.49 (dd, *J* = 11.0, 2.0 Hz, 1H), 4.32 (t, *J* = 6.4 Hz, 2H), 4.07 (d, *J* = 1.6 Hz, 2H), 1.94 – 1.80 (m, 4H). **^{13}C NMR** (100 MHz, $CDCl_3$) δ (ppm) 185.1, 149.6, 140.4, 131.8, 128.0, 125.9, 123.0, 120.6, 119.1, 116.6, 108.6, 86.3, 85.7, 77.4, 75.9, 61.3, 54.4 42.9,

37.0, 23.4. **HRMS (ESI)**: $[M+H]^{\oplus}$ calcd for $C_{26}H_{24}NO_2^{\oplus}$ 382.1802, found 382.1796. **IR (KBr)** *v* (cm^{-1}) 3049, 2924, 2855, 1670, 1628, 1596, 1484, 1462, 1453, 1381, 1347, 1326, 1154, 1053, 924, 862, 751, 724.

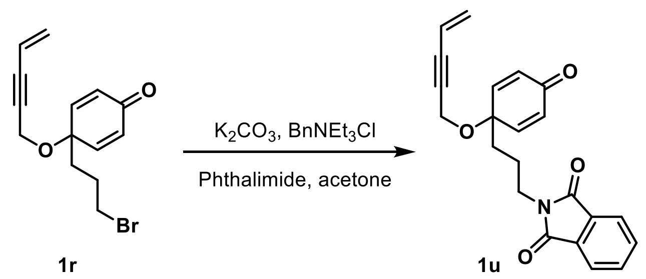

2-(3-(4-Oxo-1-(pent-4-en-2-yn-1-yloxy)cyclohexa-2,5-dien-1-yl)propyl)isoindoline-1,3-dione (1u) To a stirred solution of phthalimide (295 mg, 2.0 mmol), K_2CO_3 (830 mg, 6.0 mmol, 3.0 equiv), and $BnNEt_3Cl$ (45 mg, 0.2 mmol, 0.1 equiv) in acetone (10 ml), **1r** (737 mg, 2.5 mmol, 1.25 equiv) was added in one portion and the resulting mixture was stirred at 50 °C for 10 h. Upon completion, the reaction was filtered through Celite® and the filtrate was concentrated in vacuo. The residue was purified by flash column chromatography (PE/EA = 8/1) to afford **1u** (504 mg) as a yellow solid.

70% yield. mp 90 °C. **^{1}H NMR** (400 MHz, $CDCl_3$) δ (ppm) 7.83 (dd, *J* = 5.5, 3.1 Hz, 2H), 7.71 (dd, *J* = 5.5, 3.0 Hz, 2H), 6.85 – 6.72 (m, 2H), 6.36 – 6.31 (m, 2H), 5.78 (ddt, *J* = 17.6, 11.0, 1.9 Hz, 1H), 5.63 (dd, *J* = 17.6, 2.2 Hz, 1H), 5.49 (dd, *J* = 11.0, 2.2 Hz, 1H), 4.10 (d, *J* = 1.8 Hz, 2H), 3.67 (t, *J* = 7.1 Hz, 2H), 1.89 – 1.77 (m, 2H), 1.71 – 1.62 (m, 2H). **^{13}C NMR** (100 MHz, $CDCl_3$) δ (ppm) 185.2, 168.4, 149.7, 134.2, 132.1, 131.8, 127.9, 123.4, 116.6, 86.4, 85.6, 77.4, 75.9, 54.4, 37.8, 36.7, 23.0. **HRMS (ESI)**: $[M+NH_4]^{\oplus}$ calcd for $C_{22}H_{23}N_2O_4^{\oplus}$ 379.1652, found 379.1648. **IR (KBr)** *v* (cm^{-1}) 2935, 2859, 1771, 1713, 1670, 1631, 1437, 1397, 1361, 1187, 1171, 1057, 925, 885, 862, 794, 720, 530.

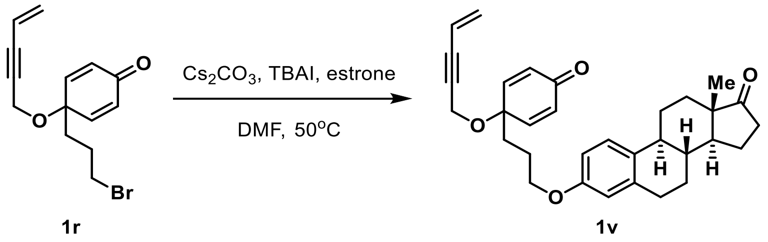

(8*R*,9*S*,13*S*,14*S*)-13-Methyl-3-(3-(4-oxo-1-(pent-4-en-2-yn-1-yloxy)cyclohexa-2,5-dien-1-yl)propoxy)-6,7,8,9,11,12,13,14,15,16-decahydro-17*H*-cyclopenta[a]phenanthren-17-one (1v) To a stirred solution of estrone (1.30 g, 4.8 mmol), Cs_2CO_3 (1.88 g, 5.76 mmol, 1.2 equiv), TBAI (177 mg, 0.48 mmol, 0.1 equiv) in DMF (12 ml), **1r** (1.55 g, 5.28 mmol, 1.1 equiv) was added in one portion and the resulting mixture was stirred at 50 °C for 24 h. Upon completion, the reaction was quenched with H_2O (20 ml), filtered through Celite® and the filtrate was extracted with DCM (20 mL x 3). The combined organic phases were washed

with saturated lithium chloride solution for 3 times, dried over anhydrous Na_2SO_4, filtered and concentrated in vacuo. The residue was purified by flash column chromatography (PE/EA = 5/1) to afford **1v** (1.80 g) as a yellow viscous oil.

20% yield. $[\alpha]_D^{25.0}$ 55.51 (*c* 2.00, $CHCl_3$). **^{1}H NMR** (400 MHz, $CDCl_3$) δ (ppm) 7.18 (d, *J* = 8.6 Hz, 1H), 6.84 (d, *J* = 10.1 Hz, 2H), 6.70 – 6.63 (m, 1H), 6.60 (s, 1H), 6.38 (d, *J* = 10.0 Hz, 2H), 5.80 (dd, *J* = 17.5, 11.0 Hz, 1H), 5.64 (dd, *J* = 17.5, 1.8 Hz, 1H), 5.50 (dd, *J* = 11.0, 1.8 Hz, 1H), 4.15 (d, *J* = 1.3 Hz, 2H), 3.90 (t, *J* = 6.1 Hz, 2H), 2.92 – 2.81 (m, 2H), 2.50 (dd, *J* = 18.8, 8.6 Hz, 1H), 2.38 (d, *J* = 9.5 Hz, 1H), 2.24 (s, 1H), 2.07 – 1.89 (m, 6H), 1.80 – 1.69 (m, 2H), 1.66 – 1.39 (m, 6H), 0.90 (s, 3H). **^{13}C NMR** (100 MHz, $CDCl_3$) δ (ppm) 221.1, 185.3, 156.9, 150.0, 137.9, 132.3, 131.7, 127.9, 126.5, 116.6, 114.6, 112.2, 86.4, 85.6, 76.1, 67.4, 54.4, 50.5, 48.1, 44.1, 38.5, 36.2, 36.0, 31.7, 29.8, 26.7, 26.0, 23.8, 21.7, 14.0. **HRMS (ESI)**: $[M+H]^{\oplus}$ calcd for $C_{32}H_{37}O_4^{\oplus}$ 485.2686, found 485.2688. **IR (KBr)** *ν* (cm^{-1}) 2928, 2862, 1737, 1671, 1631, 1608, 1499, 1471, 1453, 1373, 1238, 1163, 1055, 862, 819, 735.

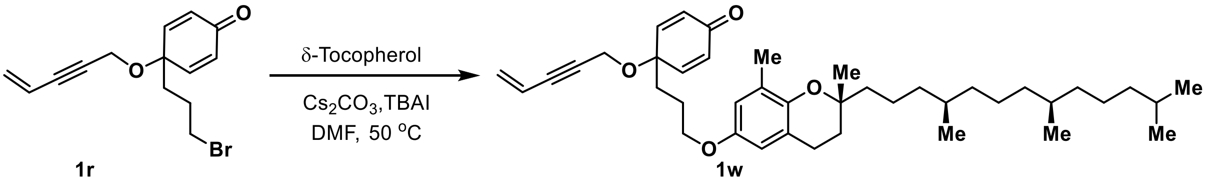

4-(3-(((*R*)-2,8-Dimethyl-2-((4*R*,8*R*)-4,8,12-trimethyltridecyl)chroman-6-yl)oxy)propyl)-4-(pent-4-en-2-yn-1-yloxy)cyclohexa-2,5-dien-1-one (1w) To a stirred solution of δ-tocopherol (482 mg, 1.2 mmol, 1.0 equiv), Cs_2CO_3 (508 mg, 1.56 mmol, 1.3 equiv), TBAI (44 mg, 0.12 mmol, 0.1 equiv) in DMF (6 ml), **1r** (423 g, 1.44 mmol, 1.2 equiv) was added in one portion and the resulting mixture was stirred at 50 °C for 16 h. Upon completion, the reaction was quenched with H_2O (20 ml), filtered through Celite® and the filtrate was extracted with DCM (20 mL x 3). The combined organic phases were washed with saturated lithium chloride solution for 3 times, dried over anhydrous Na_2SO_4, filtered and concentrated in vacuo. The residue was purified by flash column chromatography (PE/EA = 20/1) to afford **1w** (251 mg) as a yellow oil.

34% yield. $[\alpha]_D^{25.0}$ 2.72 (*c* 1.39, $CHCl_3$). **^{1}H NMR** (400 MHz, CDCl3) δ (ppm) 6.84 (d, *J* = 10.2 Hz, 2H), 6.51 (d, *J* = 2.7 Hz, 1H), 6.38 (d, *J* = 10.2 Hz, 3H), 5.80 (ddt, *J* = 17.6, 11.0, 1.9 Hz, 1H), 5.64 (dd, *J* = 17.6, 2.1 Hz, 1H), 5.50 (dd, *J* = 11.0, 2.2 Hz, 1H), 4.14 (d, *J* = 1.8 Hz, 2H), 3.84 (t, *J* = 6.1 Hz, 2H), 2.76 – 2.62 (m, 2H), 2.13 (s, 3H), 1.99 – 1.93 (m, 2H), 1.82 – 1.66 (m, 4H), 1.57 – 1.48 (m, 3H), 1.40 – 1.22 (m, 15H), 1.16 – 1.04 (m, 6H), 0.87 – 0.83 (m, 12H). **^{13}C NMR** (100 MHz, CDCl3) δ (ppm) 185.3, 151.3, 150.1, 146.3, 131.7, 127.9, 127.3, 121.0, 116.6, 115.4, 111.9, 86.4, 85.5, 76.1, 75.7, 67.9, 54.3, 40.0, 39.4, 37.5,

37.5, 37.3, 36.2, 32.9, 32.8, 31.4, 28.1, 24.9, 24.5, 24.2, 23.9, 22.8, 22.7, 21.1, 19.8, 19.7, 16.3. **HRMS (ESI)**: $[M+H]^{\oplus}$ calcd for $C_{41}H_{61}O_4^{\oplus}$ 617.4564, found 617.4571. **IR (KBr)** *v* (cm^{-1}) 2925, 2865, 1671, 1632 1607, 1469, 1371, 1219, 1154, 1057, 859.

Preparation of 1,3-enynes (1x-1y).

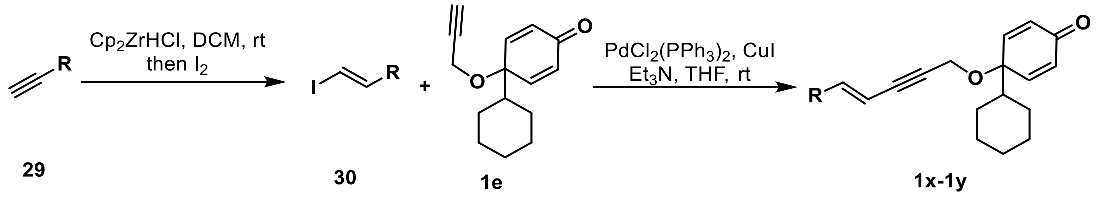

A dried flask was charged with Cp_2ZrHCl (12 mmol, 1.2 equiv), backfilled with argon for 3 times, then terminal alkyne **29** (10 mmol, l.0 equiv) and DCM (30 mL) were added. The mixture was stirred at room temperature for 30 mins. Next, I_2 (12.5 mmol, l.25 equiv)was added to the mixture in portion. And the final mixture stirred for 30 mins. Upon completion, the reaction was quenched by $Na_2S_2O_3$ aqueous solution and extracted by water and DCM, then dried by anhydrous Na_2SO_4 and filtrated. The solution then evaporated under reduced pressure to afford the crude product **30**.

A dried flask was charged with **1e** (4 mmol, 1.0 equiv), bis(triphenylphosphine)palladium(II) dichloride (280 mg, 0.4 mmol, 0. 1 equiv) and copper(I) iodide (150 mg, 0.8 mmol, 0.2 equiv), backfilled with argon for 3 times, then a mixture of crude product **30** in anhydrous THF (12 ml) and Et_3N (6 mmol, 2.0 equiv) was added. The reaction was stirred at rt and monitored by TLC. Upon completion, the reaction was filtered through Celite® and the filtrate was evaporated under reduced pressure and purified by flash column chromatography using petroleum ether/ethyl acetate eluent to afford the desired product **1x-1y**.

(*E*)-1-(Dec-4-en-2-yn-1-yloxy)-[1,1'-bi(cyclohexane)]-2,5-dien-4-one (1x)

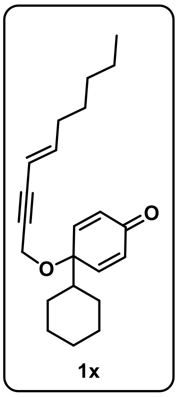

colorless oil, 10% yield. **^{1}H NMR** (400 MHz, $CDCl_3$) δ (ppm) 6.84 – 6.75 (m, 2H), 6.41 – 6.32 (m, 2H), 6.14 (dt, *J* = 15.7, 7.1 Hz, 1H), 5.51 – 5.40 (m, 1H), 4.10 (d, *J* = 1.8 Hz, 2H), 2.16 – 2.02 (m, 2H), 1.90 (d, *J* = 12.1 Hz, 2H), 1.80 – 1.61 (m, 4H), 1.42 – 1.03 (m, 9H), 0.97 – 0.83 (m, 5H). **^{13}C NMR** (100 MHz, $CDCl_3$) δ (ppm) 185.7, 149.9, 146.0, 131.9, 108.9, 85.5, 84.4, 78.6, 54.1, 46.6, 33.1, 31.3, 28.3, 27.4, 26.4, 22.5, 14.0. **HRMS (EI)**: $[M]^{\oplus}$ calcd for $C_{22}H_{30}O_2^{\oplus}$ 326.2240, found 326.2238. **IR (KBr)** *v* (cm^{-1}) 2927, 2854, 1671, 1631, 1451, 1382, 1277, 1169, 1052, 954, 864.

(*E*)-1-((6-(Benzyloxy)hex-4-en-2-yn-1-yl)oxy)-[1,1'-bi(cyclohexane)]-2,5-dien-4-one (1y)

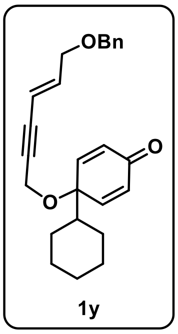

yellow oil, 9% yield. **^{1}H NMR** (400 MHz, $CDCl_3$) δ (ppm) 7.37 – 7.27 (m, 5H), 6.80 (d, *J* = 10.3 Hz, 2H), 6.37 (d, *J* = 10.3 Hz, 2H), 6.20 (dt, *J* = 16.0, 5.4 Hz, 1H), 5.82 – 5.73 (m, 1H), 4.52 (s, 2H), 4.11 (d, *J* = 1.5 Hz, 2H), 4.06 (dd, *J* = 5.4, 1.5 Hz, 2H), 1.90 (d, *J* = 12.5 Hz, 2H), 1.78 – 1.62 (m, 4H), 1.20 (dd, *J* = 25.9, 12.9 Hz, 2H), 1.13 – 1.04 (m, 1H), 0.92 (qd, *J* = 12.5, 2.9 Hz, 2H). **^{13}C NMR** (100 MHz, $CDCl_3$) δ (ppm) 185.7, 149.8, 140.2, 137.9, 132.0, 128.5, 127.8, 127.7, 111.0, 86.8, 84.5, 78.6, 72.4, 69.6, 54.1, 46.6, 27.4, 26.4. **HRMS (EI)**: $[M]^{\oplus}$ calcd for $C_{25}H_{28}O_3^{\oplus}$ 376.2033, found 376.2036. **IR (KBr)** *ν* (cm^{-1}) 2922, 2851, 1668, 1630, 1451, 1356, 1041, 865.

General procedures for the preparation of 1,3-enynes (4a-4c).

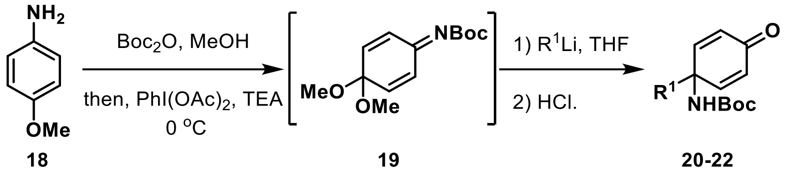

To a stirred solution of *p*-Anisidine **18** (25 g, 200 mmol) in methanol (400 mL), Boc_2O (65 g, 300 mmol, 1.5 equiv) was added in several portions. Triethylamine (83 ml, 600 mmol, 3.0 equiv) was added in one portion after the reaction was stirred at room temperature for 3 h. Subsequently, phenyliodine diacetate (83 g, 260 mmol, 1.3 equiv) was added in several portions after the reaction mixture was cooled to 0°C. Upon completion, the reaction was quenched with saturated aqueous $NaHCO_3$ solution and extracted with DCM (200 mL x 3). The combined organic phases were washed with brine (200 mL), dried over anhydrous Na_2SO_4 and concentrated under reduced pressure to afford the crude product **19**.

To an oven-dried flask charged with crude product **19** (7.6 g, about 30 mmol) and anhydrous THF (50 ml), was added organolithium reagent (45 mmol, 1.5 equiv, Methyllithium for **20**, *n*-Butyllithium for **21**, Phenyllithium for **22**) after the reaction mixture was cooled to -78 °C. The reaction was stirred for 3 h at room temperature and monitored by TLC. Upon completion, the reaction was quenched with a small amount of water under ice water bath. The reaction was stirred for another 12 h at room temperature after its pH was adjusted to pH = 2 with 1N aqueous HCl. The reaction mixture was diluted with water (200 mL) and extracted with DCM (100 mL × 3). The combined organic phases were washed with brine (200 mL), dried

over anhydrous Na_2SO_4 and concentrated under reduced pressure. The residue was purified by flash column chromatography (PE/EA = 50/1) to afford the desired product **20**, **21**, **22**.[1]

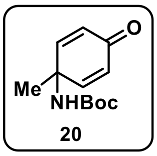

yellow solid, 60% yield. **^{1}H NMR** (400 MHz, $CDCl_3$) δ (ppm) 7.07 (d, *J* = 10.0 Hz, 2H), 6.16 (d, *J* = 10.0 Hz, 2H), 2.33 (d, *J* = 2.4 Hz, 1H), 1.71 (s, 3H), 1.39 (s, 9H).

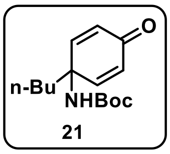

white solid. 62% yield. **^{1}H NMR** (400 MHz, $CDCl_3$) δ (ppm) 6.78 (d, *J* = 9.6 Hz, 2H), 6.28 (d, *J* = 9.6 Hz, 2H), 5.12 (s, 1H), 1.74 − 1.70 (m, 2H), 1.39 (s, 9H), 1.31 − 1.20 (m, 4H), 0.87 (t, *J* = 6.8 Hz, 3H).

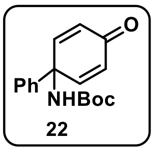

white solid. 58% yield. **^{1}H NMR** (400 MHz, $CDCl_3$) δ (ppm) 7.44 − 7.31 (m, 5H), 7.05 (d, *J* = 10.0 Hz, 2H), 6.31 (d, *J* = 10.0 Hz, 2H), 5.29 (br s, 1H), 1.39 (s, 9H).

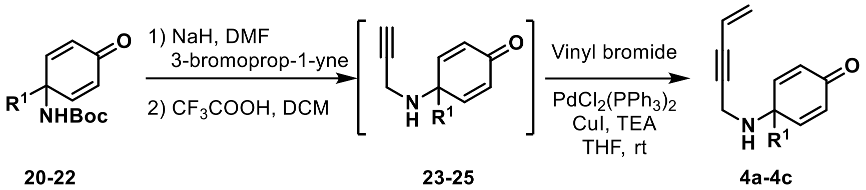

A solution of **20-22** (20 mmol) in DMF (40 ml) was cooled to 0 °C and treated with NaH (960 mg, 40 mmol, 2.0 equiv) in several portions. After the reaction was further stirred at 0 °C for 30 mins, 3-bromoprop-1-yne (2.6 ml, 30 mmol, 1.5 equiv) was added, and the resulting mixture was stirred at room temperature for 12 h. Upon completion, the reaction was quenched with H_2O (200 ml), filtered through Celite® and the filtrate was extracted with DCM (200 mL x 3). The combined organic phases were washed with saturated lithium chloride solution for 3 times, dried over anhydrous Na_2SO_4, filtered and concentrated in vacuo, then DCM (50 ml) was added, trifluoroacetic acid (18.4 ml, 240 mmol, 12.0 equiv) was added dropwise under ice water bath. The reaction was stirred at room temperature for 3 h, quenched with saturated aqueous $NaHCO_3$ solution and extracted with DCM (200 mL x 3). The combined organic phases were washed with brine (200 mL), dried over anhydrous Na_2SO_4 and concentrated under reduced pressure to afford the crude product **23-25**.

A dried Schlenk flask was charged with bis(triphenylphosphine)palladium(II) dichloride (175 mg, 0.25 mmol, 0.012 equiv) and copper(I) iodide (95 mg, 0.5 mmol, 0.025 equiv), backfilled with argon for 3 times, then a mixture of crude product **23-25** in anhydrous THF (20 ml), triethylamine (8.7 ml, 62.50 mmol, 3.125 equiv) and vinyl bromide (1.0 M, 30 ml, 30.0 mmol, 1.5 equiv) was added. The reaction was stirred at room temperature and monitored by TLC. Upon completion, the reaction was filtered through Celite® and the

filtrate was evaporated under reduced pressure and purified by flash column chromatography using petroleum ether/ethyl acetate eluent to afford the desired product **4a-4c** respectively.

4-Methyl-4-(pent-4-en-2-yn-1-ylamino)cyclohexa-2,5-dien-1-one (4a)

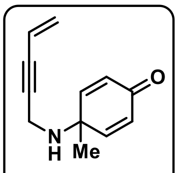

4a

yellow solid, 16% yield. mp 58 °C. **^{1}H NMR** (400 MHz, $CDCl_3$) δ (ppm) 6.84 – 6.71 (m, 2H), 6.39 – 6.20 (m, 2H), 5.75 (ddt, *J* = 17.5, 11.0, 1.9 Hz, 1H), 5.58 (dd, *J* = 17.6, 2.1 Hz, 1H), 5.44 (dd, *J* = 11.0, 2.2 Hz, 1H), 3.34 (d, *J* = 1.8 Hz, 2H), 1.64 (s, 1H), 1.37 (s, 3H). **^{13}C NMR** (100 MHz, $CDCl_3$) δ (ppm) 185.6, 153.9, 129.8, 127.2, 116.9, 88.40, 83.0, 55.5, 34.8, 26.9. **HRMS (ESI)**: $[M+H]^{\oplus}$ calcd for $C_{12}H_{14}NO^{\oplus}$ 188.1070, found 188.1070. **IR (KBr)** *v* (cm^{-1}) 3305, 2961, 2925, 1666, 1626, 1467, 1395, 1383, 1345, 1291, 1182, 1106, 1090, 974, 922, 861, 703.

4-Butyl-4-(pent-4-en-2-yn-1-ylamino)cyclohexa-2,5-dien-1-one (4b)

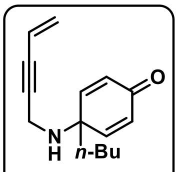

4b

yellow solid, 20% yield. mp 46 °C. **^{1}H NMR** (400 MHz, $CDCl_3$) δ (ppm) 6.76 – 6.64 (m, 2H), 6.38 – 6.25 (m, 2H), 5.76 (ddt, *J* = 17.5, 11.0, 1.9 Hz, 1H), 5.58 (dd, *J* = 17.6, 2.2 Hz, 1H), 5.44 (dd, *J* = 11.0, 2.2 Hz, 1H), 3.35 (d, *J* = 1.6 Hz, 2H), 1.69 – 1.63 (m, 2H), 1.60 (s, 1H), 1.30 – 1.14 (m, 4H), 0.85 (t, *J* = 7.2 Hz, 3H). **^{13}C NMR** (100 MHz, $CDCl_3$) δ (ppm) 186.1, 153.2, 131.0, 127.1, 116.9, 88.5, 82.9, 59.2, 39.8, 34.4, 25.9, 23.00, 13.9. **HRMS (ESI)**: $[M+H]^{\oplus}$ calcd for $C_{15}H_{20}NO^{\oplus}$ 230.1539, found 230.1539. **IR (KBr)** *v* (cm^{-1}) 3307, 2958, 2932, 2862, 1667, 1626, 1466, 1394, 1382, 1346, 1174, 1104, 973, 923, 862.

1-(Pent-4-en-2-yn-1-ylamino)-[1,1'-biphenyl]-4(1*H*)-one (4c)

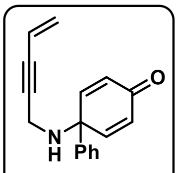

4c

yellow solid, 23% yield. mp 59 °C. **^{1}H NMR** (400 MHz, $CDCl_3$) δ (ppm) 7.54 (dt, *J* = 3.7, 2.1 Hz, 2H), 7.40 – 7.29 (m, 3H), 6.92 – 6.84 (m, 2H), 6.40 – 6.32 (m, 2H), 5.78 (ddt, *J* = 17.5, 11.0, 1.9 Hz, 1H), 5.61 (dd, *J* = 17.6, 2.1 Hz, 1H), 5.46 (dd, *J* = 11.0, 2.1 Hz, 1H), 3.54 (s, 2H), 1.91 (s, 1H). **^{13}C NMR** (100 MHz, $CDCl_3$) δ (ppm) 185.8, 152.4, 139.3, 129.2, 129.2, 128.6, 127.2, 126.4, 116.9, 88.5, 83.0, 61.3, 34.3. **HRMS (ESI)**: $[M+H]^{\oplus}$ calcd for $C_{17}H_{16}NO^{\oplus}$ 250.1226, found 250.1224. **IR (KBr)** *v* (cm^{-1}) 3303, 3059, 1665, 1626, 1599, 1487, 1447, 1392, 1271, 1166, 1075, 1029, 974, 922, 857, 754, 699.

Preparation of 1,3-enynes (4d-4f).

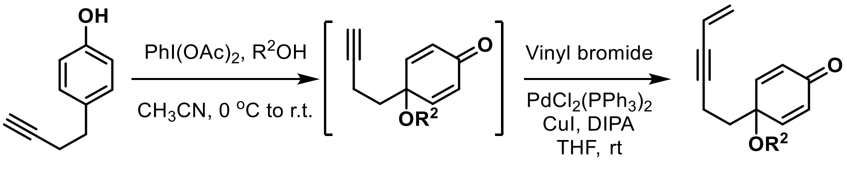

26 **27-28** **4d-4e**

A well-stirred solution of **26** (5.84 g, 40 mmol,) in alcohol (240 mmol, 6.0 equiv, methanol for **4d**, 2-bromoethan-1-ol for **4e**) and acetonitrile (5 ml) was cooled to 0 °C and treated with phenyliodine diacetate (PIDA, 19.32 g, 60 mmol, 1.5 equiv) in several portions. The resulting mixture was warmed to room temperature and stirred for 30 mins. The reaction mixture was diluted with water (300 mL) and extracted with ethyl acetate (100 mL × 3). The combined organic phases were washed with brine (200 mL), dried over anhydrous Na_2SO_4 and concentrated under reduced pressure. The residue was purified roughly by flash column chromatography using petroleum ether/ethyl acetate eluent to afford the crude product **27-28**.

A dried Schlenk flask was charged with bis(triphenylphosphine)palladium(II) dichloride (280 mg, 0.4 mmol, 0.01 equiv) and copper(I) iodide (150 mg, 0.8 mmol, 0.02 equiv), backfilled with argon for 3 times, then a mixture of crude product **27-28** in anhydrous THF (20 ml), diisopropylamine (11 ml, 80 mmol, 2.0 equiv) and vinyl bromide (1.0 M, 60 ml, 60 mmol, 1.5 equiv) was added. The reaction was monitored by TLC. Upon completion, the reaction mixture was filtered through Celite® and the filtrate was evaporated under reduced pressure and purified by flash column chromatography using petroleum ether/ethyl acetate eluent to afford the desired product **4d** and **4e**respectively.

4-(Hex-5-en-3-yn-1-yl)-4-methoxycyclohexa-2,5-dien-1-one (4d)

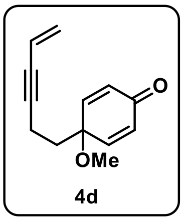

yellow oil, 36% yield. **^{1}H NMR** (400 MHz, $CDCl_3$) δ (ppm) 6.76 (d, *J* = 10.3 Hz, 2H), 6.38 (d, *J* = 10.3 Hz, 2H), 5.73 (ddt, *J* = 17.5, 11.0, 2.1 Hz, 1H), 5.52 (dd, *J* = 17.5, 2.2 Hz, 1H), 5.38 (dd, *J* = 11.0, 2.2 Hz, 1H), 3.20 (s, 3H), 2.35 (td, *J* = 7.9, 1.9 Hz, 2H), 1.98 (t, *J* = 7.8 Hz, 2H). **^{13}C NMR** (100 MHz, $CDCl_3$) δ (ppm) 185.3, 150.3, 131.9, 126.2, 117.4, 89.7, 80.4, 75.0, 53.2, 38.7, 14.2. **HRMS (ESI)**: $[M+H]^{\oplus}$ calcd for $C_{13}H_{15}O_2^{\oplus}$ 203.1067, found 203.1062. **IR (KBr)** *v* (cm^{-1}) 2934, 2826, 2227, 1672, 1633, 1607, 1392, 1259, 1164, 1097, 1076, 975, 920, 861, 801, 703.

4-(2-Bromoethoxy)-4-(hex-5-en-3-yn-1-yl)cyclohexa-2,5-dien-1-one (4e)

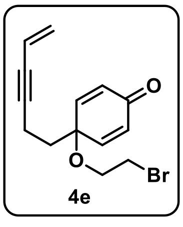

yellow oil, 25% yield. **^{1}H NMR** (400 MHz, $CDCl_3$) δ (ppm) 6.84 – 6.76 (m, 2H), 6.39 – 6.27 (m, 2H), 5.71 (ddt, *J* = 17.5, 11.0, 2.1 Hz, 1H), 5.51 (dd, *J* = 17.5, 2.2 Hz, 1H), 5.37 (dd, *J* = 11.0, 2.2 Hz, 1H), 3.60 (t, *J* = 5.9 Hz, 2H), 3.40 (t, *J* = 5.9 Hz, 2H), 2.38 (td, *J* = 7.9, 1.9 Hz, 2H), 2.00 (t, *J* = 7.8 Hz, 2H). **^{13}C NMR** (100 MHz, $CDCl_3$) δ (ppm) 185.0, 149.6, 131.6, 126.2, 117.3, 89.6, 80.5, 74.8, 65.4, 38.7, 31.0, 14.1. **HRMS (EI)**: $[M]^{\oplus}$ calcd for $C_{14}H_{15}O_2Br^{\oplus}$ 294.0255, found 294.0259. **IR (KBr)** *v* (cm^{-1}) 3293, 2930, 2859, 1671, 1630, 1426, 1392, 1382, 1278, 1096, 1073, 921, 863, 650, 571.

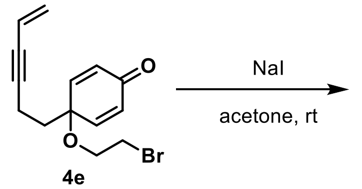

4-(Hex-5-en-3-yn-1-yl)-4-(2-iodoethoxy)cyclohexa-2,5-dien-1-one (4f) To a solution of **8ad** (0.59 g, 2 mmol) in acetone (10 ml) was added NaI (3.0 g, 20 mmol, 10.0 equiv), and the resulting mixture was stirred at room temperature. The reaction was monitored by crude ^{1}H-NMR analysis. Upon completion, the reaction was filtered through Celite® and the filtrate was evaporated under reduced pressure and purified by flash column chromatography (PE/EA = 50/1) to afford the desired product **4f** (0.58 g) as a yellow oil.

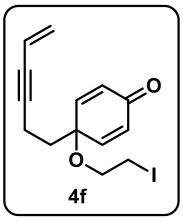

86% yield. **^{1}H NMR** (400 MHz, $CDCl_3$) δ (ppm) 6.87 – 6.76 (m, 2H), 6.41 – 6.28 (m, 2H), 5.71 (ddt, *J* = 17.5, 11.0, 2.1 Hz, 1H), 5.51 (dd, *J* = 17.5, 2.2 Hz, 1H), 5.38 (dd, *J* = 11.0, 2.2 Hz, 1H), 3.52 (t, *J* = 6.4 Hz, 2H), 3.19 (t, *J* = 6.4 Hz, 2H), 2.39 (td, *J* = 8.0, 1.9 Hz, 2H), 2.00 (t, *J* = 7.8 Hz, 2H). **^{13}C NMR** (100 MHz, $CDCl_3$) δ (ppm) 185.0, 149.7, 131.6, 126.3, 117.3, 89.6, 80.5, 74.8, 66.0, 38.7, 14.2, 3.6. **HRMS (EI)**: $[M]^{\oplus}$ calcd for $C_{14}H_{15}O_2I^{\oplus}$ 342.0117, found 342.0116. **IR (KBr)** *ν* (cm^{-1}) 2955, 2927, 2852, 1671, 1631, 1606, 1412, 1392, 1381, 1260, 1188, 1166, 1094, 1072, 974, 922, 862.

Preparation of 1,3-enynes (4g-4i).

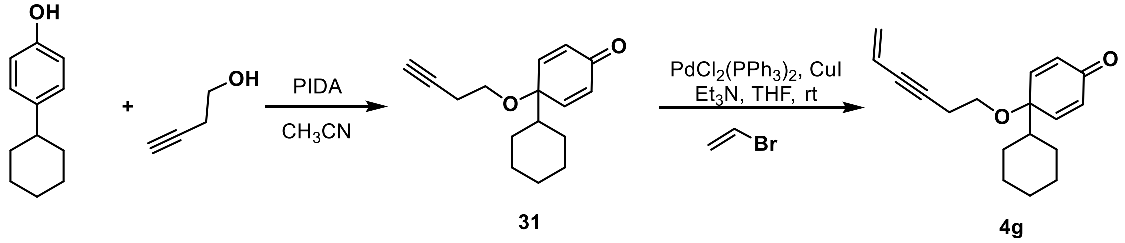

1-(Hex-5-en-3-yn-1-yloxy)-[1,1'-bi(cyclohexane)]-2,5-dien-4-one (4g)

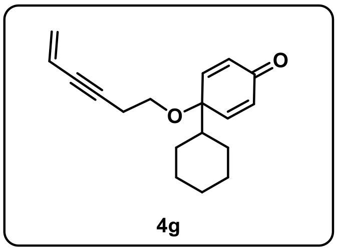

4g was prepared according to the general procedure in section 2.1. yellow oil, 7.5% yield. **^{1}H NMR** (400 MHz, $CDCl_3$) δ (ppm) 6.77 (d, *J* = 10.2 Hz, 2H), 6.36 (d, *J* = 10.2 Hz, 2H), 5.76 (dd, *J* = 17.5, 11.0 Hz, 1H), 5.56 (dd, *J* = 17.5, 1.7 Hz, 1H), 5.40 (dd, *J* = 11.0, 1.9 Hz, 1H), 3.42 (t, *J* = 6.9 Hz, 2H), 2.53 (dd, *J* = 6.8, 5.5 Hz, 2H), 1.89 (d, *J* = 12.2 Hz, 2H), 1.71 (dd, *J* = 36.6, 12.6 Hz, 4H), 1.27 – 1.04 (m, 3H), 0.92 (qd, *J* = 12.4, 2.7 Hz, 2H). **^{13}C NMR** (100 MHz, $CDCl_3$) δ (ppm) 185.7, 150.5, 131.7, 126.2, 117.3, 87.3, 80.4, 77.9, 63.4, 46.6, 27.3, 26.5, 21.4. **HRMS (EI)**: $[M]^{\oplus}$ calcd for $C_{18}H_{22}O_2^{\oplus}$

270.1614, found 270.1613. **IR (KBr)** *v* (cm^{-1}) 2974, 2928, 1766, 1727, 1600, 1452, 1416, 1372, 1316, 1161, 1072, 924.

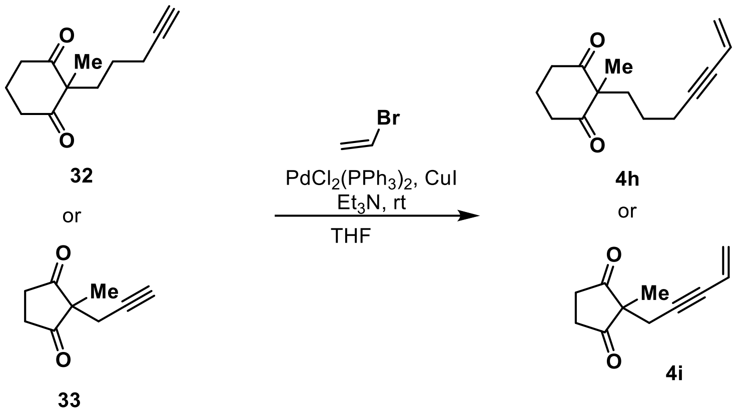

A dried flask was charged with bis(triphenylphosphine)palladium(II) dichloride (0.05 equiv) and copper(I) iodide (0.1 equiv), backfilled with argon for 3 times, then a mixture of crude product **32** or **33** in anhydrous THF, vinyl bromide (1.0 M, 1.2 equiv) and Et_3N (2.0 equiv) was added. The reaction was stirred at rt and monitored by TLC. Upon completion, the reaction was filtered through Celite® and the filtrate was evaporated under reduced pressure and purified by flash column chromatography using petroleum ether/ethyl acetate eluent to afford the desired product **4h** or **4i**.

2-(Hept-6-en-4-yn-1-yl)-2-methylcyclohexane-1,3-dione (4h)

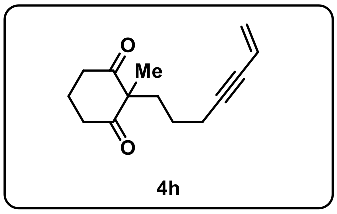

PE/EA = 5/1, yellow oil, 81% yield. **^{1}H NMR** (400 MHz, $CDCl_3$) δ (ppm) 5.76 (ddt, *J* = 17.5, 11.0, 2.1 Hz, 1H), 5.55 (dd, *J* = 17.5, 2.2 Hz, 1H), 5.39 (dd, *J* = 11.0, 2.2 Hz, 1H), 2.78 – 2.58 (m, 4H), 2.28 (td, *J* = 6.9, 2.0 Hz, 2H), 2.08 – 1.97 (m, 1H), 1.93 – 1.81 (m, 3H), 1.42 – 1.32 (m, 2H), 1.24 (s, 3H). **^{13}C NMR** (100 MHz, $CDCl_3$) δ (ppm) 210.1, 126.0, 117.4, 89.8, 80.0, 65.7, 37.9, 36.5, 23.9, 19.5, 18.9, 17.8. **HRMS (EI)**: $[M]^{\oplus}$ calcd for $C_{14}H_{18}O_2^{\oplus}$ 218.1301, found 218.1300. **IR (KBr)** *v* (cm^{-1}) 2959, 2938, 1726, 1695, 1459, 1428, 1375, 1335, 1273, 1129, 1027, 921.

2-Methyl-2-(pent-4-en-2-yn-1-yl)cyclopentane-1,3-dione (4i)

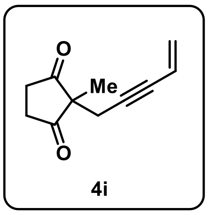

PE/EA = 5/1, yellow oil, 80% yield. **^{1}H NMR** (400 MHz, $CDCl_3$) δ (ppm) 5.68 (dd, *J* = 17.5, 11.0 Hz, 1H), 5.54 (d, *J* = 17.4 Hz, 1H), 5.42 (d, *J* = 10.9 Hz, 1H), 2.87 – 2.77 (m, 4H), 2.58 (s, 2H), 1.14 (s, 3H). **^{13}C NMR** (100 MHz, $CDCl_3$) δ (ppm) 215.5, 127.3, 116.5, 84.6, 81.5, 55.4, 35.9, 25.7, 19.0. **HRMS (EI)**: $[M]^{\oplus}$ calcd for $C_{11}H_{12}O_2^{\oplus}$ 176.0832, found 176.0837. **IR (KBr)** *v* (cm^{-1}) 3476, 2975, 2931, 1767, 1728, 1601, 1452, 1417, 1317, 1162, 1073, 926.

Substrate scope of 1,3-enynes

Condition A: for substrates (1a-1y, 4a-4c, and 4g-4i) A dried Schlenk flask was charged with CuCl (1.0 mg, 0.01 mmol, 5 mol%), (*R*,*R*)-Ph-BPE (6.1 mg, 0.012 mmol, 6 mol%), *t*-BuONa (1.5 mg, 0.015 mmol, 7.5 mol%), backfilled with argon. Then under -30ºC, anhydrous DCE (1.0 mL) was added and the solution was stirred for 10min under -30ºC. After that, PMHS (26.4 ul, 0.44 mmol, 2.2 equiv) was added dropwise and the solution was stirred for another 10min under -30ºC. Fianlly, a solution of substrate **1** or **4** (0.20 mmol, 1 equiv) and anhydrous *t*-BuOH (22 μL, 0.24 mmol, 1.2 equiv) in DCE (1.0 mL) was added. The resulting reaction mixture was stirred at -30ºC for 12h. The reaction mixture was filtered through a short column of silica gel. The diastereomeric ratio of the crude reaction mixture was determined by ^{1}H NMR spectroscopy. The residue was purified by flash silica gel (300-400 mesh) chromatography to afford the desired products **2** or **5**.

Condition B: for substrates (4d-4e) A dried Schlenk flask was charged with CuCl (1.0 mg, 0.01 mmol, 5 mol%), (*R*,*R*)-Ph-BPE (6.1 mg, 0.012 mmol, 6 mol%), *t*-BuONa (1.5 mg, 0.015 mmol, 7.5 mol%), backfilled with argon. Then under -30ºC, anhydrous DCE (1.0 mL) was added and the solution was stirred for 10min under -30ºC. After that, PMHS (26.4 ul, 0.44 mmol, 2.2 equiv) was added dropwise and the solution was stirred for another 10min under -30ºC. Fianlly, a solution of substrate **4** (0.20 mmol, 1 equiv) in DCE (1.0 mL) was added. The resulting reaction mixture was stirred at -30ºC for 12h. After that, NH_4F/MeOH (0.5 M, 0.8 ml) was added for work-up and the reaction mixture was filtered through a short column of silica gel. The diastereomeric ratio of the crude reaction mixture was determined by ^{1}H NMR spectroscopy. The residue was purified by flash silica gel (300-400 mesh) chromatography to afford the desired products **5**.

(Note: The racemic cyclization products were prepared according to the same procedure above using a mixture ligand of (*R,R*)-Ph-BPE and (*S,S*)-Ph-BPE**.)**

(3a*R*,7a*R*)-7a-Methyl-3-((*S*)-prop-1-en-1-ylidene)-2,3,3a,7a-tetrahydrobenzofuran-5(4*H*)-one (2a)

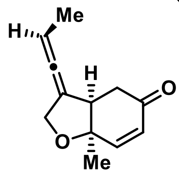
2a

colorless oil, 70% yield. R_f = 0.7 (hexane : acetone = 4 :1). $[\alpha]_D^{27.3}$ -38.7 (*c* 1.00, $CHCl_3$) for >99% *ee*. **^{1}H NMR** (400 MHz, $CDCl_3$) δ (ppm) 6.49 (dd, *J* = 10.2, 1.2 Hz, 1H), 5.94 (d, *J* = 10.3 Hz, 1H), 5.37 – 5.25 (m, 1H), 4.43 (dd, *J* = 12.0, 3.6 Hz, 1H), 4.22 (dd, *J* = 12.0, 2.1 Hz, 1H), 3.00 (s, 1H), 2.71 – 2.55 (m, 2H), 1.57 (d, *J* = 7.0 Hz, 3H), 1.51 (s, 3H). **^{13}C NMR** (100 MHz, $CDCl_3$) δ (ppm) 197.2, 195.4, 151.1, 129.7, 104.1, 93.1, 80.2, 67.2, 46.9, 37.1, 23.3, 14.8. **HRMS (EI)**: $[M]^{\oplus}$ calcd for $C_{12}H_{14}O_2^{\oplus}$ 190.0994, found 190.0995. **IR (KBr)** *ν* (cm^{-1}) 2973, 2927, 2900, 2859, 1686, 1412, 1373, 1284, 1264, 1231, 1156, 1113, 1081, 1039, 1022, 980, 907, 870, 779, 527. **HPLC**:

Chiracel AS-H Column (250 mm); detected at 214 nm; *n*-hexane/*i*-propanol = 95/5; flow = 0.8 ml/min; Retention time: 6.4 min (major), 9.9 min (minor).

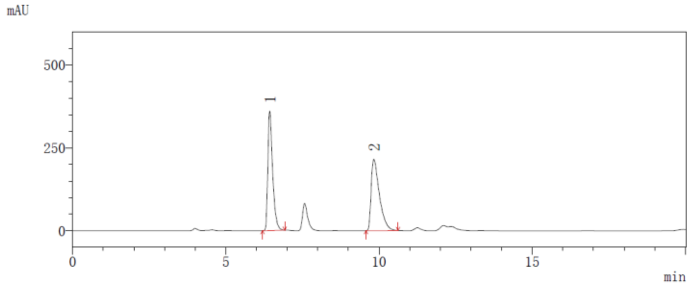

PDA Ch1 214nm

Number	Ret. Time	Area	Area%
1	6.436	3913638	49.42
2	9.833	4005027	50.58

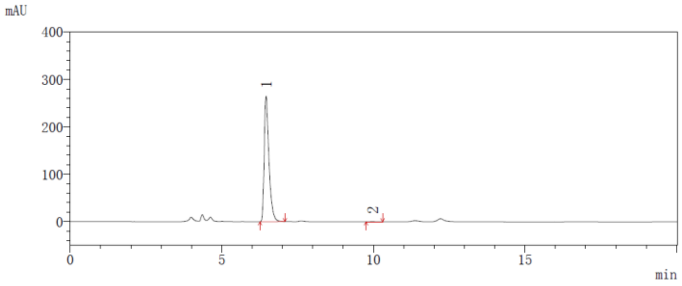

PDA Ch1 214nm

Number	Ret. Time	Area	Area%
1	6.461	2891321	99.86
2	9.970	4019	0.14

(3a*R*,7a*R*)-7a-Ethyl-3-((*S*)-prop-1-en-1-ylidene)-2,3,3a,7a-tetrahydrobenzofuran-5(4*H*)-one (2b)

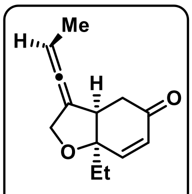

2b

colorless oil, 68% yield. R_f = 0.7 (hexane : acetone = 4 :1). $[\alpha]_D^{27.4}$ -26.9 (*c* 1.00, $CHCl_3$) for >99% *ee*. **^{1}H NMR** (400 MHz, $CDCl_3$) δ (ppm) 6.52 (dd, *J* = 10.3, 1.7 Hz, 1H), 6.00 (d, *J* = 10.3 Hz, 1H), 5.35 – 5.25 (m, 1H), 4.42 (dd, *J* = 12.0, 3.7 Hz, 1H), 4.23 (ddd, *J* = 12.0, 3.9, 1.8 Hz, 1H), 3.07 (s, 1H), 2.61 (qd, *J* = 17.0, 3.5 Hz, 2H), 1.96 – 1.73 (m, 2H), 1.58 (d, *J* = 7.0 Hz, 3H), 1.05 (t, *J* = 7.5 Hz, 3H). **^{13}C NMR** (100 MHz, $CDCl_3$) δ (ppm) 197.4, 195.4, 150.5, 130.4, 104.5, 93.1, 82.4, 67.0, 44.5, 37.4, 30.2, 14.8, 8.2. **HRMS (EI)**: $[M]^{\oplus}$ calcd for $C_{13}H_{16}O_2^{\oplus}$ 204.1150, found 204.1153. **IR (KBr)** *v* (cm^{-1}) 2969, 2922, 2858, 1687, 1462, 1412, 1380, 1247, 1153, 1078, 1055, 1031, 968, 940, 917, 896, 779. **HPLC**: Chiracel ID-H Column (250 mm); detected at 214 nm; *n*-hexane/*i*-propanol = 95/5; flow = 1.0 ml/min; Retention time: 5.5 min (major), 5.8 min (minor).

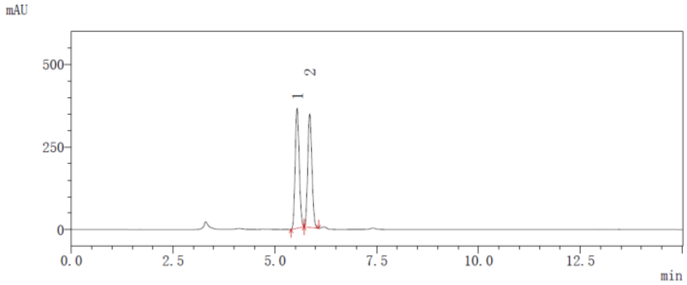

PDA Ch1 214nm

Number	Ret. Time	Area	Area%
1	5.546	2518932	50.66
2	5.854	2453456	49.34

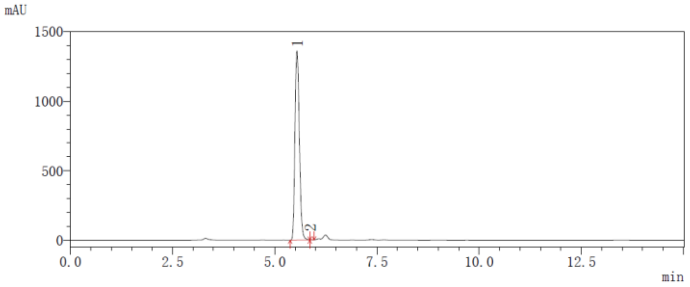

PDA Ch1 214nm

Number	Ret. Time	Area	Area%
1	5.544	10231807	99.90
2	5.861	9799	0.10

(3a*R*,7a*R*)-7a-Isopropyl-3-((*S*)-prop-1-en-1-ylidene)-2,3,3a,7a-tetrahydrobenzofuran-5(4*H*)-one (2c)

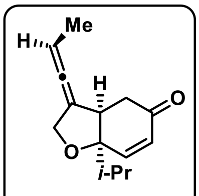

2c

colorless oil, 87% yield. R_f = 0.8 (hexane : acetone = 4 :1). $[\alpha]_D^{27.5}$ -24.3 (*c* 1.00, $CHCl_3$) for >99% *ee*. **^{1}H NMR** (400 MHz, $CDCl_3$) δ (ppm) 6.53 (dd, *J* = 10.4, 1.8 Hz, 1H), 6.07 (d, *J* = 10.4 Hz, 1H), 5.38 – 5.24 (m, 1H), 4.40 (dd, *J* = 12.0, 3.5 Hz, 1H), 4.22 (ddd, *J* = 11.9, 4.0, 1.8 Hz, 1H), 3.15 (s, 1H), 2.63 (d, *J* = 3.9 Hz, 2H), 2.07 (dt, *J* = 13.9, 6.9 Hz, 1H), 1.58 (d, *J* = 7.0 Hz, 3H), 1.06 (dd, *J* = 6.9, 3.7 Hz, 6H). **^{13}C NMR** (100 MHz, $CDCl_3$) δ (ppm) 197.7, 195.4,

149.6, 131.2, 105.4, 93.2, 84.5, 66.8, 42.4, 38.4, 35.5, 18.0, 17.2, 14.8. **HRMS (EI)**: [M]$^{\oplus}$ calcd for $C_{14}H_{18}O_2^{\oplus}$ 218.1307, found 218.1313. **IR (KBr)** *v* (cm^{-1}) 2962, 2922, 2877, 1686, 1463, 1411, 1384, 1368, 1247, 1224, 1174, 1135, 1050, 1029, 948, 915, 896, 775, 701. **HPLC**: Chiracel AS-H Column (250 mm); detected at 214 nm; *n*-hexane/*i*-propanol = 95/5; flow = 0.8 ml/min; Retention time: 6.3 min (major), 7.2 min (minor).

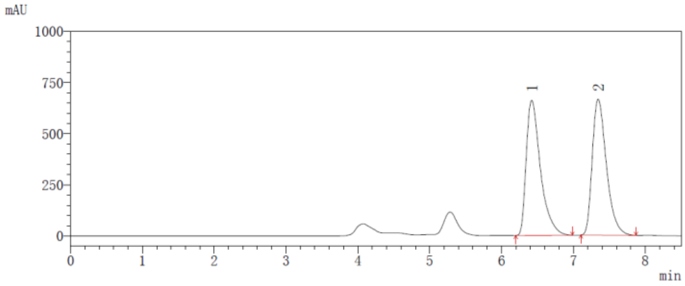

PDA Ch1 214nm

Number	Ret.Time	Area	Area%
1	6.420	9078669	49.92
2	7.345	9108008	50.08

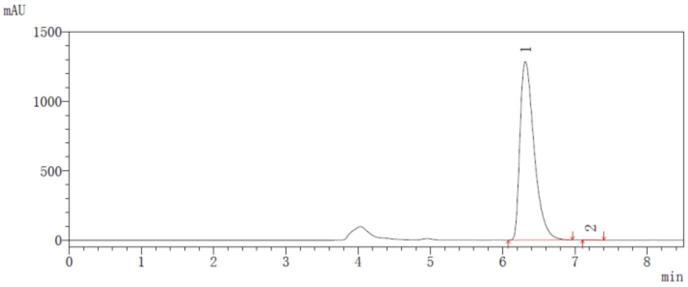

PDA Ch1 214nm

Number	Ret.Time	Area	Area%
1	6.314	17481382	99.93
2	7.212	11685	0.07

(3a*R*,7a*R*)-7a-(*tert*-Butyl)-3-((*S*)-prop-1-en-1-ylidene)-2,3,3a,7a-tetrahydrobenzofuran-5(4*H*)-one (2d)

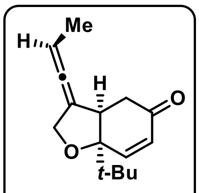

2d

colorless oil, 87% yield. R_f = 0.7 (hexane : acetone = 5 :1). $[\alpha]_D^{27.6}$ -42.0 (*c* 1.00, $CHCl_3$) for >99% *ee*. **^{1}H NMR** (400 MHz, $CDCl_3$) δ (ppm) 6.65 (dd, *J* = 10.6, 1.8 Hz, 1H), 6.09 (d, *J* = 10.5 Hz, 1H), 5.34 – 5.22 (m, 1H), 4.43 – 4.33 (m, 1H), 4.25 – 4.16 (m, 1H), 3.32 (s, 1H), 2.65 (d, *J* = 4.7 Hz, 2H), 1.58 (d, *J* = 7.0 Hz, 3H), 1.07 (s, 9H). **^{13}C NMR** (100 MHz, $CDCl_3$) δ (ppm) 197.5, 195.4, 149.3, 131.3, 105.9, 93.2, 86.0, 66.7, 40.8, 38.9, 37.6, 25.8, 14.8. **HRMS (DART)**: [M+H]$^{\oplus}$ calcd for $C_{15}H_{21}O_2^{\oplus}$ 233.1536, found 233.1536. **IR (KBr)** *v* (cm^{-1}) 2958, 2874, 1688, 1482, 1466, 1412, 1387, 1365, 1344, 1258, 1234, 1177, 1140, 1096, 1053, 956, 918, 776, 695, 543. **HPLC**: Chiracel ID-H Column (250 mm); detected at 214 nm, at 32°C; *n*-hexane/*i*-propanol = 95/5; flow = 0.7 ml/min; Retention time: 6.1 min (major).

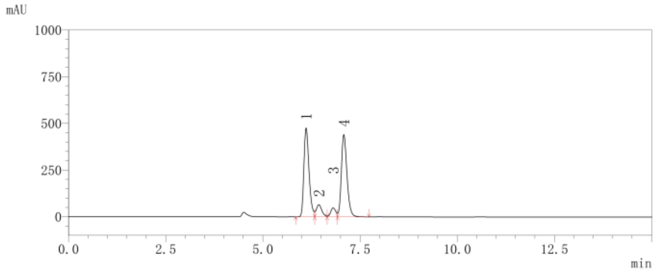

PDA Ch1 214nm

Number	Ret.Time	Area	Area%
1	6.113	4377286	44.34
2	6.442	645197	6.54
3	6.810	444647	4.50
4	7.084	4404845	44.62

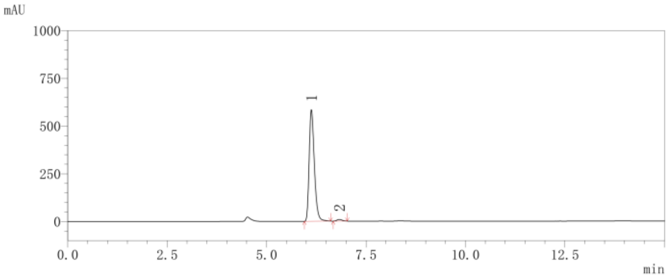

PDA Ch1 214nm

Number	Ret.Time	Area	Area%
1	6.127	5319243	98.49
2	6.829	81806	1.51

(3a*R*,7a*R*)-7a-Cyclohexyl-3-((*S*)-prop-1-en-1-ylidene)-2,3,3a,7a-tetrahydrobenzofuran-5(4*H*)-one (2e)

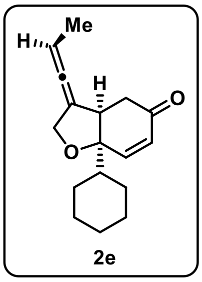

colorless oil, 99% yield. R_f = 0.8 (hexane : acetone = 4 :1). $[\alpha]_D^{27.7}$ -29.7 (*c* 2.00, $CHCl_3$) for >99% *ee*. **^{1}H NMR** (400 MHz, $CDCl_3$) δ (ppm) 6.51 (dd, *J* = 10.4, 1.8 Hz, 1H), 6.04 (d, *J* = 10.4 Hz, 1H), 5.37 – 5.18 (m, 1H), 4.38 (dd, *J* = 11.9, 3.7 Hz, 1H), 4.20 (ddd, *J* = 11.9, 4.0, 1.8 Hz, 1H), 3.17 (s, 1H), 2.63 (d, *J* = 3.8 Hz, 2H), 1.91 (dd, *J* = 25.8, 12.2 Hz, 2H), 1.78 (dd, *J* = 11.4, 8.2 Hz, 2H), 1.75 – 1.65 (m, 2H), 1.57 (d, *J* = 7.0 Hz, 3H), 1.29 – 1.09 (m, 5H). **^{13}C NMR** (100 MHz, $CDCl_3$) δ (ppm) 197.8, 195.4, 150.1, 130.9, 105.4, 93.2, 84.2, 66.7, 45.8, 42.4, 38.4, 28.1, 27.3, 26.7, 26.5, 14.8. **HRMS (EI)**: $[M]^{\oplus}$ calcd for $C_{17}H_{22}O_2^{\oplus}$ 258.1620, found 258.1622. **IR (KBr)** *v* (cm^{-1}) 2926, 2854, 1688, 1450, 1415, 1385, 1371, 1256, 1238, 1151, 1080, 1045, 962, 914, 900, 888, 822, 777. **HPLC**: Phenomenex Lux 5u Cellulose-2 (PC-2) (250 mm); detected at 214 nm, at 32°C; *n*-hexane/*i*-propanol = 98/2; flow = 0.7 ml/min; Retention time: 11.9 min (major), 10.6 min (minor).

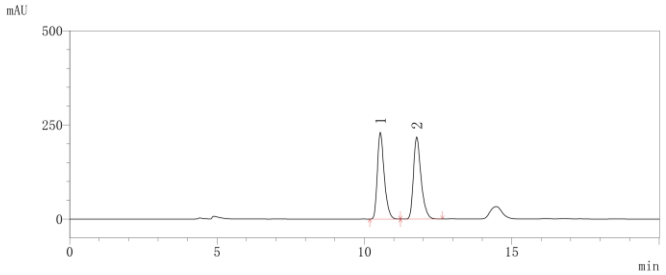

PDA Ch1 214nm

Number	Ret.Time	Area	Area%
1	10.540	3736305	48.89
2	11.774	3906557	51.11

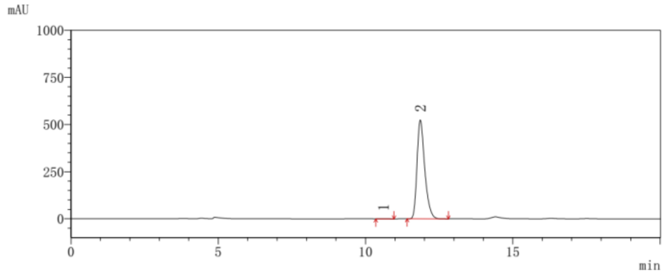

PDA Ch1 214nm

Number	Ret.Time	Area	Area%
1	10.609	7905	0.08
2	11.860	9572001	99.92

(3a*R*,7a*R*)-7a-((3*R*,5*R*,7*R*)-Adamantan-1-yl)-3-((*S*)-prop-1-en-1-ylidene)-2,3,3a,7a-tetrahydrobenzofuran-5(4*H*)-one (2f)

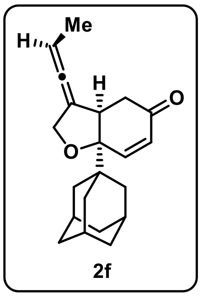

colorless oil, 98% yield. R_f = 0.8 (hexane : acetone = 4 :1). $[\alpha]_D^{24.9}$ -63.8 (*c* 2.00, $CHCl_3$) for >99% *ee*. **^{1}H NMR** (400 MHz, $CDCl_3$) δ (ppm) 6.67 (dd, *J* = 10.6, 1.6 Hz, 1H), 6.09 (d, *J* = 10.6 Hz, 1H), 5.34 – 5.23 (m, 1H), 4.35 (dd, *J* = 11.8, 3.7 Hz, 1H), 4.24 – 4.14 (m, 1H), 3.37 (s, 1H), 2.73 – 2.57 (m, 2H), 2.01 (s, 3H), 1.84 – 1.60 (m, 12H), 1.57 (d, *J* = 7.0 Hz, 3H). **^{13}C NMR** (100 MHz, $CDCl_3$) δ (ppm) 197.7, 195.4, 149.2, 131.2, 105.8, 93.2, 86.0, 66.6, 39.8, 39.4, 38.9, 37.0, 37.0, 28.5, 14.8. **HRMS (EI)**: $[M]^{\oplus}$ calcd for $C_{27}H_{26}O_2^{\oplus}$ 310.1933, found 310.1931. **IR (KBr)** *v* (cm^{-1}) 2906, 2850, 1687, 1451, 1414, 1386, 1361, 1343, 1245, 1222, 1151, 1082, 1048, 1020, 995, 976, 915, 776. **HPLC**: Chiracel AS-H Column (250 mm); detected at 214 nm; *n*-hexane/*i*-propanol = 95/5; flow = 0.8 ml/min; Retention time: 8.9 min (major), 7.9 min (minor).

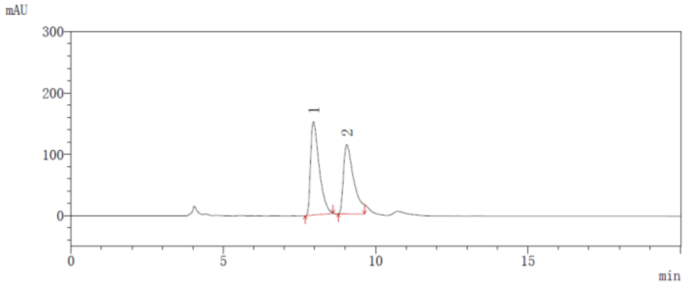

PDA Ch1 214nm

Number	Ret. Time	Area	Area%
1	7.962	2989404	53.12
2	9.055	2638432	46.88

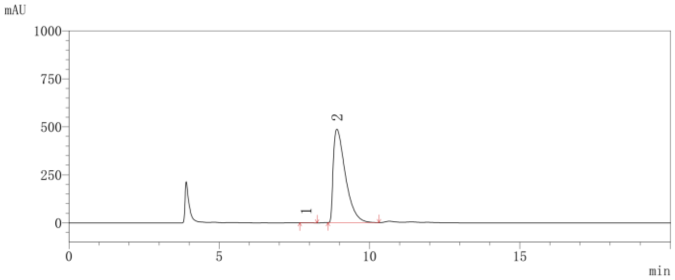

PDA Ch1 214nm

Number	Ret. Time	Area	Area%
1	7.884	8494	0.06
2	8.913	13587911	99.94

(3a*R*,7a*R*)-3-((*S*)-Prop-1-en-1-ylidene)-7a-vinyl-2,3,3a,7a-tetrahydrobenzofuran-5(4*H*)-one (2g)

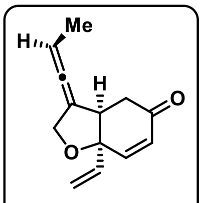

2g

colorless oil, 74% yield. R_f = 0.6 (hexane : acetone = 4 :1). $[\alpha]_D^{25.0}$ -0.6 (*c* 1.00, $CHCl_3$) for >99% *ee*. **^{1}H NMR** (400 MHz, $CDCl_3$) δ (ppm) 6.46 (dd, *J* = 10.3, 1.9 Hz, 1H), 6.09 (d, *J* = 10.3 Hz, 1H), 6.00 (dd, *J* = 17.9, 10.4 Hz, 1H), 5.38 – 5.26 (m, 3H), 4.50 (dd, *J* = 12.0, 4.0 Hz, 1H), 4.28 (ddd, *J* = 12.0, 4.0, 1.9 Hz, 1H), 3.09 (s, 1H), 2.60 (qd, *J* = 16.9, 3.6 Hz, 2H), 1.58 (d, *J* = 7.0 Hz, 3H). **^{13}C NMR** (100 MHz, $CDCl_3$) δ (ppm) 197.2, 195.3, 148.0, 137.3, 131.1, 117.4, 103.5, 93.3, 82.8, 67.3, 46.2, 36.2, 14.7. **HRMS (EI)**: $[M]^{\oplus}$ calcd for $C_{13}H_{14}O_2^{\oplus}$ 202.0994, found 202.0989. **IR (KBr)** *v* (cm^{-1}) 3368, 2925, 1726, 1687, 1600, 1412, 1383, 1255, 1163, 1092, 1052, 990, 936, 899, 797. **HPLC**: Chiracel AS-H Column (250 mm); detected at 214 nm; *n*-hexane/*i*-propanol = 95/5; flow = 0.8 ml/min; Retention time: 6.5 min (major), 7.6 min (minor).

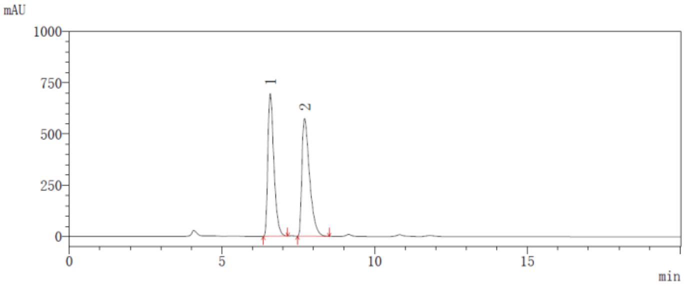

PDA Ch1 214nm

Number	Ret. Time	Area	Area%
1	6.584	9586599	48.85
2	7.707	10036691	51.15

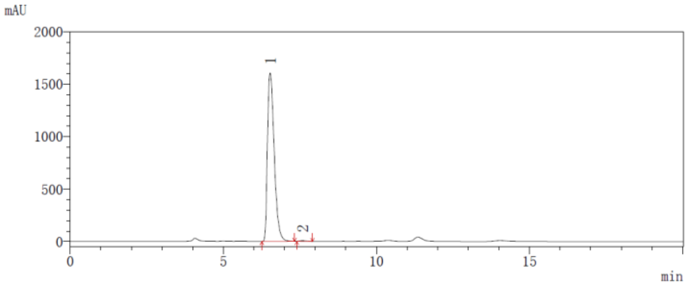

PDA Ch1 214nm

Number	Ret. Time	Area	Area%
1	6.531	24555183	99.63
2	7.591	91991	0.37

(3a*R*,7a*R*)-7a-Benzyl-3-((*S*)-prop-1-en-1-ylidene)-2,3,3a,7a-tetrahydrobenzofuran-5(4*H*)-one (2h)

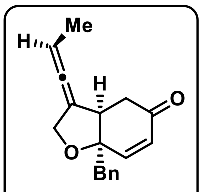

2h

colorless oil, 71% yield. R_f = 0.8 (hexane : acetone = 4 :1). $[\alpha]_D^{27.9}$ -44.9 (*c* 1.00, $CHCl_3$) for >99% *ee*. **^{1}H NMR** (400 MHz, $CDCl_3$) δ (ppm) 7.33 – 7.22 (m, 5H), 6.46 (dd, *J* = 10.3, 1.9 Hz, 1H), 5.98 (d, *J* = 10.3 Hz, 1H), 5.28 (qd, *J* = 6.9, 2.9 Hz, 1H), 4.42 (dd, *J* = 12.0, 3.3 Hz, 1H), 4.22 (ddd, *J* = 12.0, 4.0, 1.9 Hz, 1H), 3.17 – 3.04 (m, 3H), 2.54 (dd, *J* = 16.9, 1.3 Hz, 1H), 2.27 (dd, *J* = 16.9, 5.1 Hz, 1H), 1.56 (d, *J* = 7.0 Hz, 3H). **^{13}C NMR** (100 MHz, $CDCl_3$) δ (ppm) 197.3, 195.4, 149.9, 135.5, 130.5, 130.4, 128.4, 127.2, 104.1, 93.2, 82.2, 67.0, 44.6, 43.7, 37.1, 14.7. **HRMS (EI)**: $[M]^{\oplus}$ calcd for $C_{18}H_{18}O_2^{\oplus}$ 266.1307, found 266.1317. **IR (KBr)** *v* (cm^{-1}) 3029,

2919, 2857, 1686, 1495, 1455, 1412, 1383, 1249, 1232, 1145, 1110, 1080, 1037, 1012, 969, 919, 761, 712, 525. **HPLC**: Chiracel AS-H Column (250 mm); detected at 214 nm; *n*-hexane/*i*-propanol = 95/5; flow = 0.8 ml/min; Retention time: 6.6 min (minor), 7.5 min (major).

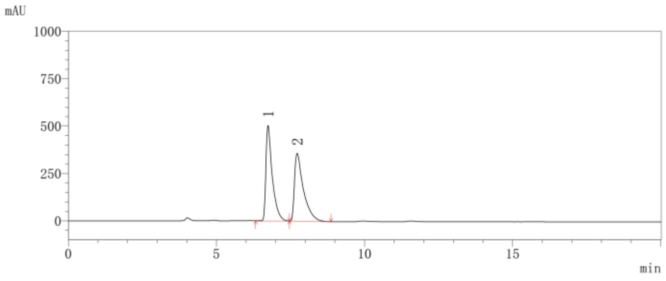

PDA Ch1 214nm

Number	Ret.Time	Area	Area%
1	6.741	7567468	50.14
2	7.725	7524564	49.86

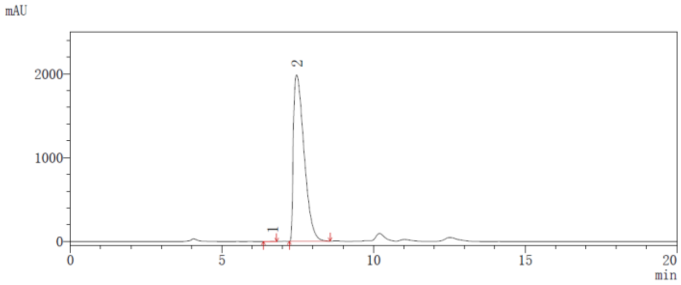

PDA Ch1 214nm

Number	Ret.Time	Area	Area%
1	6.668	6165	0.01
2	7.465	48281001	99.99

(3a*R*,7a*R*)-7a-Phenyl-3-((*S*)-prop-1-en-1-ylidene)-2,3,3a,7a-tetrahydrobenzofuran-5(4*H*)-one (2i)

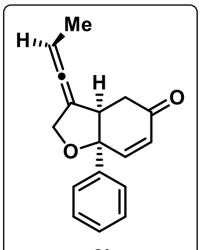

2i

colorless oil, 89% yield. R_f = 0.7 (hexane : acetone = 4 :1). $[\alpha]_D^{28.0}$ -22.0 (*c* 1.00, $CHCl_3$) for >99% *ee*. **^{1}H NMR** (400 MHz, $CDCl_3$) δ (ppm) 7.51 (d, *J* = 7.2 Hz, 2H), 7.40 (t, *J* = 7.3 Hz, 2H), 7.38 – 7.32 (m, 1H), 6.62 (dd, *J* = 10.3, 1.9 Hz, 1H), 6.23 (d, *J* = 10.3 Hz, 1H), 5.40 – 5.30 (m, 1H), 4.71 (dd, *J* = 11.9, 3.5 Hz, 1H), 4.44 (ddd, *J* = 12.0, 3.9, 1.9 Hz, 1H), 3.18 (s, 1H), 2.64 (d, *J* = 3.8 Hz, 2H), 1.62 (d, *J* = 7.0 Hz, 3H). **^{13}C NMR** (100 MHz, $CDCl_3$) δ (ppm) 197.4, 195.2, 148.5, 140.0, 131.1, 128.9, 128.4, 125.5, 104.2, 93.3, 83.8, 67.5, 49.1, 36.5, 14.7. **HRMS (EI)**: $[M]^{\oplus}$ calcd for $C_{17}H_{16}O_2^{\oplus}$ 252.1150, found 252.1144. **IR (KBr)** *ν* (cm^{-1}) 3060, 3031, 2951, 2923, 2858, 1689, 1491, 1448, 1411, 1379, 1329, 1248, 1195, 1133, 1077, 1045, 1012, 974, 955, 918, 776, 754, 670. **HPLC**: Phenomenex Lux 5u Cellulose-2 (PC-2) Column (250 mm); detected at 214 nm; *n*-hexane/*i*-propanol = 95/5; flow = 0.8 ml/min; Retention time: 7.7 min (minor), 8.5 min (major).

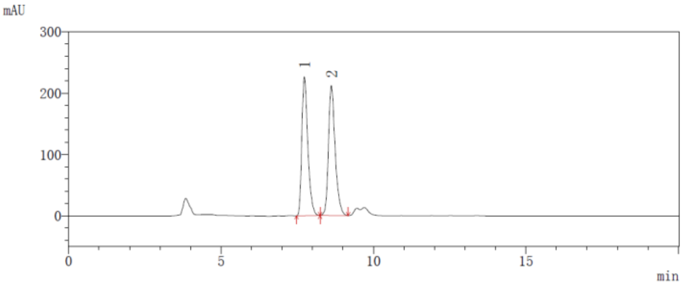

PDA Ch1 214nm

Number	Ret.Time	Area	Area%
1	7.734	3086216	48.83
2	8.619	3234529	51.17

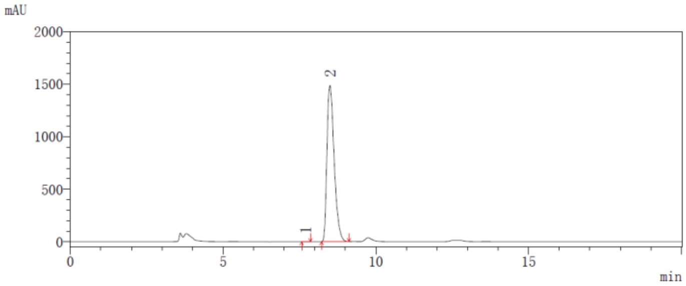

PDA Ch1 214nm

Number	Ret.Time	Area	Area%
1	7.696	3675	0.02
2	8.498	24316970	99.98

(3a*R*,7a*R*)-7a-(4-Bromophenyl)-3-((*S*)-prop-1-en-1-ylidene)-2,3,3a,7a-tetrahydrobenzofuran-5(4*H*)-one (2j)

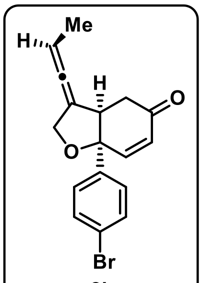

2j

colorless oil, 83% yield. R_f = 0.6 (hexane : acetone = 4 :1). $[\alpha]_D^{27.8}$ -5.9 (*c* 2.00, $CHCl_3$) for 98% *ee*. **^{1}H NMR** (400 MHz, $CDCl_3$) δ (ppm) 7.56 – 7.50 (m, 2H), 7.40 – 7.34 (m, 2H), 6.58 (dd, *J* = 10.3, 2.0 Hz, 1H), 6.23 (dd, *J* = 10.3, 0.7 Hz, 1H), 5.41 – 5.27 (m, 1H), 4.70 (ddd, *J* = 12.0, 4.0, 1.1 Hz, 1H), 4.43 (ddd, *J* = 12.0, 4.0, 1.9 Hz, 1H), 3.12 (s, 1H), 2.72 – 2.50 (m, 2H), 1.61 (d, *J* = 7.0 Hz, 3H). **^{13}C NMR** (100 MHz, $CDCl_3$) δ (ppm) 197.0, 195.2, 147.8, 139.1, 132.0, 131.4, 127.3, 122.6, 103.9, 93.6, 83.4, 67.5, 49.1, 36.3, 14.7. **HRMS (EI)**: $[M]^{\oplus}$ calcd for $C_{17}H_{15}O_2Br^{\oplus}$ 330.0255, found 330.0251. **IR (KBr)** *ν* (cm^{-1}) 2952, 2923, 2858, 1690, 1486, 1412, 1396, 1379, 1248, 1178, 1133, 1074, 1048, 1008, 976, 955, 919, 899, 824, 777, 722, 682, 533, 508. **HPLC**: Chiracel ID-H Column (250 mm); detected at 214 nm; *n*-hexane/*i*-propanol = 94/6; flow = 1.0 ml/min; Retention time: 6.2 min (major), 9.1 min (minor).

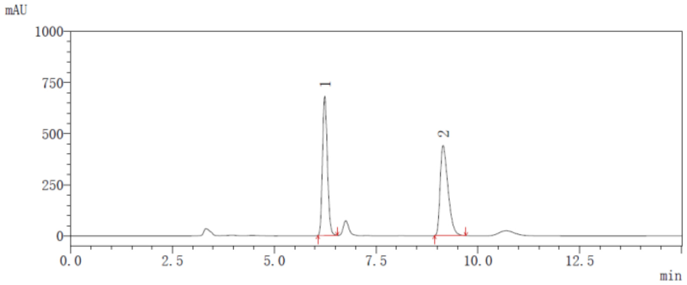

PDA Ch1 214nm

Number	Ret.Time	Area	Area%
1	6.240	5680867	49.12
2	9.148	5884802	50.88

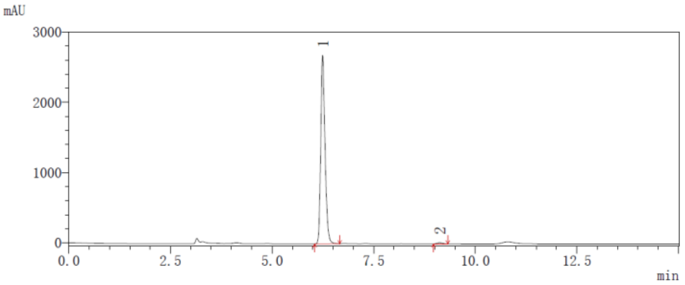

PDA Ch1 214nm

Number	Ret.Time	Area	Area%
1	6.244	19663401	99.12
2	9.126	174452	0.88

(3a*R*,7a*R*)-7a-(4-Nitrophenyl)-3-((*S*)-prop-1-en-1-ylidene)-2,3,3a,7a-tetrahydrobenzofuran-5(4*H*)-one (2k)

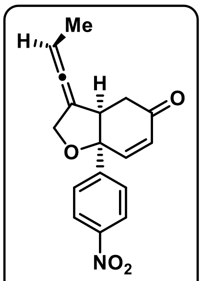

2k

white solid, 76% yield. R_f = 0.4 (hexane : acetone = 3 :1). $[\alpha]_D^{25.0}$ -5.7 (*c* 2.00, $CHCl_3$) for >99% *ee*. mp 150 °C. **^{1}H NMR** (400 MHz, $CDCl_3$) δ (ppm) 8.25 (d, *J* = 8.9 Hz, 2H), 7.70 (d, *J* = 8.9 Hz, 2H), 6.64 – 6.52 (m, 1H), 6.29 (d, *J* = 10.3 Hz, 1H), 5.45 – 5.31 (m, 1H), 4.74 (dd, *J* = 12.0, 3.9 Hz, 1H), 4.47 (ddd, *J* = 12.0, 3.9, 1.9 Hz, 1H), 3.14 (s, 1H), 2.65 (ddd, *J* = 21.9, 17.1, 3.4 Hz, 2H), 1.62 (d, *J* = 7.1 Hz, 3H). **^{13}C NMR** (100 MHz, $CDCl_3$) δ (ppm) 196.4, 195.3, 148.0, 147.4, 146.8, 131.8, 126.6, 124.1, 103.6, 94.0, 83.3, 67.7, 49.1, 36.2, 14.7. **HRMS (EI)**: $[M]^{\oplus}$ calcd for $C_{17}H_{15}NO_2^{\oplus}$ 297.1001, found 297.0993. **IR (KBr)** *ν* (cm^{-1}) 2924, 2864, 1690, 1598, 1522, 1491, 1410, 1349, 1249, 1134, 1049, 1011, 976, 920, 899, 853, 779, 750, 721, 698, 684. **HPLC**: Chiracel AD-H Column (250 mm); detected at 214 nm; *n*-hexane/*i*-propanol = 95/5; flow = 0.8 ml/min; Retention time: 17.3 min (major), 24.2 min (minor).

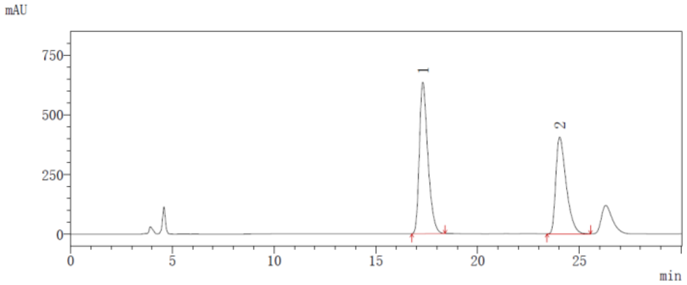

PDA Ch1 214nm

Number	Ret. Time	Area	Area%
1	17.308	18389226	56.10
2	24.033	14388034	43.90

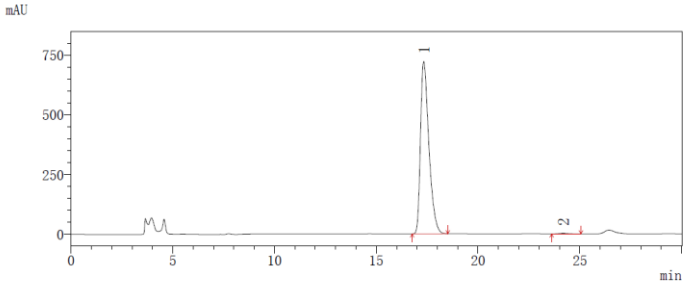

PDA Ch1 214nm

Number	Ret. Time	Area	Area%
1	17.333	20653380	99.63
2	24.177	76885	0.37

4-((3a*R*,7a*R*)-5-Oxo-3-((*S*)-prop-1-en-1-ylidene)-3,3a,4,5-tetrahydrobenzofuran-7a(2*H*)-yl)benzonitrile (2l)

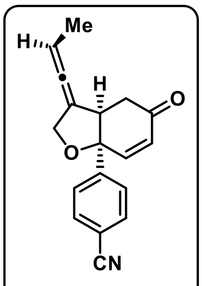

2l

white solid, 79% yield. R_f = 0.5 (hexane : acetone = 3 :1). $[\alpha]_D^{25.0}$ -9.3 (*c* 1.00, $CHCl_3$) for >99% *ee*. mp 180 °C. **^{1}H NMR** (400 MHz, $CDCl_3$) δ (ppm) 7.70 (d, *J* = 8.4 Hz, 2H), 7.63 (d, *J* = 8.4 Hz, 2H), 6.56 (dd, *J* = 10.3, 2.0 Hz, 1H), 6.27 (d, *J* = 10.3 Hz, 1H), 5.45 – 5.31 (m, 1H), 4.72 (dd, *J* = 12.0, 3.8 Hz, 1H), 4.45 (ddd, *J* = 12.0, 3.9, 1.8 Hz, 1H), 3.12 (s, 1H), 2.63 (ddd, *J* = 21.9, 17.1, 3.5 Hz, 2H), 1.61 (d, *J* = 7.1 Hz, 3H). **^{13}C NMR** (100 MHz, $CDCl_3$) δ (ppm) 196.5, 195.3, 147.0, 145.4, 132.7, 131.8, 126.4, 118.6, 112.4, 103.6, 93.9, 83.3, 67.6, 49.1, 36.2, 14.7. **HRMS (EI)**: $[M]^{\oplus}$ calcd for $C_{18}H_{15}NO_2^{\oplus}$ 277.1103, found 277.1099. **IR (KBr)** *ν* (cm^{-1}) 2956, 2927, 2874, 2230, 1689, 1607, 1503, 1410, 1379, 1252, 1230, 1192, 1077, 1043, 1022, 1007, 996, 977, 950, 844, 790, 764, 737, 693, 567, 528, 404. **HPLC**: Chiracel ID-H Column (250 mm); detected at 214 nm; *n*-hexane/*i*-propanol = 95/5; flow = 1.0 ml/min; Retention time: 16.3 min (major), 24.9 min (minor).

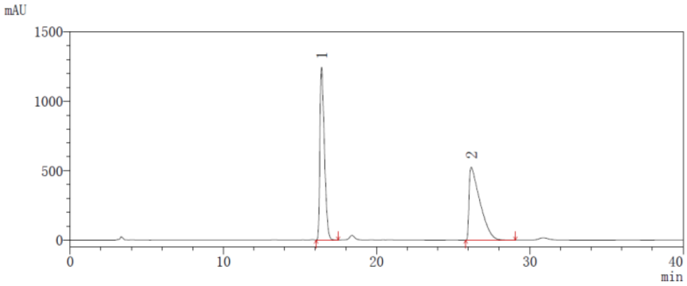

PDA Ch1 214nm

Number	Ret. Time	Area	Area%
1	16.409	25362331	49.48
2	26.180	25895860	50.52

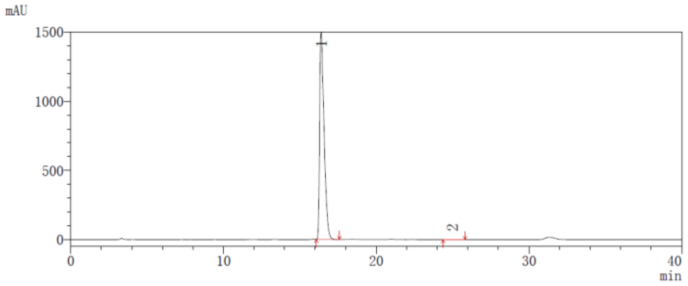

PDA Ch1 214nm

Number	Ret. Time	Area	Area%
1	16.393	30355095	99.94
2	24.970	17708	0.06

(3a*R*,7a*R*)-3-((*S*)-Prop-1-en-1-ylidene)-7a-(pyridin-2-yl)-2,3,3a,7a-tetrahydrobenzofuran-5(4*H*)-one (2m)

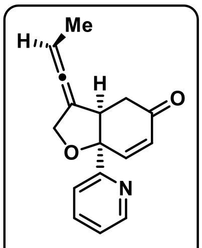

2m

red oil, 71% yield. R_f = 0.5 (hexane : acetone = 4 :1). $[\alpha]_D^{25.0}$ 67.4 (*c* 1.00, $CHCl_3$) for 98% *ee*. **^{1}H NMR** (400 MHz, $CDCl_3$) δ (ppm) 8.55 (d, *J* = 4.3 Hz, 1H), 7.74 (td, *J* = 7.8, 1.7 Hz, 1H), 7.65 (d, *J* = 7.9 Hz, 1H), 7.29 – 7.15 (m, 1H), 6.59 (dd, *J* = 10.2, 1.9 Hz, 1H), 6.16 (d, *J* = 10.2 Hz, 1H), 5.34 (dt, *J* = 9.1, 4.0 Hz, 1H), 4.66 (dd, *J* = 11.9, 3.3 Hz, 1H), 4.45 (ddd, *J* = 11.9, 4.0, 1.9 Hz, 1H), 3.37 (s, 1H), 3.11 (dd, *J* = 16.5, 5.1 Hz, 1H), 2.66 (dd, *J* = 16.5, 1.7 Hz, 1H), 1.61 (d, *J* = 7.0 Hz, 3H). **^{13}C NMR** (100 MHz, $CDCl_3$) δ (ppm) 197.9, 195.3, 160.4, 149.2, 148.0, 137.2, 130.9, 123.2, 120.2, 104.1, 93.4, 84.1, 67.5, 47.4, 37.4, 14.7. **HRMS (DART)**: $[M+H]^{\oplus}$ calcd for $C_{16}H_{16}NO_2^{\oplus}$ 254.1187, found 254.1175. **IR (KBr)** *ν* (cm^{-1}) 2923, 2856, 1687, 1588, 1571, 1468, 1435, 1410, 1381, 1248, 1101, 1053, 1014, 997, 776, 749. **HPLC**: Phenomenex Lux 5u Cellulose-2 (PC-2) Column (250 mm); detected at 214 nm; *n*-hexane/*i*-propanol = 95/5; flow = 0.8 ml/min; Retention time: 11.6 min (major), 16.1 min (minor).

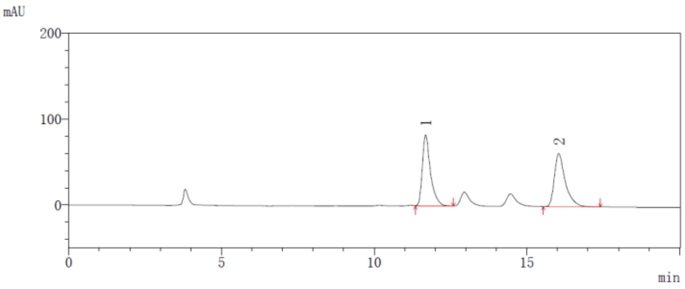

PDA Ch1 214nm

Number	Ret. Time	Area	Area%
1	11.686	1562138	50.25
2	16.043	1546660	49.75

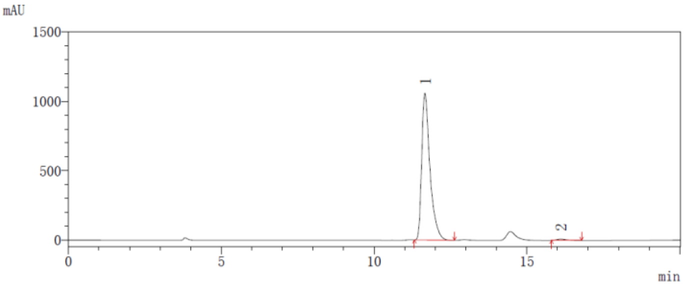

PDA Ch1 214nm

Number	Ret. Time	Area	Area%
1	11.664	19909041	98.83
2	16.107	235191	1.17

(3a*R*,7a*R*)-7a-(4-Oxocyclohexyl)-3-((*S*)-prop-1-en-1-ylidene)-2,3,3a,7a-tetrahydrobenzofuran-5(4*H*)-one (2n)

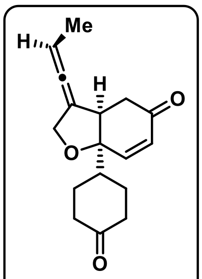

2n

colorless oil, 61% yield. R_f = 0.4 (hexane : acetone = 4 :1). $[\alpha]_D^{25.0}$ -25.9 (*c* 1.00, $CHCl_3$) for 98% *ee*. **^{1}H NMR** (400 MHz, $CDCl_3$) δ (ppm) 6.52 (dd, *J* = 10.4, 1.9 Hz, 1H), 6.08 (d, *J* = 10.4 Hz, 1H), 5.38 – 5.28 (m, 1H), 4.47 – 4.36 (m, 1H), 4.23 (ddd, *J* = 12.0, 4.1, 1.9 Hz, 1H), 3.20 (s, 1H), 2.63 (qd, *J* = 17.3, 3.4 Hz, 2H), 2.51 – 2.41 (m, 2H), 2.41 – 2.29 (m, 2H), 2.29 – 2.18 (m, 2H), 1.75 – 1.61 (m, 3H), 1.58 (d, *J* = 7.0 Hz, 3H). **^{13}C NMR** (100 MHz, $CDCl_3$) δ (ppm) 210.6, 196.9, 195.4, 148.7, 131.5, 104.8, 93.6, 83.4, 66.8, 43.8, 42.6, 41.0, 40.6, 38.2, 27.6, 26.9, 14.7. **HRMS (EI)**: $[M]^{\oplus}$ calcd for $C_{17}H_{20}O_3^{\oplus}$ 272.1412, found 272.1408. **IR (KBr)** *ν* (cm^{-1}) 2925, 2869, 1715, 1683, 1456, 1417, 1387, 1329, 1260, 1168, 1045, 940, 918, 802, 762.

HPLC: Chiracel AD-H Column (250 mm); detected at 214 nm; *n*-hexane/*i*-propanol = 95/5; flow = 0.8 ml/min; Retention time: 22.3 min (minor), 24.8 min (major).

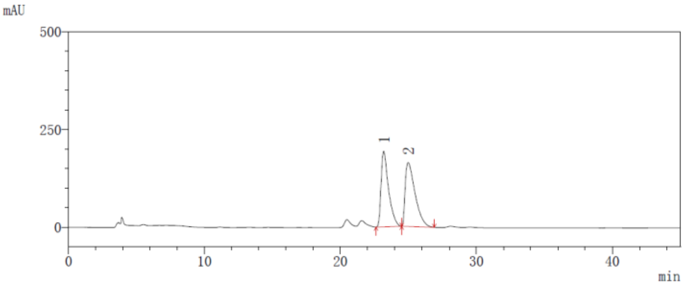

PDA Ch1 214nm

Number	Ret. Time	Area	Area%
1	23.187	7384030	47.25
2	24.984	8242062	52.75

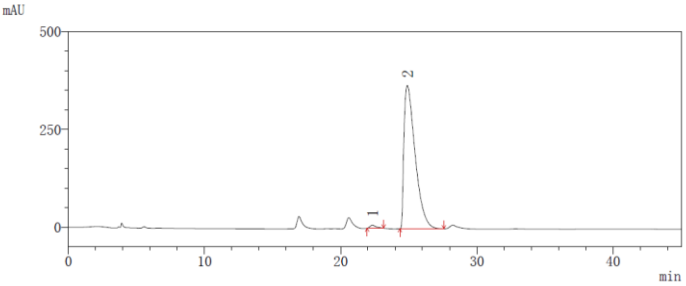

PDA Ch1 214nm

Number	Ret. Time	Area	Area%
1	22.330	236539	1.12
2	24.895	20790307	98.88

Methyl3-((3a*R*,7a*R*)-5-oxo-3-((*S*)-prop-1-en-1-ylidene)-3,3a,4,5-tetrahydrobenzofuran-7a(2*H*)-yl)propanoate (2o)

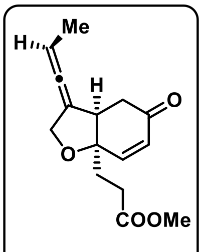

2o

colorless oil, 69% yield. R_f = 0.3 (hexane : acetone = 4 :1). $[\alpha]_D^{28.3}$ -58.5 (*c* 1.00, $CHCl_3$) for >99% *ee*. **^{1}H NMR** (400 MHz, $CDCl_3$) δ (ppm) 6.47 (dd, *J* = 10.3, 1.9 Hz, 1H), 5.98 (d, *J* = 10.3 Hz, 1H), 5.36 – 5.20 (m, 1H), 4.40 (dd, *J* = 12.0, 3.9 Hz, 1H), 4.21 (ddd, *J* = 12.0, 3.9, 1.8 Hz, 1H), 3.67 (s, 3H), 3.04 (s, 1H), 2.70 – 2.39 (m, 4H), 2.27 – 1.98 (m, 2H), 1.56 (d, *J* = 7.0 Hz, 3H). **^{13}C NMR** (100 MHz, $CDCl_3$) δ (ppm) 196.9, 195.4, 173.6, 149.5, 130.5, 103.8, 93.3, 81.2, 67.1, 51.9, 44.4, 36.9, 31.4, 28.4, 14.7. **HRMS (EI)**: $[M]^{\oplus}$ calcd for $C_{15}H_{18}O_4^{\oplus}$ 262.1205, found 262.1206. **IR (KBr)** *v* (cm^{-1}) 2951, 2925, 2856, 1738, 1688, 1438, 1471, 1414, 1382, 1317, 1198, 1171, 1072, 1028, 916, 891, 802. **HPLC**: Chiracel OD-H Column (250 mm); detected at 214 nm, at 30 °C; *n*-hexane/*i*-propanol = 95/5; flow = 0.7 ml/min; Retention time: 17.6 min (major).

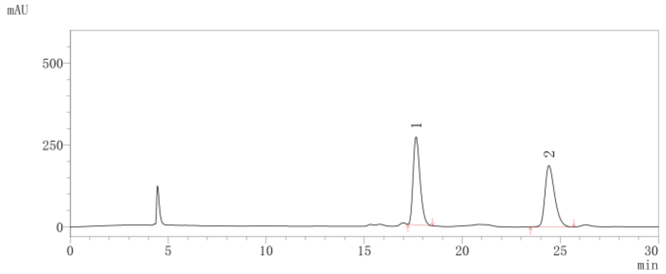

PDA Ch1 214nm

Number	Ret. Time	Area	Area%
1	17.645	6530103	50.41
2	24.420	6424583	49.59

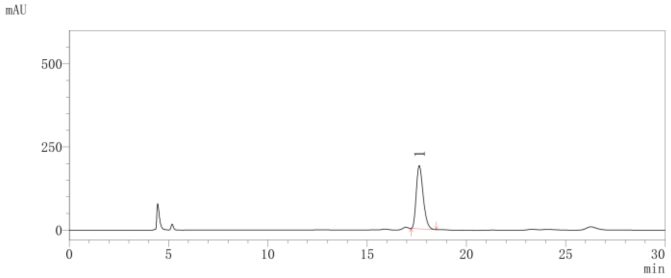

PDA Ch1 214nm

Number	Ret. Time	Area	Area%
1	17.626	4609037	100.00

(3a*R*,7a*R*)-7a-(2-((*tert*-Butyldimethylsilyl)oxy)ethyl)-3-((*S*)-prop-1-en-1-ylidene)-2,3,3a,7a-tetrahydrobenzofuran-5(4*H*)-one (2p)

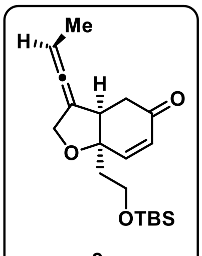

2p

colorless oil, 61% yield. R_f = 0.7 (hexane : acetone = 4 :1). $[\alpha]_D^{25.0}$ -24.9 (*c* 1.00, $CHCl_3$) for >99% *ee*. **^{1}H NMR** (400 MHz, $CDCl_3$) δ (ppm) 6.51 (dd, *J* = 10.3, 1.9 Hz, 1H), 5.96 (d, *J* = 10.3 Hz, 1H), 5.38 – 5.19 (m, 1H), 4.48 – 4.38 (m, 1H), 4.22 (ddd, *J* = 12.0, 4.1, 1.9 Hz, 1H), 3.94 – 3.73 (m, 2H), 3.22 (s, 1H), 2.68 (ddd, *J* = 18.5, 16.9, 3.3 Hz, 2H), 2.14 – 1.90 (m, 2H), 1.57 (d, *J* = 7.0 Hz, 3H), 0.87 (s, 9H), 0.05 (s, 6H). **^{13}C NMR** (100 MHz, $CDCl_3$) δ (ppm) 197.7, 195.3, 150.6, 129.9, 104.3, 93.1, 81.6, 67.0, 58.5, 45.1, 40.2, 37.1, 26.0, 18.3, 14.8, -5.3. **HRMS (EI)**: $[M]^{\oplus}$ calcd for $C_{19}H_{30}SiO_3^{\oplus}$ 334.1964, found 334.1959. **IR (KBr)** *v* (cm^{-1}) 3428, 2929, 2857, 1725, 1688, 1601, 1471, 1410, 1389, 1257, 1093, 939, 837, 811, 778. **HPLC**: Phenomenex Lux 5u Cellulose-2 (PC-2) Column (250 mm); detected at 214 nm, at 30 ºC; *n*-hexane/*i*-propanol = 98/2; flow = 0.7 ml/min; Retention time: 7.7 min (major), 7.2 min (minor).

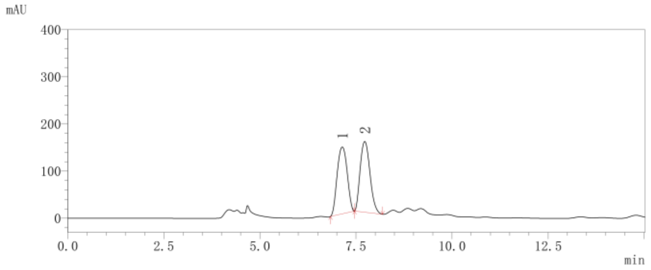

PDA Ch1 214nm

Number	Ret.Time	Area	Area%
1	7.143	2568819	49.11
2	7.727	2661463	50.89

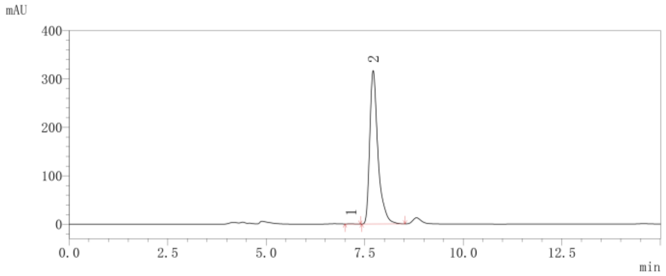

PDA Ch1 214nm

Number	Ret.Time	Area	Area%
1	7.150	3364	0.07
2	7.716	4595587	99.93

(3a*R*,7a*R*)-7a-(3-Chloropropyl)-3-((*S*)-prop-1-en-1-ylidene)-2,3,3a,7a-tetrahydrobenzofuran-5(4*H*)-one (2q)

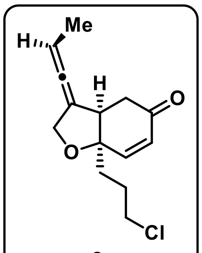

2q

colorless oil, 69% yield. R_f = 0.6 (hexane : acetone = 4 :1). $[\alpha]_D^{25.0}$ -72.7 (*c* 0.50, $CHCl_3$) for >99% *ee*. **^{1}H NMR** (400 MHz, $CDCl_3$) δ (ppm) 6.52 (dd, *J* = 10.4, 1.9 Hz, 1H), 6.00 (d, *J* = 10.4 Hz, 1H), 5.41 – 5.24 (m, 1H), 4.43 (dd, *J* = 12.1, 3.9 Hz, 1H), 4.23 (ddd, *J* = 12.0, 4.0, 1.9 Hz, 1H), 3.60 (dd, *J* = 7.9, 4.2 Hz, 2H), 3.08 (s, 1H), 2.63 (qd, *J* = 17.0, 3.5 Hz, 2H), 2.05 – 1.94 (m, 3H), 1.93 – 1.85 (m, 1H), 1.58 (d, *J* = 7.0 Hz, 3H). **^{13}C NMR** (100 MHz, $CDCl_3$) δ (ppm) 197.0, 195.4, 149.8, 130.5, 103.9, 93.3, 81.7, 67.1, 45.2, 44.9, 37.1, 34.4, 27.1, 14.7. **HRMS (EI)**: $[M]^{\oplus}$ calcd for $C_{14}H_{17}O_2Cl^{\oplus}$ 252.0917, found 252.0925. **IR (KBr)** *v* (cm^{-1}) 2924, 2856, 1972, 1687, 1443, 1412, 1384, 1257, 1234, 1178, 1075, 1030, 916, 897, 781, 654, 567, 538. **HPLC**: Chiracel ID-H

Column (250 mm); detected at 214 nm; *n*-hexane/*i*-propanol = 95/5; flow = 1.0 ml/min; Retention time: 7.6 min (major), 9.2 min (minor).

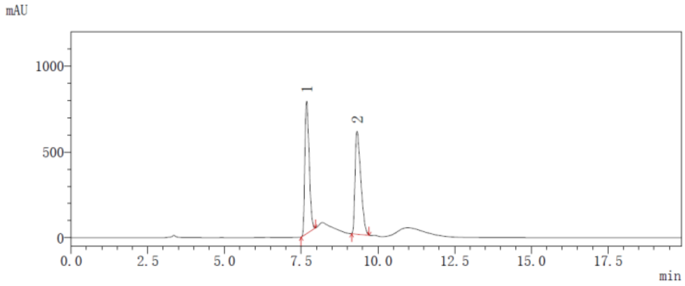

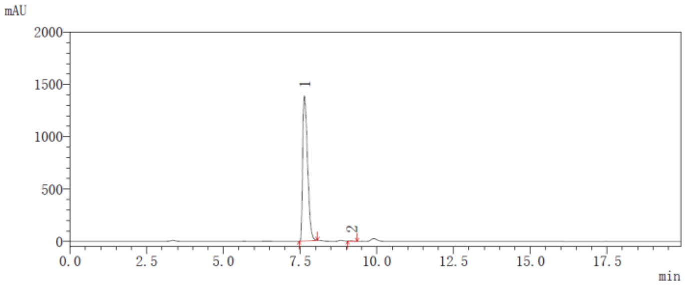

PDA Ch1 214nm

Number	Ret.Time	Area	Area%
1	7.676	7541916	49.18
2	9.318	7794305	50.82

PDA Ch1 214nm

Number	Ret.Time	Area	Area%
1	7.639	15414955	99.84
2	9.169	25242	0.16

(3a*R*,7a*R*)-7a-(3-Bromopropyl)-3-((*S*)-prop-1-en-1-ylidene)-2,3,3a,7a-tetrahydrobenzofuran-5(4*H*)-one (2r)

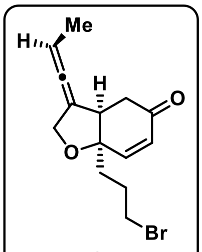

2r

colorless oil, 61% yield. R_f = 0.6 (hexane : acetone = 4 :1). $[\alpha]_D^{25.0}$ -13.3 (*c* 1.00, $CHCl_3$) for >99% *ee*. **^{1}H NMR** (400 MHz, $CDCl_3$) δ (ppm) 6.50 (dd, *J* = 10.3, 1.9 Hz, 1H), 5.99 (d, *J* = 10.3 Hz, 1H), 5.38 – 5.26 (m, 1H), 4.42 (dd, *J* = 12.1, 3.8 Hz, 1H), 4.22 (ddd, *J* = 12.0, 4.0, 1.8 Hz, 1H), 3.46 (t, *J* = 6.4 Hz, 2H), 3.07 (s, 1H), 2.62 (qd, *J* = 17.0, 3.4 Hz, 2H), 2.12 – 1.96 (m, 3H), 1.93 – 1.83 (m, 1H), 1.57 (d, *J* = 7.0 Hz, 3H). **^{13}C NMR** (100 MHz, $CDCl_3$) δ (ppm) 197.0, 195.4, 149.7, 130.5, 103.9, 93.3, 81.6, 67.1, 44.9, 37.0, 35.7, 33.8, 27.2, 14.7. **HRMS (EI)**: [M]$^\oplus$ calcd for $C_{14}H_{17}O_2Br^\oplus$ 296.0412, found 296.0404. **IR (KBr)** *v* (cm^{-1}) 3360, 2925, 2856, 1722, 1683, 1601, 1440, 1412, 1385, 1260, 1228, 1029, 912, 800. **HPLC**: Phenomenex Lux 5u Cellulose-2 (PC-2) Column (250 mm); detected at 214 nm; *n*-hexane/*i*-propanol = 95/5; flow = 0.8 ml/min; Retention time: 14.1 min (major), 16.3 min (minor).

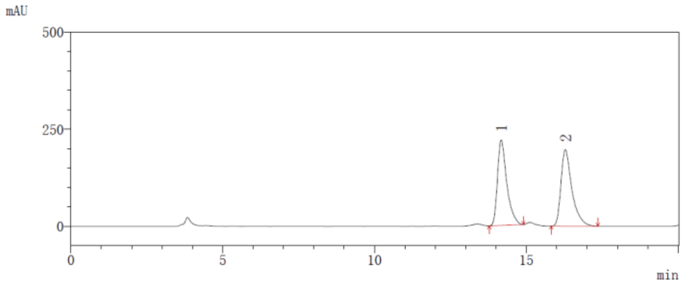

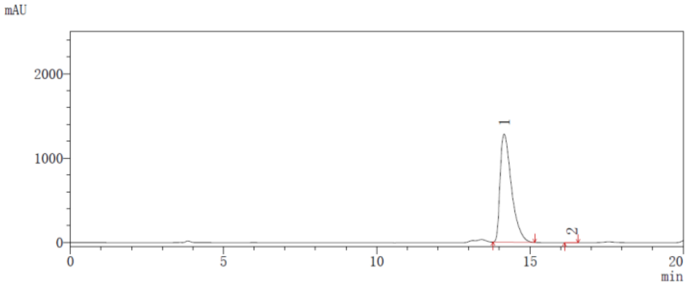

PDA Ch1 214nm

Number	Ret.Time	Area	Area%
1	14.173	4706858	48.78
2	16.283	4941580	51.22

PDA Ch1 214nm

Number	Ret.Time	Area	Area%
1	14.159	32892924	99.98
2	16.366	6377	0.02

(3a*R*,7a*R*)-7a-(3-Iodopropyl)-3-((*S*)-prop-1-en-1-ylidene)-2,3,3a,7a-tetrahydrobenzofuran-5(4*H*)-one (2s)

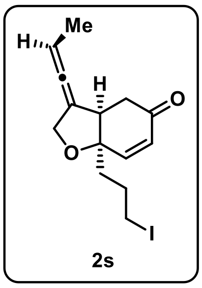

colorless oil, 64% yield. R_f = 0.6 (hexane : acetone = 4 :1). $[\alpha]_D^{24.9}$ 13.5 (*c* 1.00, $CHCl_3$) for >99% *ee*. **^{1}H NMR** (400 MHz, $CDCl_3$) δ (ppm) 6.56 – 6.44 (m, 1H), 5.99 (d, *J* = 10.3 Hz, 1H), 5.37 – 5.25 (m, 1H), 4.42 (dd, *J* = 12.0, 3.9 Hz, 1H), 4.21 (ddd, *J* = 12.0, 4.0, 1.9 Hz, 1H), 3.23 (t, *J* = 6.6 Hz, 2H), 3.07 (s, 1H), 2.62 (qd, *J* = 17.0, 3.4 Hz, 2H), 2.10 – 1.91 (m, 3H), 1.90 – 1.78 (m, 1H), 1.57 (d, *J* = 7.0 Hz, 3H). **^{13}C NMR** (100 MHz, $CDCl_3$) δ (ppm) 197.0, 195.4, 149.8, 130.5, 103.9, 93.3, 81.6, 67.1, 44.9, 38.0, 37.0, 27.9, 14.7, 6.6. **HRMS (EI)**: $[M]^{\oplus}$ calcd for $C_{14}H_{17}O_2I^{\oplus}$ 344.0273, found 344.0272. **IR (KBr)** *v* (cm^{-1}) 3418, 2924, 2855, 1723, 1683, 1601, 1443, 1411, 1385, 1260, 1172, 1036, 914, 800. **HPLC**: Chiracel ID-H Column (250 mm); detected at 214 nm; *n*-hexane/*i*-propanol = 95/5; flow = 1.0 ml/min; Retention time: 8.2 min (major), 9.2 min (minor).

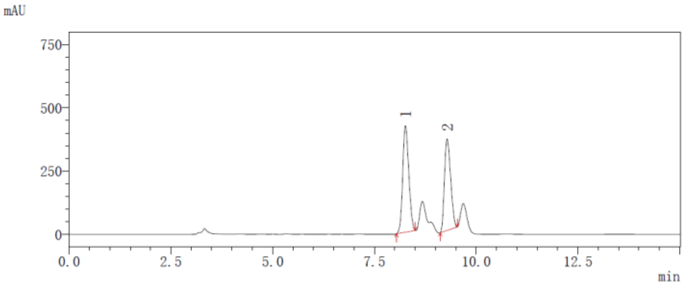

PDA Ch1 214nm

Number	Ret. Time	Area	Area%
1	8.261	4371593	53.45
2	9.286	3806992	46.55

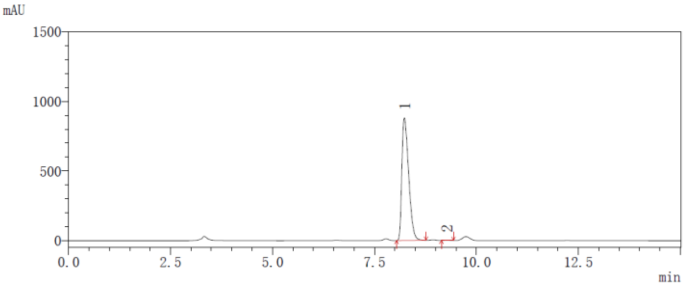

PDA Ch1 214nm

Number	Ret. Time	Area	Area%
1	8.234	10188903	99.82
2	9.273	18132	0.18

(3a*R*,7a*R*)-7a-(3-(9*H*-carbazol-9-yl)propyl)-3-((*S*)-prop-1-en-1-ylidene)-2,3,3a,7a-tetrahydrobenzofuran-5(4*H*)-one (2t)

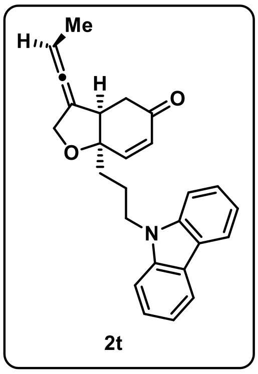

colorless oil, 61% yield. R_f = 0.6 (hexane : acetone = 4 :1). $[\alpha]_D^{25.0}$ -31.8 (*c* 2.00, $CHCl_3$) for >99% *ee*. **^{1}H NMR** (400 MHz, $CDCl_3$) δ (ppm) 8.10 (d, *J* = 7.7 Hz, 2H), 7.52 – 7.36 (m, 4H), 7.23 (dd, *J* = 9.3, 4.6 Hz, 2H), 6.45 (dd, *J* = 10.3, 1.8 Hz, 1H), 5.93 (d, *J* = 10.3 Hz, 1H), 5.38 – 5.17 (m, 1H), 4.47 – 4.31 (m, 3H), 4.24 – 4.13 (m, 1H), 2.99 (s, 1H), 2.66 – 2.38 (m, 2H), 2.09 (tt, *J* = 16.5, 8.3 Hz, 2H), 1.87 (tdd, *J* = 14.0, 12.0, 6.0 Hz, 2H), 1.55 (d, *J* = 7.0 Hz, 3H). **^{13}C NMR** (100 MHz, $CDCl_3$) δ (ppm) 197.0, 195.4, 149.7, 140.4, 130.4, 125.8, 123.0, 120.6, 119.1, 108.7, 103.9, 93.3, 81.8, 67.1, 44.9, 43.2, 37.0, 34.4, 23.5, 14.7. **HRMS (EI)**: $[M]^{\oplus}$ calcd for $C_{26}H_{25}O_2N^{\oplus}$ 383.1885, found 383.1887. **IR (KBr)** *v* (cm^{-1}) 3050, 3021, 2924, 2857, 1686, 1626, 1596, 1484, 1453, 1411, 1382, 1347, 1326, 1238, 1153, 1121, 1030, 918, 750, 724, 529. **HPLC**: Phenomenex Lux 5u Cellulose-2 (PC-2) Column

(250 mm); detected at 214 nm; *n*-hexane/*i*-propanol = 90/10; flow = 1.0 ml/min; Retention time: 10.2 min (major), 12.0 min (minor).

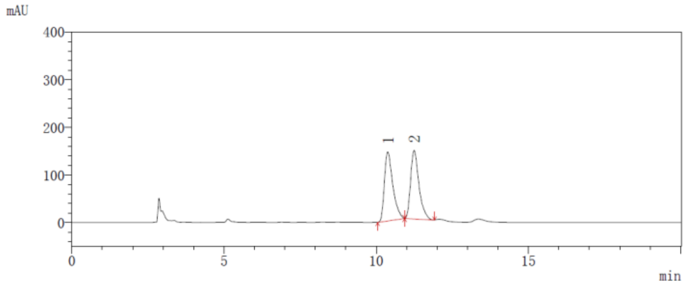

PDA Ch1 214nm

Number	Ret.Time	Area	Area%
1	10.380	2786013	50.51
2	11.247	2729333	49.49

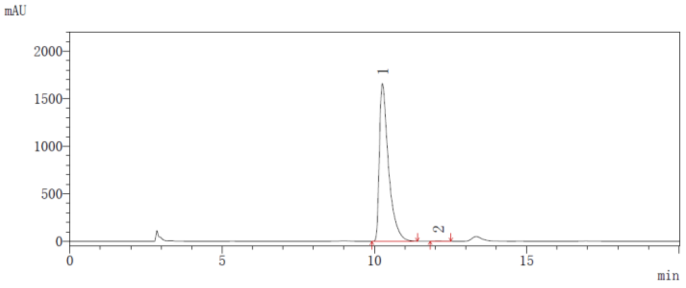

PDA Ch1 214nm

Number	Ret.Time	Area	Area%
1	10.265	34500937	99.88
2	12.091	41800	0.12

2-(3-((3a*R*,7a*R*)-5-Oxo-3-((*S*)-prop-1-en-1-ylidene)-3,3a,4,5-tetrahydrobenzofuran-7a(2*H*)-yl)propyl)isoindoline-1,3-dione (2u)

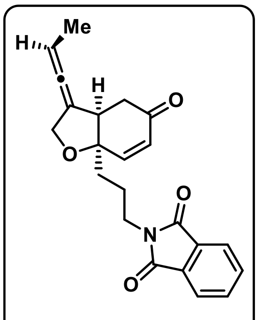

2u

yellow oil, 72% yield. R_f = 0.4 (hexane : acetone = 4 :1). $[\alpha]_D^{25.0}$ -52.3 (*c* 2.00, $CHCl_3$) for >99% *ee*. **^{1}H NMR** (400 MHz, $CDCl_3$) δ (ppm) 7.82 (dd, *J* = 4.8, 3.0 Hz, 2H), 7.77 – 7.63 (m, 2H), 6.50 (d, *J* = 10.2 Hz, 1H), 5.95 (d, *J* = 10.3 Hz, 1H), 5.28 (s, 1H), 4.37 (dd, *J* = 11.9, 3.2 Hz, 1H), 4.17 (d, *J* = 11.8 Hz, 1H), 3.73 (s, 2H), 3.04 (s, 1H), 2.71 – 2.45 (m, 2H), 1.95 – 1.73 (m, 4H), 1.54 (d, *J* = 7.0 Hz, 3H). **^{13}C NMR** (100 MHz, $CDCl_3$) δ (ppm) 197.0, 195.4, 168.4, 149.8, 134.1, 132.1, 130.4, 123.4, 104.0, 93.2, 81.6, 67.0, 44.9, 38.1, 37.1, 34.4, 23.2, 14.7. **HRMS (DART)**: $[M+H]^{\oplus}$ calcd for $C_{22}H_{22}O_4N^{\oplus}$ 364.1543, found 364.1543. **IR (KBr)** *ν* (cm^{-1}) 2925, 2857, 1771, 1712, 1686, 1466, 1437, 1397, 1361, 1188, 1046, 919, 876, 779, 720, 530. **HPLC**: Phenomenex Lux 5u Cellulose-2 (PC-2) Column (250 mm); detected at 214 nm; *n*-hexane/*i*-propanol = 90/10; flow = 1.0 ml/min; Retention time: 40.2 min (major), 48.8 min (minor).

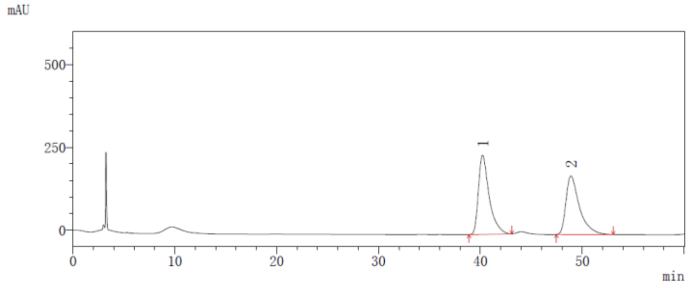

PDA Ch1 214nm

Number	Ret.Time	Area	Area%
1	40.207	17584278	51.88
2	48.882	16307948	48.12

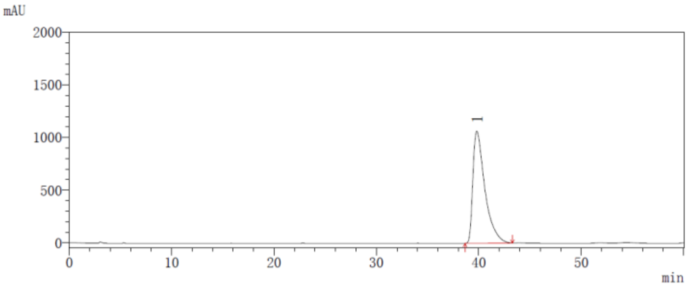

PDA Ch1 214nm

Number	Ret.Time	Area	Area%
1	39.805	86826166	100.00

(3a*R*,7a*R*)-7a-(3-(((8*R*,9*S*,13*S*,14*S*)-13-Methyl-17-oxo-7,8,9,11,12,13,14,15,16,17-decahydro-6*H*-cyclope

nta[a]phenanthren-3-yl)oxy)propyl)-3-((*R*)-prop-1-en-1-ylidene)-2,3,3a,7a-tetrahydrobenzofuran-5(4*H*)-one (2v)

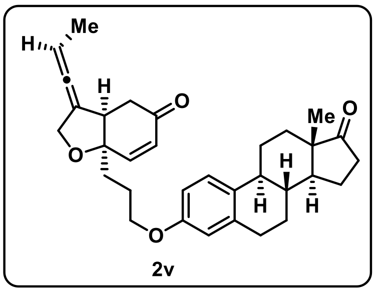

colorless oil, 73% yield. R_f = 0.4 (hexane : acetone = 4 :1). $[\alpha]_D^{25.0}$ 38.3 (*c* 2.00, $CHCl_3$) for >99% dr. **^{1}H NMR** (400 MHz, $CDCl_3$) δ (ppm) 7.19 (d, *J* = 8.6 Hz, 1H), 6.70 (dd, *J* = 8.5, 2.5 Hz, 1H), 6.63 (d, *J* = 2.2 Hz, 1H), 6.55 (dd, *J* = 10.3, 1.8 Hz, 1H), 6.01 (d, *J* = 10.3 Hz, 1H), 5.39 – 5.26 (m, 1H), 4.44 (dd, *J* = 11.9, 3.6 Hz, 1H), 4.24 (ddd, *J* = 12.0, 3.9, 1.8 Hz, 1H), 3.98 (t, *J* = 5.6 Hz, 2H), 3.11 (s, 1H), 2.89 (dd, *J* = 10.5, 4.5 Hz, 2H), 2.65 (qd, *J* = 17.0, 3.5 Hz, 2H), 2.50 (dd, *J* = 18.7, 8.6 Hz, 1H), 2.43 – 2.35 (m, 1H), 2.24 (t, *J* = 10.0 Hz, 1H), 2.19 – 2.10 (m, 1H), 2.10 – 1.89 (m, 6H), 1.59 (d, *J* = 7.0 Hz, 3H), 1.54 – 1.35 (m, 5H), 1.27 (d, *J* = 12.3 Hz, 2H), 0.90 (s, 3H). **^{13}C NMR** (100 MHz, $CDCl_3$) δ (ppm) 221.0, 197.2, 195.4, 156.9, 150.1, 137.8, 132.2, 130.3, 126.4, 114.6, 112.1, 104.1, 93.2, 81.9, 67.8, 67.0, 50.5, 48.1, 44.9, 44.0, 38.4, 37.1, 35.9, 33.7, 31.6, 29.7, 26.6, 26.0, 23.8, 21.6, 14.7, 13.9. **HRMS (DART)**: $[M+H]^{\oplus}$ calcd for $C_{32}H_{39}O_4^{\oplus}$ 487.2843, found 487.2845. **IR (KBr)** ν (cm^{-1}) 2926, 2859, 1739, 1686, 1609, 1499, 1472, 1455, 1410, 1385, 1281, 1254, 1188, 1155, 1056, 1032, 1007, 965, 918, 817, 780. **HPLC**: Chiracel OD-H Column (250 mm); detected at 214 nm; *n*-hexane/*i*-propanol = 90/10; flow = 1.0 ml/min; Retention time: 21.1 min (major), 30.9 min (minor).

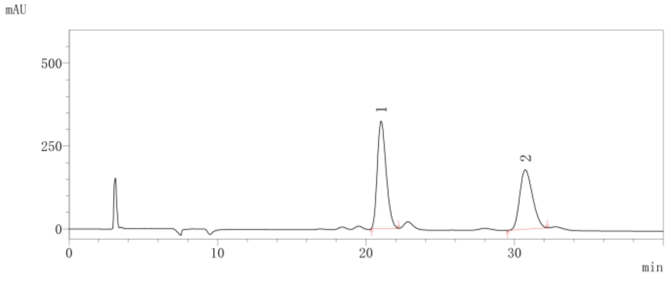

PDA Ch1 214nm

Number	Ret.Time	Area	Area%
1	21.016	13356109	55.39
2	30.725	10755343	44.61

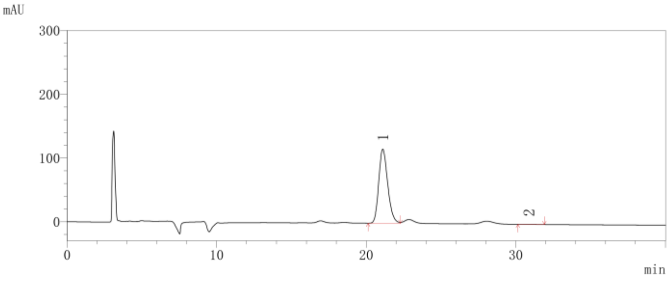

PDA Ch1 214nm

Number	Ret.Time	Area	Area%
1	21.106	4885605	99.78
2	30.870	10627	0.22

(3a*R*,7a*R*)-7a-(3-(((*R*)-2,8-dimethyl-2-((4*R*,8*R*)-4,8,12-trimethyltridecyl)chroman-6-yl)oxy)propyl)-3-((*S*)-prop-1-en-1-ylidene)-2,3,3a,7a-tetrahydrobenzofuran-5(4*H*)-one (2w)

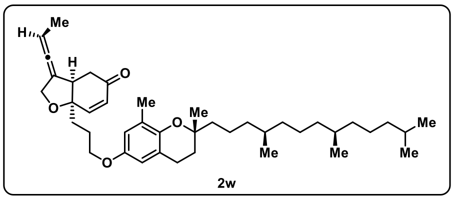

yellow oil, 46% yield. R_f = 0.4 (hexane : acetone = 15 :1). $[\alpha]_D^{25.0}$ -18.6 (*c* 1.43, $CHCl_3$) for 99.8:0.2 dr. **^{1}H NMR** (400 MHz, $CDCl_3$) δ (ppm) 6.58 – 6.53 (m, 2H), 6.43 (d, *J* = 2.8 Hz, 1H), 6.01 (d, *J* = 10.4 Hz, 1H), 5.37 – 5.27 (m, 1H), 4.45 (dd, *J* = 12.0, 3.2 Hz, 1H), 4.24 (ddd, *J* = 12.0, 4.1, 1.9 Hz, 1H), 3.92 (t, *J* = 5.6 Hz, 2H), 3.12 (s, 1H), 2.74 – 2.58 (m, 4H), 2.13 (s, 3H), 2.06 – 1.89 (m, 4H), 1.83 – 1.68 (m, 2H), 1.63 – 1.50 (m, 6H), 1.40 – 1.21 (m, 15H), 1.16 – 1.02 (m, 6H), 0.88 – 0.83

(m, 12H). **^{13}C NMR** (100 MHz, $CDCl_3$) δ (ppm) 197.3, 195.4, 151.4, 150. 2, 146.3, 130.3, 127.3, 121.1, 115.4, 111.9, 104.1, 93.1, 81.9, 75.7, 68.3, 67.0, 44.9, 40.0, 39.4, 37.5, 37.5, 37.3, 37.2, 33.8, 32.9, 32.8, 31.4, 28.1, 24.9, 24.5, 24.2, 24.0, 22.8, 22.8, 22.7, 21.1, 19.8, 19.7, 16.3, 14.7. **HRMS (DART)**: $[M+H]^{\oplus}$ calcd for $C_{41}H_{63}O_4^{\oplus}$ 619.4721, found 619.4725. **IR (KBr)** *ν* (cm^{-1}) 2924, 2853, 1688, 1470, 1377, 1219, 1152, 1060, 1032, 777. **HPLC**: Chiracel OD-H Column (250 mm); detected at 214 nm; *n*-hexane/*i*-propanol = 95/5; flow = 0.7 ml/min; Retention time: 7.5 min (major), 10.4 min (minor).

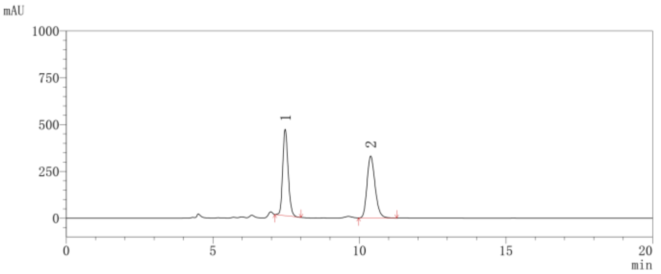

PDA Ch1 214nm

Number	Ret. Time	Area	Area%
1	7.469	5928425	48.59
2	10.386	6271647	51.41

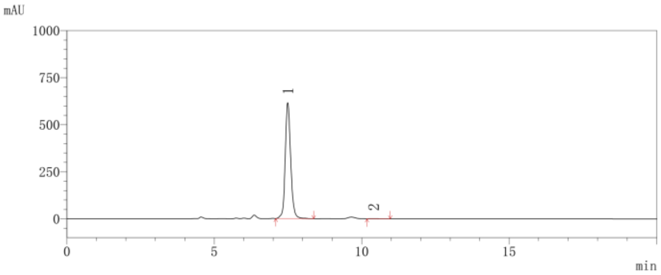

PDA Ch1 214nm

Number	Ret. Time	Area	Area%
1	7.491	7918355	99.85
2	10.402	11677	0.15

(3a*S*,7a*S*)-7a-(3-(((*R*)-2,8-dimethyl-2-((4*R*,8*R*)-4,8,12-trimethyltridecyl)chroman-6-yl)oxy)propyl)-3-((*R*)-prop-1-en-1-ylidene)-2,3,3a,7a-tetrahydrobenzofuran-5(4*H*)-one (2w')

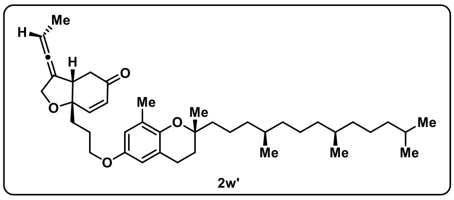

yellow oil, 45% yield. R_f = 0.4 (hexane : acetone = 15 :1). $[\alpha]_D^{25.0}$ 26.8 (*c* 1.3, $CHCl_3$) for 0.2:99.8 dr. **^{1}H NMR** (400 MHz, $CDCl_3$) δ (ppm) 6.58 – 6.53 (m, 2H), 6.43 (d, *J* = 2.8 Hz, 1H), 6.01 (d, *J* = 10.4 Hz, 1H), 5.37 – 5.27 (m, 1H), 4.45 (dd, *J* = 12.0, 3.2 Hz, 1H), 4.24 (ddd, *J* = 12.0, 4.1, 1.9 Hz, 1H), 3.92 (t, *J* = 5.6 Hz, 2H), 3.12 (s, 1H), 2.74 – 2.58 (m, 4H), 2.13 (s, 3H), 2.06 – 1.89 (m, 4H), 1.83 – 1.68 (m, 2H), 1.63 – 1.50 (m, 6H), 1.40 – 1.21 (m, 15H), 1.16 – 1.02 (m, 6H), 0.88 – 0.83 (m, 12H). **^{13}C NMR** (100 MHz, $CDCl_3$) δ (ppm) 197.3, 195.4, 151.4, 150. 2, 146.3, 130.3, 127.3, 121.1, 115.4, 111.9, 104.1, 93.1, 81.9, 75.7, 68.3, 67.0, 44.9, 40.0, 39.4, 37.5, 37.5, 37.3, 37.2, 33.8, 32.9, 32.8, 31.4, 28.1, 24.9, 24.5, 24.2, 24.0, 22.8, 22.8, 22.7, 21.1, 19.8, 19.7, 16.3, 14.7. **HRMS (DART)**: $[M+H]^{\oplus}$ calcd for $C_{41}H_{63}O_4^{\oplus}$ 619.4721, found 619.4725. **IR (KBr)** *ν* (cm^{-1}) 2925, 2865, 1689, 1469, 1377, 1220, 1152, 1060, 1032, 800. **HPLC**: Chiracel OD-H Column (250 mm); detected at 214 nm; *n*-hexane/*i*-propanol = 95/5; flow = 0.7 ml/min; Retention time: 10.4 min (major), 7.5 min (minor).

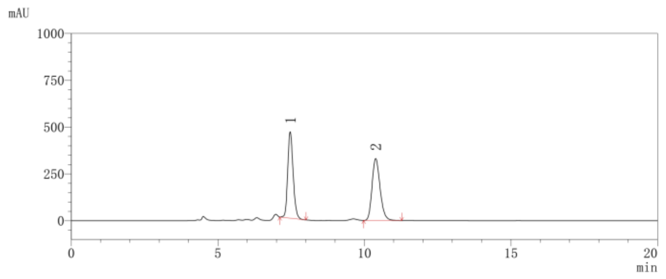

PDA Ch1 214nm

Number	Ret.Time	Area	Area%
1	7.469	5928425	48.59
2	10.386	6271647	51.41

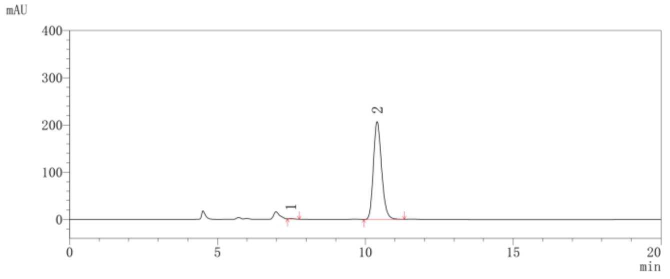

PDA Ch1 214nm

Number	Ret.Time	Area	Area%
1	7.475	8269	0.21
2	10.401	3971475	99.79

(3a*R*,7a*R*)-7a-Cyclohexyl-3-((*S*)-oct-1-en-1-ylidene)-2,3,3a,7a-tetrahydrobenzofuran-5(4H)-one (2x)

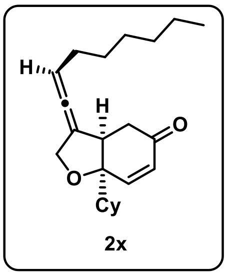

colorless oil, 25% yield. R_f = 0.8 (hexane : acetone = 5 :1). $[\alpha]_D^{25.0}$ -37.6 (*c 0*.55, $CHCl_3$) for >99% *ee*. **^{1}H NMR** (400 MHz, $CDCl_3$) δ (ppm) 6.54 (dd, *J* = 10.4, 1.9 Hz, 1H), 6.06 (d, *J* = 10.4 Hz, 1H), 5.38 – 5.30 (m, 1H), 4.41 (ddd, *J* = 11.9, 3.9, 0.9 Hz, 1H), 4.21 (ddd, *J* = 11.9, 4.2, 1.9 Hz, 1H), 3.19 (s, 1H), 2.67 – 2.61 (m, 2H), 1.98 – 1.66 (m, 8H), 1.32 – 1.12 (m, 13H), 0.88 (t, *J* = 6.9 Hz, 3H). **^{13}C NMR** (150 MHz, $CDCl_3$) δ (ppm) 197.5, 194.3, 150.2, 130.9, 105.9, 98.7, 84.2, 66.9, 45.8, 42.2, 38.2, 31.7, 29.3, 29.0, 28.9, 28.0, 27.3, 26.7, 26.4, 22.7, 14.2. **HRMS (EI)**: [M]$^\oplus$ calcd for $C_{22}H_{32}O_2^\oplus$ 328.2397, found 328.2389. **IR (KBr)** *v* (cm^{-1}) 2926, 2853, 1688, 1451, 1385, 1269, 1235, 1130, 1046, 957, 885. **HPLC**: Phenomenex Lux 5u Cellulose-2 (PC-2) Column (250 mm); detected at 214 nm; *n*-hexane/*i*-propanol = 98/2; flow = 0.7 ml/min; Retention time: 7.7 min (major).

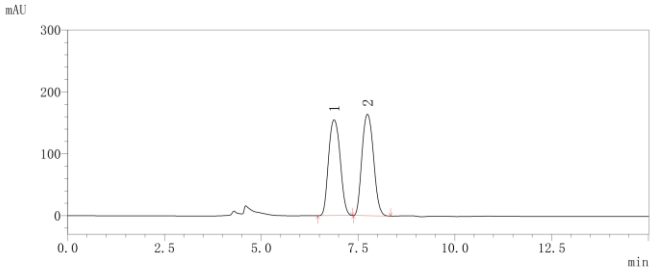

PDA Ch1 214nm

Number	Ret.Time	Area	Area%
1	6.882	3225264	48.50
2	7.744	3425394	51.50

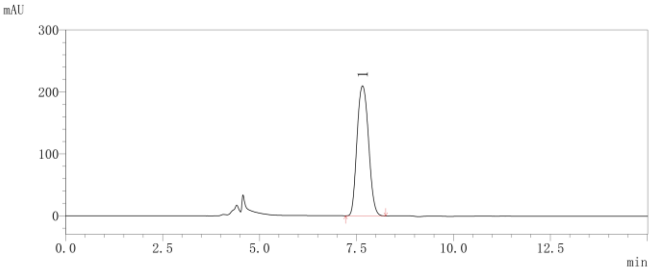

PDA Ch1 214nm

Number	Ret.Time	Area	Area%
1	7.657	4349456	100.00

(3a*R*,7a*R*)-3-((*S*)-4-(Benzyloxy)but-1-en-1-ylidene)-7a-cyclohexyl-2,3,3a,7a-tetrahydrobenzofuran-5(4 H)-one (2y)

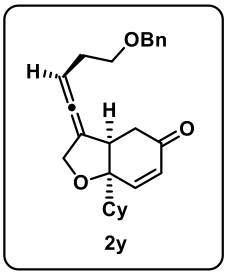

colorless oil, 33% yield. R_f = 0.6 (hexane : acetone = 5 :1). $[\alpha]_D^{25.0}$ -14.5 (*c 0*.48, $CHCl_3$) for 99% *ee*. **^{1}H NMR** (400 MHz, $CDCl_3$) δ (ppm) 7.36 – 7.27 (m, 5H), 6.53 (dd, *J* = 10.4, 1.8 Hz, 1H), 6.05 (d, *J* = 10.4 Hz, 1H), 5.46 – 5.38 (m, 1H), 4.53 – 4.45 (m, 2H), 4.41 (dd, *J* = 12.0, 3.3 Hz, 1H), 4.19 (ddd, *J* = 12.0, 4.2, 1.9 Hz, 1H), 3.47 (t, *J* = 6.5 Hz, 2H), 3.20 (s, 1H), 2.64 (d, *J* = 3.8 Hz, 2H), 2.25 (q, *J* = 6.6 Hz, 2H), 1.98 – 1.66 (m, 6H),

1.30 – 1.12 (m, 5H). **^{13}C NMR** (125 MHz, $CDCl_3$) δ (ppm) 197.7, 194.9, 150.2, 138.5, 130.9, 128.4, 127.8, 127.6, 106.3, 95.2, 84.3, 72.9, 69.5, 66.8, 45.7, 42.2, 38.4, 29.7, 28.0, 27.2, 26.6, 26.4. **HRMS (EI)**: [M]$^{\oplus}$ calcd for $C_{25}H_{30}O_3^{\oplus}$ 378.2189, found 378.2187. **IR (KBr)** *v* (cm^{-1}) 2925, 2852, 1738, 1686, 1452, 1372, 1269, 1239, 1103, 1046, 736. **HPLC**: Chiracel OD-H Column (250 mm); detected at 214 nm; *n*-hexane/*i*-propanol = 95/5; flow = 0.7 ml/min; Retention time: 9.0 min (major), 10.1 min (minor).

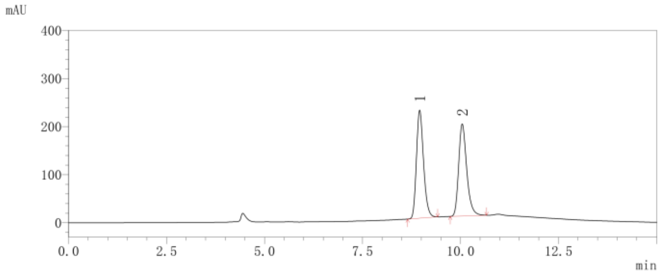

PDA Ch1 214nm

Number	Ret.Time	Area	Area%
1	8.959	2828836	50.76
2	10.046	2744515	49.24

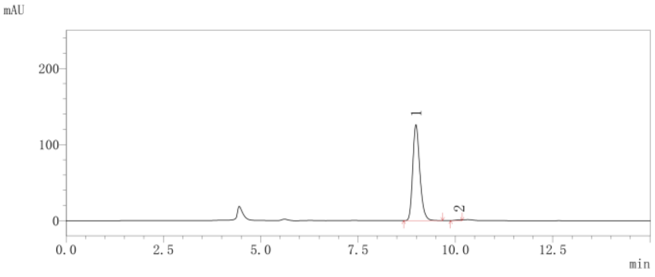

PDA Ch1 214nm

Number	Ret.Time	Area	Area%
1	8.989	1593870	99.40
2	10.094	9613	0.60

(3a*R*,7a*R*)-7a-Methyl-3-((*S*)-prop-1-en-1-ylidene)-1,2,3,3a,4,7a-hexahydro-5*H*-indol-5-one (5a)

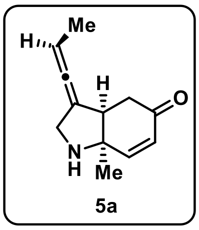

colorless oil, 74% yield. R_f = 0.2 (hexane : acetone = 3 :1). $[\alpha]_D^{25.0}$ -90.3 (*c* 1.00, $CHCl_3$) for >99% *ee*. **^{1}H NMR** (400 MHz, $CDCl_3$) δ (ppm) 6.44 (dd, *J* = 10.2, 1.9 Hz, 1H), 5.92 (d, *J* = 10.2 Hz, 1H), 5.28 – 5.16 (m, 1H), 3.64 (dd, *J* = 14.6, 3.0 Hz, 1H), 3.51 – 3.36 (m, 1H), 2.83 (s, 1H), 2.61 (d, *J* = 3.7 Hz, 2H), 1.98 (s, 1H), 1.56 (d, *J* = 7.0 Hz, 3H), 1.42 (s, 3H). **^{13}C NMR** (100 MHz, $CDCl_3$) δ (ppm) 197.7, 196.9, 153.6, 129.6, 105.3, 91.6, 61.4, 48.4, 47.8, 37.2, 23.8, 14.8. **HRMS (EI)**: [M]$^{\oplus}$ calcd for $C_{12}H_{15}ON^{\oplus}$ 189.1154, found 189.1158. **IR (KBr)** *v* (cm^{-1}) 3310, 2924, 1682, 1455, 1412, 1258, 1153, 1078, 1042, 978, 915, 797. **HPLC**: Chiracel AS-H Column (250 mm); detected at 214 nm; *n*-hexane/*i*-propanol = 97.5/2.5; flow = 0.8 ml/min; Retention time: 12.3 min (major), 21.7 min (minor).

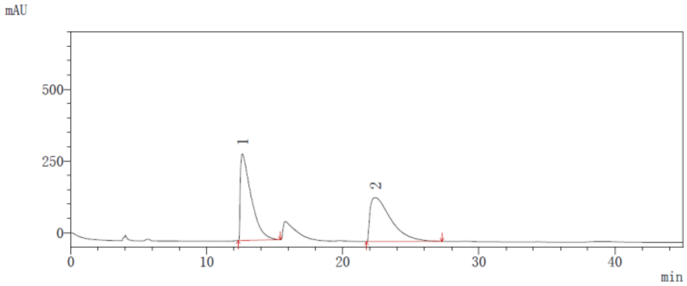

PDA Ch1 214nm

Number	Ret.Time	Area	Area%
1	12.612	16730876	50.71
2	22.377	16264484	49.29

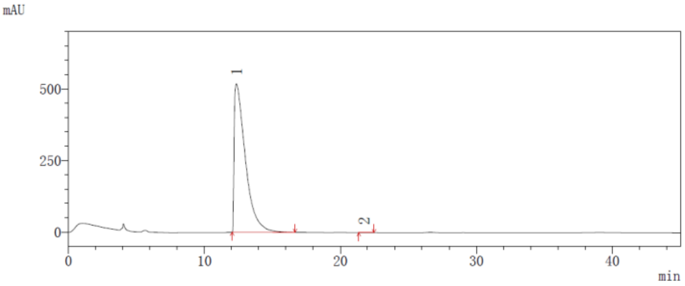

PDA Ch1 214nm

Number	Ret.Time	Area	Area%
1	12.358	29572955	99.96
2	21.726	12851	0.04

(3a*R*,7a*R*)-7a-Butyl-3-((*S*)-prop-1-en-1-ylidene)-1,2,3,3a,4,7a-hexahydro-5*H*-indol-5-one (5b)

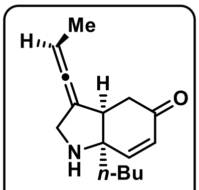

5b

colorless oil, 74% yield. R_f = 0.6 (hexane : acetone = 4 :1). $[\alpha]_D^{25.0}$ -58.0 (*c* 1.00, $CHCl_3$) for 98% *ee*. **^{1}H NMR** (400 MHz, $CDCl_3$) δ (ppm) 6.49 (dd, *J* = 10.3, 1.9 Hz, 1H), 5.97 (d, *J* = 10.3 Hz, 1H), 5.32 – 5.03 (m, 1H), 3.62 (dd, *J* = 14.4, 2.5 Hz, 1H), 3.43 (d, *J* = 13.8 Hz, 1H), 2.87 (s, 1H), 2.60 (d, *J* = 3.8 Hz, 2H), 2.06 (s, 1H), 1.81 (ddd, *J* = 13.7, 10.8, 6.1 Hz, 1H), 1.67 – 1.60 (m, 1H), 1.56 (d, *J* = 7.0 Hz, 3H), 1.47 – 1.28 (m, 4H), 0.90 (t, *J* = 7.2 Hz, 3H). **^{13}C NMR** (100 MHz, $CDCl_3$) δ (ppm) 197.9, 196.9, 152.9, 130.2, 105.5, 91.7, 64.0, 47.6, 46.9, 38.3, 37.6, 26.6, 23.5, 14.8, 14.1. **HRMS (EI)**: $[M]^{\oplus}$ calcd for $C_{15}H_{21}ON^{\oplus}$ 231.1623, found 231.1620. **IR (KBr)** *v* (cm^{-1}) 3324, 2956, 2930, 2860, 1683, 1458, 1413, 1380, 1242, 1151, 1073, 919, 801, 774. **HPLC**: Chiracel AS-H Column (250 mm); detected at 214 nm; *n*-hexane/*i*-propanol = 95/5; flow = 0.8 ml/min; Retention time: 6.9 min (major), 9.6 min (minor).

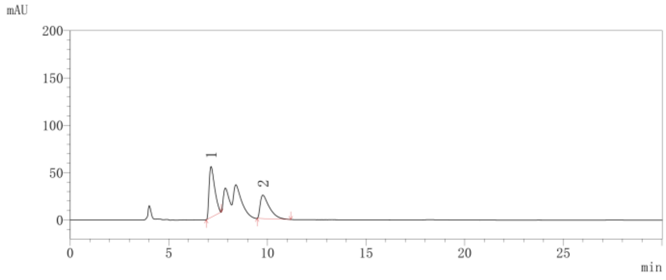

PDA Ch1 214nm

Number	Ret. Time	Area	Area%
1	7.146	1068655	57.29
2	9.772	796738	42.71

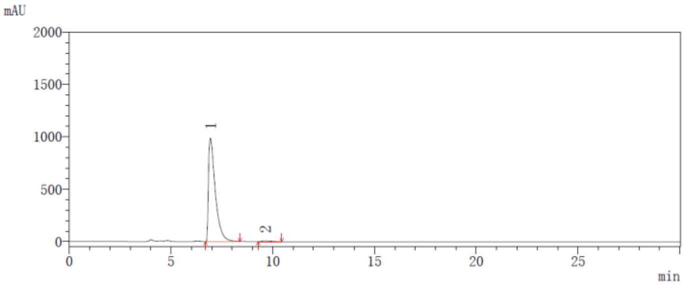

PDA Ch1 214nm

Number	Ret. Time	Area	Area%
1	6.938	23036992	99.05
2	9.618	220077	0.95

(3a*R*,7a*R*)-7a-Phenyl-3-((*S*)-prop-1-en-1-ylidene)-1,2,3,3a,4,7a-hexahydro-5*H*-indol-5-one (5c)

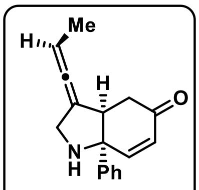

5c

colorless oil, 70% yield. R_f = 0.5 (hexane : acetone = 4 :1). $[\alpha]_D^{25.0}$ -48.5 (*c* 0.50, $CHCl_3$) for 96% *ee*. **^{1}H NMR** (400 MHz, $CDCl_3$) δ (ppm) 7.59 (d, *J* = 7.4 Hz, 2H), 7.39 (t, *J* = 7.4 Hz, 2H), 7.33 (t, *J* = 7.2 Hz, 1H), 6.55 (dd, *J* = 10.2, 1.8 Hz, 1H), 6.21 (d, *J* = 10.2 Hz, 1H), 5.33 – 5.21 (m, 1H), 3.87 (dd, *J* = 13.2, 3.0 Hz, 1H), 3.67 (dd, *J* = 13.3, 2.2 Hz, 1H), 3.13 (s, 1H), 2.58 (d, *J* = 3.6 Hz, 2H), 1.85 (s, 1H), 1.60 (d, *J* = 7.0 Hz, 3H). **^{13}C NMR** (100 MHz, $CDCl_3$) δ (ppm) 198.2, 197.2, 151.1, 141.4, 130.5, 128.9, 128.2, 126.3, 104.4, 92.0, 66.3, 48.9, 47.2, 36.9, 14.8. **HRMS (EI)**: $[M]^{\oplus}$ calcd for $C_{17}H_{17}ON^{\oplus}$ 251.1310, found 251.1304. **IR (KBr)** *v* (cm^{-1}) 3301, 3059, 2922, 2855, 1968, 1681, 1489, 1447, 1407, 1261, 1245, 1176, 1125, 1030, 918, 799, 768, 700, 549. **HPLC**: Phenomenex Lux 5u Cellulose-2 (PC-2) Column (250 mm); detected at 214 nm; *n*-hexane/*i*-propanol = 95/5; flow = 0.8 ml/min; Retention time: 13.0 min (major), 15.7 min (minor).

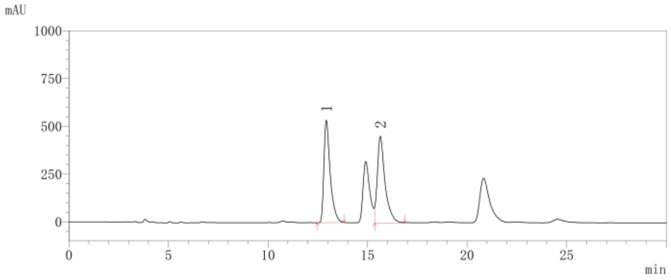

PDA Ch1 214nm

Number	Ret. Time	Area	Area%
1	12.928	11741286	48.80
2	15.638	12320513	51.20

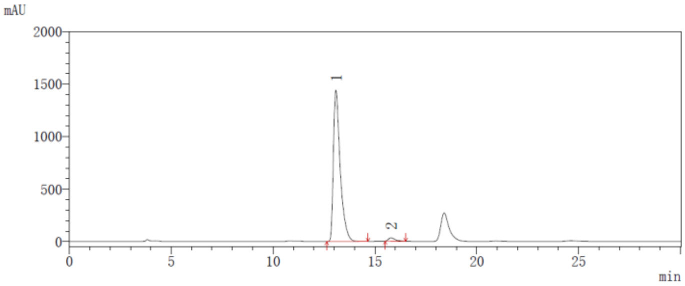

PDA Ch1 214nm

Number	Ret. Time	Area	Area%
1	13.086	33808785	97.75
2	15.797	776806	2.25

(3a*R*,7a*S*)-7a-Methoxy-3-((*R*)-prop-1-en-1-ylidene)-1,2,3,3a,4,7a-hexahydro-5*H*-inden-5-one (5d)

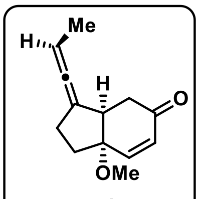

5d

colorless oil, 56% yield. R_f = 0.7 (hexane : acetone = 4 :1). $[\alpha]_D^{25.0}$ 64.9 (*c* 1.00, $CHCl_3$) for >99% *ee*. **^{1}H NMR** (400 MHz, $CDCl_3$) δ (ppm) 6.66 (dd, *J* = 10.3, 1.3 Hz, 1H), 6.10 (d, *J* = 10.3 Hz, 1H), 5.27 – 5.07 (m, 1H), 3.36 (s, 3H), 3.19 – 3.10 (m, 1H), 2.70 (dd, *J* = 16.7, 5.5 Hz, 1H), 2.62 – 2.47 (m, 2H), 2.33 (ddddd, *J* = 12.9, 10.6, 8.6, 4.2, 2.0 Hz, 1H), 2.09 – 1.91 (m, 2H), 1.57 (d, *J* = 7.0 Hz, 3H). **^{13}C NMR** (100 MHz, $CDCl_3$) δ (ppm) 199.2, 198.2, 147.8, 132.2, 104.2, 90.2, 81.1, 52.2, 45.2, 39.3, 35.7, 27.0, 14.9. **HRMS (EI)**: $[M]^{\oplus}$ calcd for $C_{13}H_{16}O_2^{\oplus}$ 204.1150, found 204.1154. **IR (KBr)** *ν* (cm^{-1}) 2930, 2826, 1687, 1491, 1458, 1390, 1261, 1235, 1152, 1082, 1020, 965, 907, 861, 795, 734. **HPLC**: Chiracel AS-H Column (250 mm); detected at 214 nm; *n*-hexane/*i*-propanol = 95/5; flow = 0.8 ml/min; Retention time: 11.1 min (major), 14.4 min (minor).

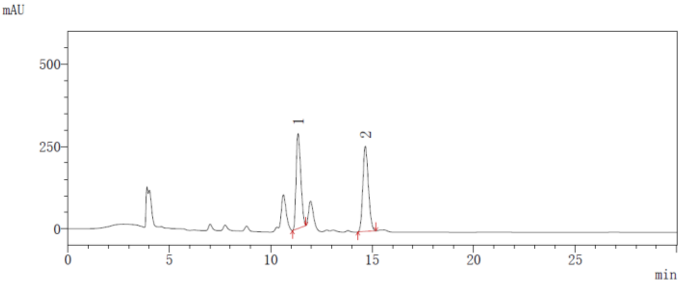

PDA Ch1 214nm

Number	Ret. Time	Area	Area%
1	11.343	4638673	48.70
2	14.657	4886712	51.30

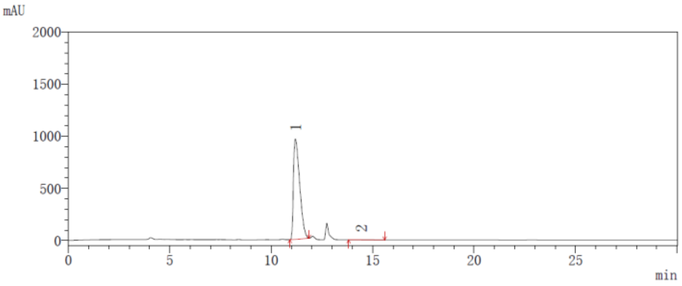

PDA Ch1 214nm

Number	Ret. Time	Area	Area%
1	11.192	20579945	99.69
2	14.434	63468	0.31

(3a*R*,7a*S*)-7a-(2-Bromoethoxy)-3-((*R*)-prop-1-en-1-ylidene)-1,2,3,3a,4,7a-hexahydro-5*H*-inden-5-one (5e)

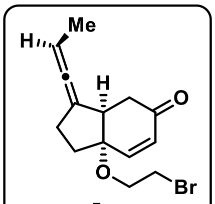

5e

colorless oil, 57% yield. R_f = 0.4 (hexane : acetone = 4 :1). $[\alpha]_D^{25.0}$ 54.9 (*c* 2.00, $CHCl_3$) for >99% *ee*. **^{1}H NMR** (400 MHz, $CDCl_3$) δ (ppm) 6.65 (dd, *J* = 10.3, 1.3 Hz, 1H), 6.10 (d, *J* = 10.3 Hz, 1H), 5.23 – 5.12 (m, 1H), 3.85 (dt, *J* = 10.1, 5.9 Hz, 1H), 3.82 – 3.73 (m, 1H), 3.43 (t, *J* = 6.1 Hz, 2H), 3.16 (d, *J* = 4.6 Hz, 1H), 2.70 (dd, *J* = 16.7, 5.5 Hz, 1H), 2.62 – 2.48 (m, 2H), 2.34 (ddddd, *J* = 12.8, 10.6, 8.6, 4.1, 2.0 Hz, 1H), 2.11 – 1.92 (m, 2H), 1.57 (d, *J* = 7.0

Hz, 3H). **^{13}C NMR** (100 MHz, $CDCl_3$) δ (ppm) 199.2, 198.0, 147.4, 132.2, 103.9, 90.4, 81.4, 64.8, 45.7, 39.2, 36.0, 31.1, 27.1, 14.9. **HRMS (EI)**: $[M]^{\oplus}$ calcd for $C_{14}H_{17}O_2Br^{\oplus}$ 296.0412, found 296.0415. **IR (KBr)** *ν* (cm^{-1}) 2925, 2854, 1718, 1687, 1457, 1382, 1261, 1236, 1184, 1102, 964, 909, 798. **HPLC**: Chiracel AS-H Column (250 mm); detected at 214 nm; *n*-hexane/*i*-propanol = 95/5; flow = 0.8 ml/min; Retention time: 14.0 min (major), 13.2 min (minor).

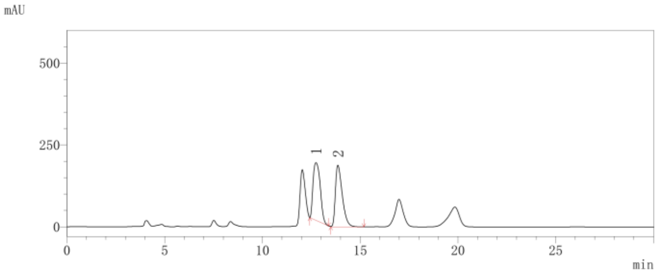

PDA Ch1 214nm

Number	Ret.Time	Area	Area%
1	12.741	4477484	49.403
2	13.861	4585729	50.597

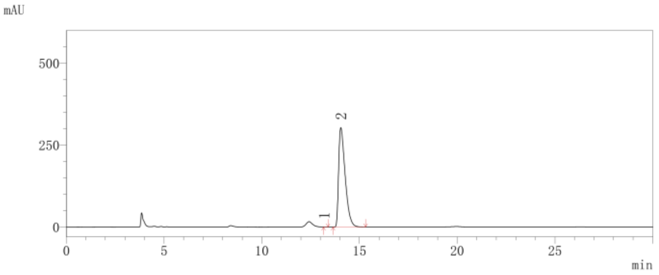

PDA Ch1 214nm

Number	Ret.Time	Area	Area%
1	13.181	138	0.002
2	14.043	7042346	99.998

(3a*R*,7a*S*)-7a-(2-Iodoethoxy)-3-((*R*)-prop-1-en-1-ylidene)-1,2,3,3a,4,7a-hexahydro-5*H*-inden-5-one (5f)

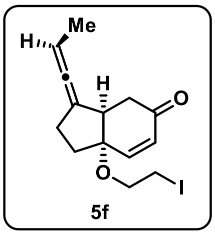

colorless oil, 52% yield. R_f = 0.5 (hexane : acetone = 3 :1). $[\alpha]_D^{25.0}$ 62.4 (*c* 1.00, $CHCl_3$) for >99% *ee*. **^{1}H NMR** (400 MHz, $CDCl_3$) δ (ppm) 6.65 (dd, *J* = 10.3, 1.3 Hz, 1H), 6.10 (d, *J* = 10.3 Hz, 1H), 5.23 – 5.13 (m, 1H), 3.83 – 3.63 (m, 2H), 3.21 (dt, *J* = 6.4, 5.4 Hz, 2H), 2.70 (dd, *J* = 16.7, 5.5 Hz, 1H), 2.62 – 2.47 (m, 2H), 2.34 (dtdd, *J* = 10.6, 8.6, 4.2, 2.1 Hz, 1H), 2.01 (tdd, *J* = 17.4, 8.7, 4.2 Hz, 2H), 1.57 (d, *J* = 7.0 Hz, 3H). **^{13}C NMR** (100 MHz, $CDCl_3$) δ (ppm) 199.2, 198.0, 147.5, 132.2, 103.9, 90.4, 81.4, 65.4, 45.8, 39.2, 36.0, 27.1, 14.9, 3.8. **HRMS (EI)**: $[M]^{\oplus}$ calcd for $C_{14}H_{17}O_2I^{\oplus}$ 344.0273, found 344.0267. **IR (KBr)** *ν* (cm^{-1}) 2925, 2853, 1686, 1457, 1412, 1382, 1262, 1236, 1154, 1100, 966, 908, 799. **HPLC**: Chiracel OJ-H Column (250 mm); detected at 214 nm; *n*-hexane/*i*-propanol = 98.5/1.5; flow = 0.8 ml/min; Retention time: 13.5 min (minor), 14.7 min (major).

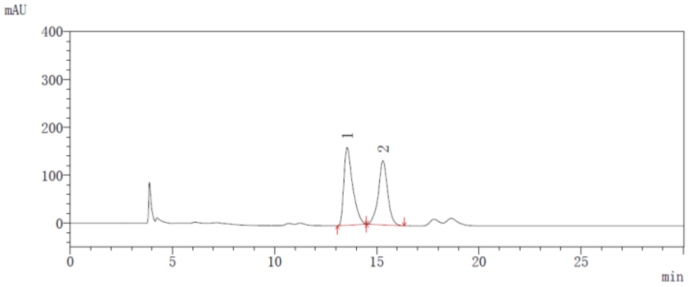

PDA Ch1 214nm

Number	Ret.Time	Area	Area%
1	13.551	4856676	53.34
2	15.302	4248394	46.66

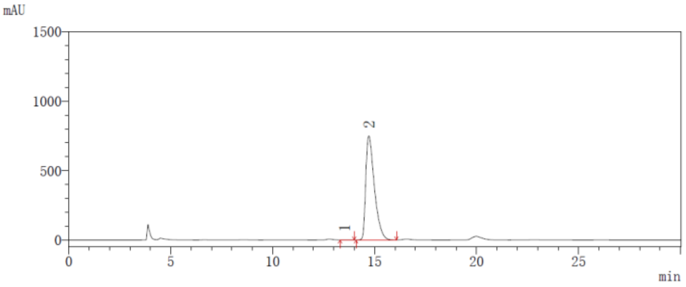

PDA Ch1 214nm

Number	Ret.Time	Area	Area%
1	13.513	18808	0.09
2	14.716	22025687	99.91

(*S*)-1-(Hexa-3,4-dien-1-yloxy)-[1,1'-bi(cyclohexane)]-2,5-dien-4-one (5g)

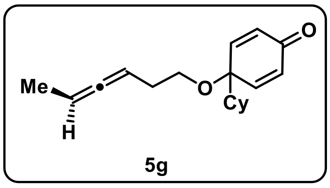

yellow oil, 47% yield. R_f = 0.8 (hexane : acetone = 5 :1). $[\alpha]_D^{25.0}$ 22.7 (*c* 0.7, $CHCl_3$) for 98% *ee*. **^{1}H NMR** (400 MHz, $CDCl_3$) δ (ppm) 6.76 (d, *J* = 10.0 Hz, 2H), 6.35 (d, *J* = 10.1 Hz, 2H), 5.10 – 4.98 (m, 2H), 3.35 (t, *J* = 6.5 Hz, 2H), 2.24 – 2.15 (m, 2H), 1.89 (d, *J* = 12.1 Hz, 2H), 1.75 (d, *J* = 12.8 Hz, 2H), 1.65 – 1.59 (m, 4H), 1.27 – 1.04 (m, 4H), 0.97 – 0.86 (m, 2H). **^{13}C NMR** (100 MHz, $CDCl_3$) δ (ppm) 205.3, 186.1, 151.3, 131.6, 86.8, 85.9, 77.8, 64.6, 46.8, 30.2, 27.4, 26.5, 14.6. **HRMS (EI)**: $[M]^{\oplus}$ calcd for $C_{18}H_{24}O_2^{\oplus}$ 272.1771, found 272.1765. **IR (KBr)** *v* (cm^{-1}) 2927, 2855, 1672, 1630, 1451, 1381, 1263, 1084, 848, 799. **HPLC**: Chiracel IF-3 Column (250 mm); detected at 214 nm; *n*-hexane/*i*-propanol = 99/1; flow = 0.5 ml/min; Retention time: 21.5 min (minor), 20.0 min (major).

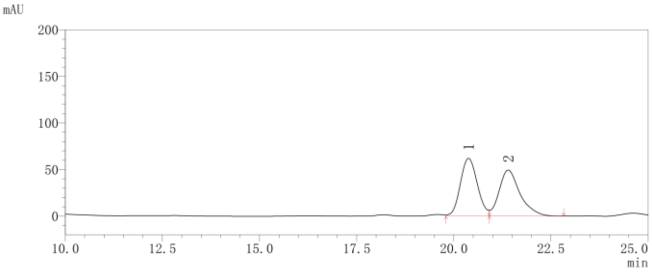

PDA Ch1 214nm

Number	Ret.Time	Area	Area%
1	20.377	1876929	50.62
2	21.396	1830776	49.38

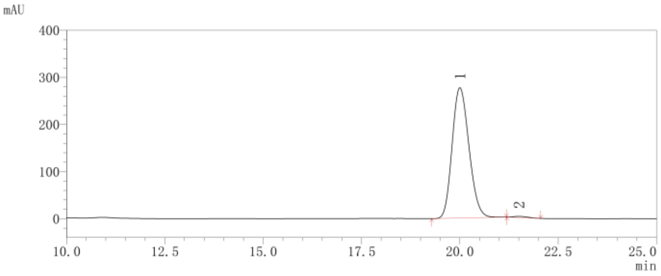

PDA Ch1 214nm

Number	Ret.Time	Area	Area%
1	20.000	8241772	99.24
2	21.498	62849	0.76

(*S*)-2-(Hepta-4,5-dien-1-yl)-2-methylcyclohexane-1,3-dione (5h)

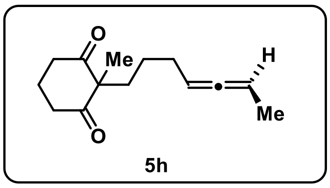

colorless oil, 75% yield. R_f = 0.8 (hexane : acetone = 4 :1). $[\alpha]_D^{25.0}$ 17.5 (*c* 0.2, $CHCl_3$) for 99% *ee*. **^{1}H NMR** (400 MHz, $CDCl_3$) δ (ppm) 5.09 – 5.01 (m, 1H), 5.01 – 4.93 (m, 1H), 2.77 – 2.67 (m, 2H), 2.65 – 2.56 (m, 2H), 2.08 – 1.98 (m, 1H), 1.93 (qd, *J* = 7.0, 3.0 Hz, 2H), 1.89 – 1.79 (m, 3H), 1.64 (dd, *J* = 6.9, 3.2 Hz, 3H), 1.29 – 1.21 (m, 5H). **^{13}C NMR** (125 MHz, $CDCl_3$) δ (ppm) 210.4, 204.8, 89.4, 86.1, 65.9, 38.0, 37.3, 28.9, 24.1, 18.5, 17.8, 14.7. **HRMS (EI)**: $[M]^{\oplus}$ calcd for $C_{14}H_{20}O_2^{\oplus}$ 220.1458, found 220.1452. **IR (KBr)** *v* (cm^{-1}) 2929, 2851, 1726, 1695, 1461, 1380, 1326, 1268, 1025, 877. **HPLC**: Chiracel IE Column (250 mm); detected at 214 nm; *n*-hexane/*i*-propanol = 95/5; flow = 0.7 ml/min; Retention time: 12.8 min (minor), 13.4 min (major).

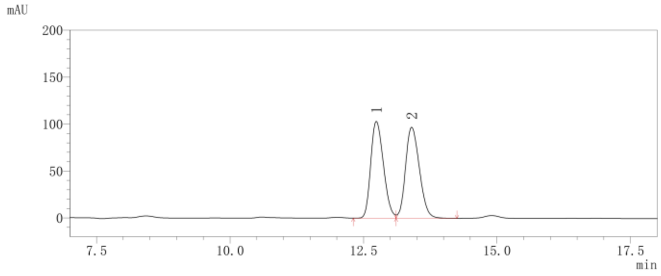

PDA Ch1 214nm

Number	Ret.Time	Area	Area%
1	12.740	1708789	49.37
2	13.400	1752605	50.63

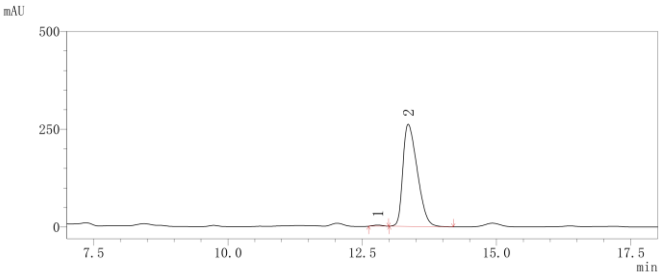

PDA Ch1 214nm

Number	Ret.Time	Area	Area%
1	12.789	34146	0.70
2	13.356	4848606	99.30

(*S*)-2-Methyl-2-(penta-2,3-dien-1-yl)cyclopentane-1,3-dione (5i)

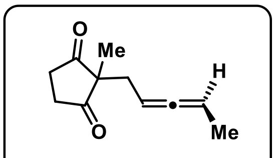

5i

colorless oil, 55% yield. R_f = 0.5 (hexane : acetone = 4 :1). $[\alpha]_D^{25.0}$ 52.0 (*c* 0.39, $CHCl_3$) for 99% *ee*. **^{1}H NMR** (400 MHz, $CDCl_3$) δ (ppm) 5.08 – 4.98 (m, 1H), 4.96 – 4.86 (m, 1H), 2.86 – 2.63 (m, 4H), 2.48 – 2.37 (m, 2H), 1.61 – 1.54 (m, 3H), 1.14 (s, 3H). **^{13}C NMR** (125 MHz, $CDCl_3$) δ (ppm) 216.2, 215.8, 205.7, 88.2, 85.6, 56.6, 35.1, 34.9, 34.6, 20.8, 14.4. **HRMS (EI)**: $[M]^{\oplus}$ calcd for $C_{11}H_{14}O_2^{\oplus}$ 178.0988, found 178.0986. **IR (KBr)** *ν* (cm^{-1}) 2964, 2926, 1765, 1723, 1452, 1421, 1370, 1261, 1065, 1021, 798. **HPLC**: Chiracel IE Column (250 mm); detected at 214 nm; *n*-hexane/*i*-propanol = 95/5; flow = 0.7 ml/min; Retention time: 14.2 min (minor), 15.1 min (major).

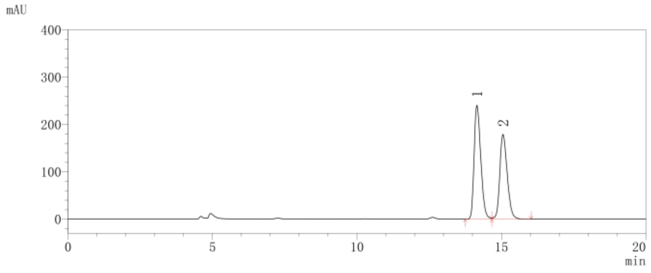

PDA Ch1 214nm

Number	Ret.Time	Area	Area%
1	14.152	3867520	54.66
2	15.053	3208264	45.34

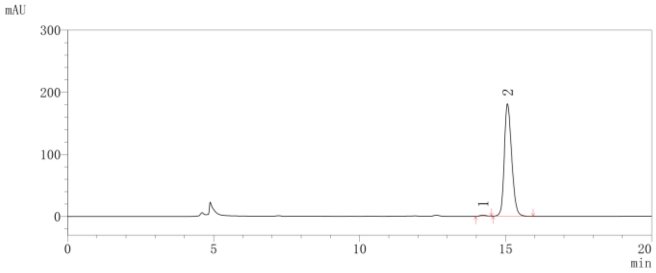

PDA Ch1 214nm

Number	Ret.Time	Area	Area%
1	14.223	22587	0.71
2	15.064	3176931	99.29

X-ray crystal structures of 2k

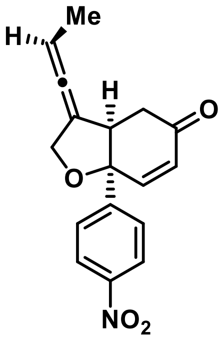

2k

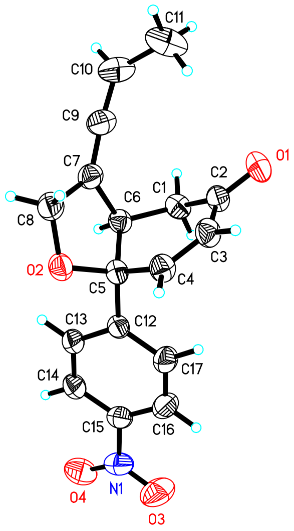

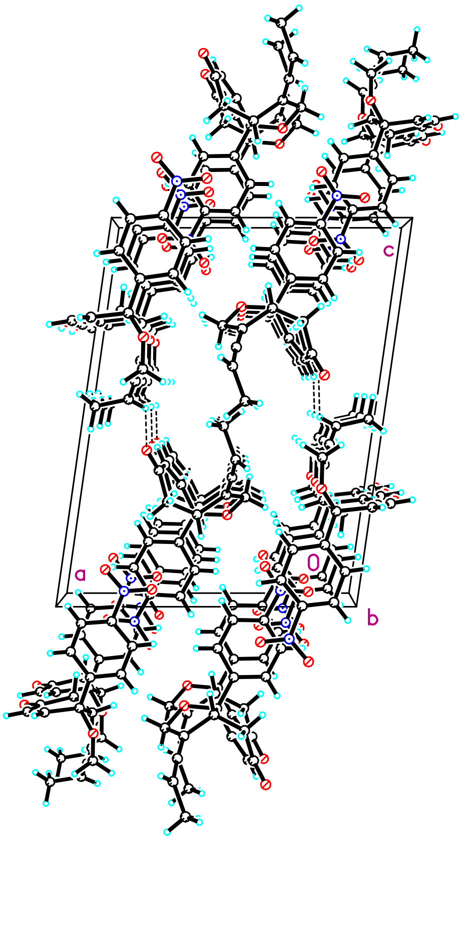

Supplementary Figure 1. X-ray crystal structures of **2k**

Supplementary Table 1. Crystal data and structure refinement for **2k**.

Identification code	cu_d8v18673_0m	
Empirical formula	C17 H15 N O4	
Formula weight	297.30	
Temperature	296(2) K	
Wavelength	1.54178 Å	
Crystal system	Monoclinic	
Space group	P 21	
Unit cell dimensions	a = 12.9635(3) Å	α= 90°.
	b = 6.9796(2) Å	β= 98.1510(10)°.
	c = 16.9418(3) Å	γ = 90°.
Volume	1517.41(6) Å^3	
Z	4	
Density (calculated)	1.301 Mg/m^3	
Absorption coefficient	0.772 mm^{-1}	
F(000)	624	
Crystal size	0.180 x 0.140 x 0.100 mm^3	
Theta range for data collection	3.444 to 66.997°.	
Index ranges	-15<=h<=15, -8<=k<=8, -20<=l<=19	
Reflections collected	28014	
Independent reflections	5376 [R(int) = 0.0609]	
Completeness to theta = 67.679°	98.1 %	
Absorption correction	Semi-empirical from equivalents	
Max. and min. transmission	0.7533 and 0.5362	
Refinement method	Full-matrix least-squares on F^2	
Data / restraints / parameters	5376 / 1 / 400	
Goodness-of-fit on F^2	1.035	
Final R indices [I>2sigma(I)]	R1 = 0.0431, wR2 = 0.1105	
R indices (all data)	R1 = 0.0505, wR2 = 0.1198	
Absolute structure parameter	0.08(13)	
Extinction coefficient	0.0177(18)	
Largest diff. peak and hole	0.128 and -0.127 e.Å^{-3}	

Gram-scale experiment.

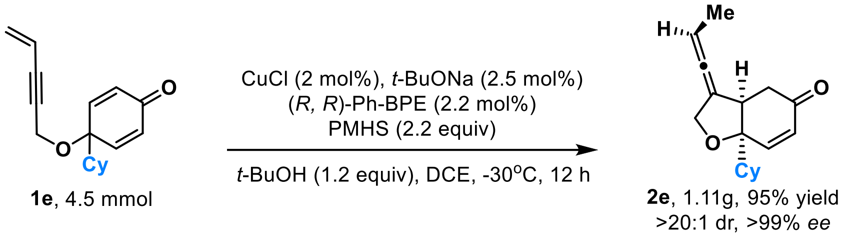

A dried Schlenk flask was charged with CuCl (2 mol%), (*R*,*R*)-Ph-BPE (2.2 mol%), *t*-BuONa (2.5 mol%), backfilled with argon. Then under -30°C, anhydrous DCE (10.0 mL) was added and the solution was stirred for 10min under -30°C. After that, PMHS (2.2 equiv) was added dropwise and the solution was stirred for another 10min under -30°C. Fianlly, a solution of substrate **1e** (4.5 mmol, 1 equiv) and anhydrous *t*-BuOH (1.2 equiv) in DCE (10.0 mL) was added. The resulting reaction mixture was stirred at -30°C for 12h. The reaction mixture was filtered through a short column of silica gel. The diastereomeric ratio of the crude reaction mixture was determined by ^{1}H NMR spectroscopy. The residue was purified by flash silica gel (300-400 mesh) chromatography to afford the desired products **2**e as colorless oil, 95% yield.

Transformation of the cyclization products.

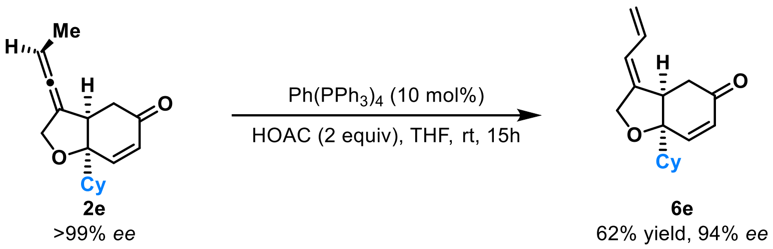

A dried Schlenk flask was charged with tetrakis(triphenylphosphine)palladium (26 mg, 0.01 mmol, 0.1 equiv), backfilled with argon for 3 times, a mixture of **2e** (26 mg, 0.1 mmol) in anhydrous THF (2 ml) was added and then glacial acetic acid (12 μl, 0.2 mmol, 2.0 equiv) was added dropwise. The resulting mixture was stirred at room temperature for 15 h. Upon completion, the reaction mixture was filtered through Celite® and the filtrate was evaporated under reduced pressure and purified by flash column chromatography (PE/EA = 30/1) to afford **6e** (16 mg).

(3a*R*,7a*R*,*E*)-3-allylidene-7a-cyclohexyl-2,3,3a,7a-tetrahydrobenzofuran-5(4*H*)-one (6e) colorless oil, 62% yield, $[\alpha]_D^{25.0}$ 4.9 (*c* 1.00, $CHCl_3$) for 94% *ee*. **^{1}H NMR** (400 MHz, $CDCl_3$) δ (ppm) 6.55 (d, *J* = 10.4 Hz,

1H), 6.15 (dt, J = 16.8, 10.5 Hz, 1H), 6.01 (d, J = 10.4 Hz, 1H), 5.85 (d, J = 10.9 Hz, 1H), 5.10 (dd, J = 26.5, 13.4 Hz, 2H), 4.56 (d, J = 14.0 Hz, 1H), 4.30 (d, J = 14.1 Hz, 1H), 3.13 (s, 1H), 2.75 (d, J = 3.8 Hz, 2H), 1.98 – 1.66 (m, 5H), 1.34 – 1.11 (m, 6H). **^{13}C NMR** (100 MHz, $CDCl_3$) δ (ppm) 197.4, 150.6, 144.2, 132.6, 130.9, 121.1, 117.5, 84.4, 68.0, 45.4, 42.8, 38.4, 28.0, 27.4, 26.7, 26.5, 26.4. **HRMS (EI)**: $[M]^{\oplus}$ calcd for $C_{17}H_{22}O_2^{\oplus}$ 258.1620, found 258.1614. **IR** (KBr) ν (cm^{-1}) 2925, 2853, 1687, 1451, 1417, 1384, 1342, 1261, 1239, 1050, 989, 938, 890, 823, 801, 737. **HPLC**: Chiracel ID-H Column (250 mm); detected at 214 nm; *n*-hexane/*i*-propanol = 95/5; flow = 0.8 ml/min; Retention time: 12.2 min (minor), 13.7 min (major).

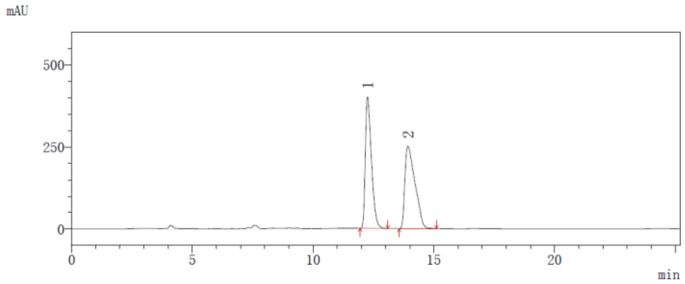

PDA Ch1 214nm

Number	Ret.Time	Area	Area%
1	12.256	7050129	49.05
2	13.925	7324426	50.95

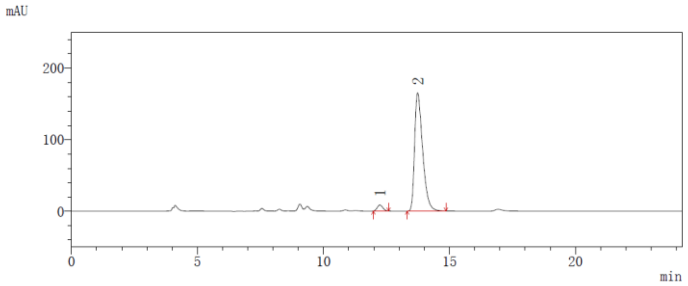

PDA Ch1 214nm

Number	Ret.Time	Area	Area%
1	12.237	128476	3.40
2	13.736	3655445	96.60

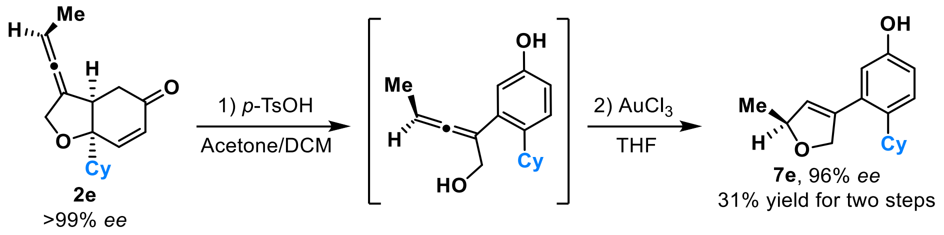

To a solution of **2e** (52 mg, 0.2 mmol) in THF (3.0 mL) and acetone (1 ml), *p*-TsOH • H_2O (190 mg, 1 mmol, 5.0 equiv) was added, and the resulting mixture was stirred at room temperature for 24 h. The reaction mixture was diluted with petroleum ether/ethyl acetate (PE/EA = 2/1), filtered through Celite® and the filtrate was evaporated under reduced pressure to yield the crude product. To a stirred solution of crude in anhydrous THF (2 ml), gold (III) chloride (3 mg, 0.01 mmol, 0.05 equiv) was added and the resulting mixture was stirred at room temperature for 20 mins. Upon completion, the reaction mixture was filtered through Celite® and the filtrate was evaporated under reduced pressure and purified by flash column chromatography (PE/EA = 6/1) to afford **7e** (16 mg).

(*S*)-4-cyclohexyl-3-(5-methyl-2,5-dihydrofuran-3-yl)phenol (7e) colorless oil, 31% yield for 2 steps. $[\alpha]_D^{24.9}$ 17.0 (c 0.50, $CHCl_3$) for 96% *ee*. **^{1}H NMR** (400 MHz, $CDCl_3$) δ (ppm) 7.16 (d, J = 8.5 Hz, 1H), 6.75 (dd, J = 8.5, 2.7 Hz, 1H), 6.61 (d, J = 2.7 Hz, 1H), 5.68 (d, J = 1.6 Hz, 1H), 5.20 – 5.10 (m, 1H), 4.96 (s, 1H), 4.88 – 4.76 (m, 2H), 2.71 – 2.60 (m, 1H), 1.77 (dd, J = 38.2, 11.0 Hz, 5H), 1.38 (d, J = 6.3 Hz, 4H), 1.33 – 1.22 (m, 4H). **^{13}C NMR** (100 MHz, $CDCl_3$) δ (ppm) 153.2, 138.9, 138.5, 134.0, 129.1, 127.7, 115.2,

115.1, 83.5, 78.0, 40.1, 35.2, 35.0, 27.2, 27.1, 26.3, 22.1. **HRMS (ESI)**: [M+H]$^{\oplus}$ calcd for $C_{17}H_{23}O_2^{\oplus}$ 259.1693, found 259.1694. **IR** (KBr) *v* (cm^{-1}) 3323, 2924, 2851, 1606, 1575, 1499, 1448, 1294, 1261, 1195, 1105, 1056, 1019, 863, 808. **HPLC**: Chiracel ID-H Column (250 mm); detected at 214 nm; *n*-hexane/*i*-propanol = 95/5; flow = 0.8 ml/min; Retention time: 10.6 min (major), 11.8 min (minor).

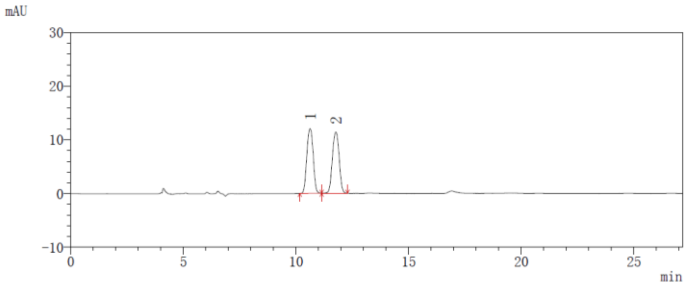

PDA Ch1 254nm

Number	Ret. Time	Area	Area%
1	10.627	237436	49.88
2	11.770	238614	50.12

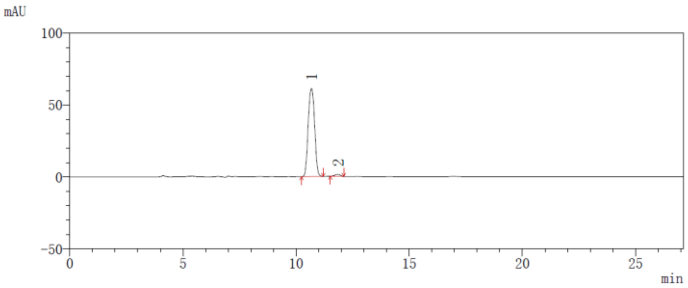

PDA Ch1 254nm

Number	Ret. Time	Area	Area%
1	10.682	1208893	98.12
2	11.846	23143	1.88

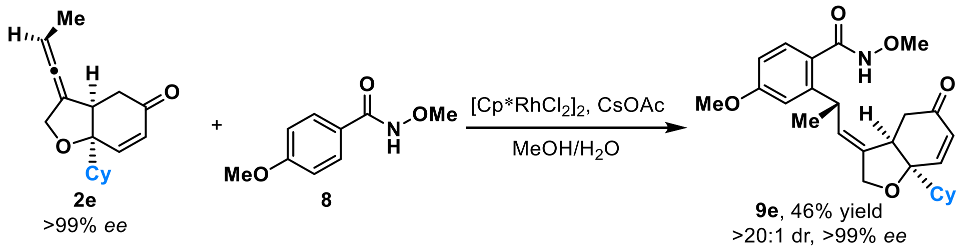

A dried Schlenk flask was charged with *N*,4-dimethoxybenzamide **8** (1.5 equiv), [Cp*RhCl$_2$]$_2$ (5 mol%), CsOAc (1.0 equiv) backfilled with argon for 3 times, a mixture of **2e** (26 mg, 0.1 mmol) in anhydrous MeOH/H_2O (1.05 mL) was added. The resulting mixture was stirred at -10°C for 14 h. Upon completion, the reaction mixture was filtered through Celite® and the filtrate was evaporated under reduced pressure and purified by flash column chromatography (PE/EA = 1/1) to afford **9e** (20.3 mg).

2-((*S*)-1-((3a*R*,7a*R*,*E*)-7a-cyclohexyl-5-oxo-3a,4,5,7a-tetrahydrobenzofuran-3(2*H*)-ylidene)propan-2-yl)-*N*,4-dimethoxybenzamide (9e) yellow solid, mp 56-57°C, 46% yield. $[\alpha]_D^{25.0}$ 124.7 (*c* 0.78, $CHCl_3$) for >99% *ee*. **^{1}H NMR** (400 MHz, $CDCl_3$) δ (ppm) 8.45 (s, 1H), 7.27 (d, *J* = 8.4 Hz, 1H), 6.89 (d, *J* = 2.5 Hz, 1H), 6.71 (dd, *J* = 8.5, 2.5 Hz, 1H), 6.54 (d, *J* = 10.4 Hz, 1H), 6.10 (d, *J* = 10.4 Hz, 1H), 5.35 (dd, *J* = 9.7, 1.9 Hz, 1H), 4.31 – 4.22 (m, 1H), 4.21 – 4.11 (m, 2H), 3.92 – 3.79 (m, 6H), 3.41 (s, 1H), 2.80 – 2.67 (m, 2H), 1.89 – 1.73 (m, 4H), 1.69 – 1.57 (m, 2H), 1.34 (d, *J* = 7.0 Hz, 3H), 1.21 – 0.95 (m, 5H). **^{13}C NMR** (125 MHz, $CDCl_3$) δ (ppm) 198.7, 168.1, 161.7, 148.7, 147.3, 140.7, 130.9, 129.1, 126.6, 124.7, 113.5, 110.5, 84.6, 70.0, 64.7, 55.4, 45.3, 41.4, 40.2, 34.4, 27.3, 27.3, 26.5, 26.4, 22.3. **HRMS (ESI)**: [M+H]$^{\oplus}$ calcd for $C_{26}H_{34}NO_5^{\oplus}$ 440.2431, found 440.2431. **IR** (KBr) *v* (cm^{-1}) 3231, 2926, 2852, 1681, 1605, 1455, 1276, 1234, 1022, 827, 758. **HPLC**: Chiracel ID-H Column (250 mm); detected at 214 nm; *n*-hexane/*i*-propanol = 80/20; flow = 1.0 ml/min; Retention time: 7.5 min (major).

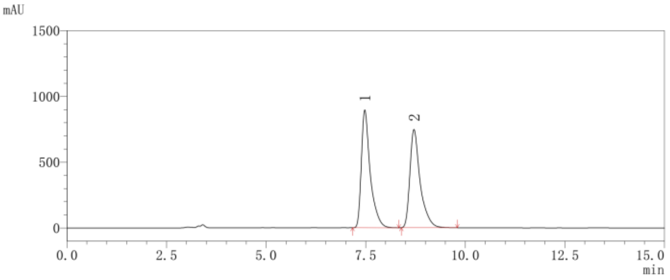

PDA Ch1 214nm

Number	Ret. Time	Area	Area%
1	7.477	13904053	50.92
2	8.713	13400062	49.08

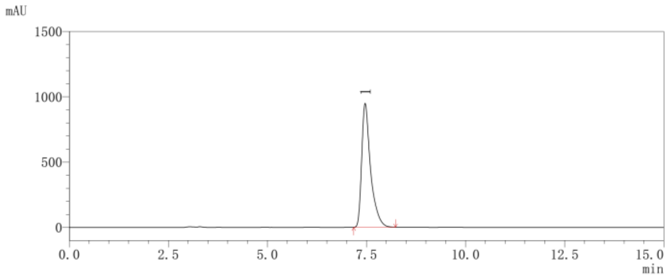

PDA Ch1 214nm

Number	Ret. Time	Area	Area%
1	7.466	14811343	100.00

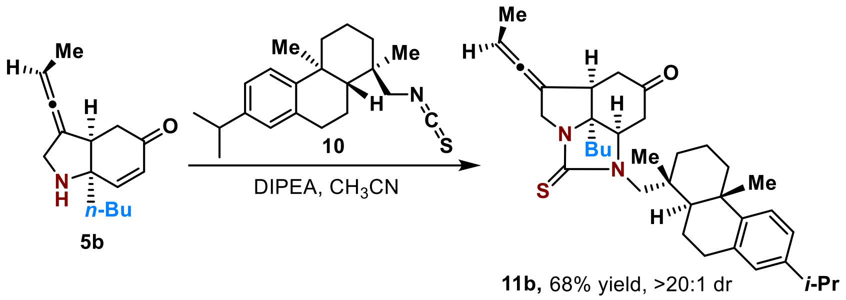

To a stirred solution of **5b** (22 mg, 0.095 mmol), DIPEA (24 ul, 0.143 mmol, 1.5 equiv) in acetonitrile (4 ml), dehydroabietyl **10** (47 mg, 0.143 mmol, 1.5 equiv) was added and the resulting mixture was stirred at 80 °C for 20 h. Upon completion, the reaction mixture was concentrated in vacuo. The residue was purified by flash column chromatography (PE/EA = 15/1) to afford **11b** (36 mg).

(2a^1*S*,5a*R*)-2a^1-butyl-2-(((1*S*,4a*R*,10a*S*)-7-isopropyl-1,4a-dimethyl-1,2,3,4,4a,9,10,10a-octahydrophenanthren-1-yl)methyl)-6-((*S*)-prop-1-en-1-ylidene)-1-thioxooctahydroimidazo[4,5,1-hi]indol-4(2a^1*H*)-one (11b) colorless oil, 68% yield. $[\alpha]_D^{24.9}$ -22.2 (*c* 2.00, $CHCl_3$). **^{1}H NMR** (400 MHz, $CDCl_3$) δ (ppm) 7.13 (d, *J* = 8.2 Hz, 1H), 6.95 (d, *J* = 8.1 Hz, 1H), 6.87 (s, 1H), 5.41 – 5.28 (m, 1H), 5.12 (dd, *J* = 15.3, 3.9 Hz, 1H), 4.51 – 4.38 (m, 2H), 3.86 (dd, *J* = 15.3, 3.5 Hz, 1H), 3.00 (dd, *J* = 17.9, 2.5 Hz, 1H), 2.91 – 2.68 (m, 5H), 2.41 – 2.32 (m, 2H), 2.28 (d, *J* = 11.8 Hz, 1H), 2.08 – 1.88 (m, 2H), 1.77 – 1.68 (m, 4H), 1.65 (d, *J* = 7.0 Hz, 4H), 1.58 (t, *J* = 5.3 Hz, 2H), 1.44 – 1.37 (m, 2H), 1.33 – 1.24 (m, 4H), 1.22 – 1.17 (m, 9H), 1.07 (s, 3H), 0.77 (t, *J* = 6.9 Hz, 3H). **^{13}C NMR** (100 MHz, $CDCl_3$) δ (ppm) 206.8, 197.6, 188.1, 147.1, 145.7, 134.8, 127.0, 124.1, 123.8, 100.5, 91.6, 71.1, 59.9, 55.4, 49.1, 48.1, 46.7, 41.9, 39.8, 38.4, 38.1, 37.8, 36.2, 36.0, 33.6, 30.3, 26.3, 25.5, 24.1, 22.8, 21.1, 19.9, 18.8, 14.8, 14.0. **HRMS (ESI)**: $[M+H]^{\oplus}$ calcd for $C_{36}H_{51}N_2OS^{\oplus}$ 559.3717, found 559.3719. **IR** (KBr) *ν* (cm^{-1}) 2957, 2928, 2869, 1722, 1497, 1455, 1427, 1409, 1380, 1311, 1258, 1204, 1081, 978, 821, 757.

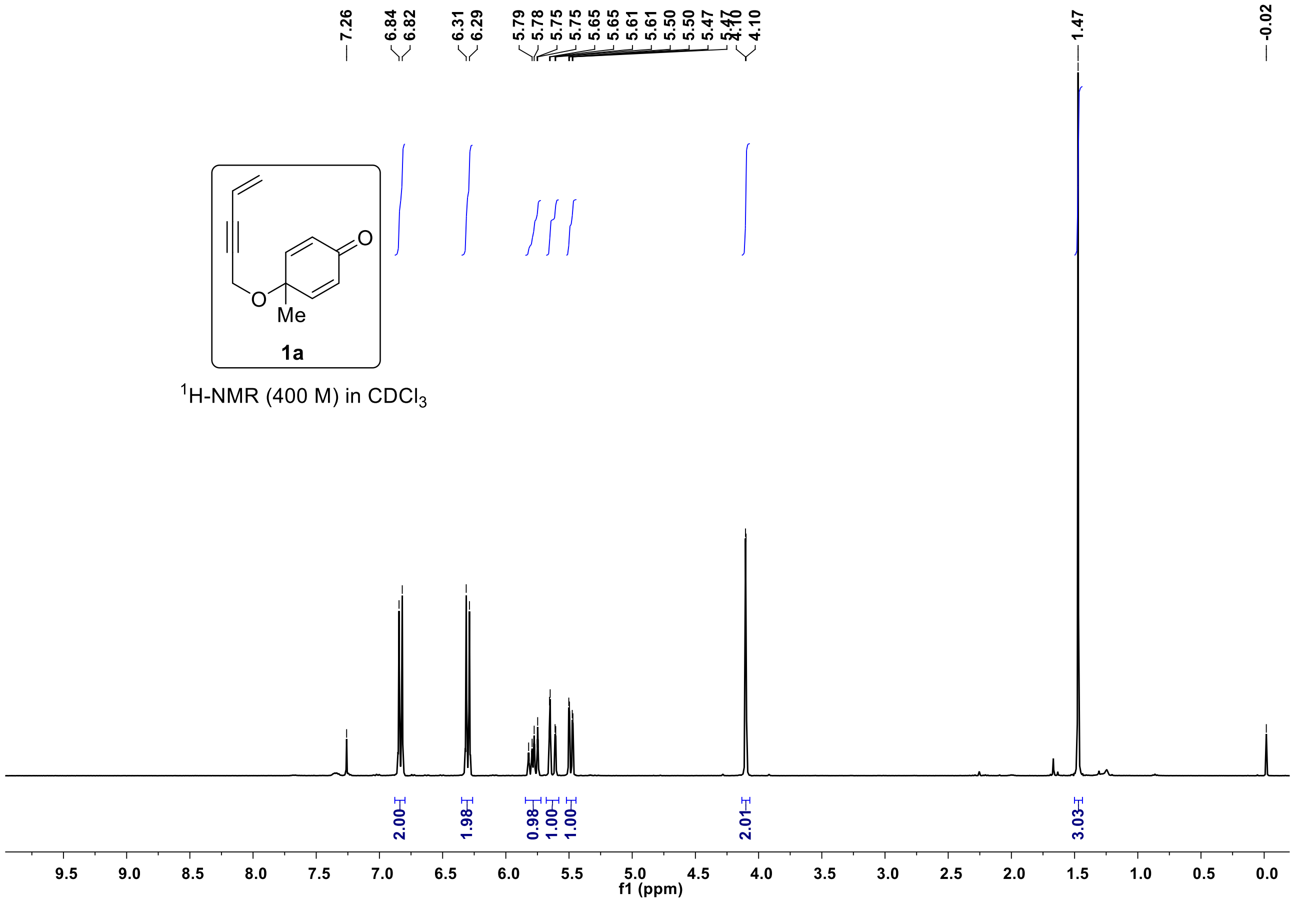

Supplementary Figure 2. ^{1}H NMR spectra for **1a**

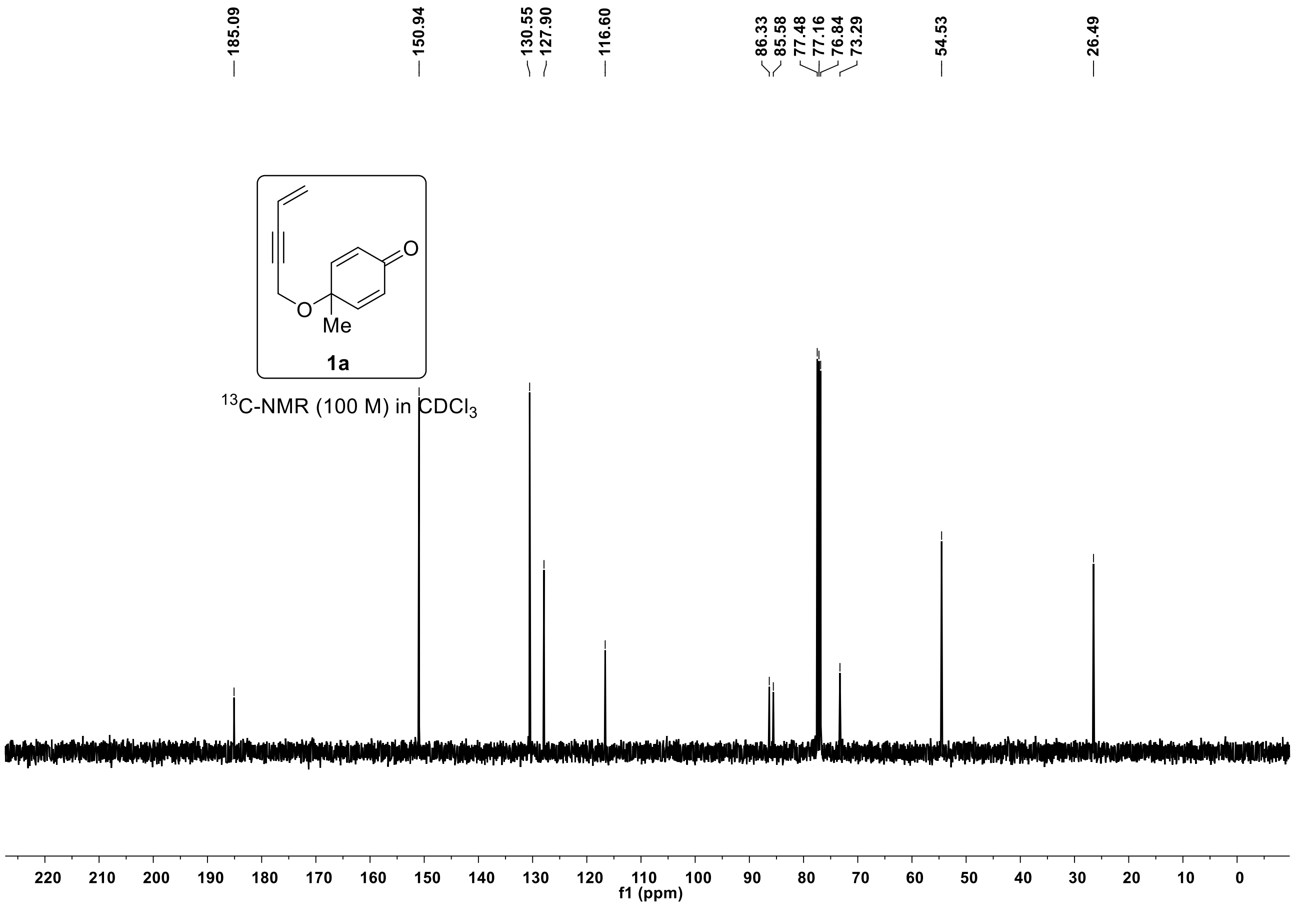

Supplementary Figure 3. ^{13}C NMR spectra for **1a**

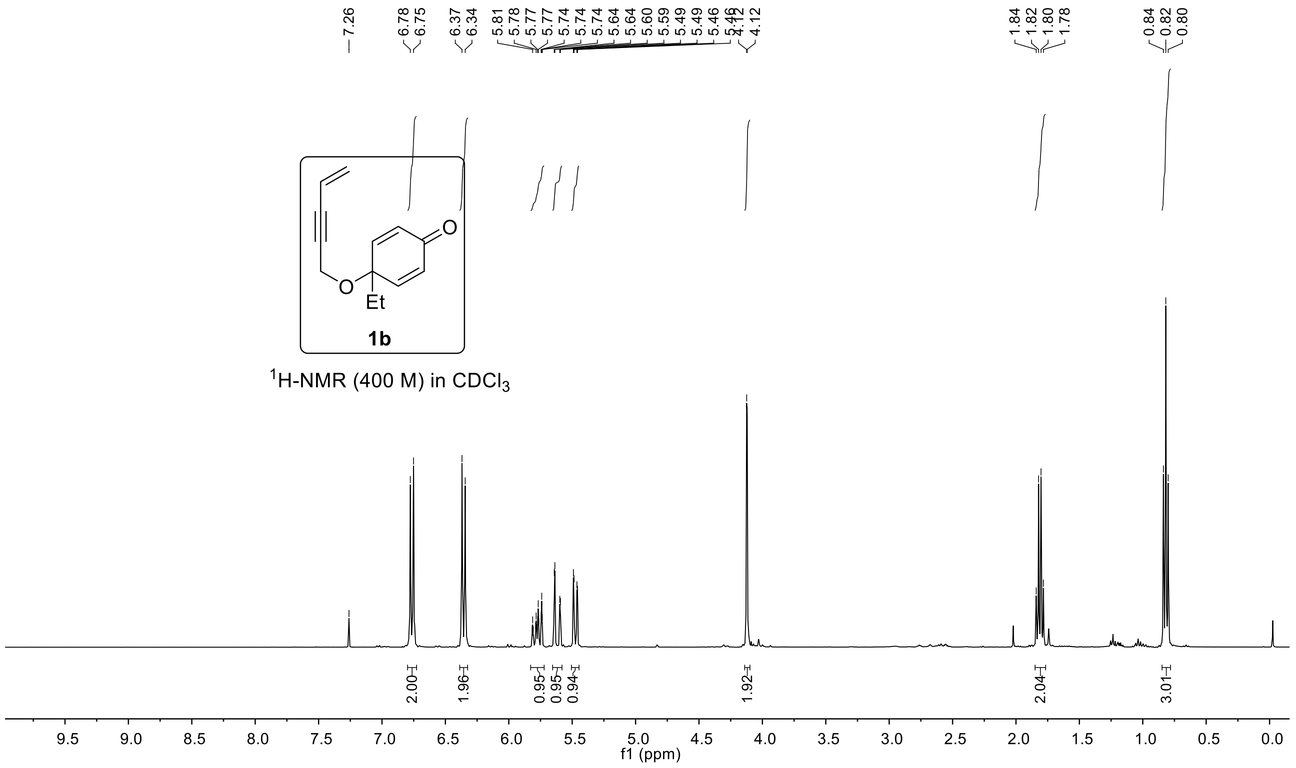

Supplementary Figure 4. ^{1}H NMR spectra for **1b**

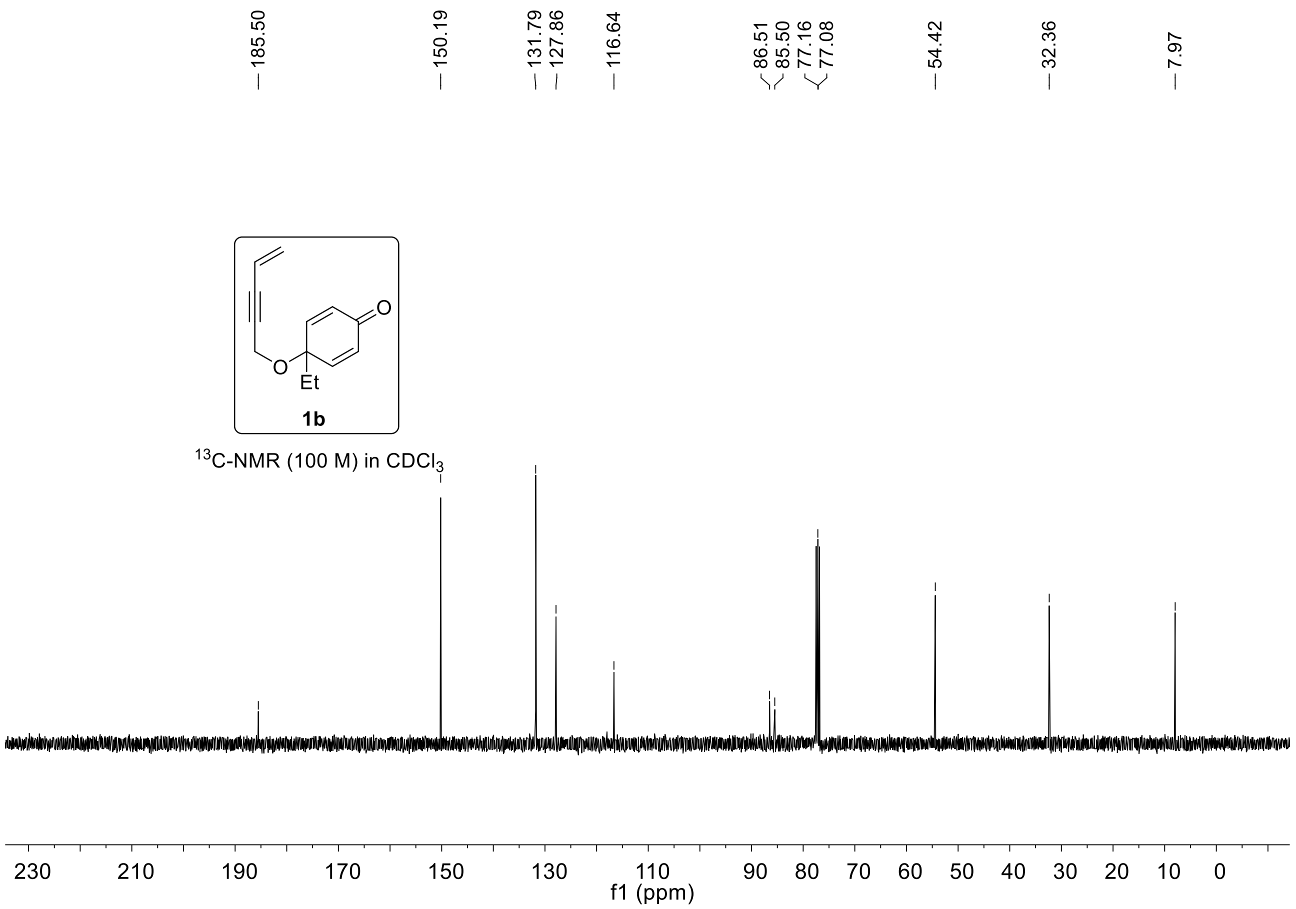

Supplementary Figure 5. ^{13}C NMR spectra for **1b**

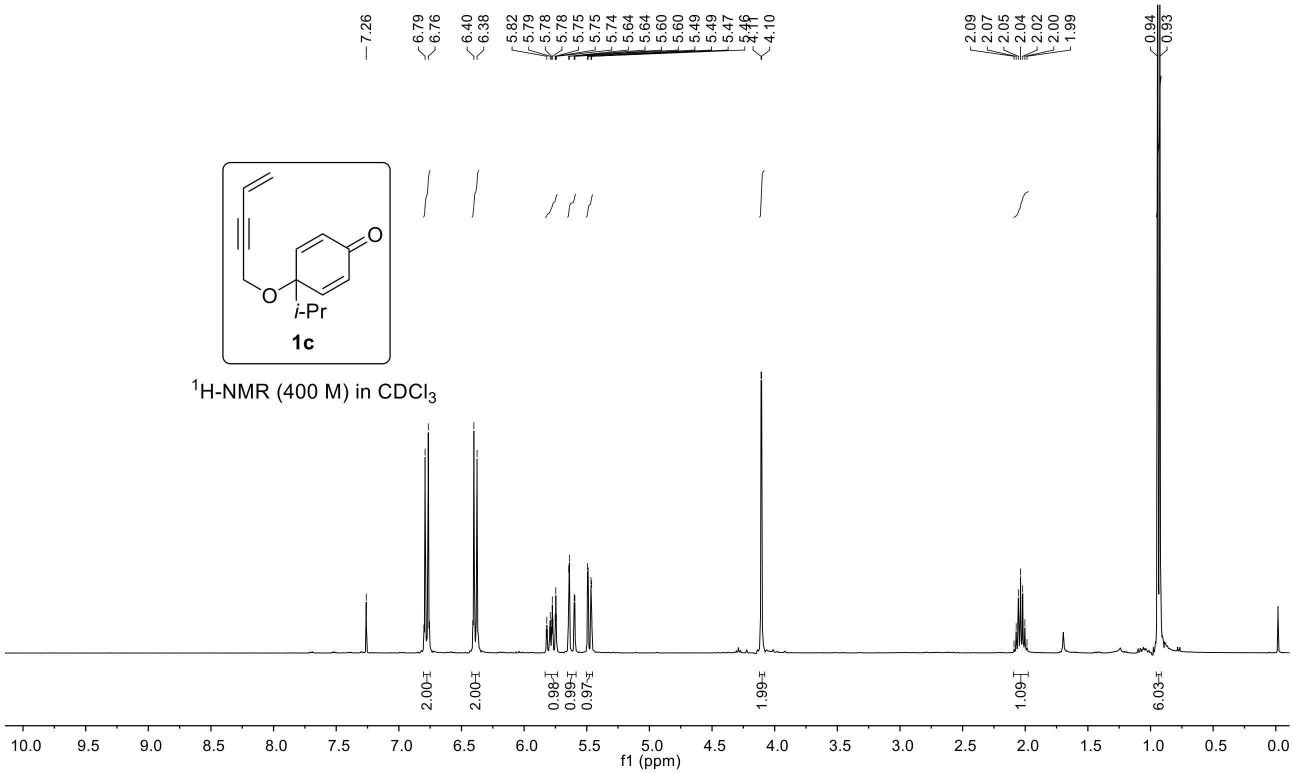

Supplementary Figure 6. ^{1}H NMR spectra for **1c**

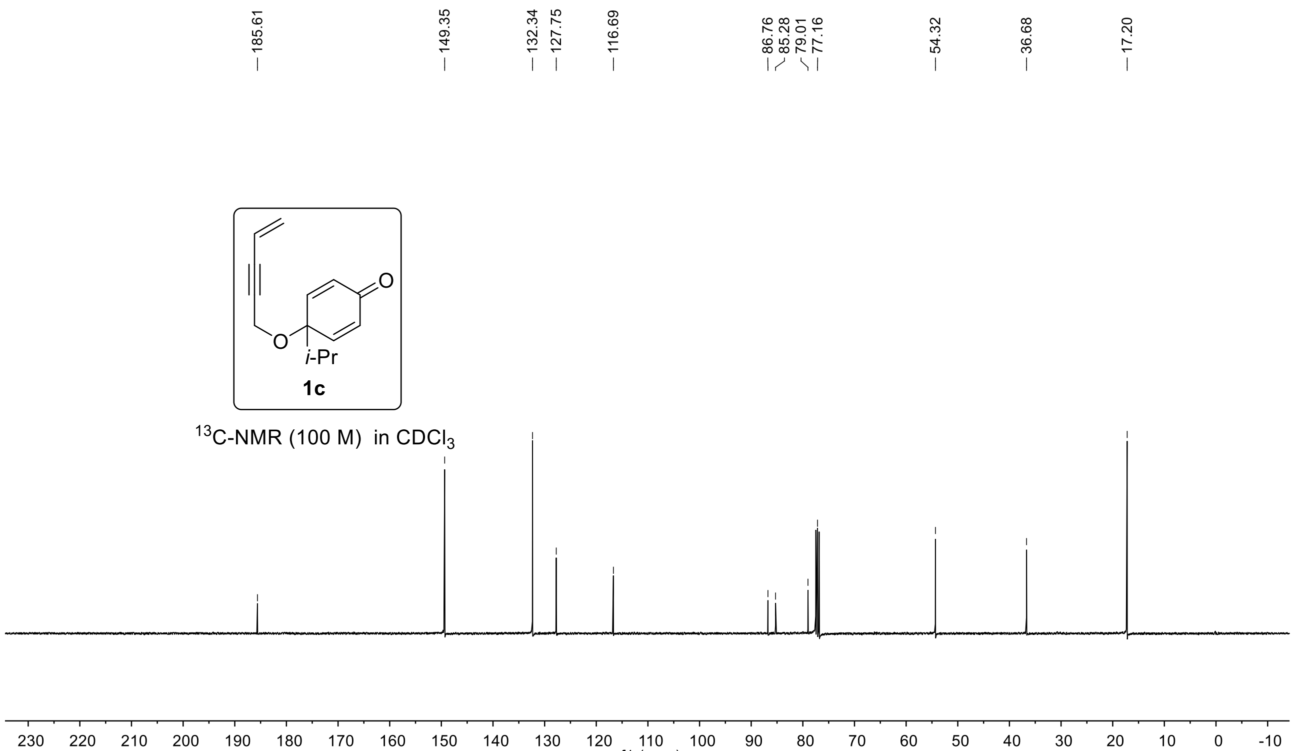

Supplementary Figure 7. ^{13}C NMR spectra for **1c**

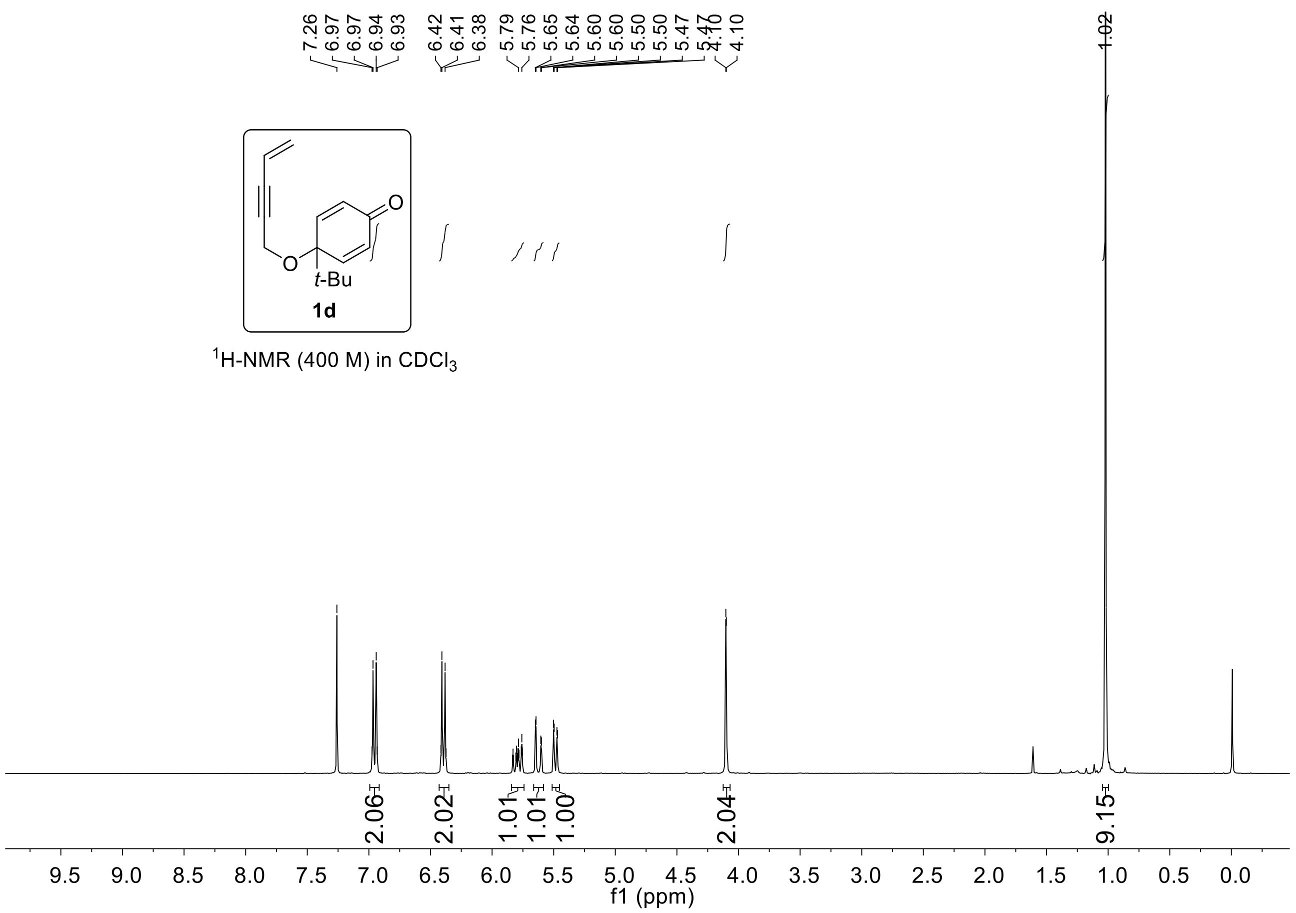

Supplementary Figure 8. ^{1}H NMR spectra for **1d**

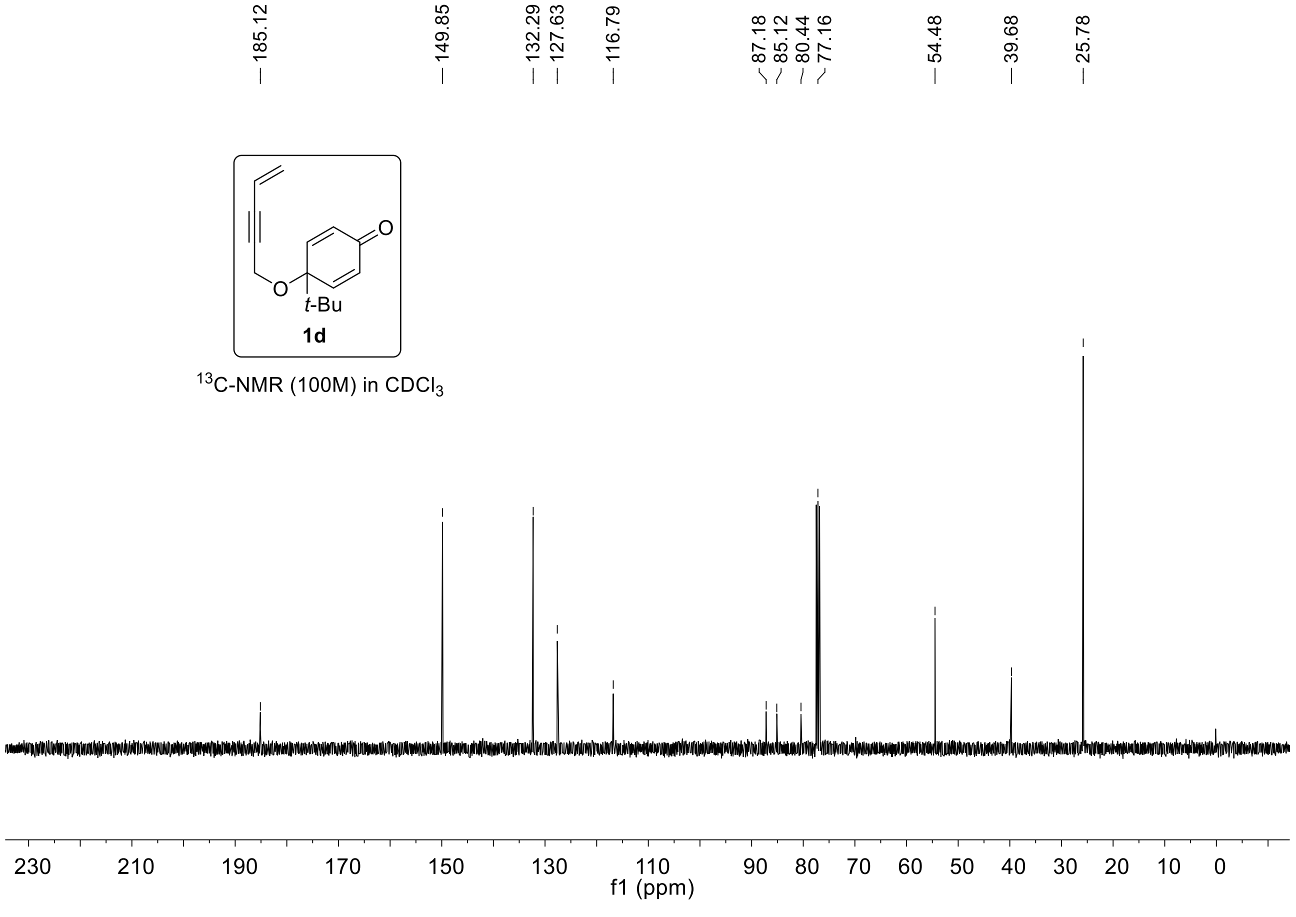

Supplementary Figure 9. ^{13}C NMR spectra for **1d**

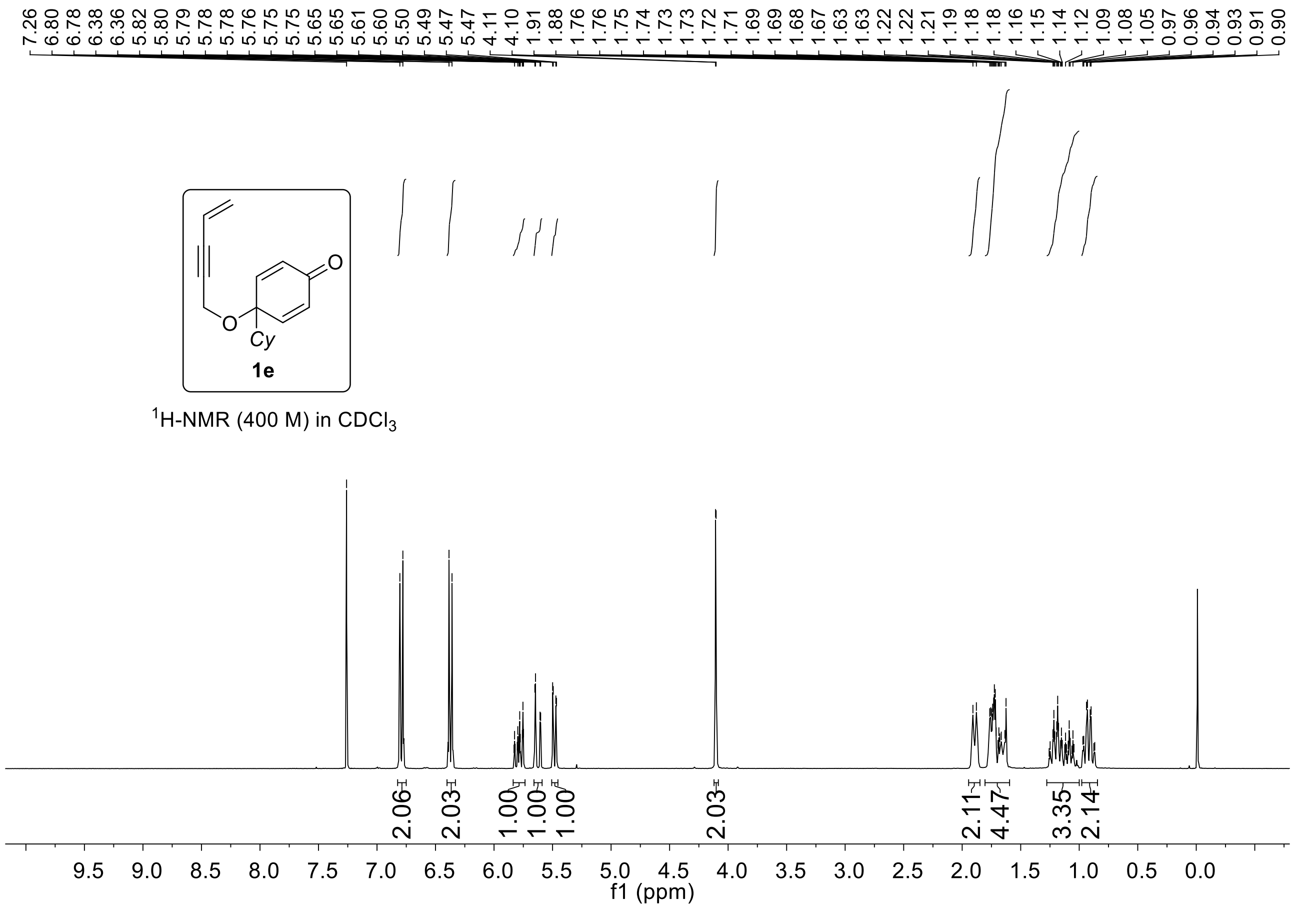

Supplementary Figure 10. ^{1}H NMR spectra for **1e**

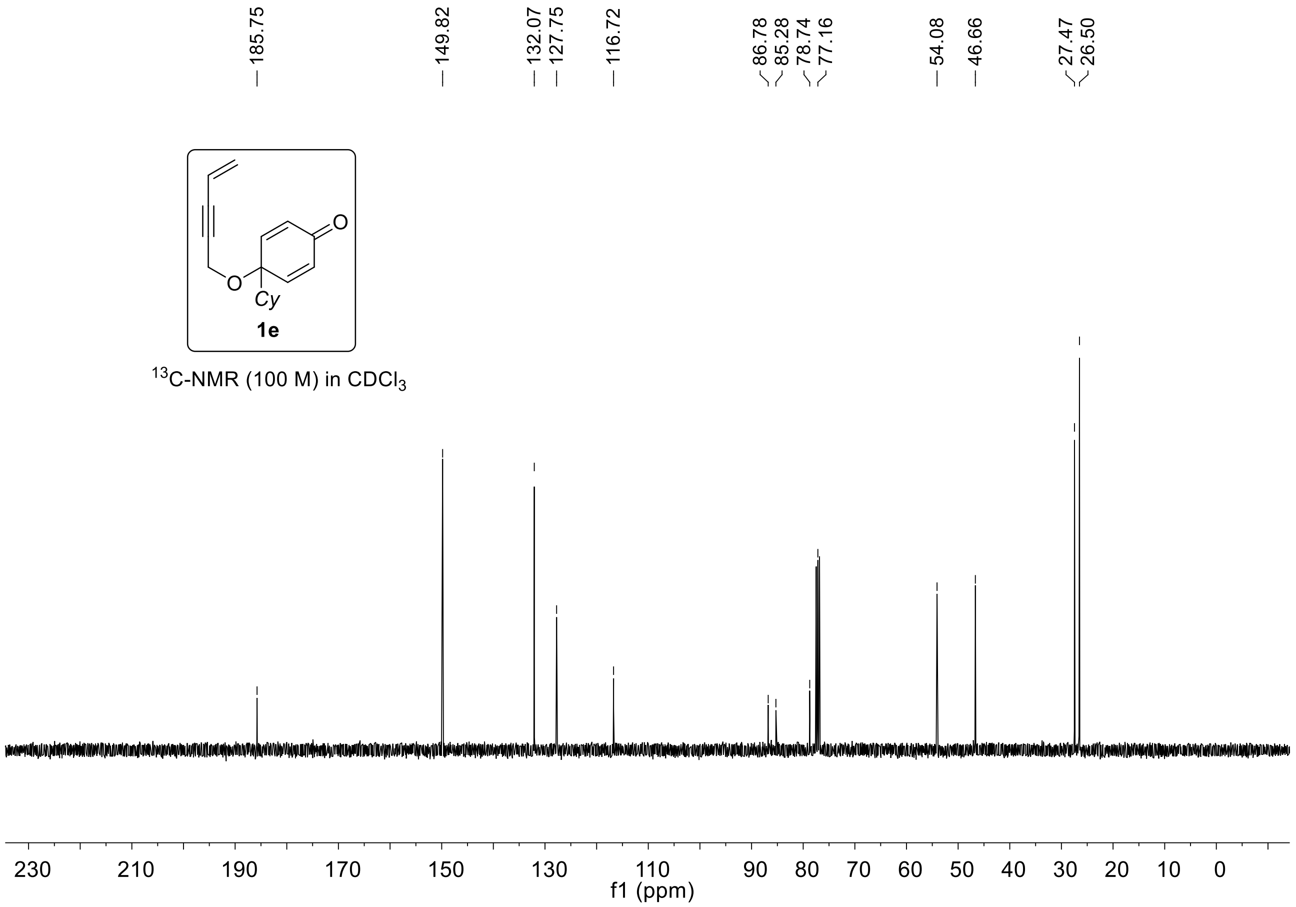

Supplementary Figure 11. ^{13}C NMR spectra for **1e**

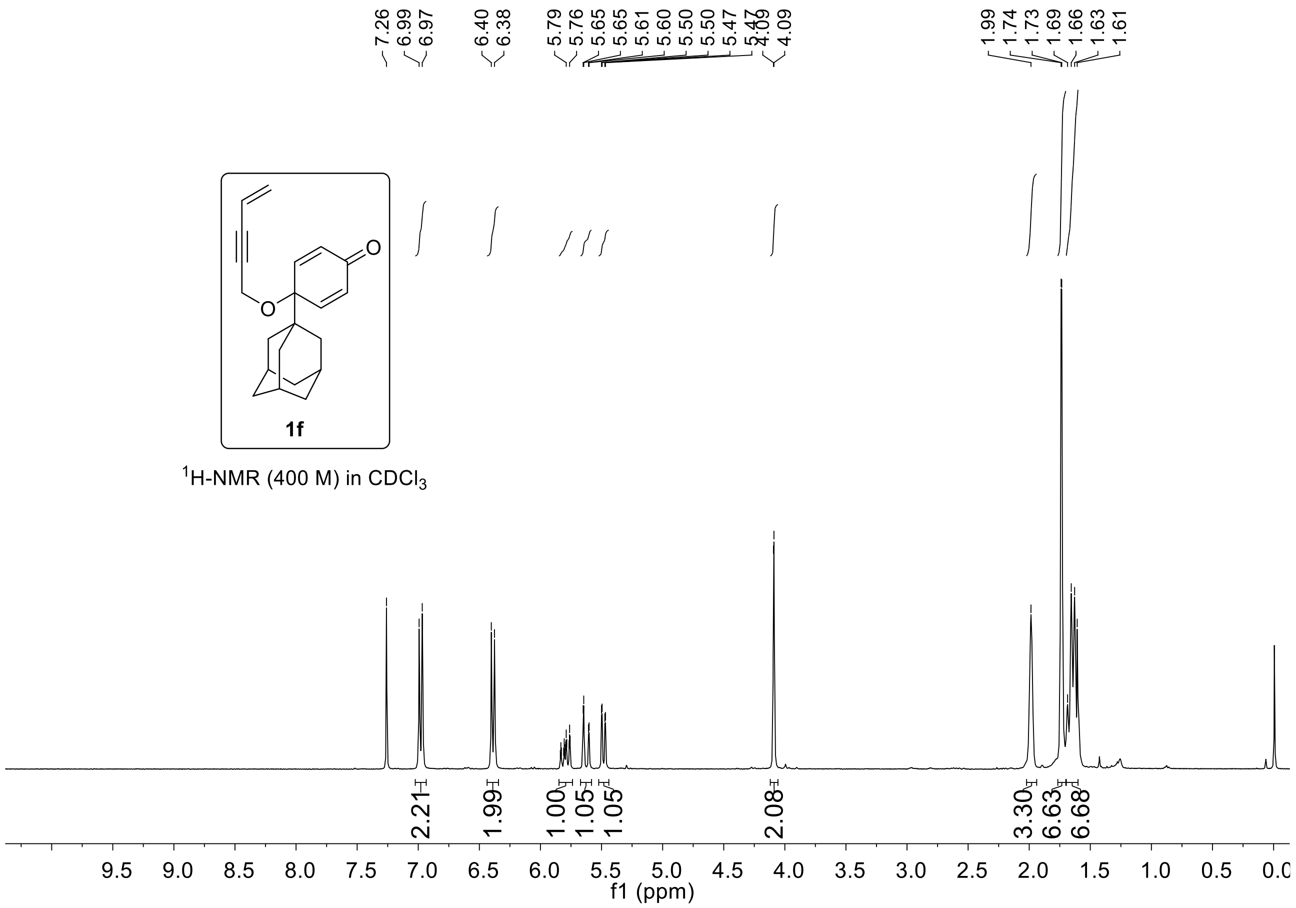

Supplementary Figure 12. ^{1}H NMR spectra for **1f**

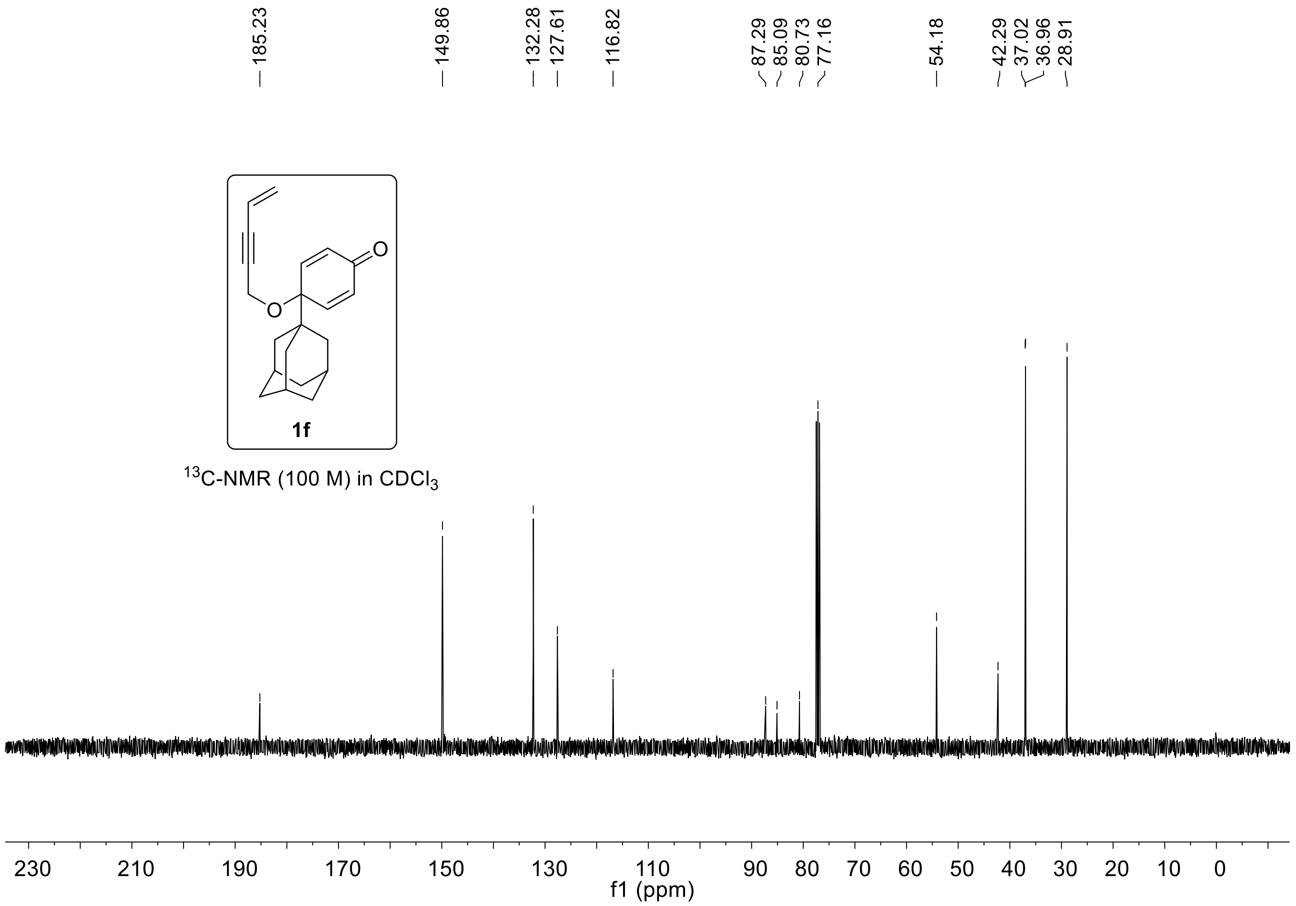

Supplementary Figure 13. ^{13}C NMR spectra for **1f**

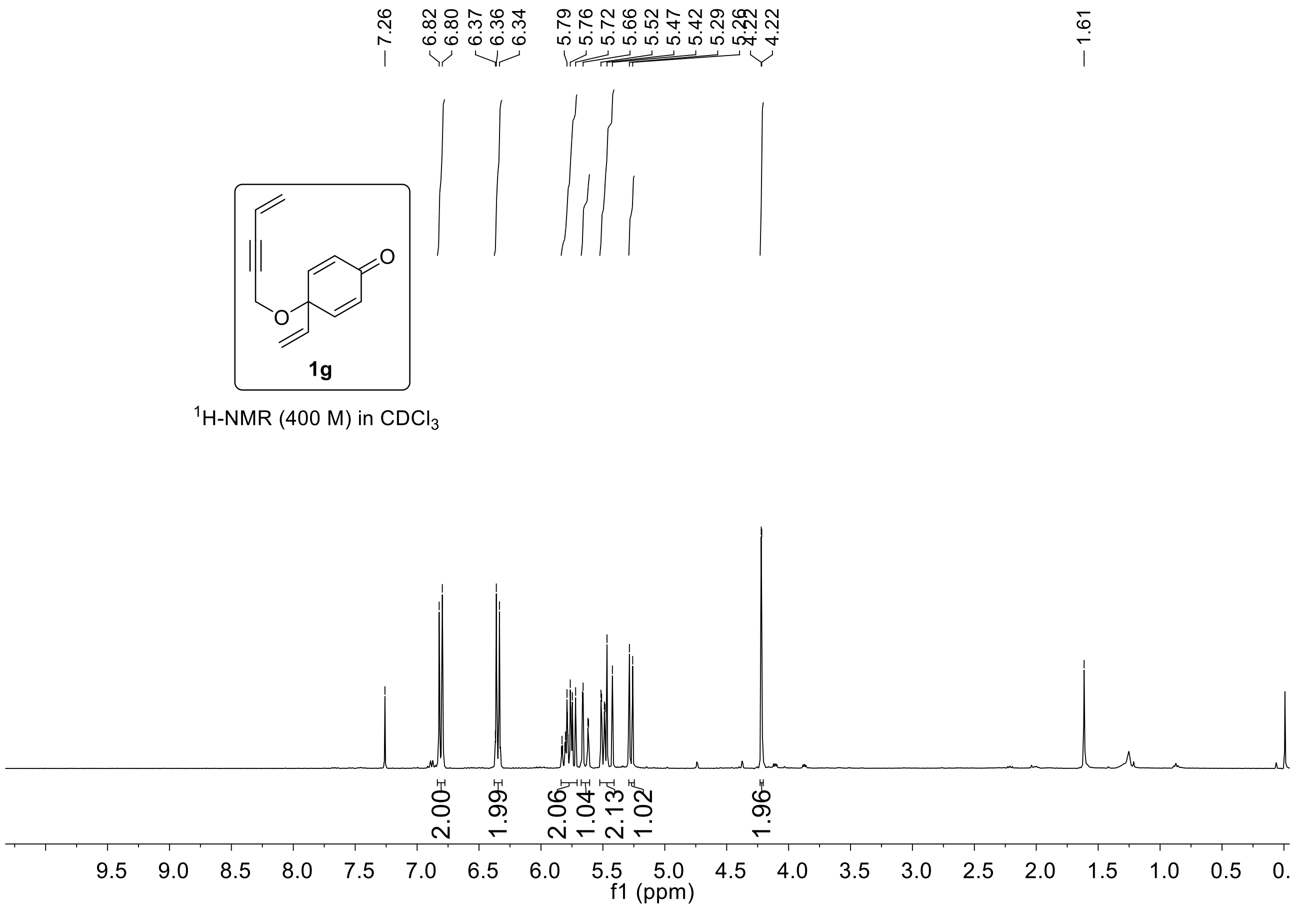

Supplementary Figure 14. ^{1}H NMR spectra for **1g**

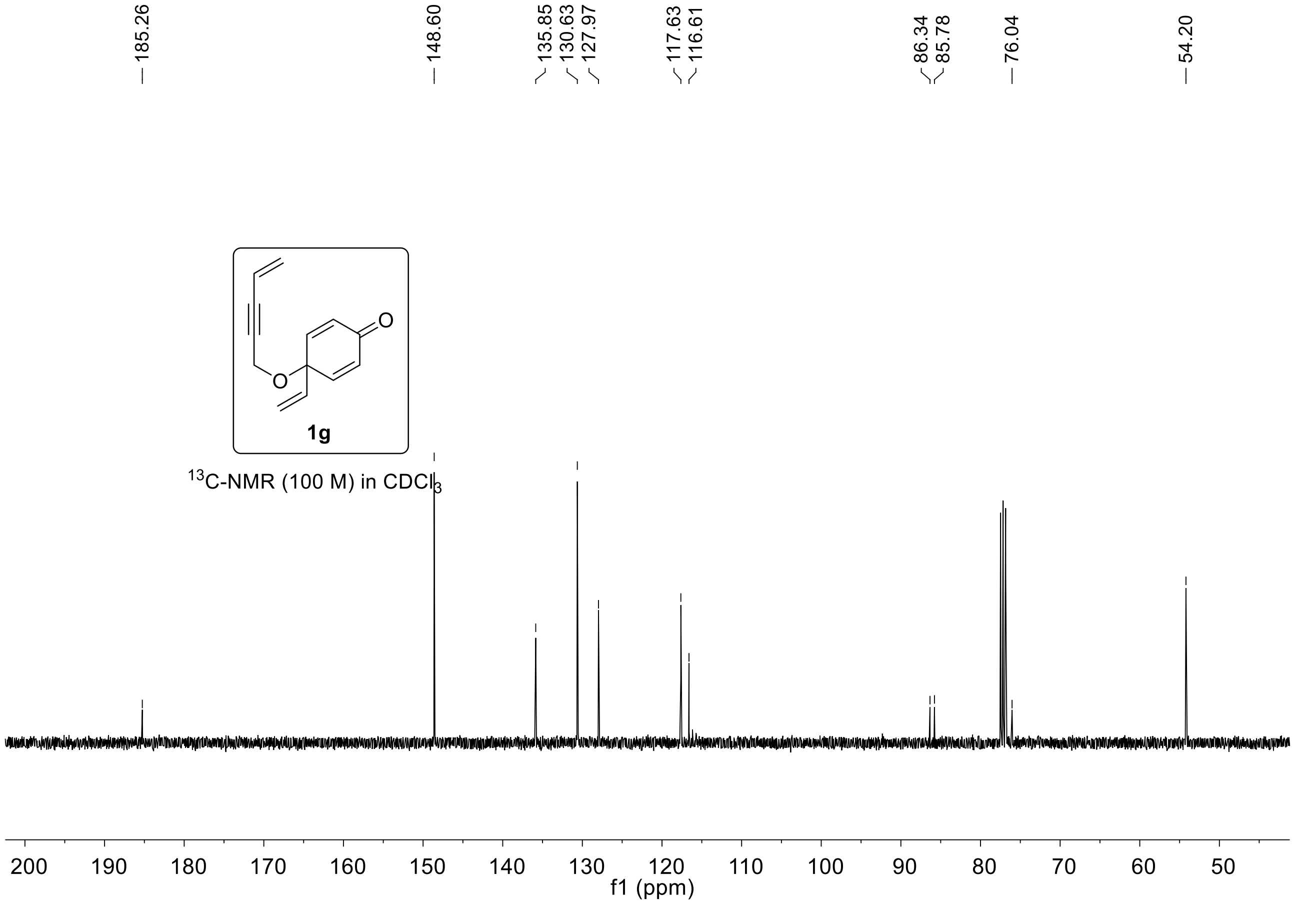

Supplementary Figure 15. ^{13}C NMR spectra for **1g**

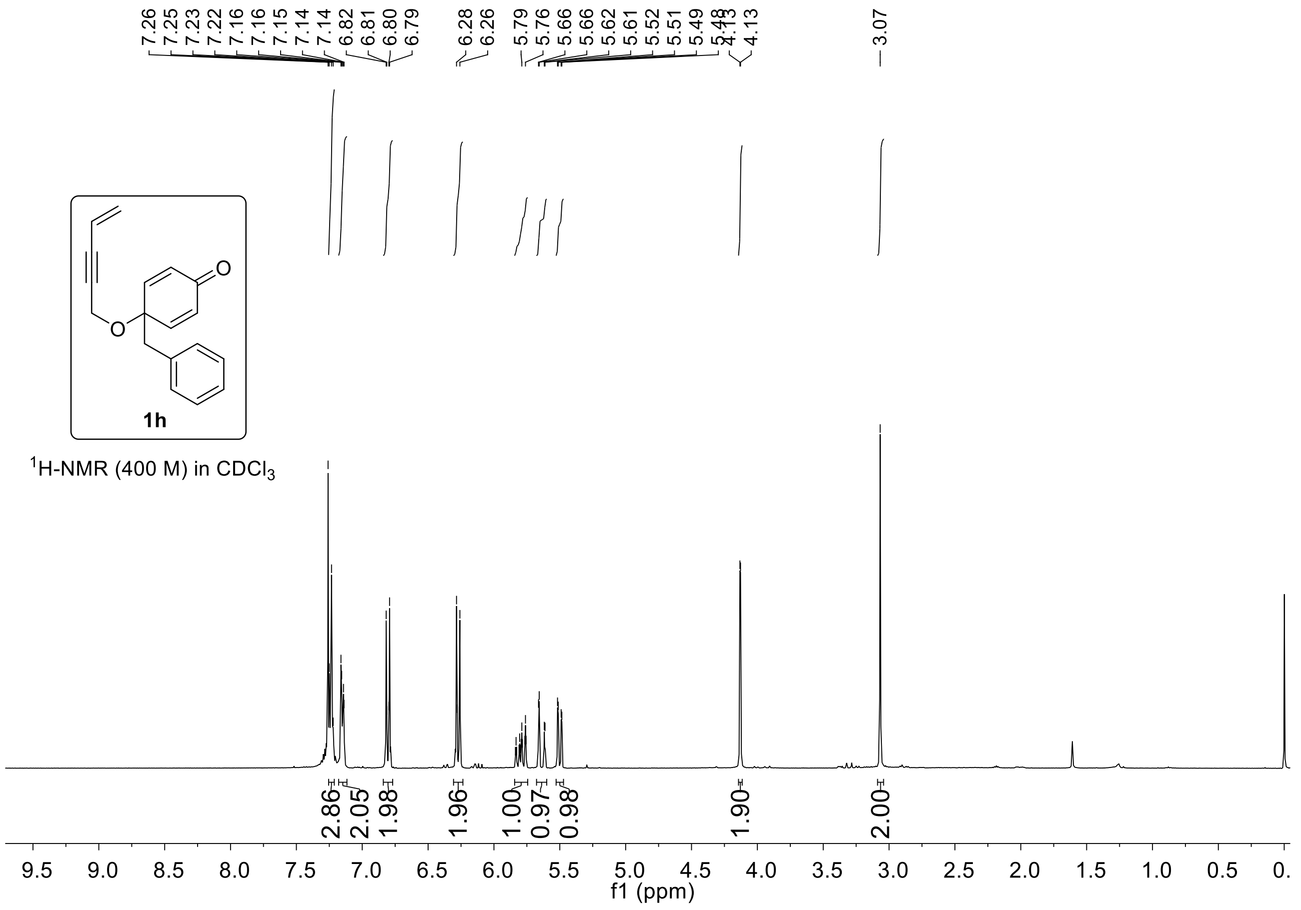

Supplementary Figure 16. ^{1}H NMR spectra for **1h**

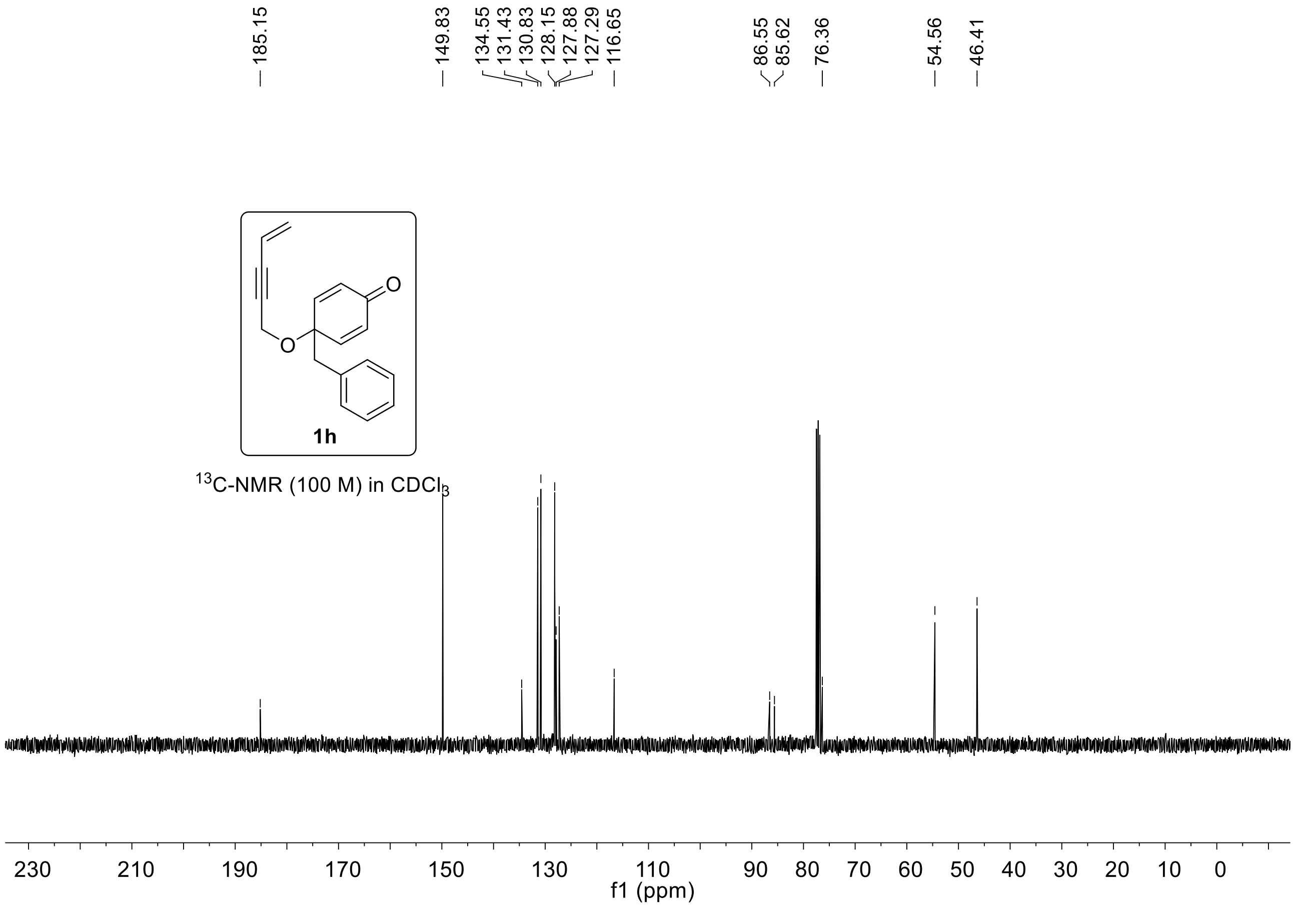

Supplementary Figure 17. ^{13}C NMR spectra for **1h**

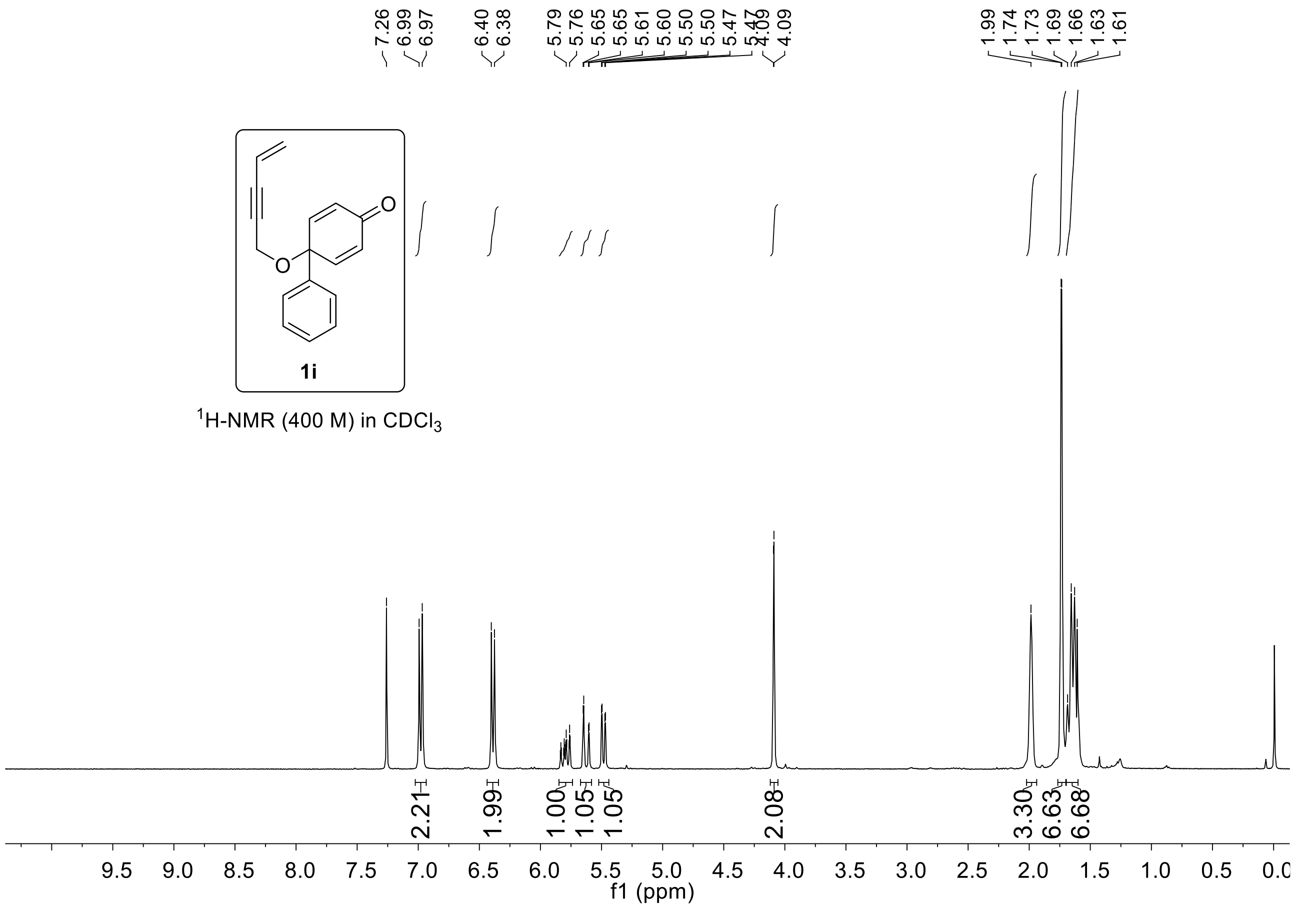

Supplementary Figure 18. ^{1}H NMR spectra for **1i**

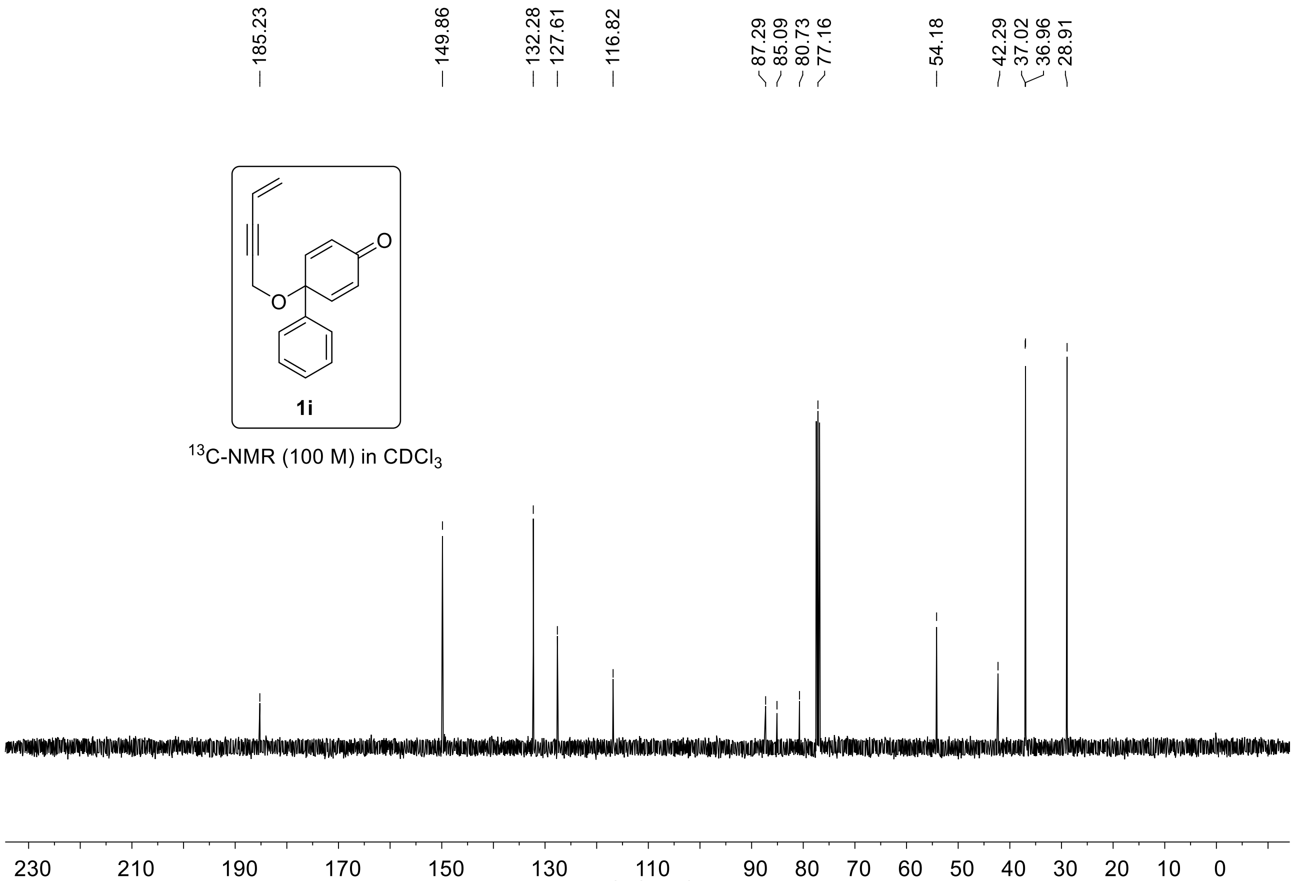

Supplementary Figure 19. ^{13}C NMR spectra for **1i**

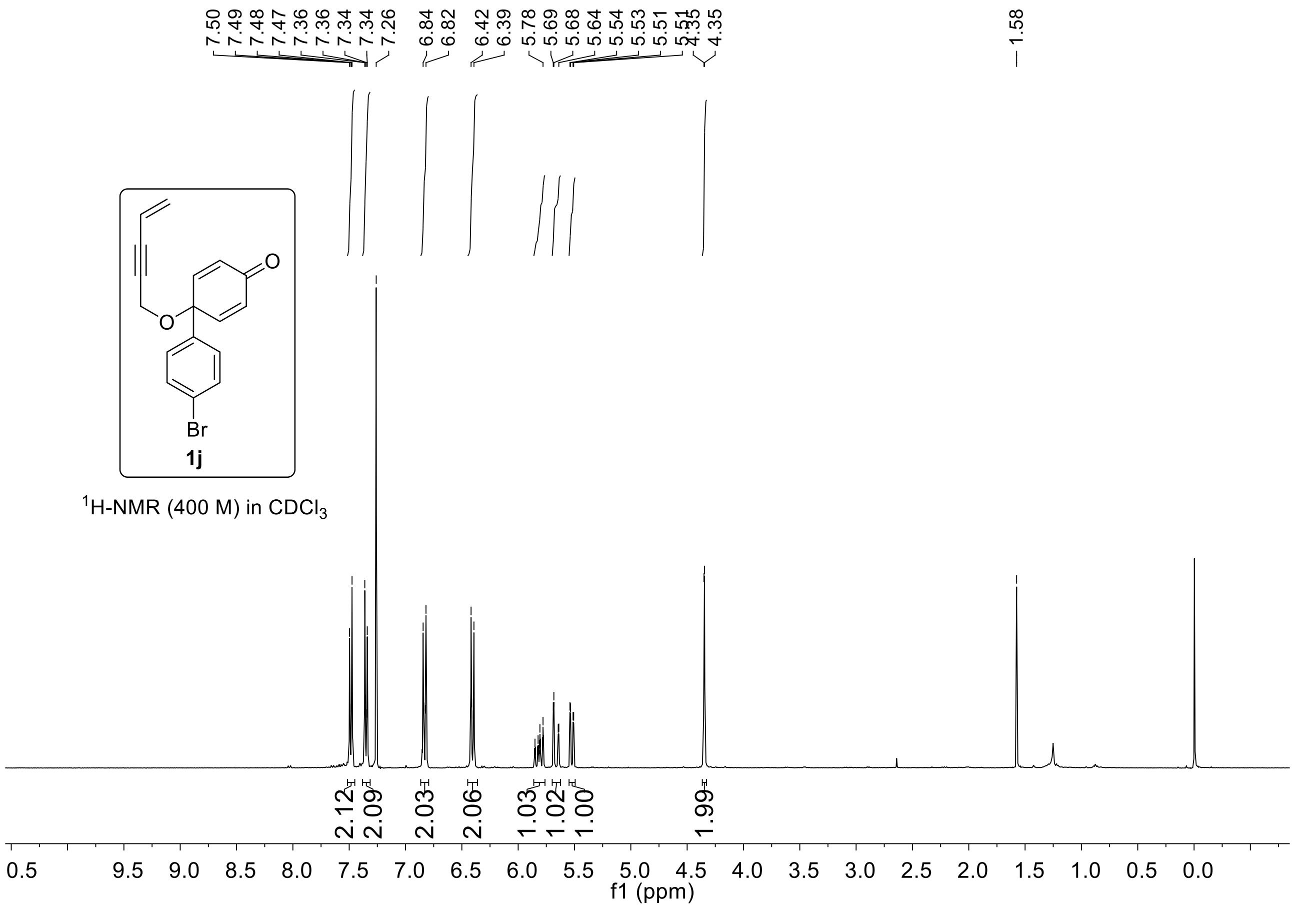

Supplementary Figure 20. ^{1}H NMR spectra for **1j**

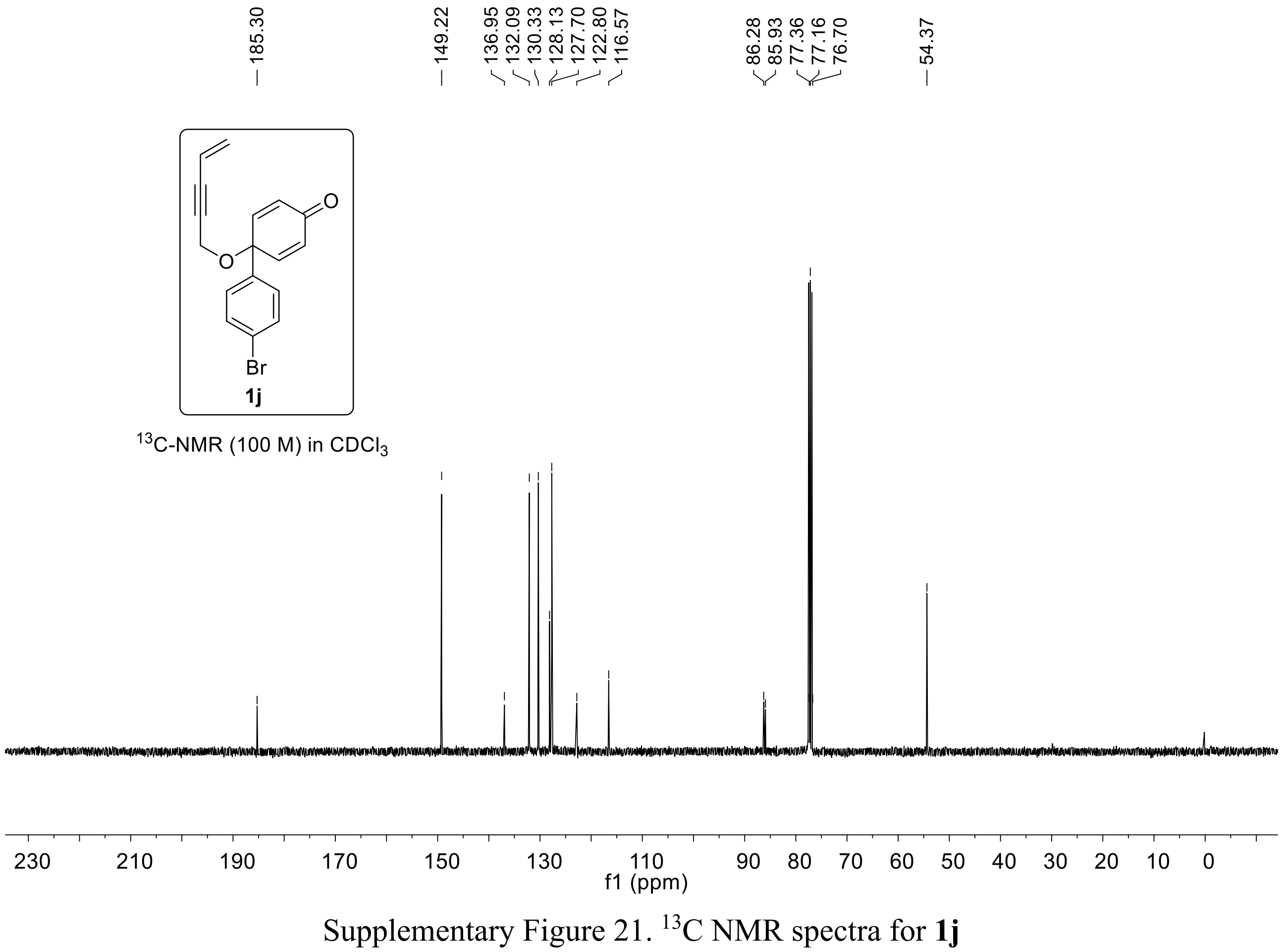

Supplementary Figure 21. ^{13}C NMR spectra for **1j**

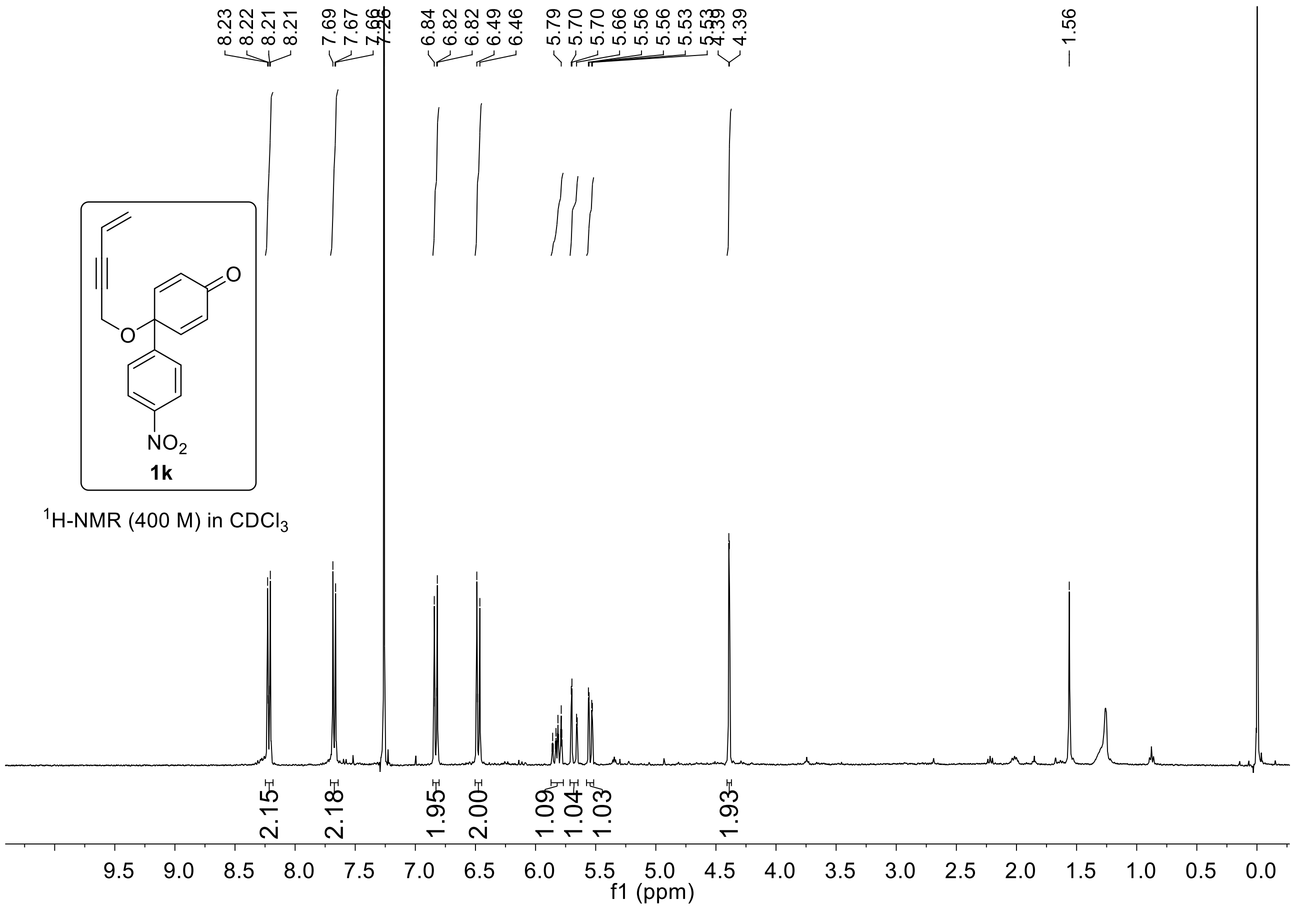

Supplementary Figure 22. ^{1}H NMR spectra for **1k**

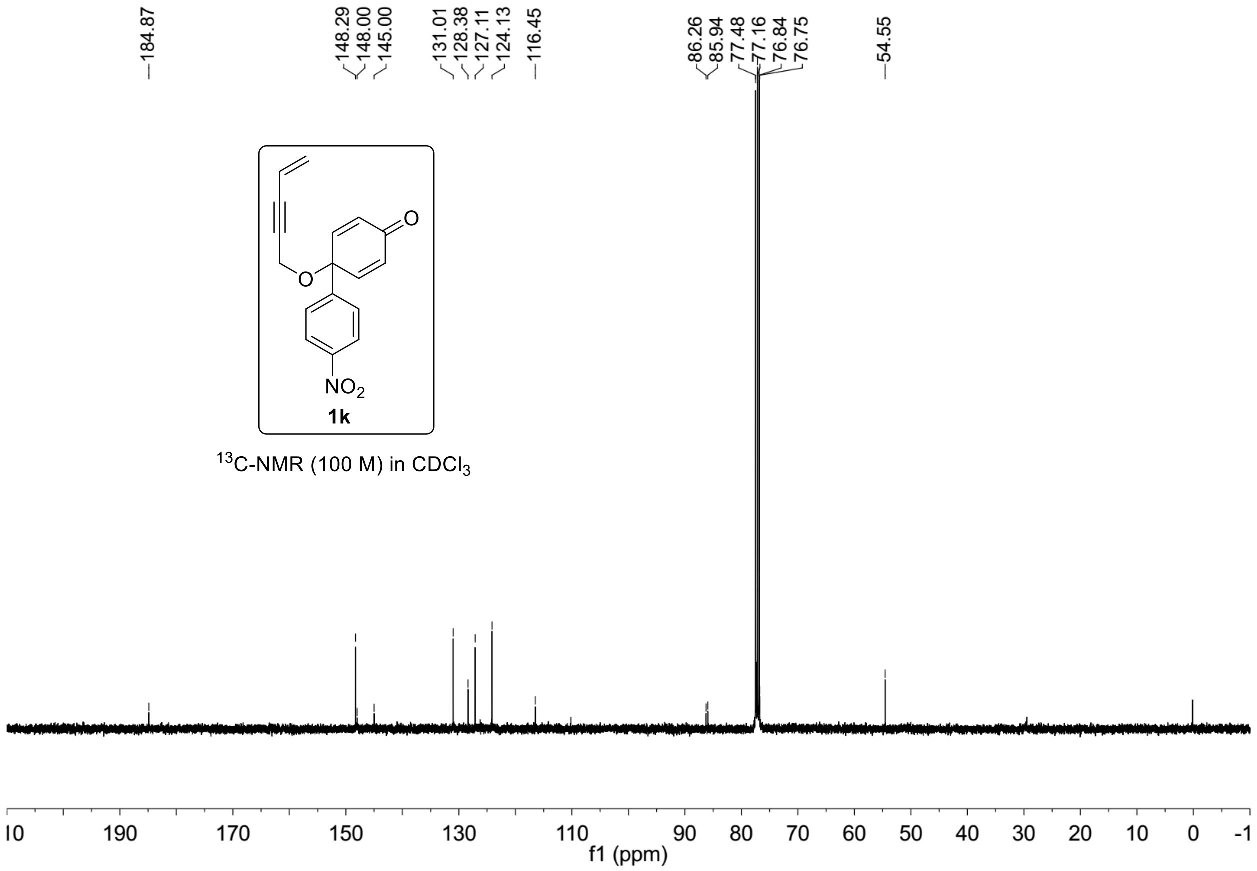

Supplementary Figure 23. ^{13}C NMR spectra for **1k**

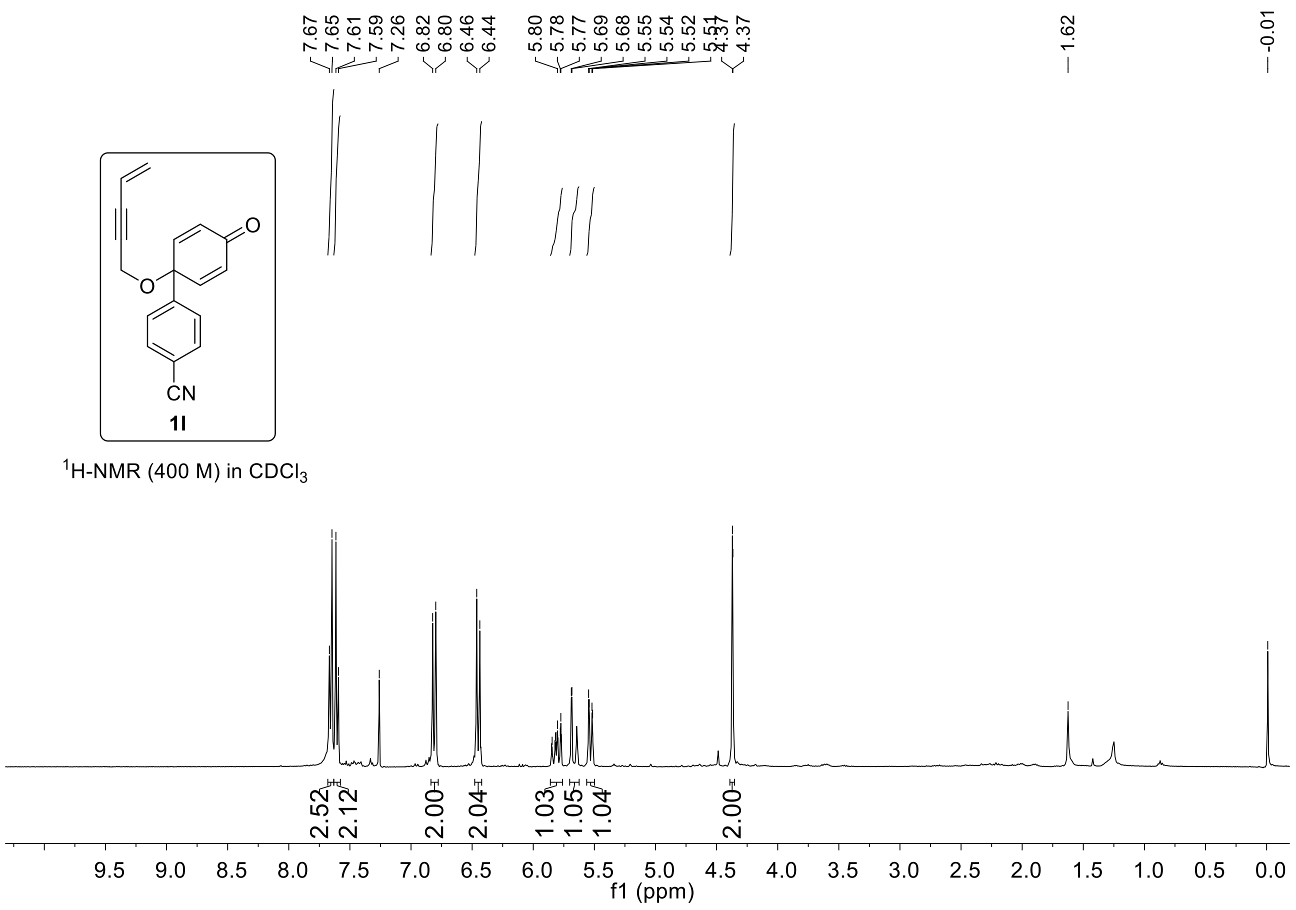

Supplementary Figure 24. ^{1}H NMR spectra for **1l**

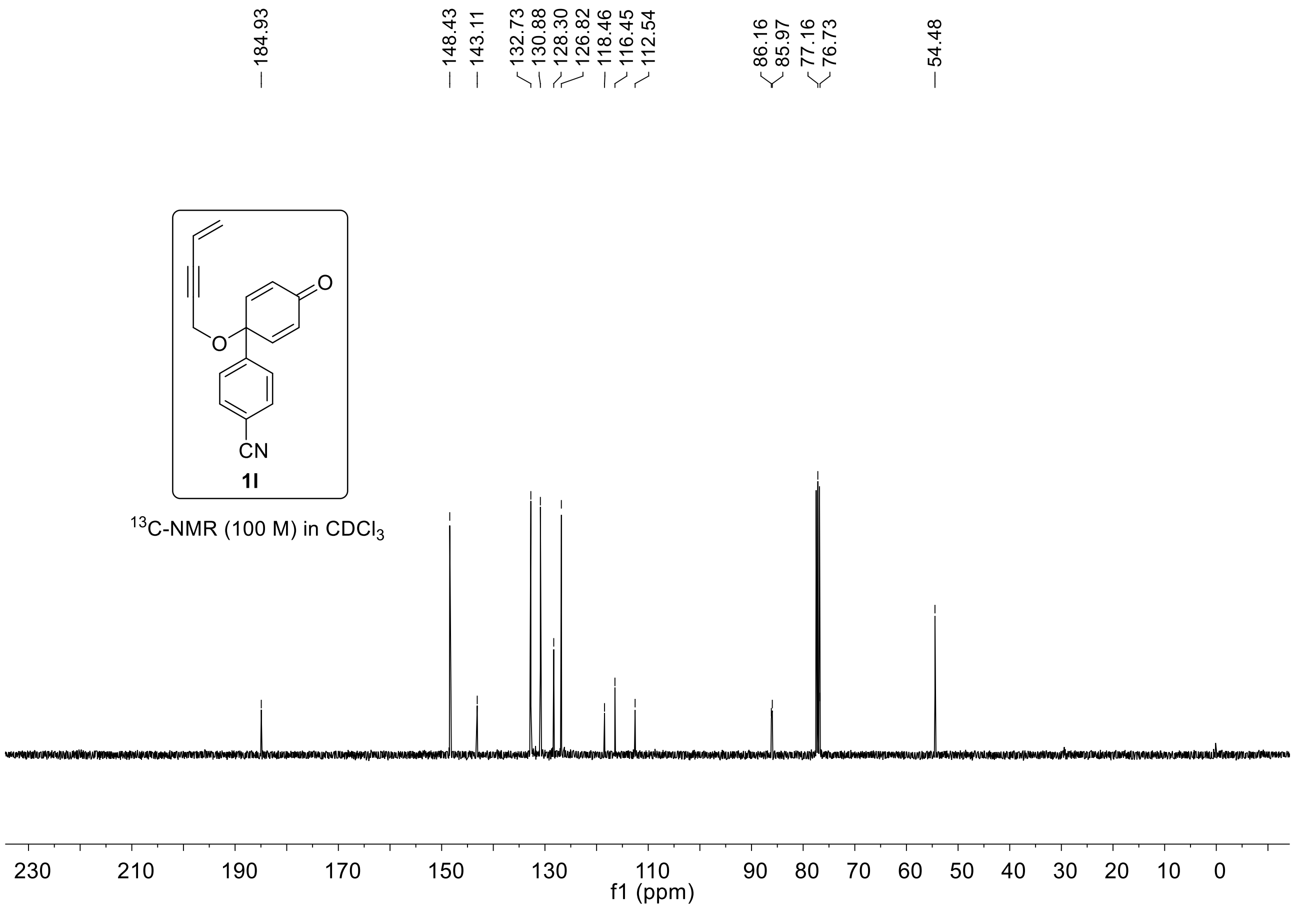

Supplementary Figure 25. ^{13}C NMR spectra for **1l**

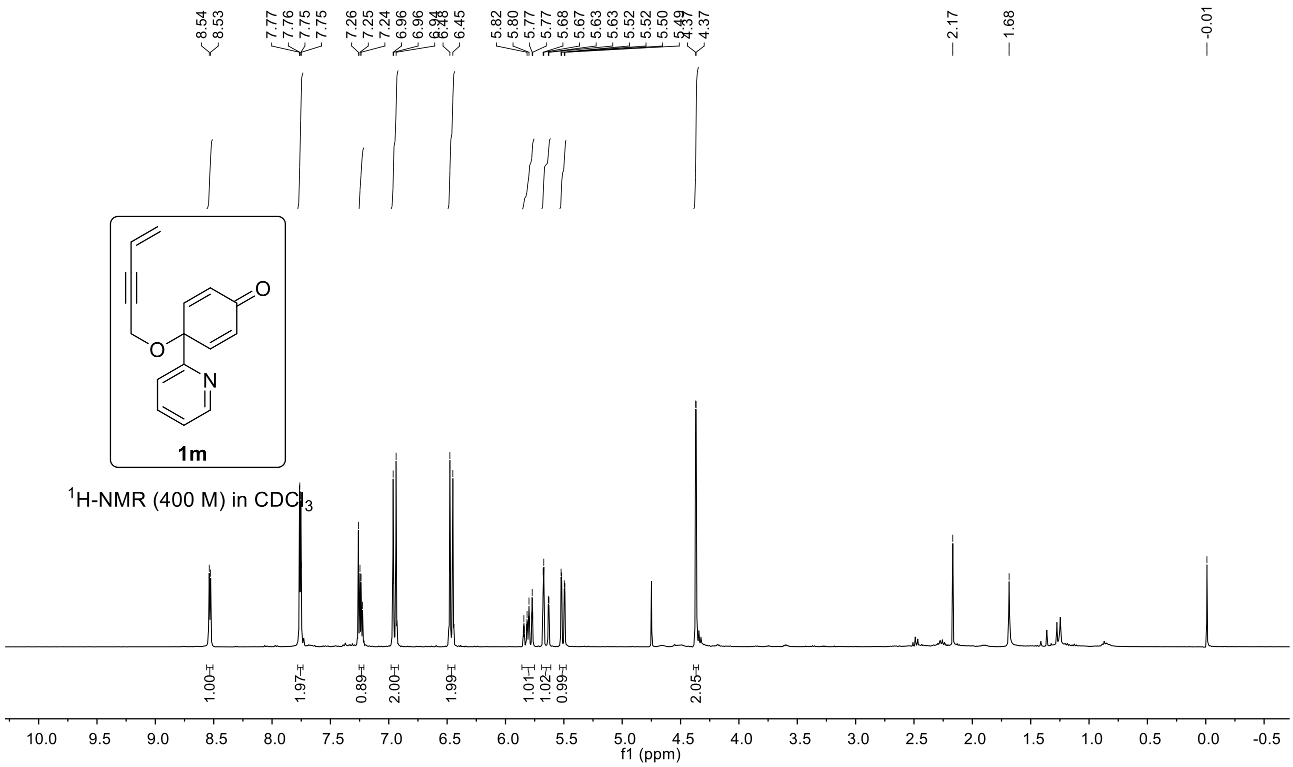

Supplementary Figure 26. ^{1}H NMR spectra for **1m**

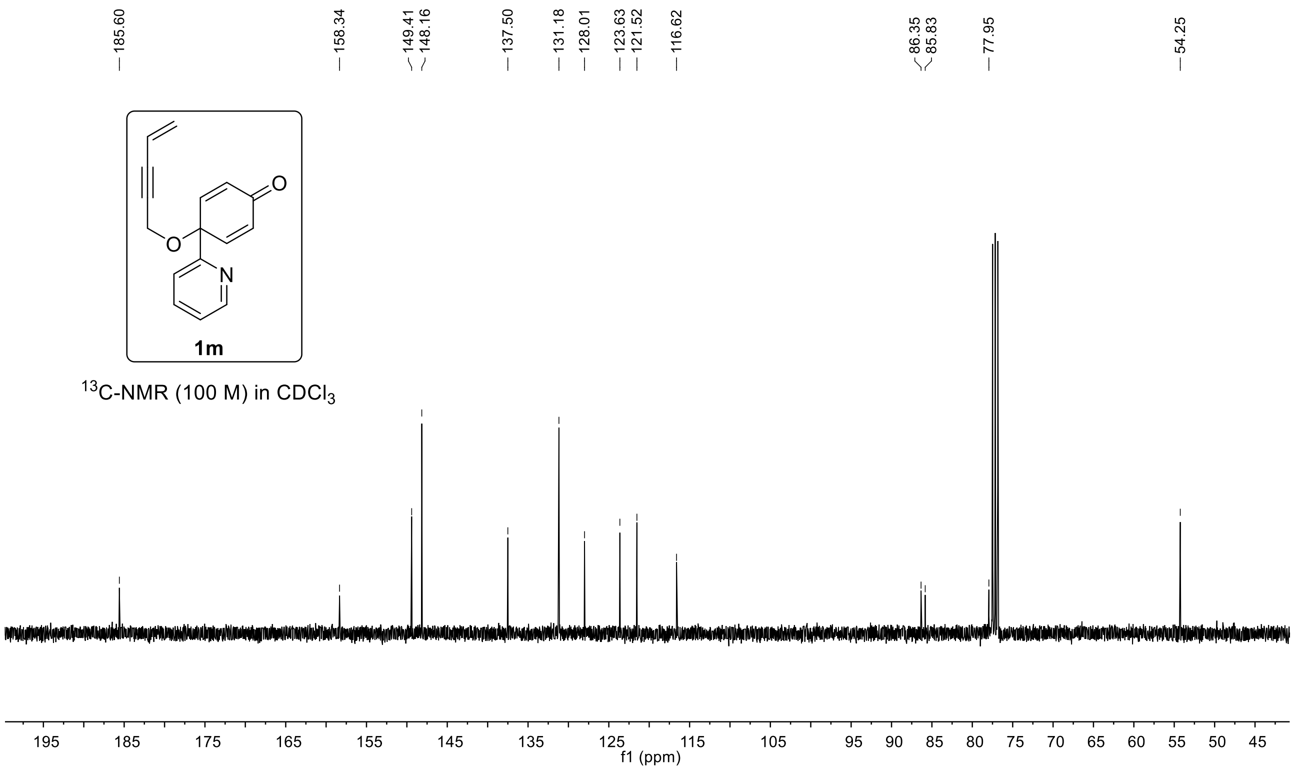

Supplementary Figure 27. ^{13}C NMR spectra for **1m**

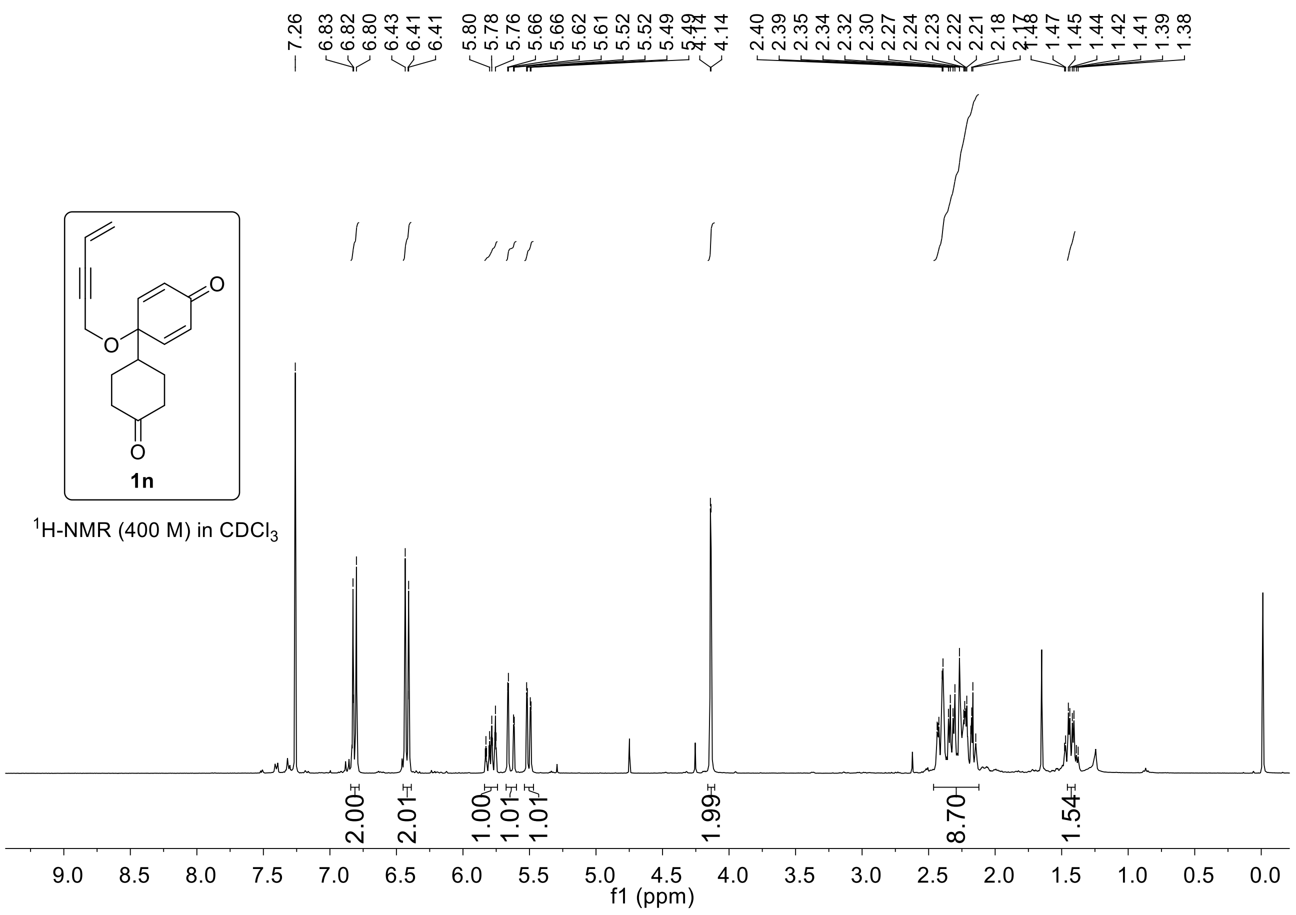

Supplementary Figure 28. ^{1}H NMR spectra for **1n**

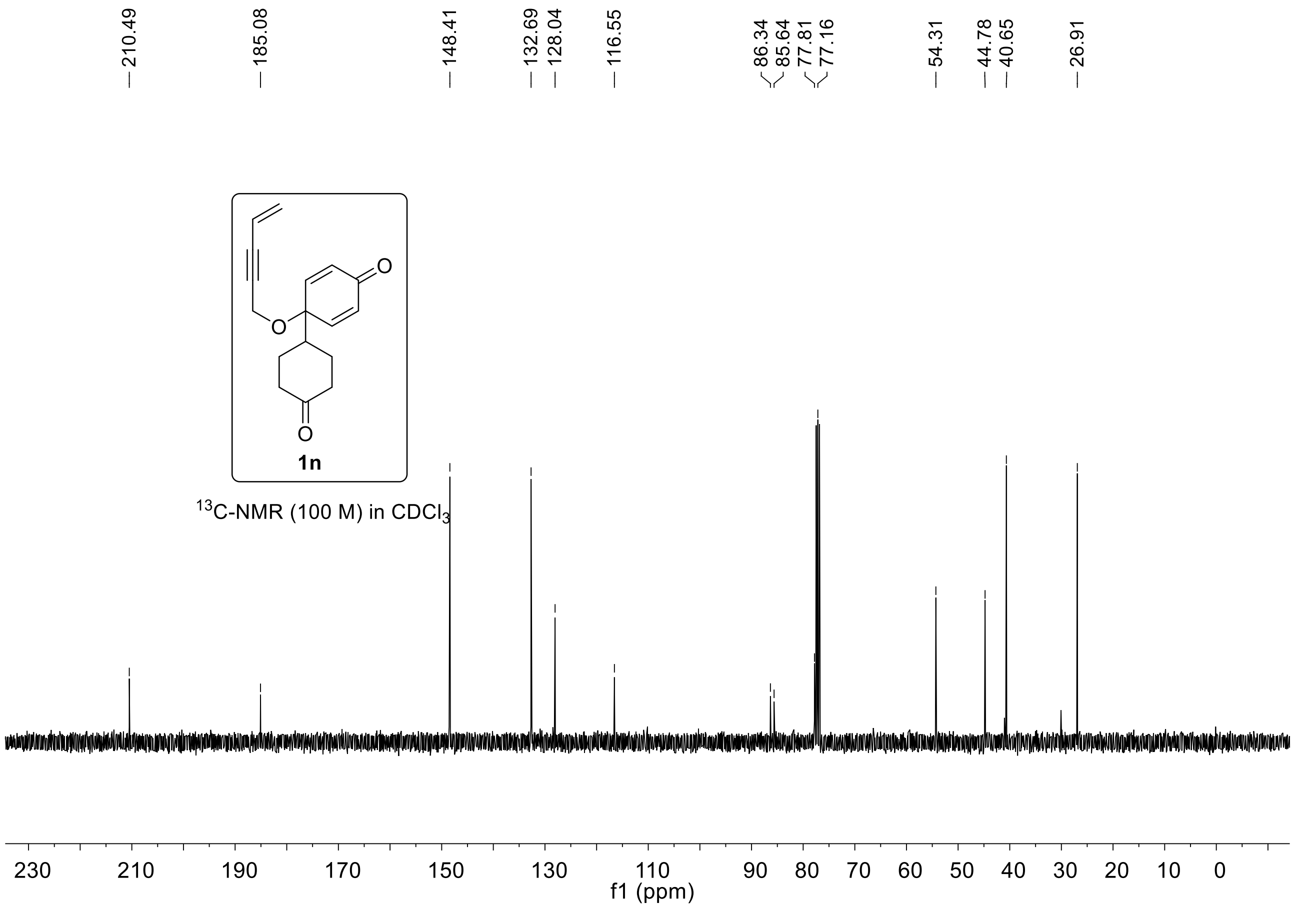

Supplementary Figure 29. ^{13}C NMR spectra for **1n**

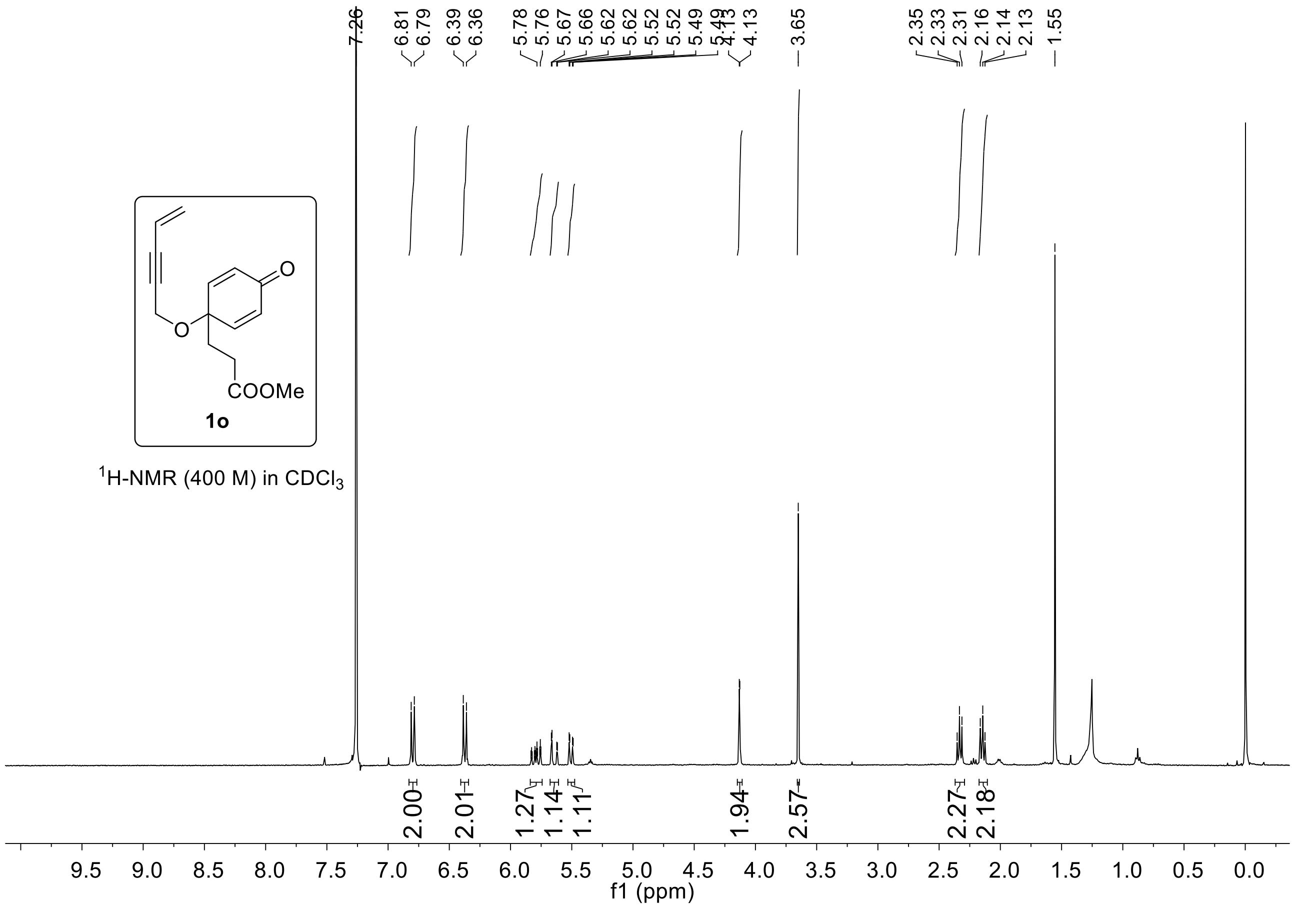

Supplementary Figure 30. ^{1}H NMR spectra for **1o**

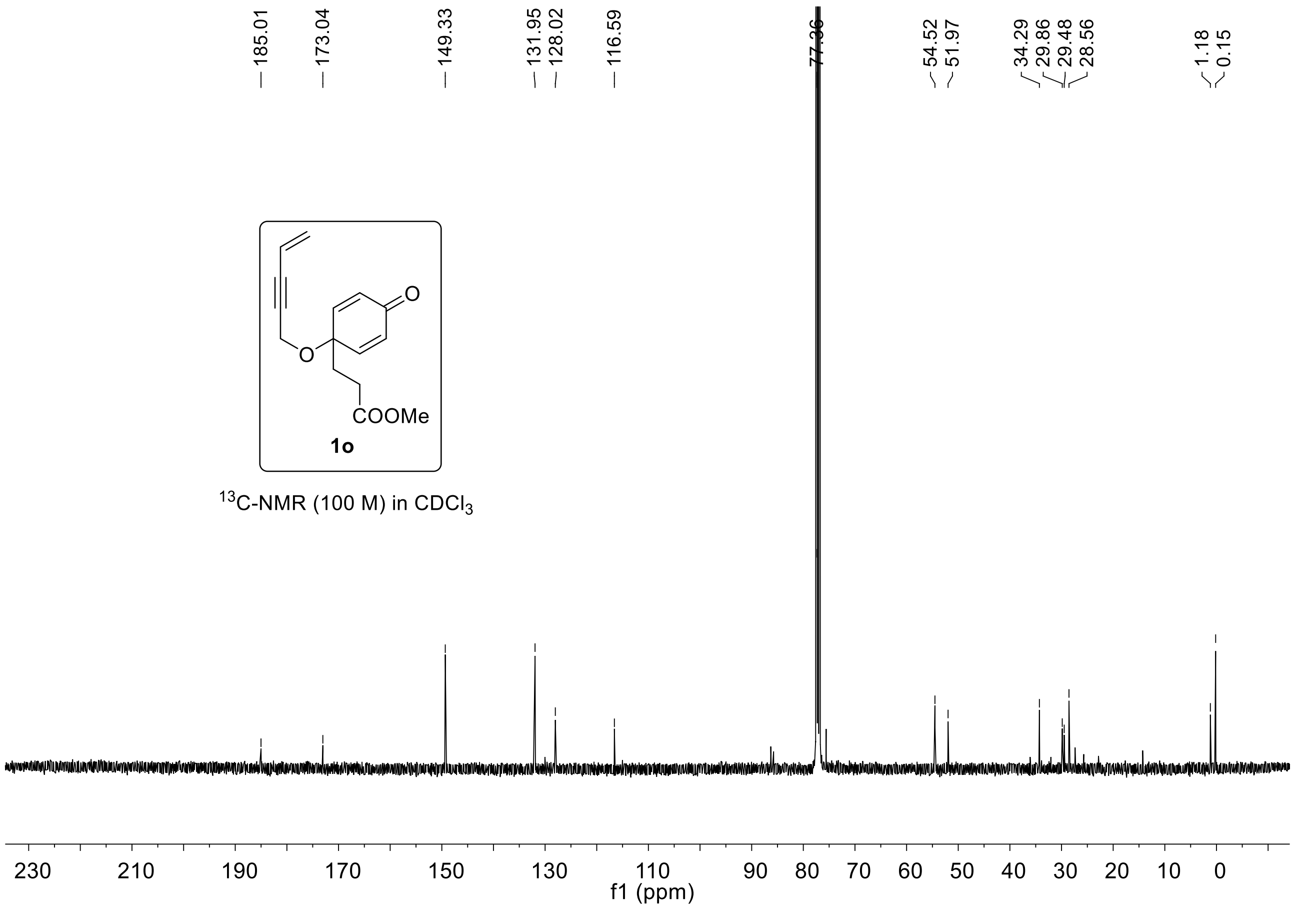

Supplementary Figure 31. ^{13}C NMR spectra for **1o**

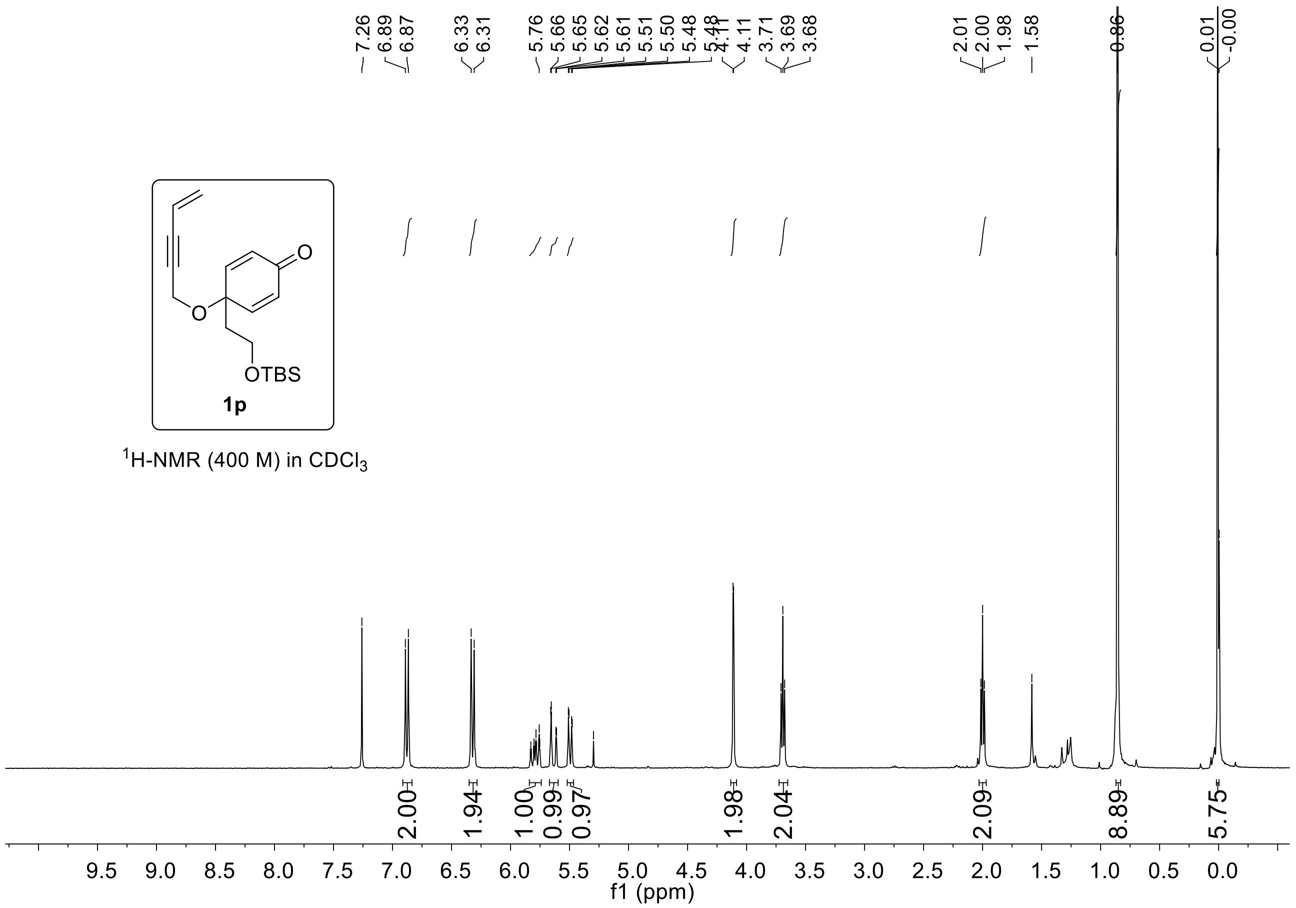

Supplementary Figure 32. ^{1}H NMR spectra for **1p**

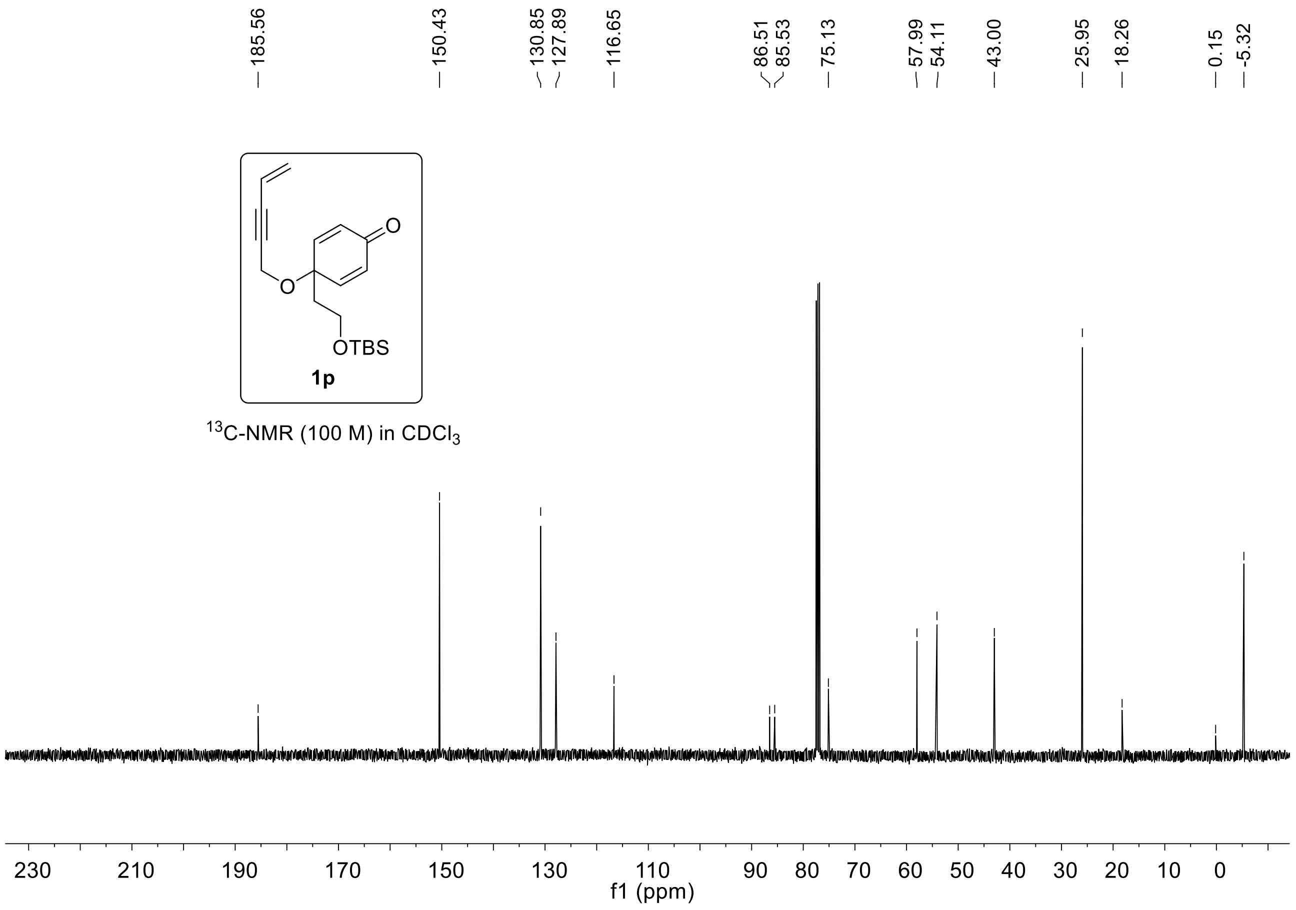

Supplementary Figure 33. ^{13}C NMR spectra for **1p**

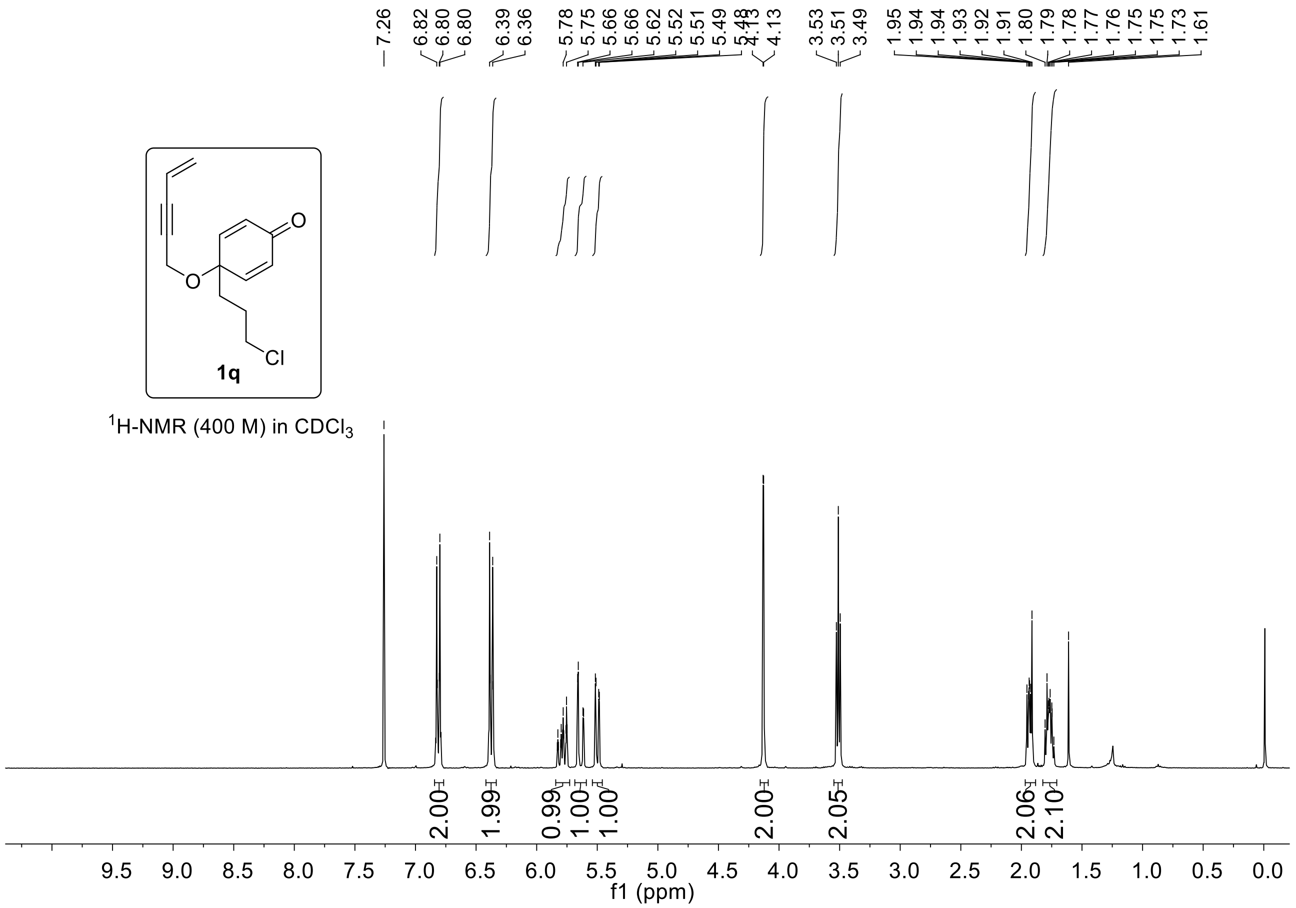

Supplementary Figure 34. ^{1}H NMR spectra for **1q**

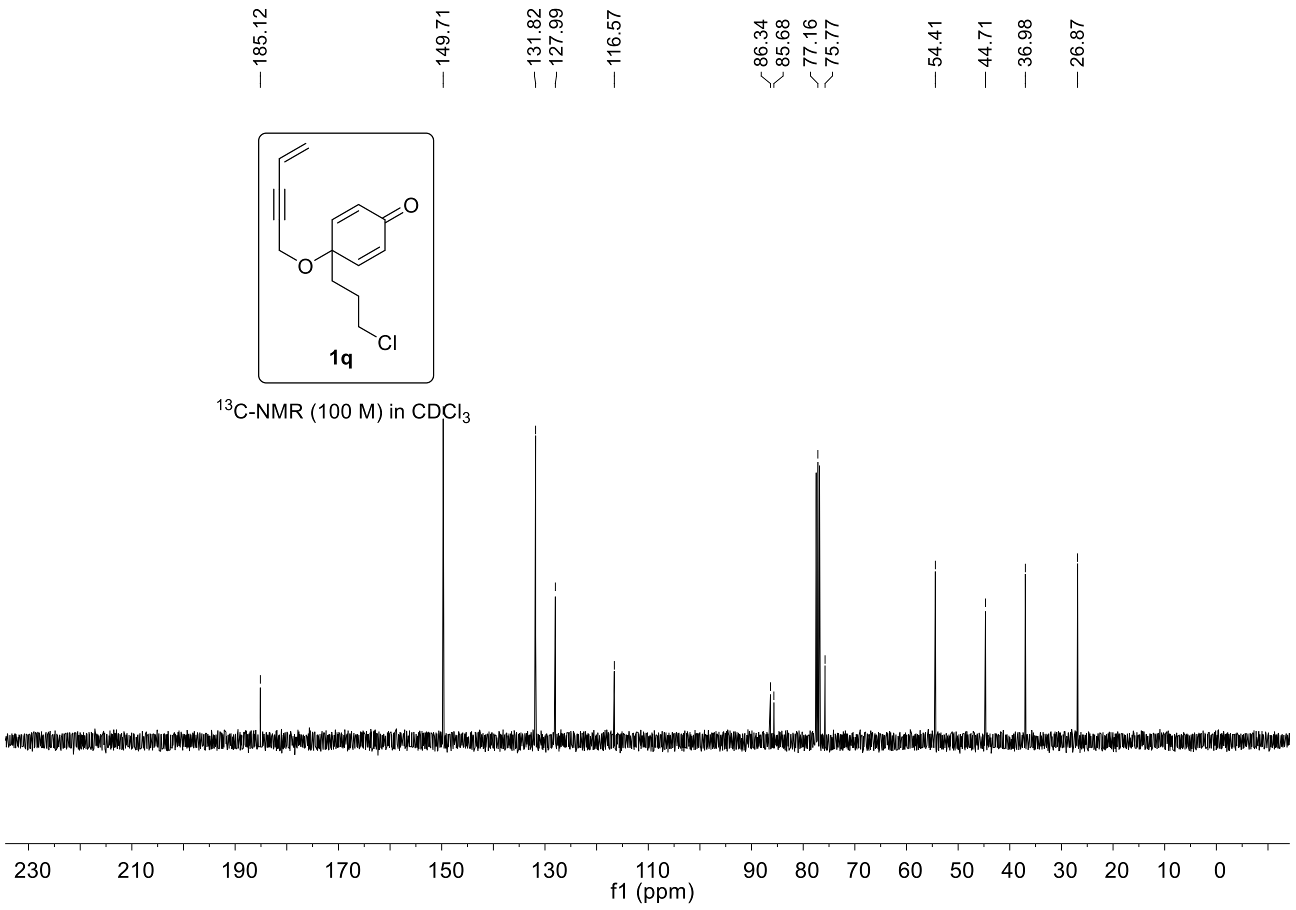

Supplementary Figure 35. ^{13}C NMR spectra for **1q**

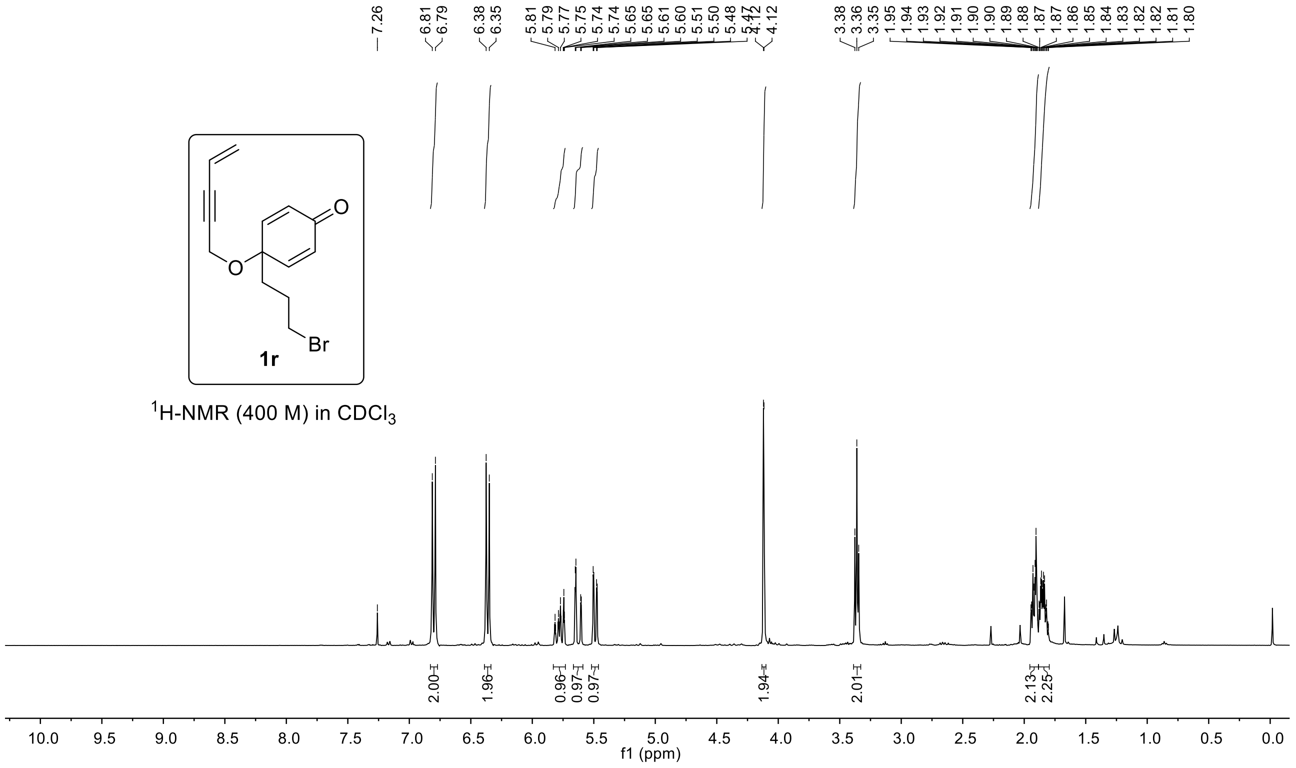

Supplementary Figure 36. ^{1}H NMR spectra for **1r**

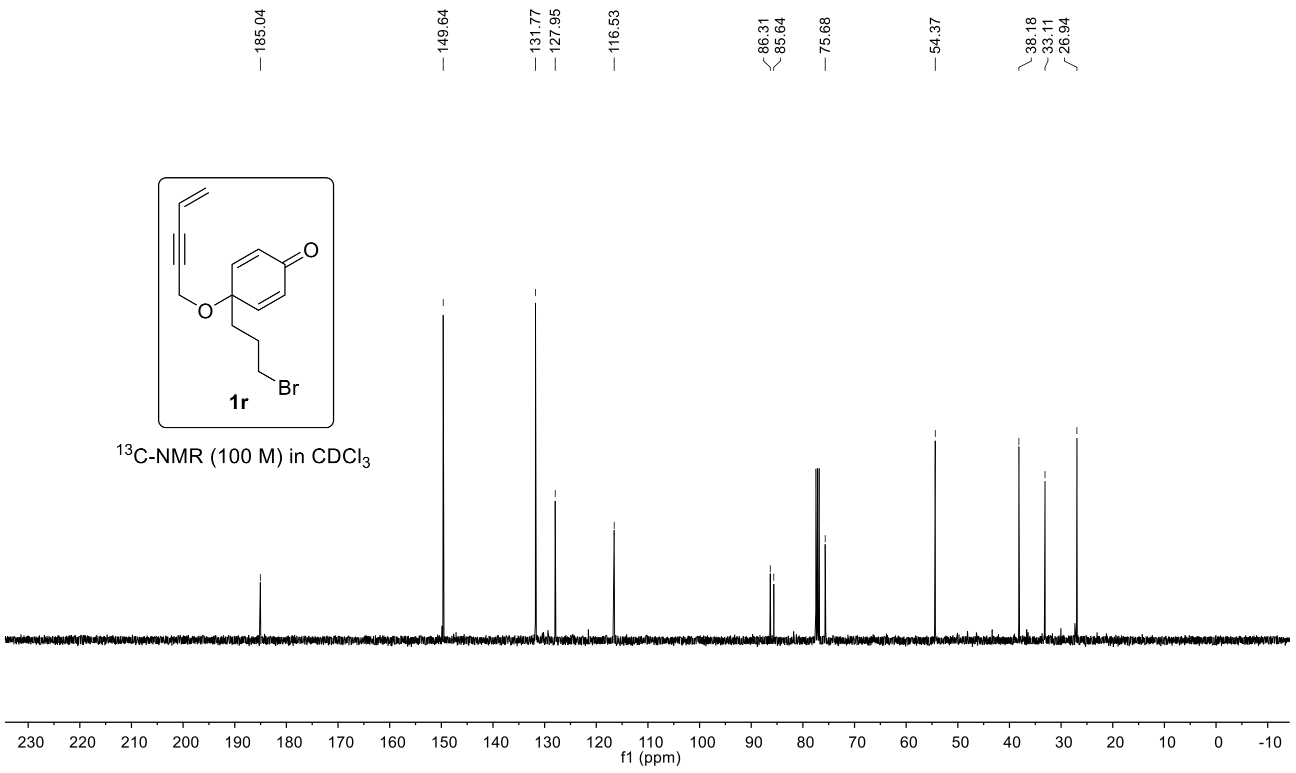

Supplementary Figure 37. ^{13}C NMR spectra for **1r**

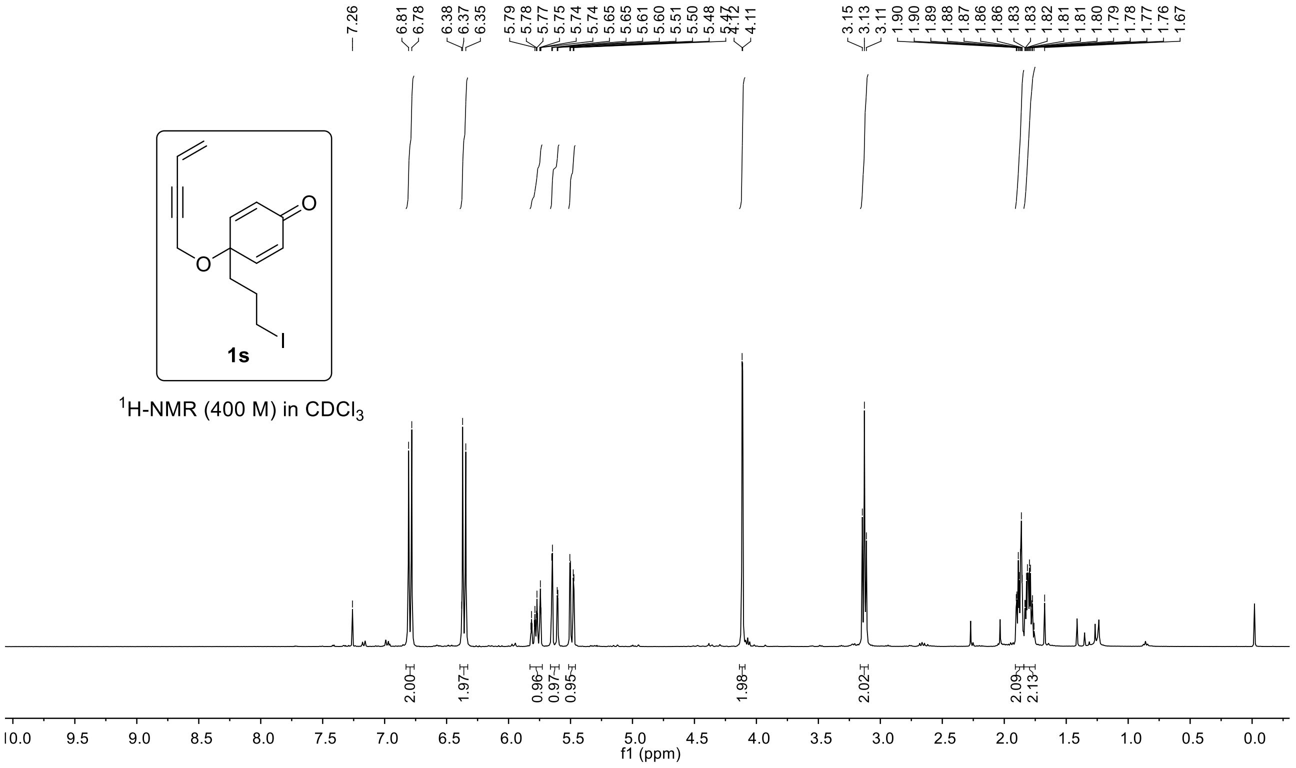

Supplementary Figure 38. ^{1}H NMR spectra for **1s**

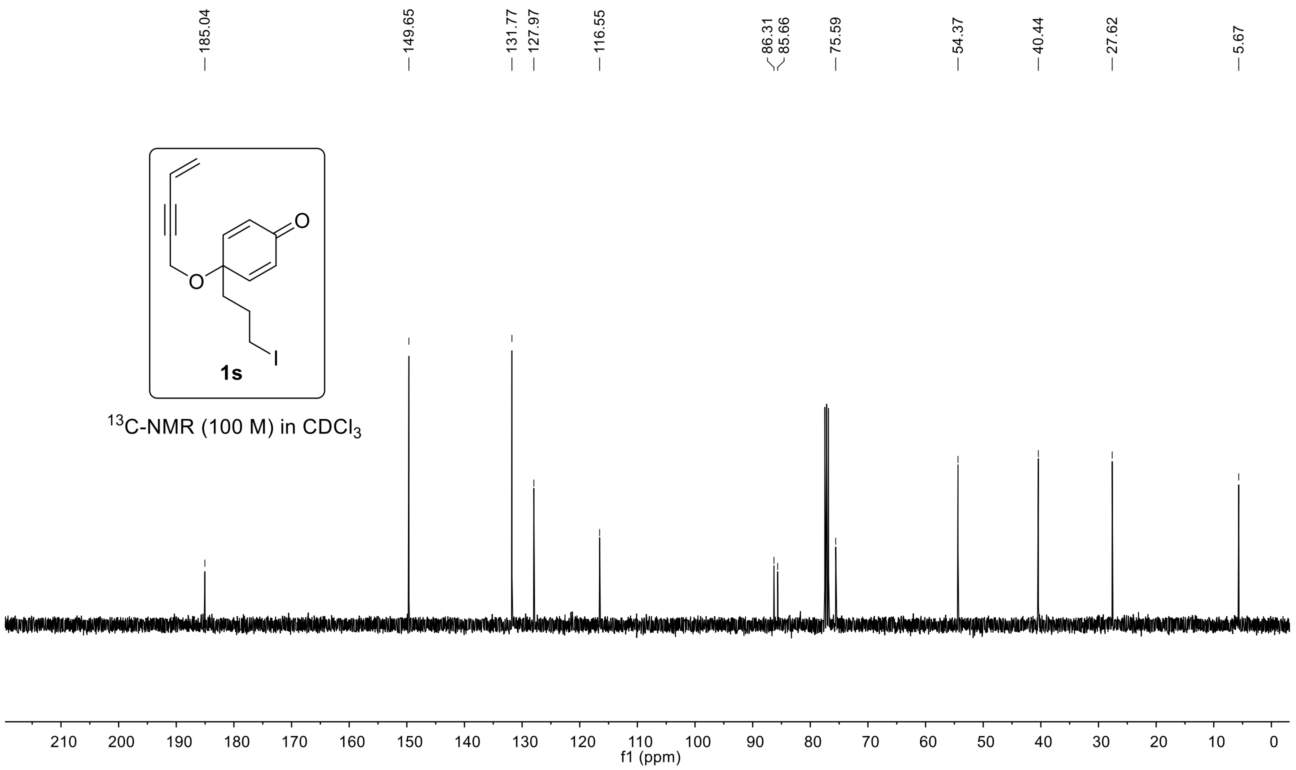

Supplementary Figure 39. ^{13}C NMR spectra for **1s**

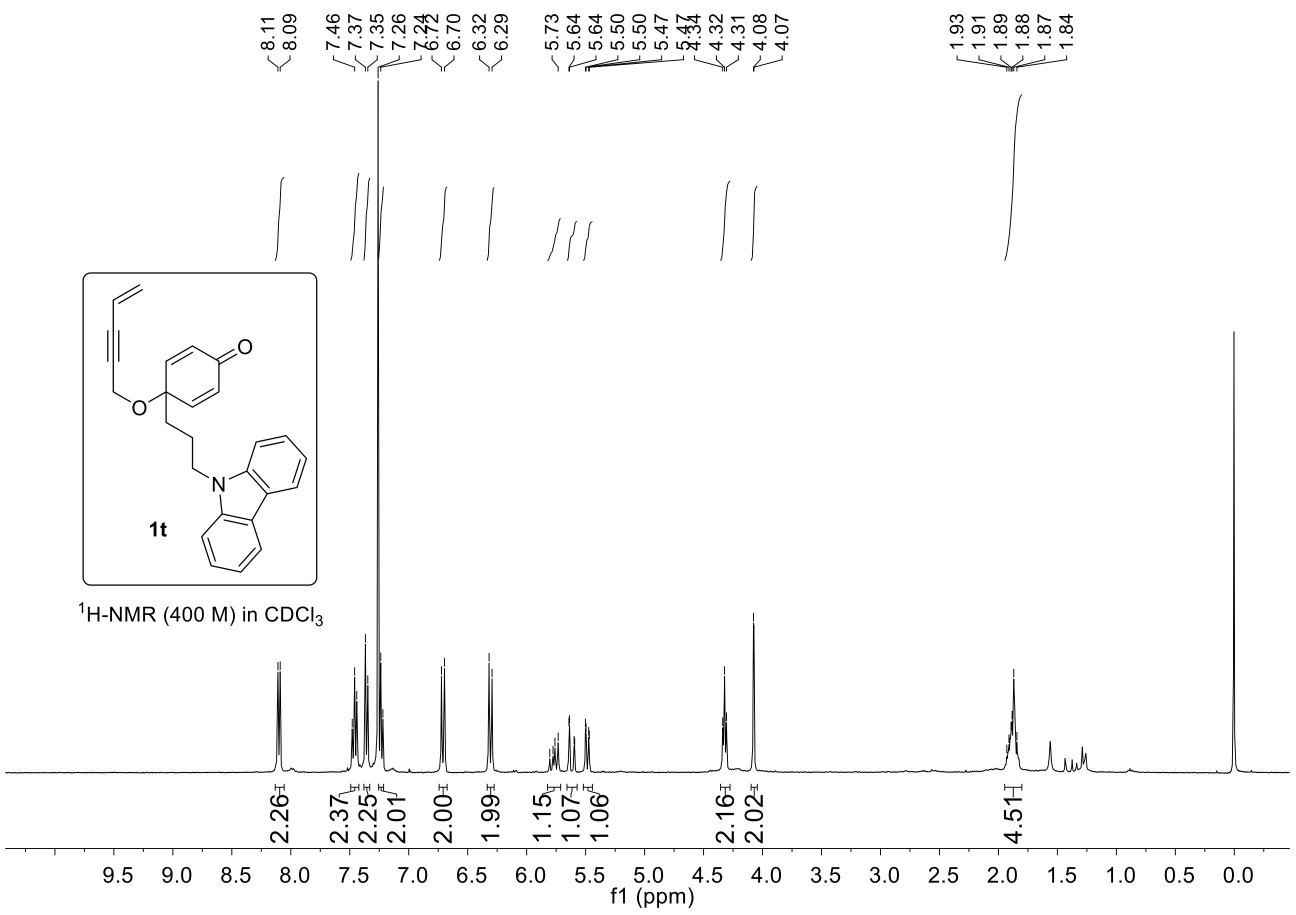

Supplementary Figure 40. ^{1}H NMR spectra for **1t**

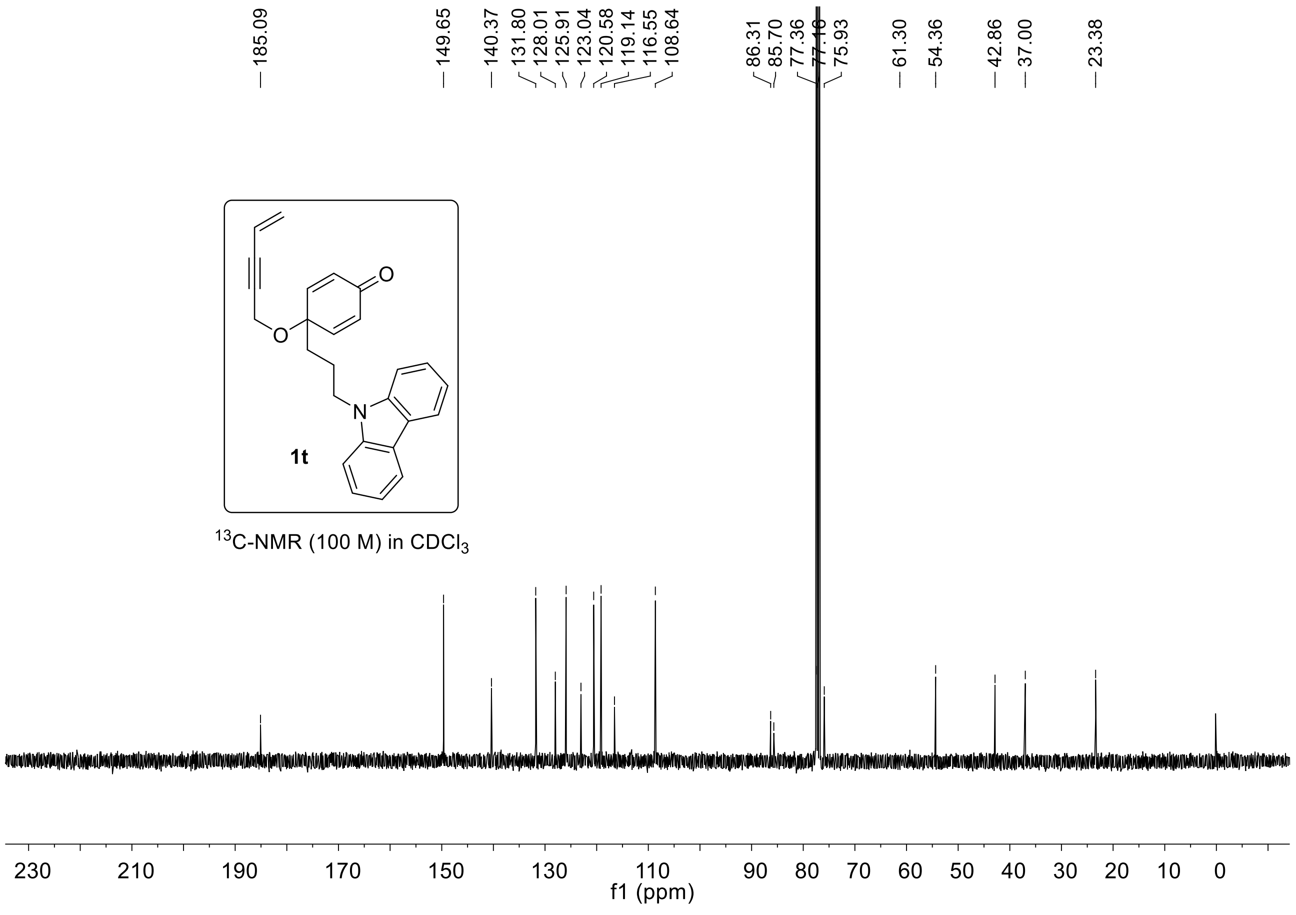

Supplementary Figure 41. ^{13}C NMR spectra for **1t**

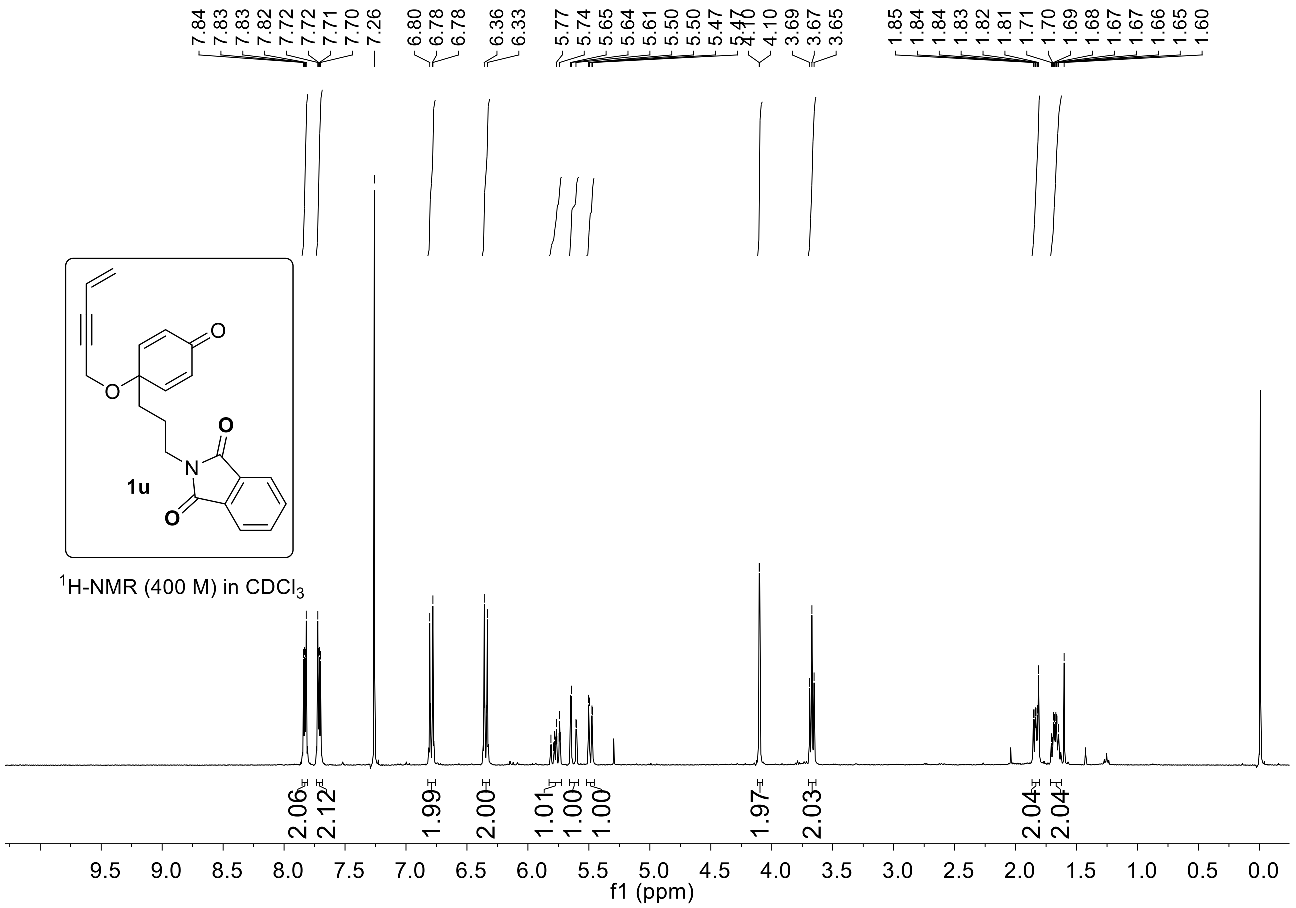

Supplementary Figure 42. ^{1}H NMR spectra for **1u**

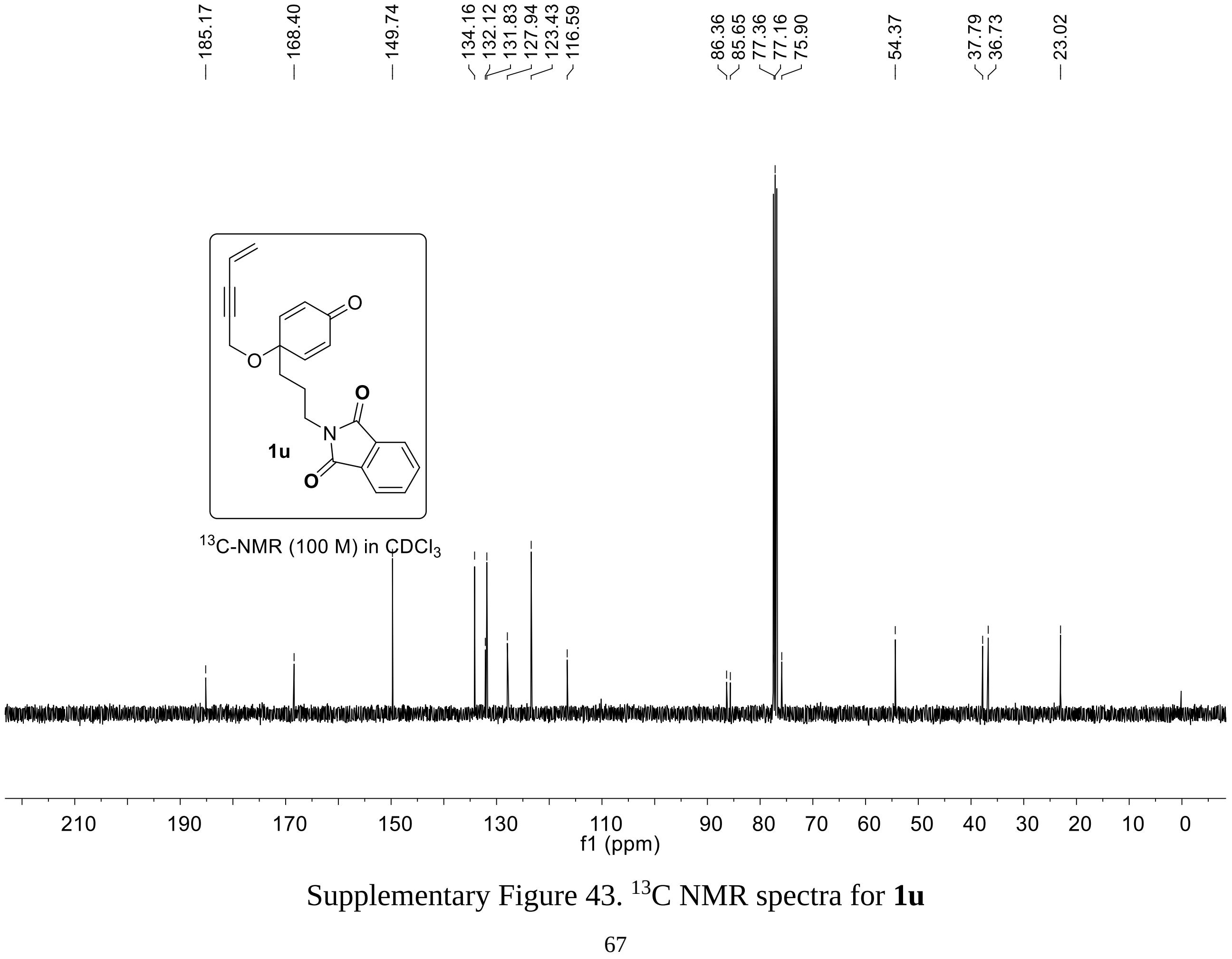

Supplementary Figure 43. ^{13}C NMR spectra for **1u**

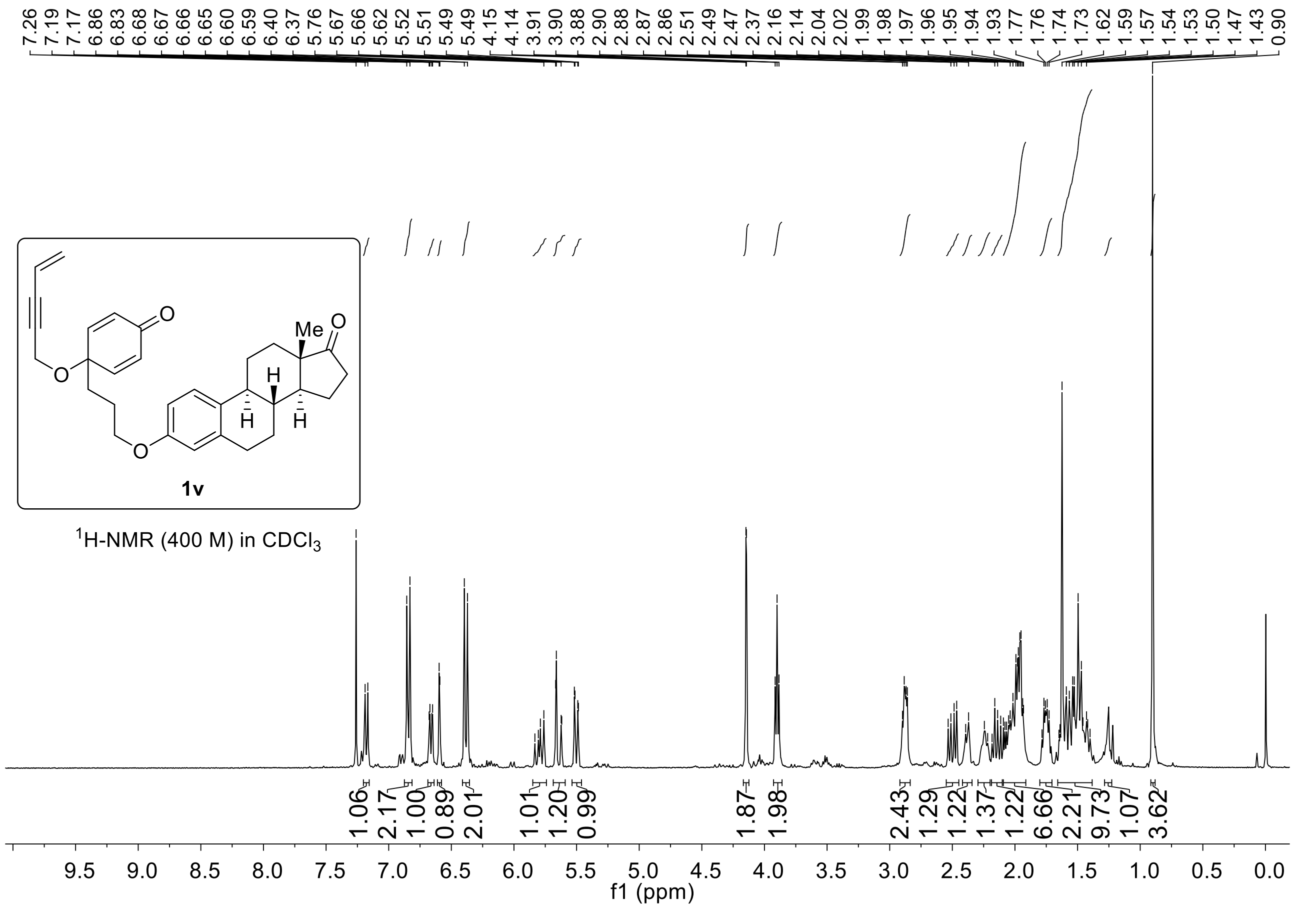

Supplementary Figure 44. ^{1}H NMR spectra for **1v**

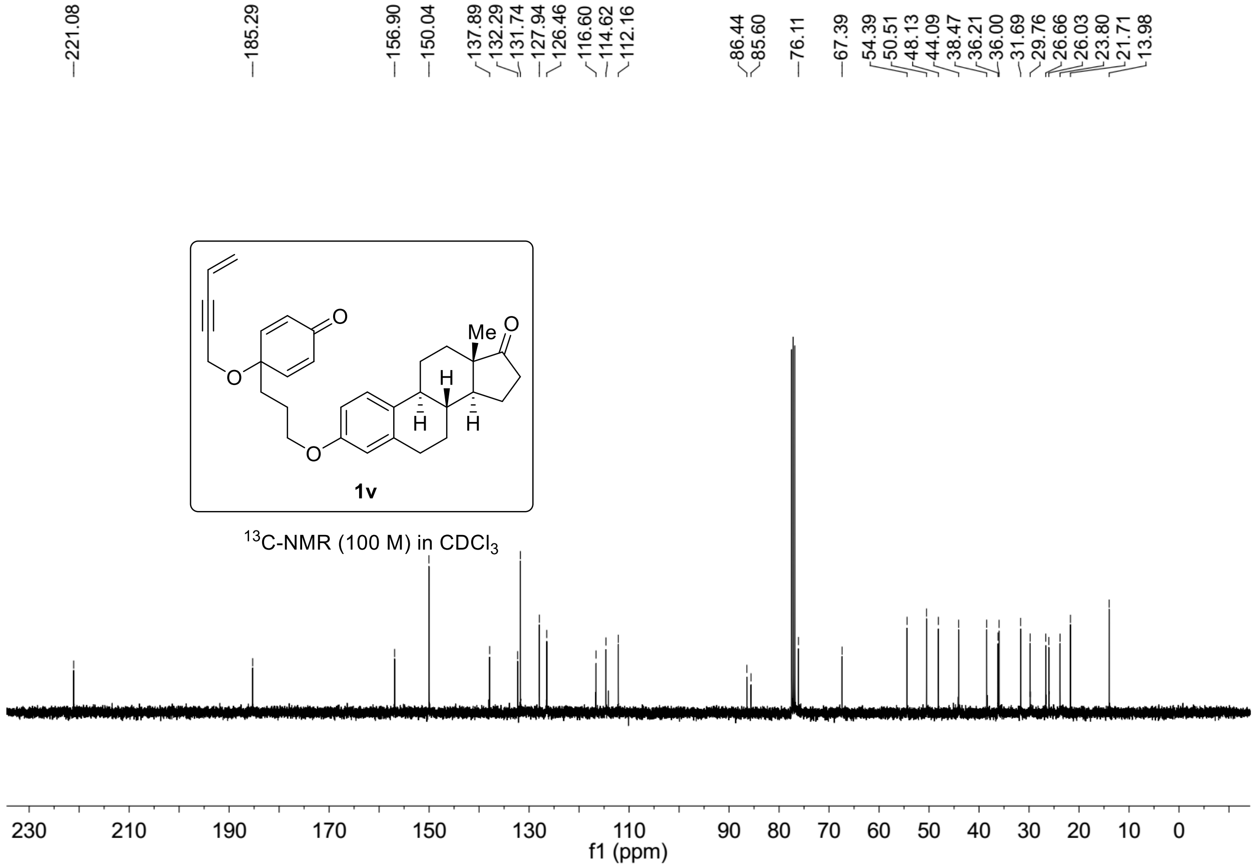

Supplementary Figure 45. ^{13}C NMR spectra for **1v**

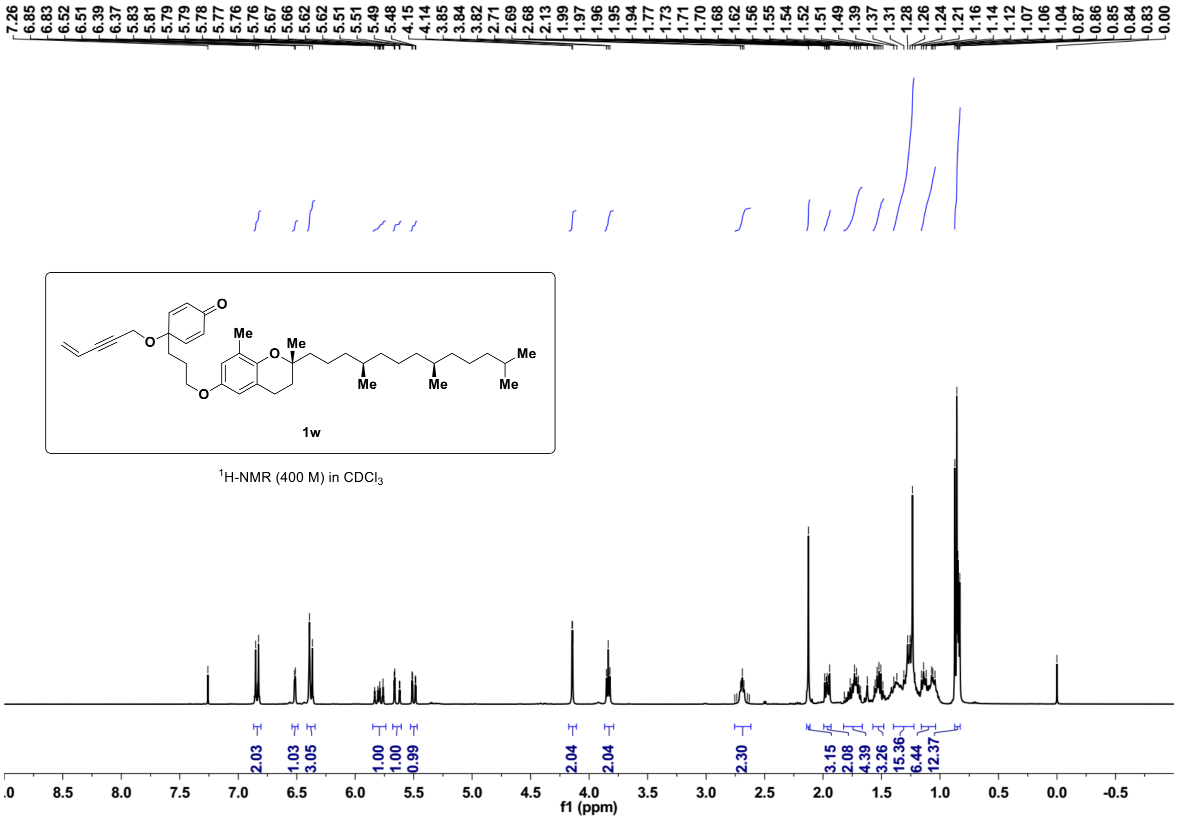

Supplementary Figure 46. ^{1}H NMR spectra for **1w**

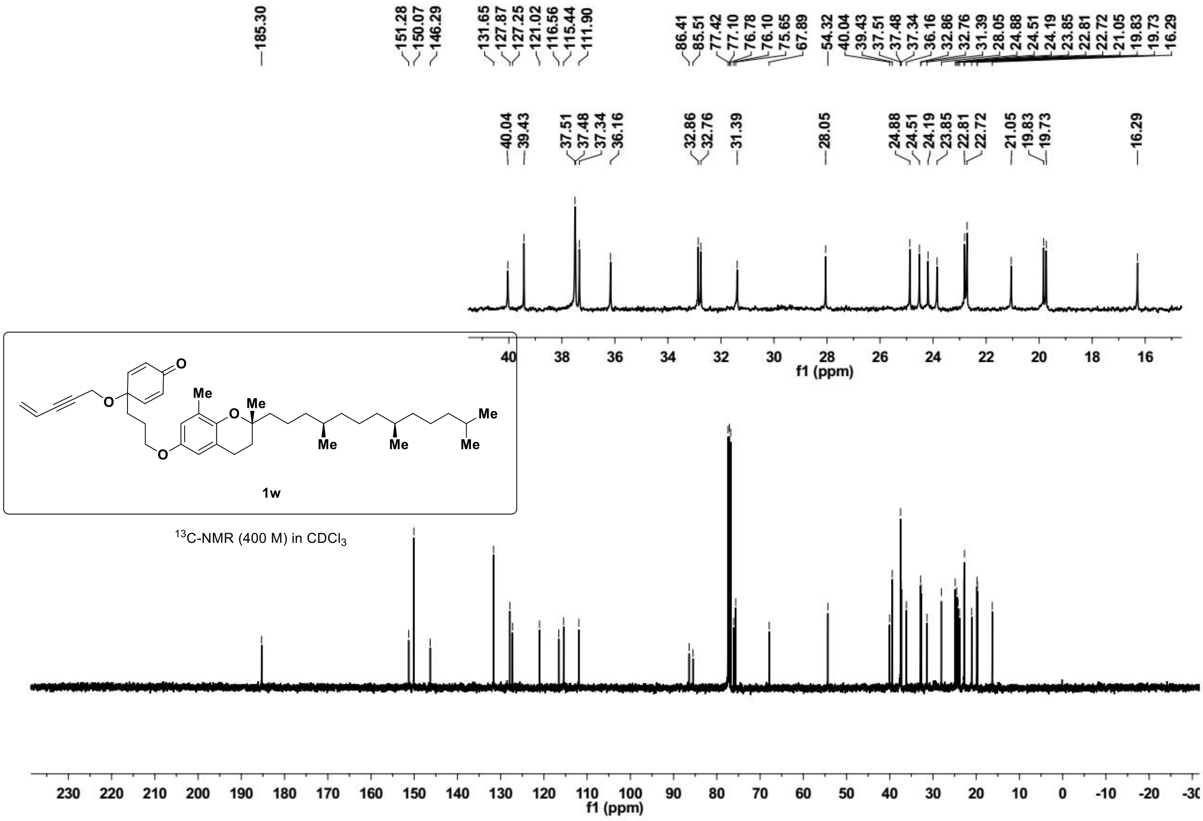

Supplementary Figure 47. ^{13}C NMR spectra for **1w**

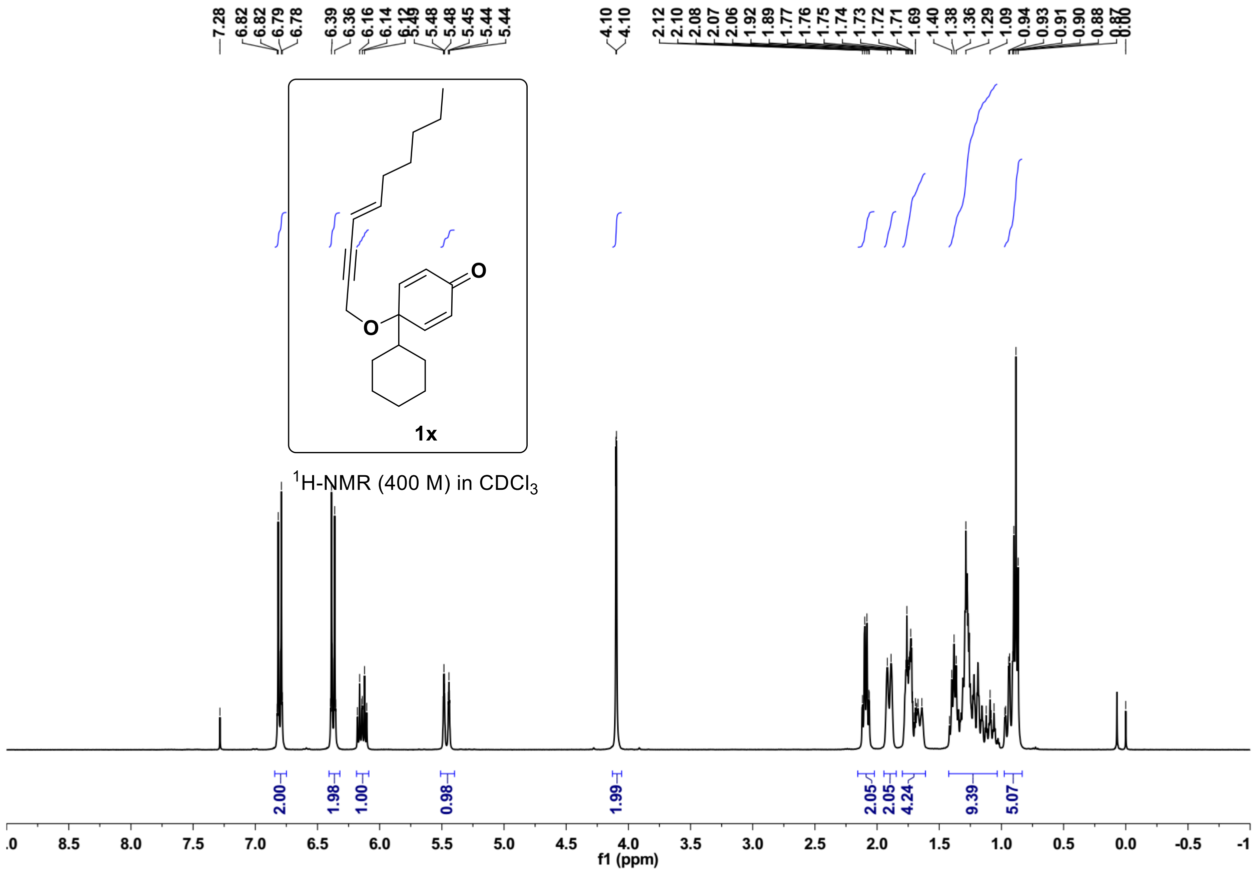

Supplementary Figure 48. ^{1}H NMR spectra for **1x**

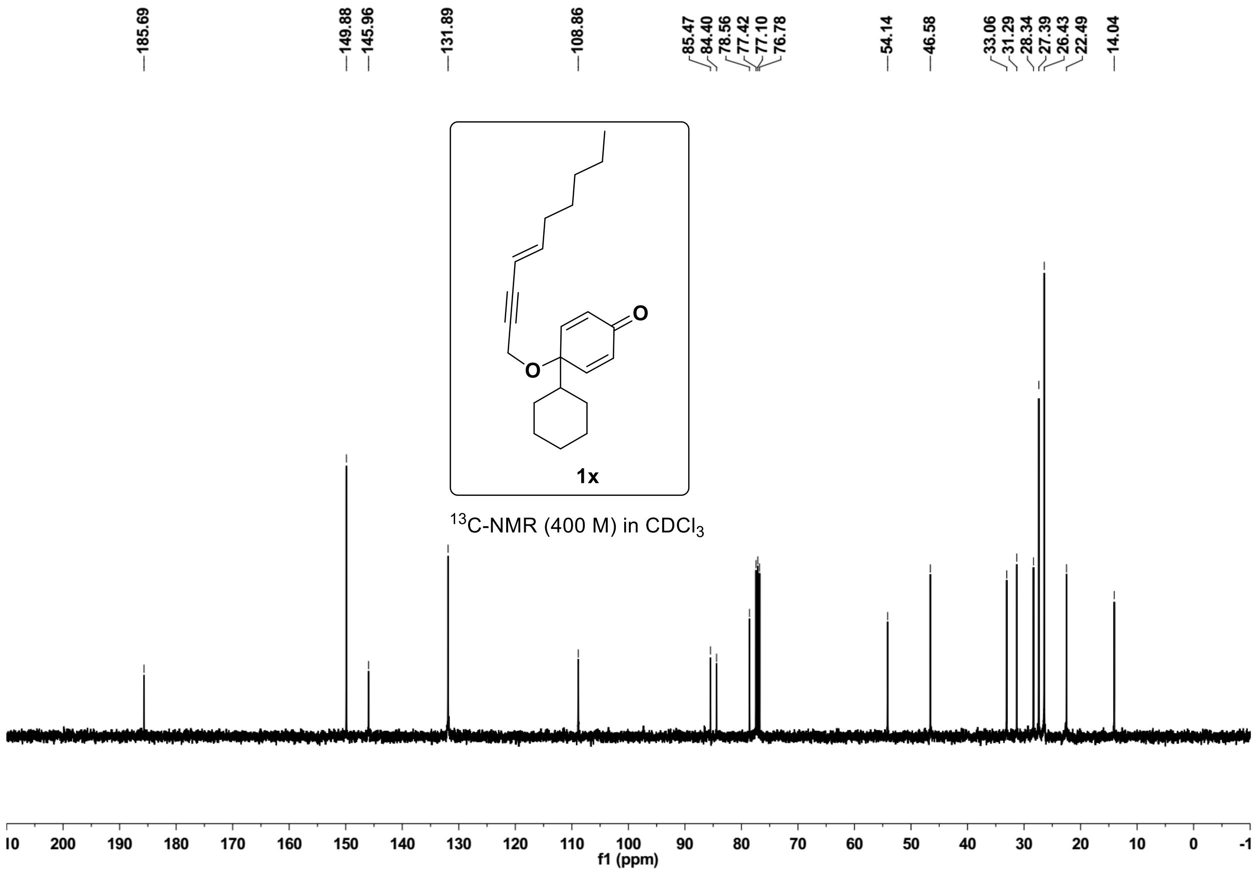

Supplementary Figure 49. ^{13}C NMR spectra for **1x**

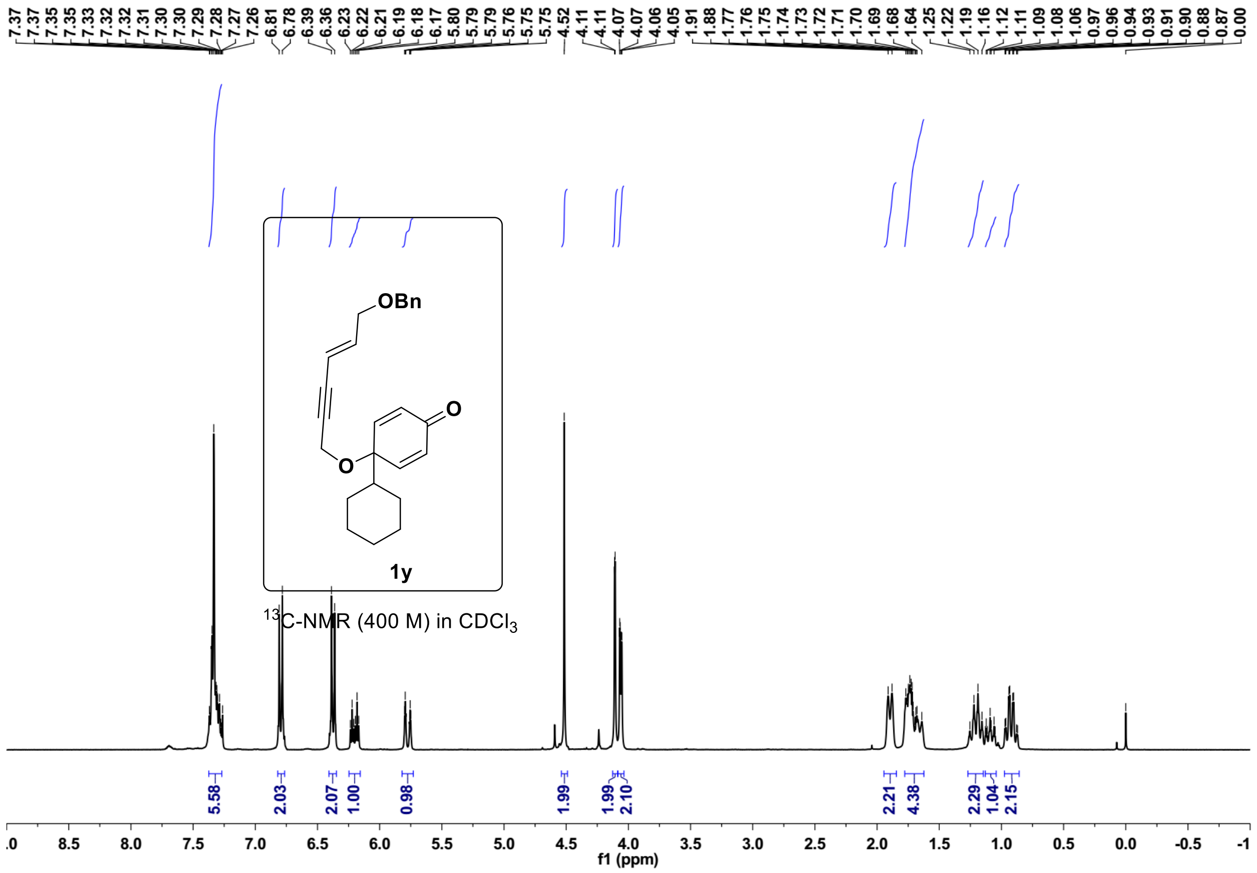

Supplementary Figure 50. ^{1}H NMR spectra for **1y**

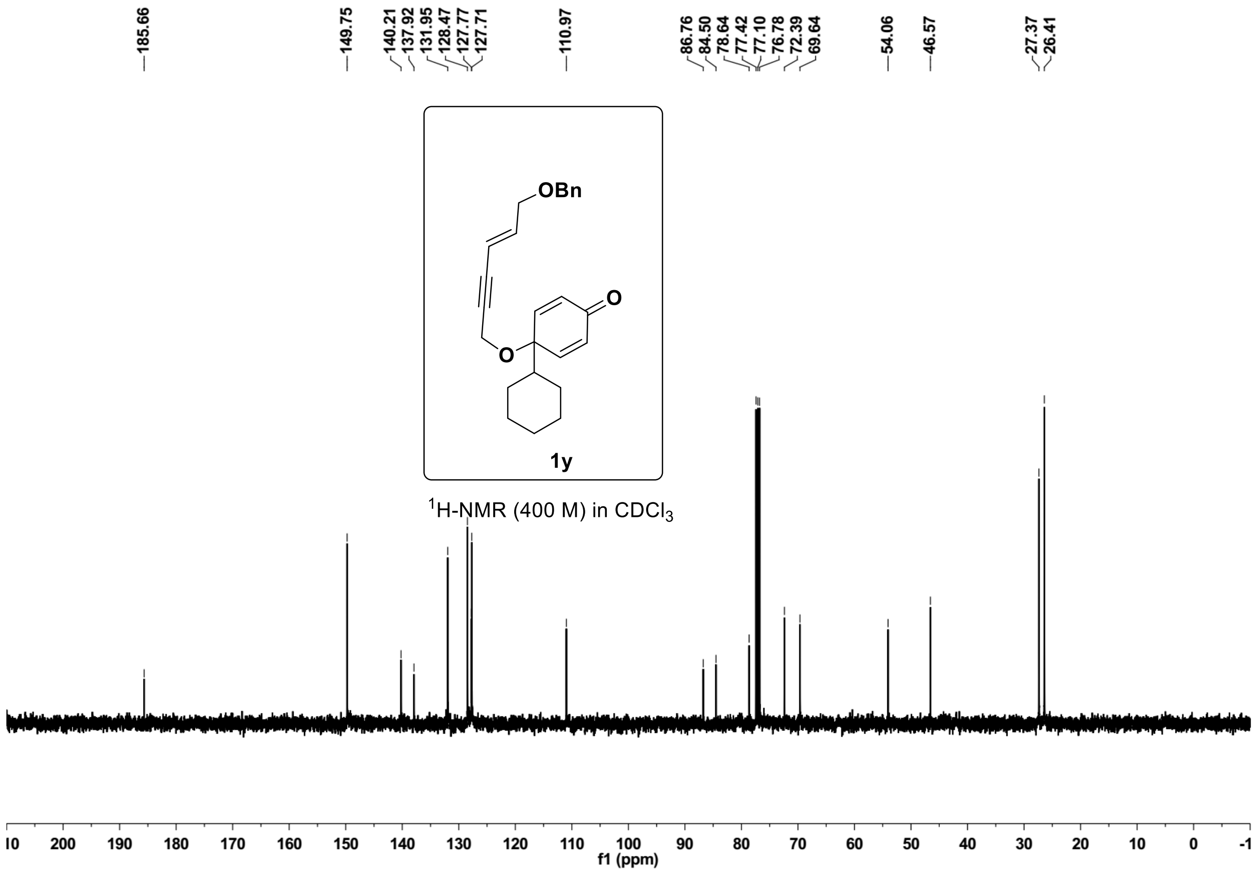

Supplementary Figure 51. ^{13}C NMR spectra for **1y**

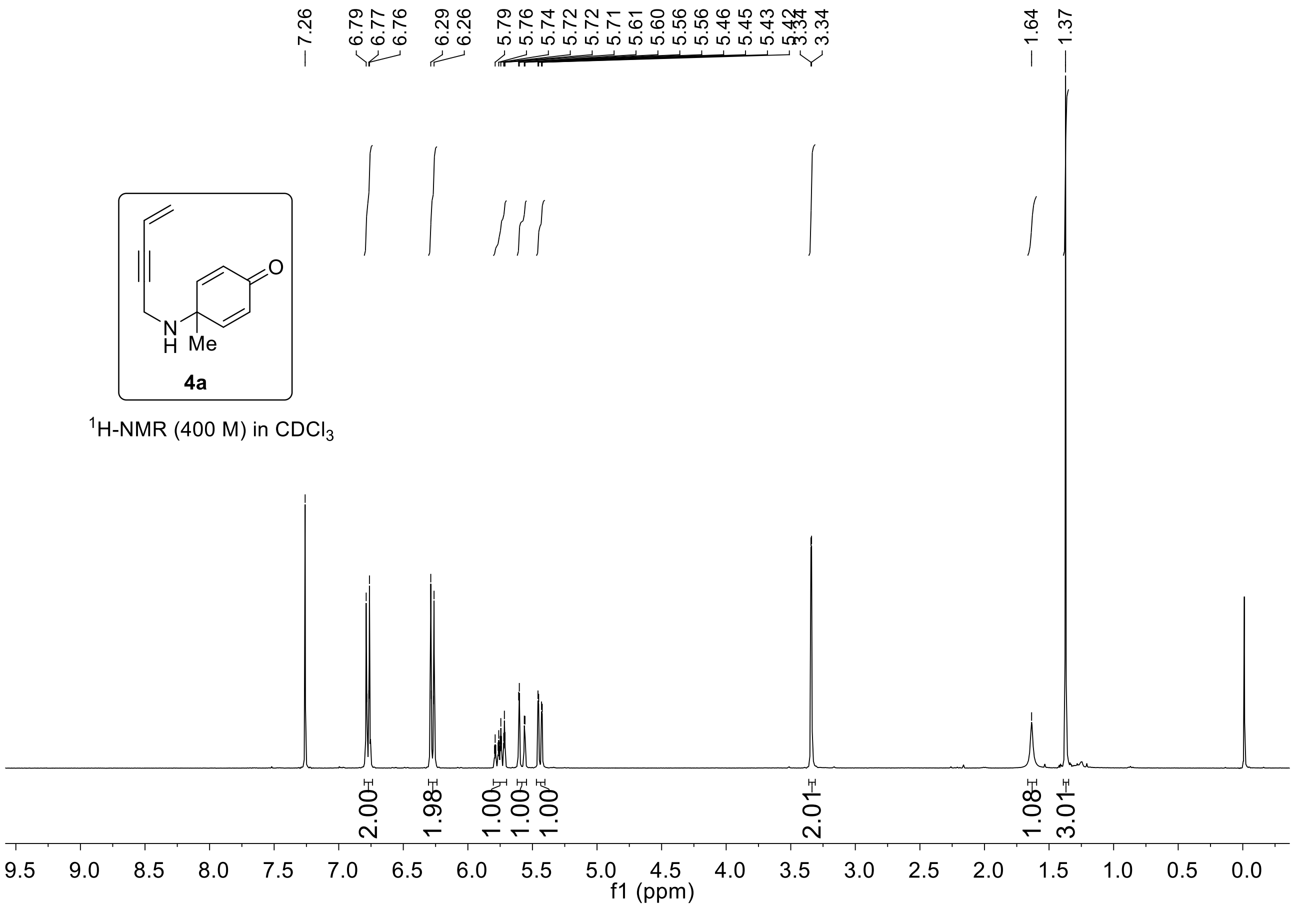

Supplementary Figure 52. ^{1}H NMR spectra for **4a**

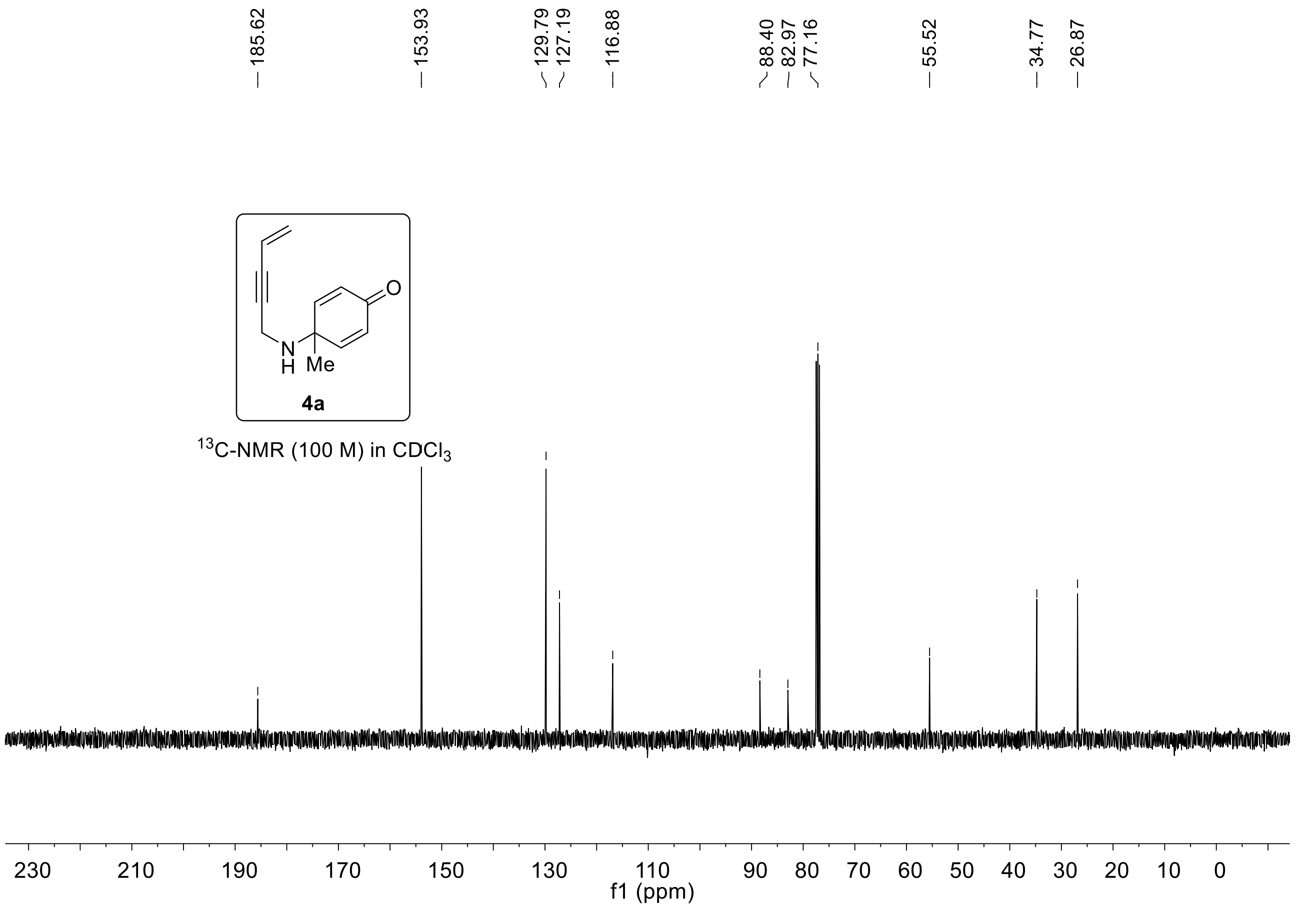

Supplementary Figure 53. ^{13}C NMR spectra for **4a**

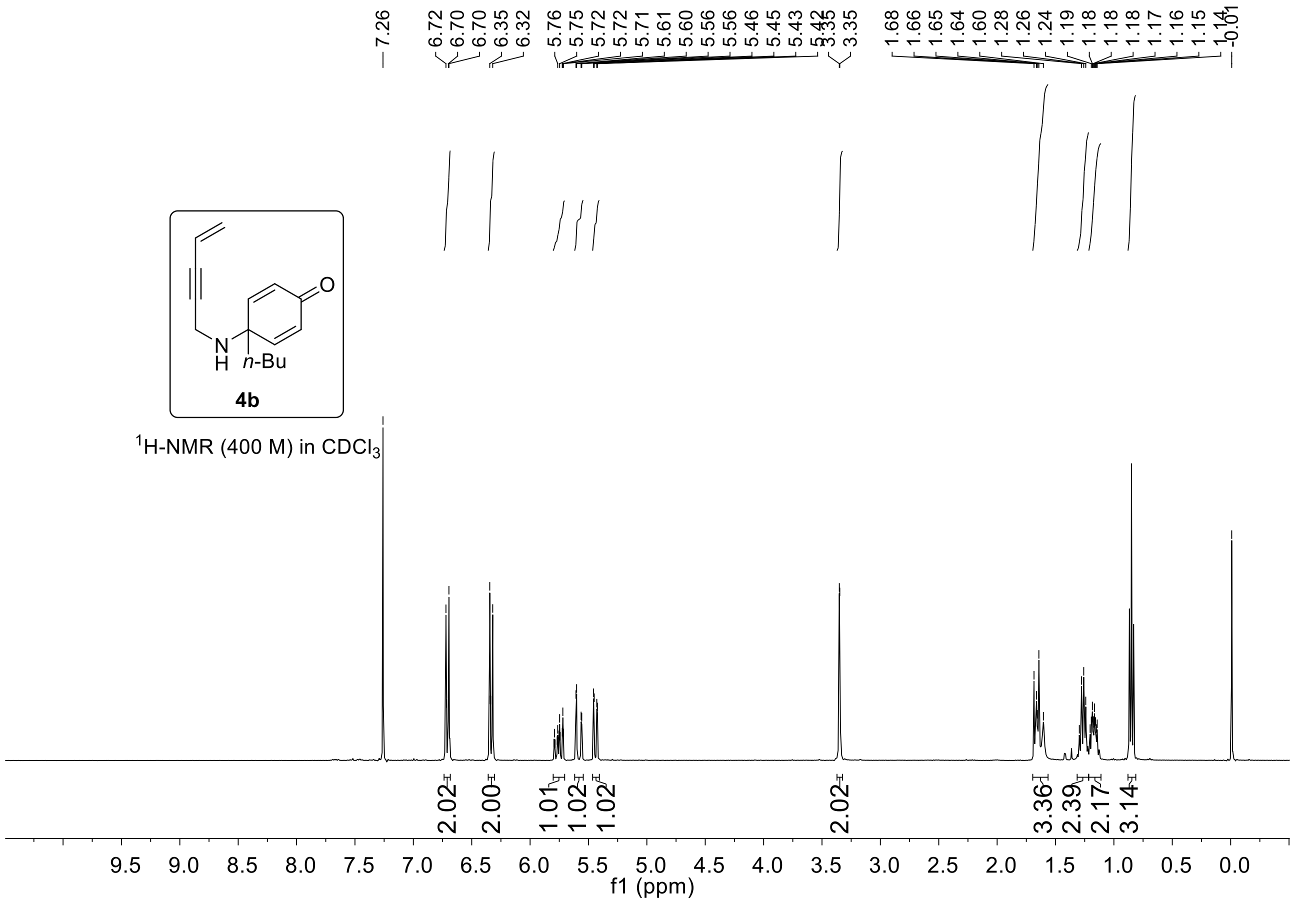

Supplementary Figure 54. ^{1}H NMR spectra for **4b**

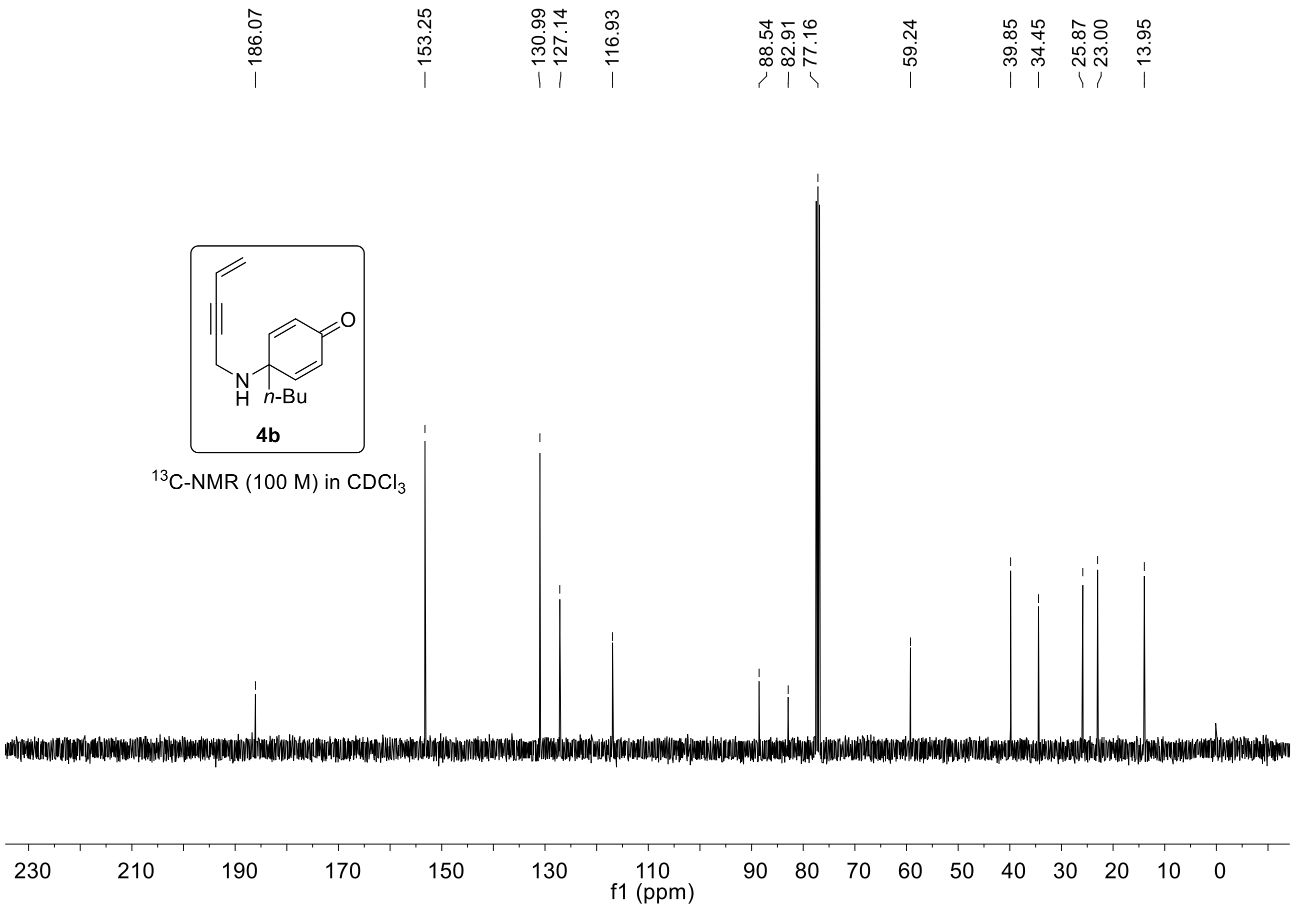

Supplementary Figure 55. ^{13}C NMR spectra for **4b**

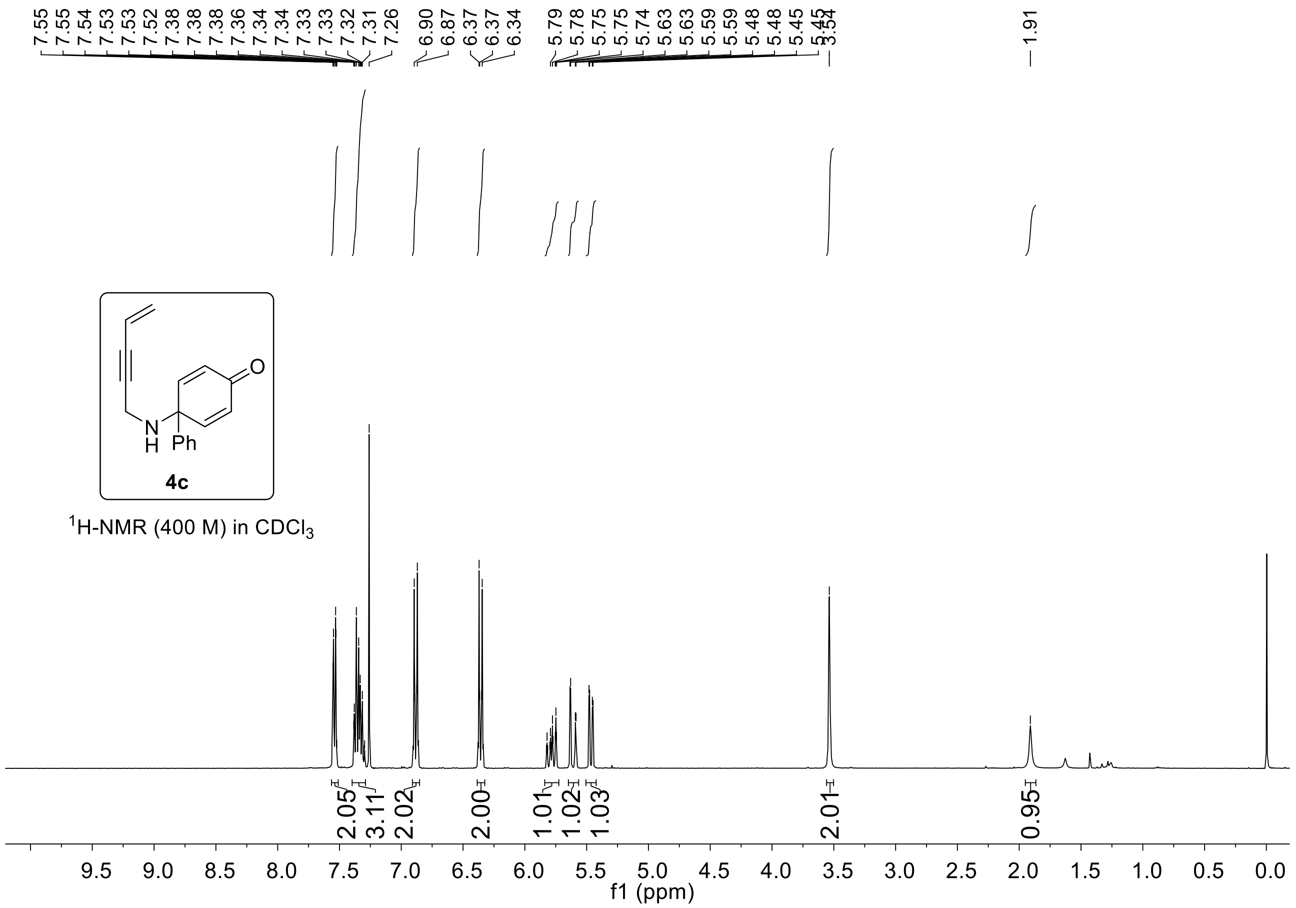

Supplementary Figure 56. ^{1}H NMR spectra for **4c**

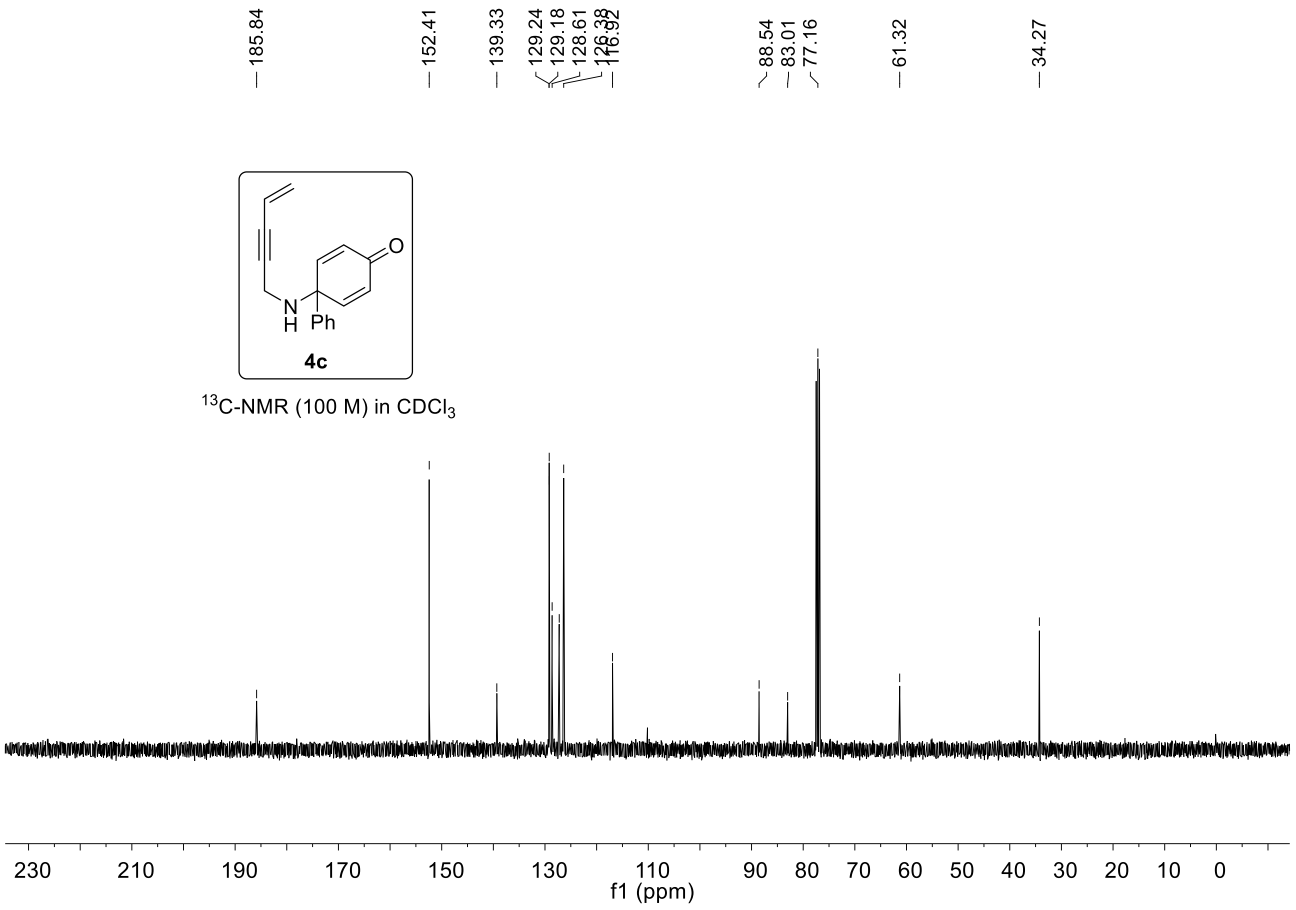

Supplementary Figure 57. ^{13}C NMR spectra for **4c**

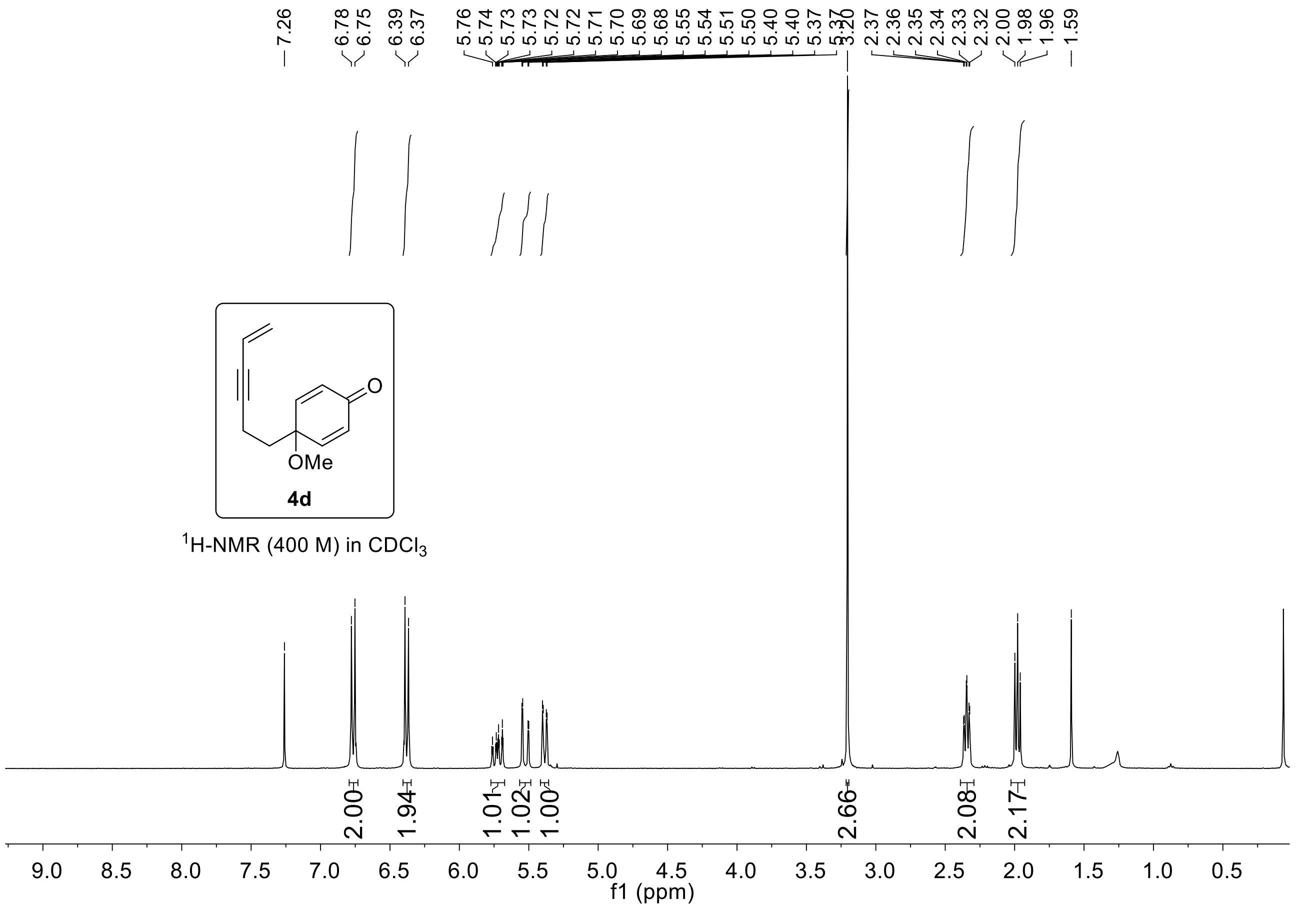

Supplementary Figure 58. ^{1}H NMR spectra for **4d**

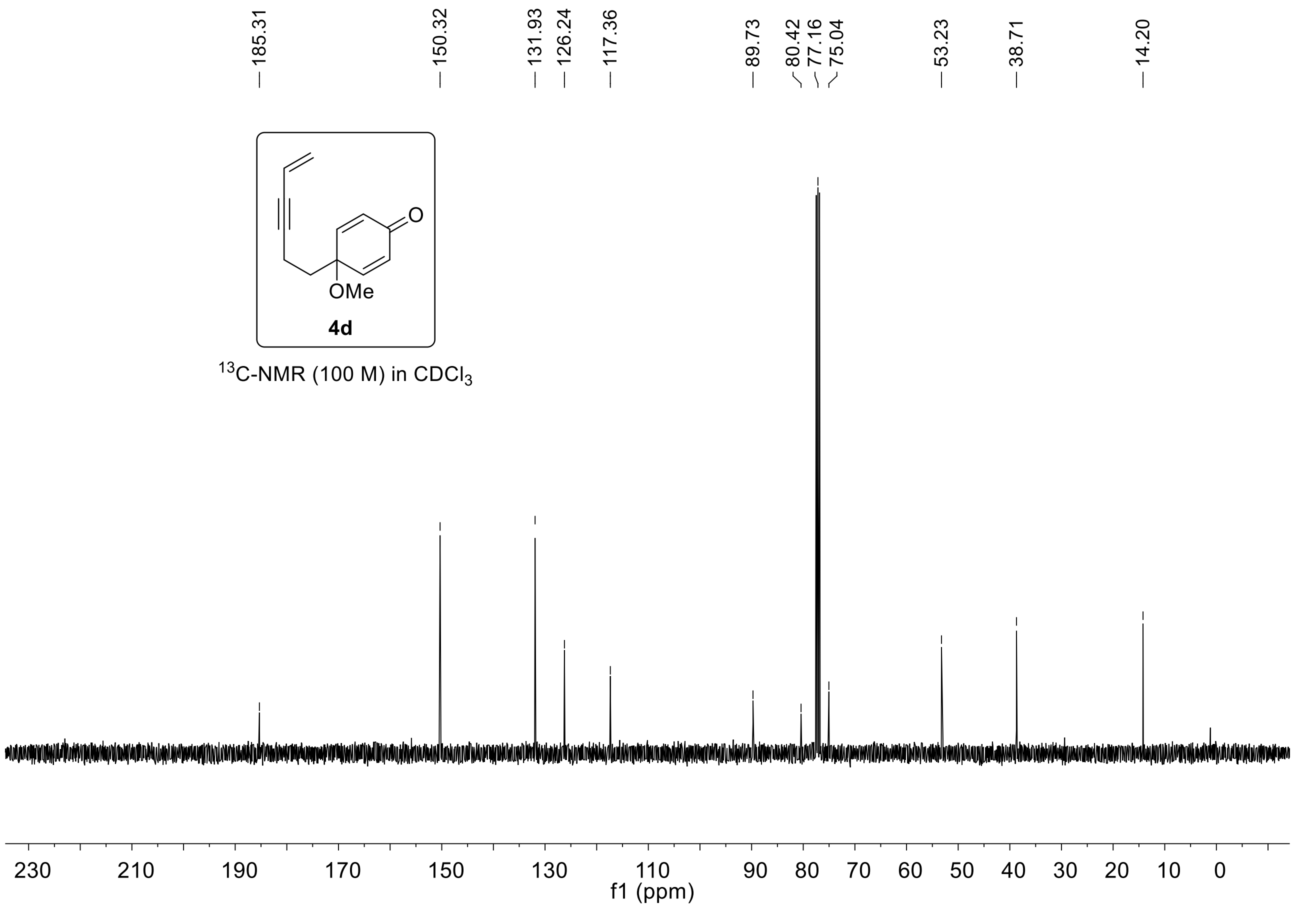

Supplementary Figure 59. ^{13}C NMR spectra for **4d**

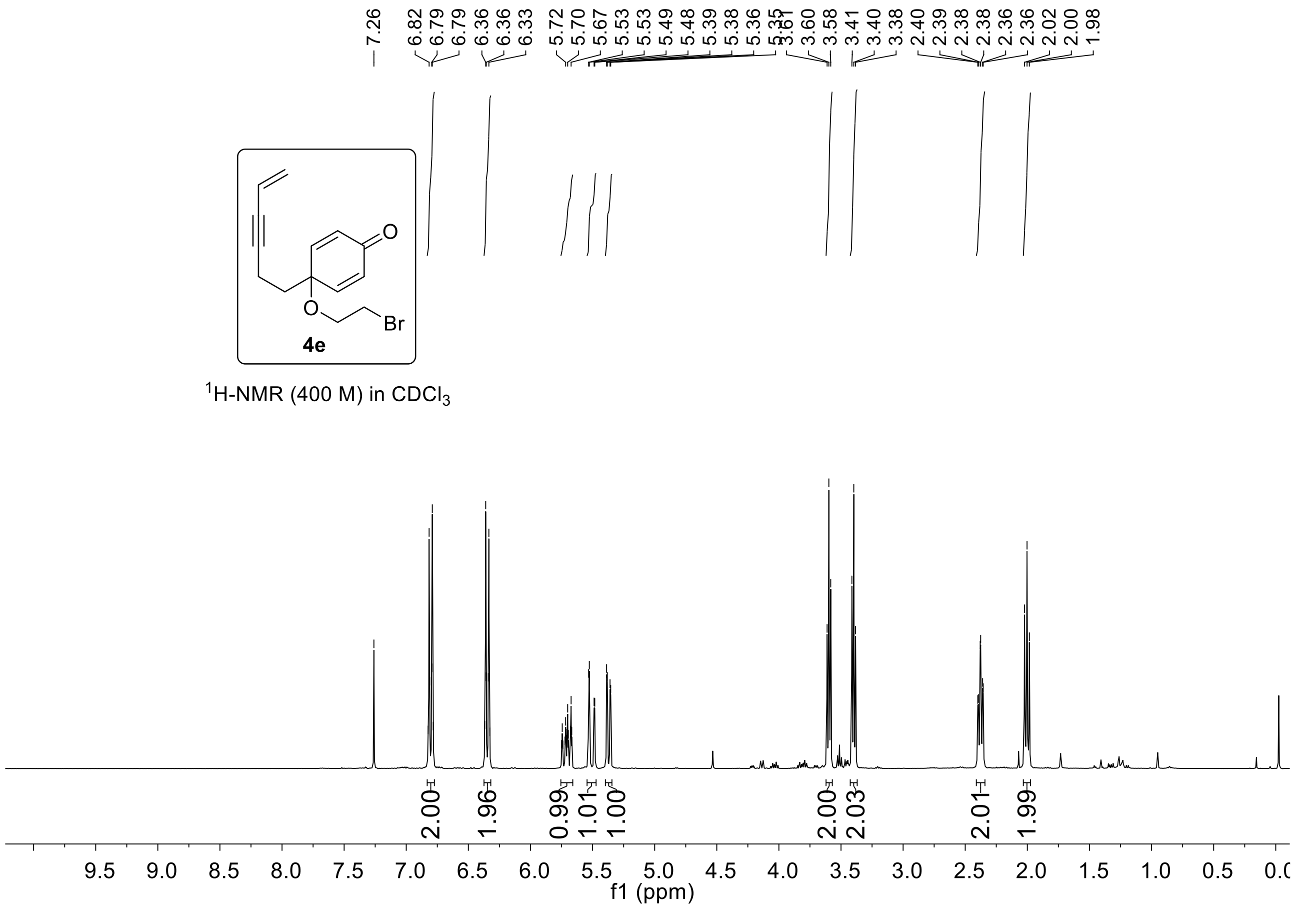

Supplementary Figure 60. ^{1}H NMR spectra for **4e**

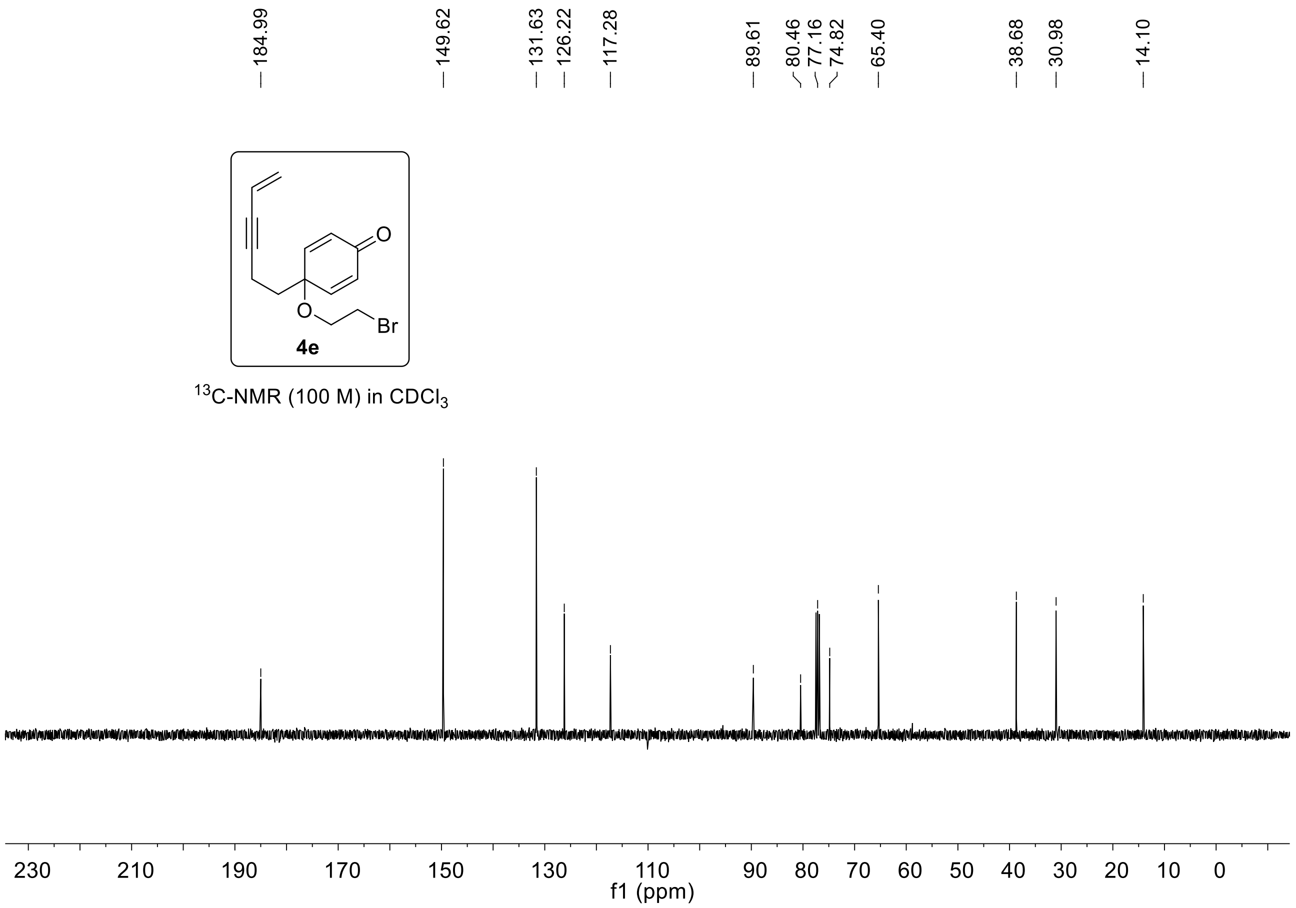

Supplementary Figure 61. ^{13}C NMR spectra for **4e**

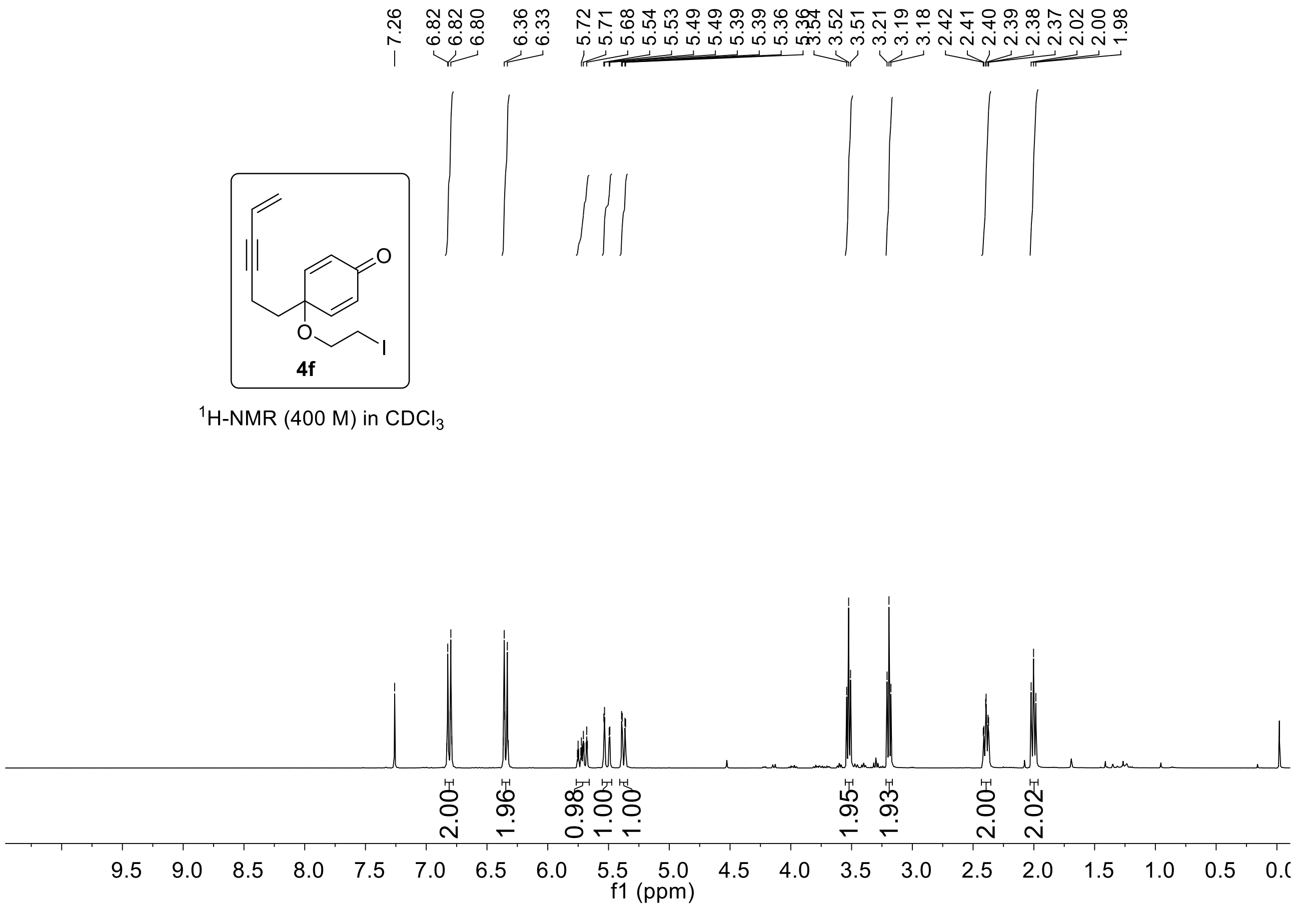

Supplementary Figure 62. ^{1}H NMR spectra for **4f**

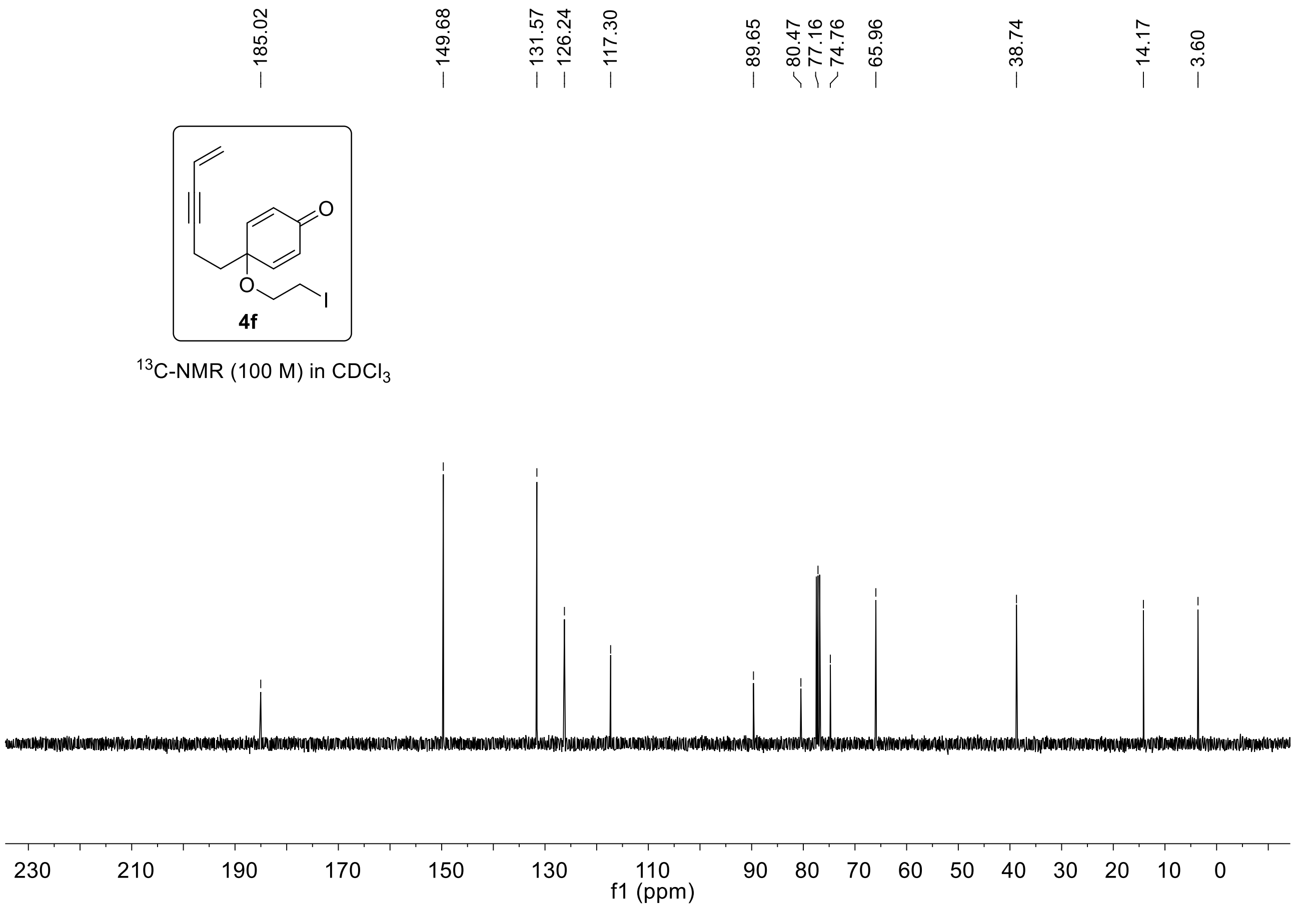

Supplementary Figure 63. ^{13}C NMR spectra for **4f**

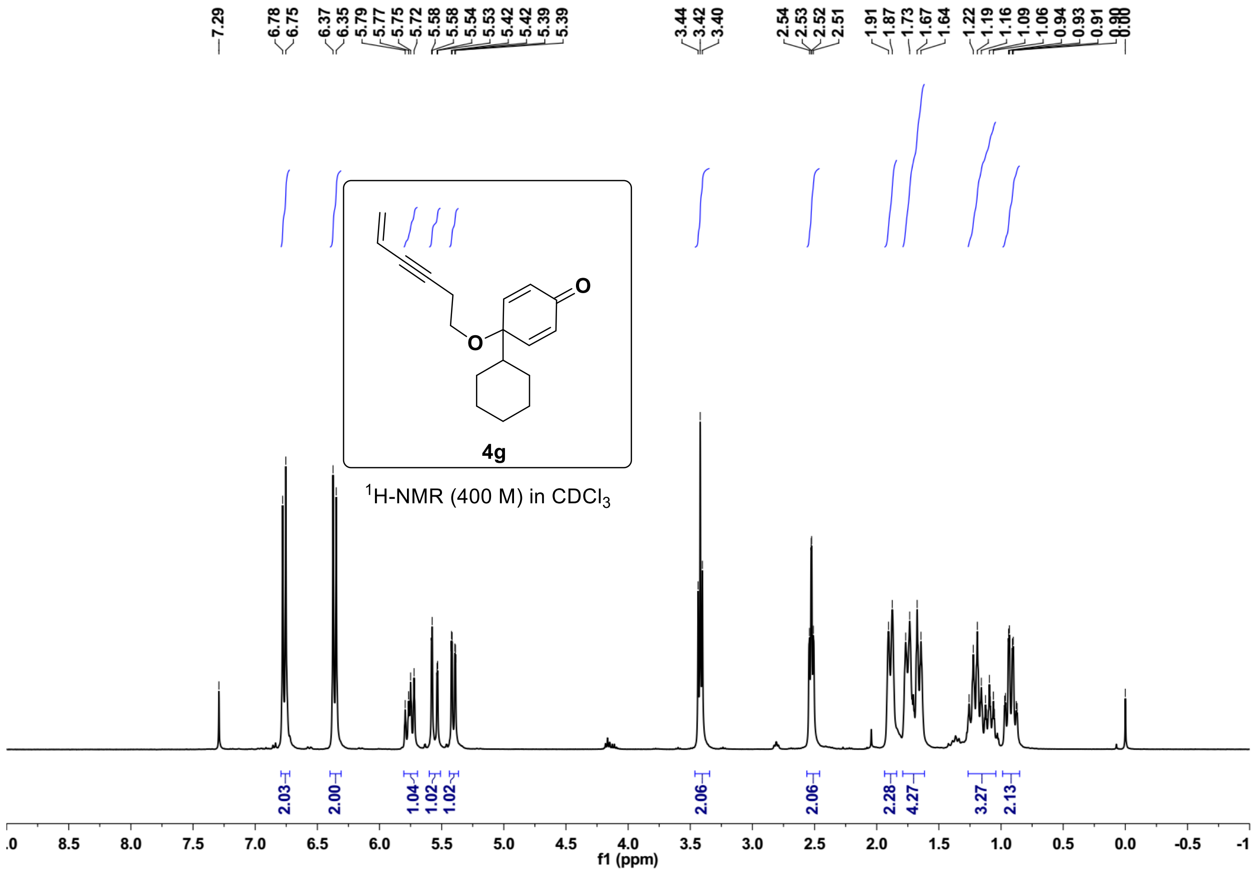

Supplementary Figure 64. ^{1}H NMR spectra for **4g**

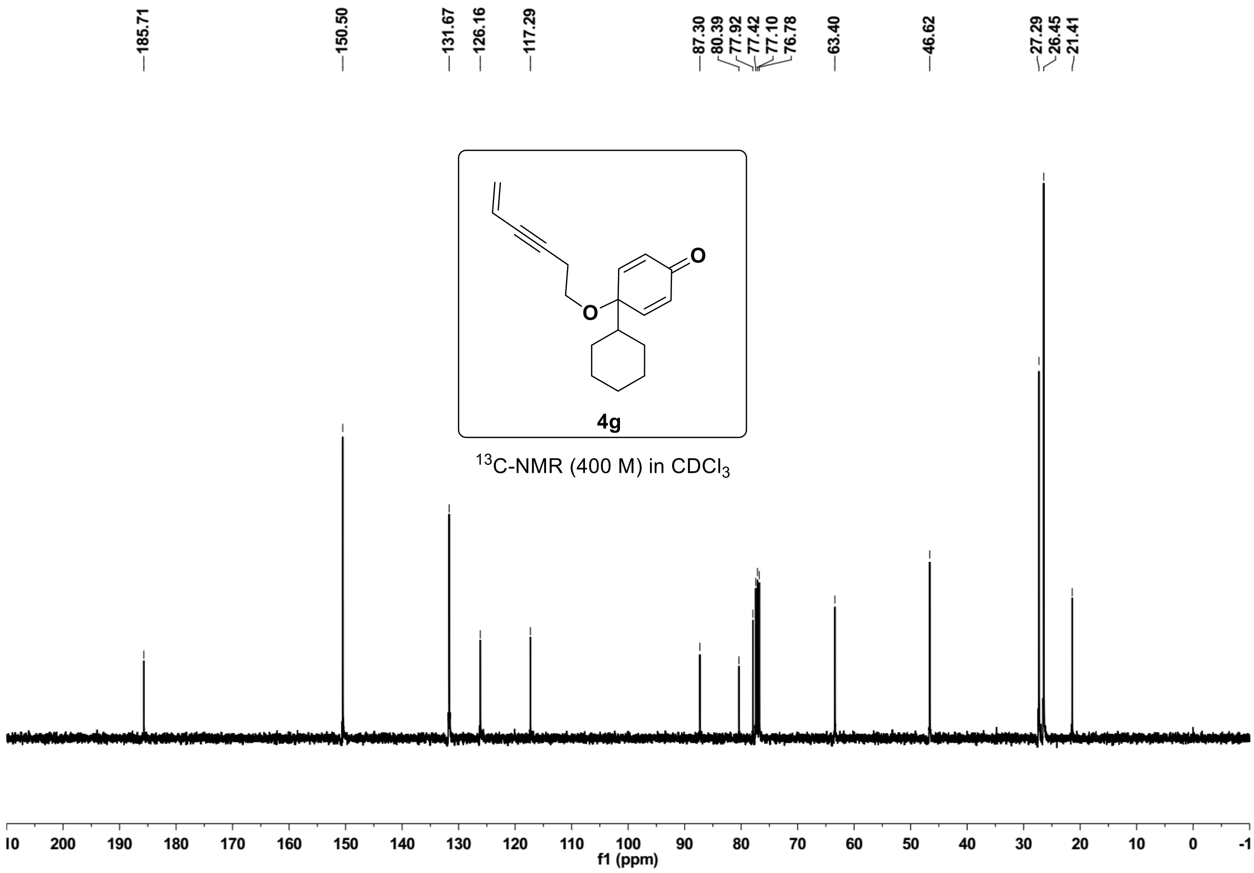

Supplementary Figure 65. ^{13}C NMR spectra for **4g**

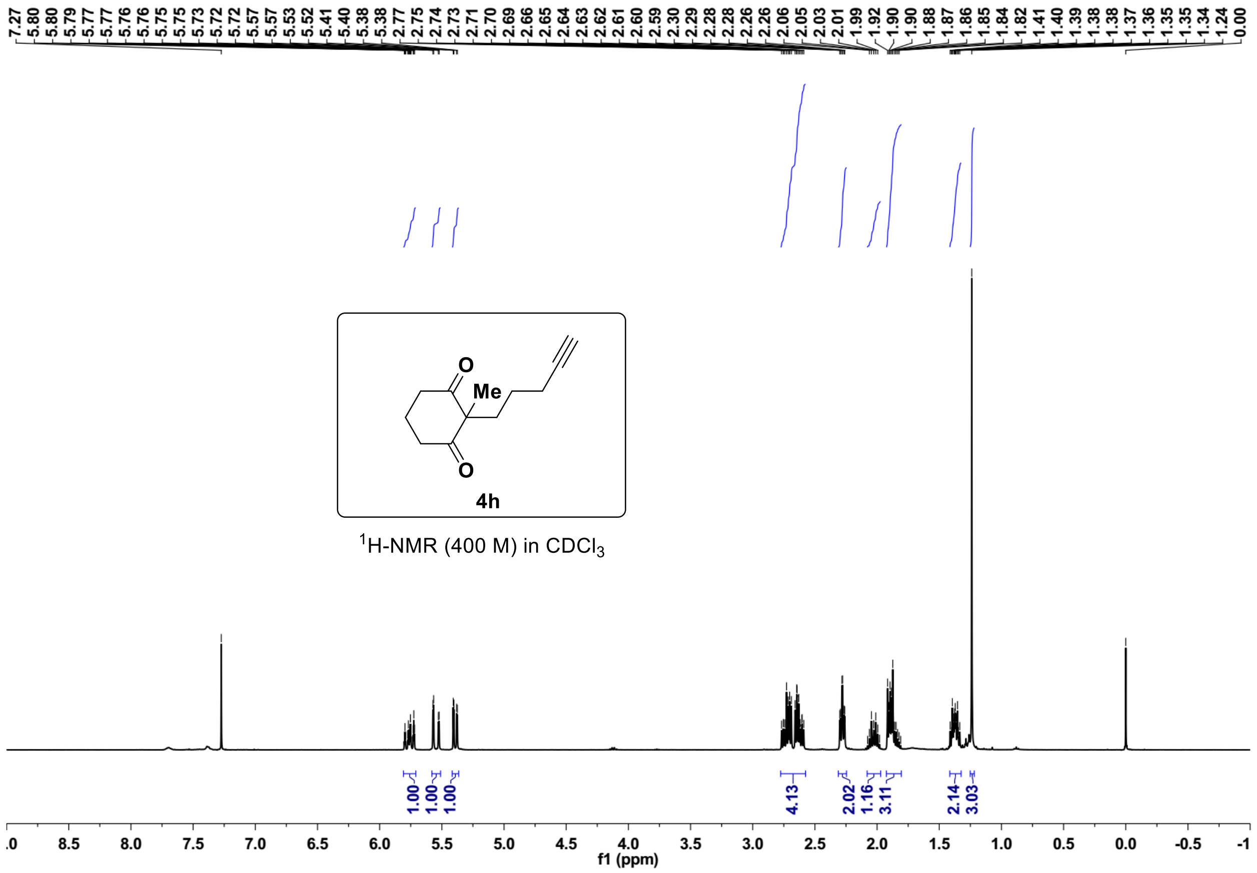

Supplementary Figure 66. ^{1}H NMR spectra for **4h**

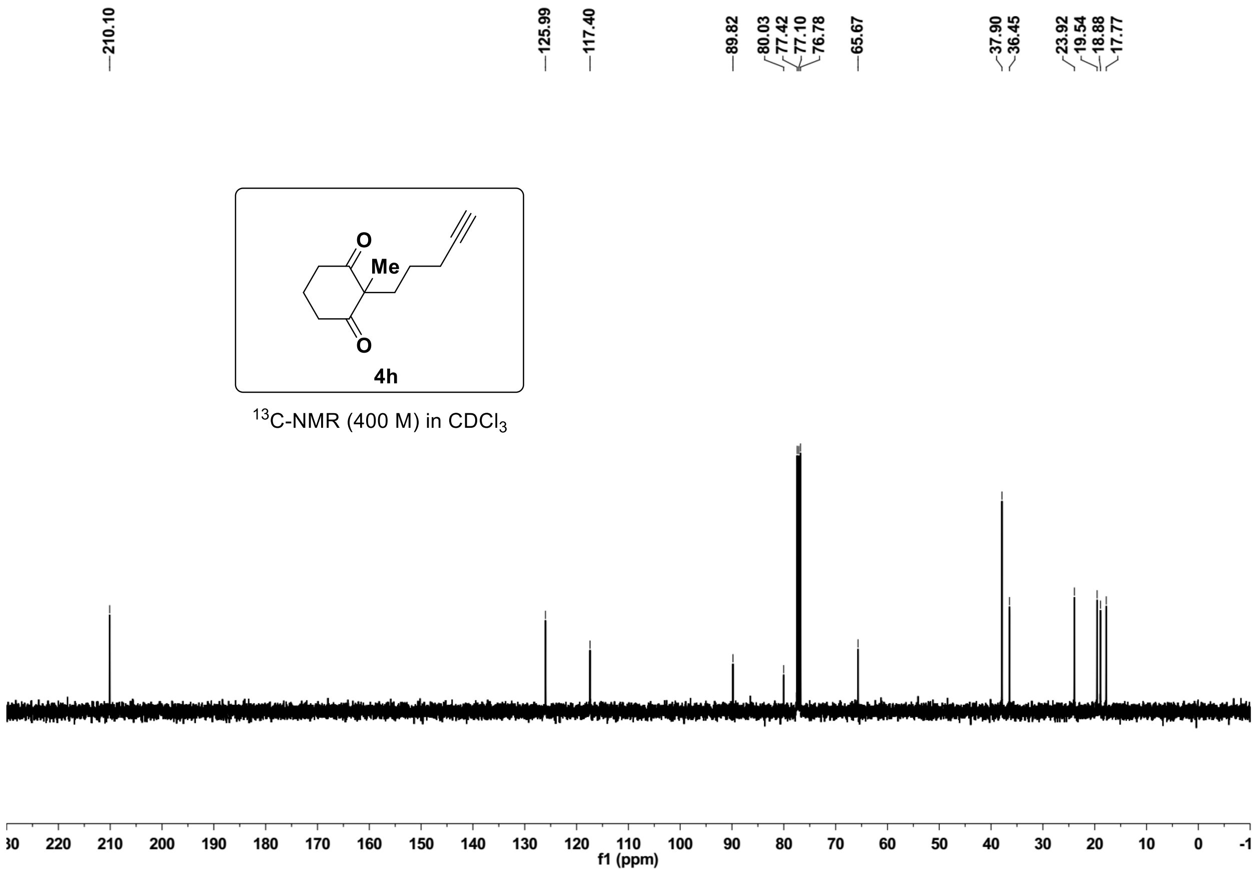

Supplementary Figure 67. ^{13}C NMR spectra for **4h**

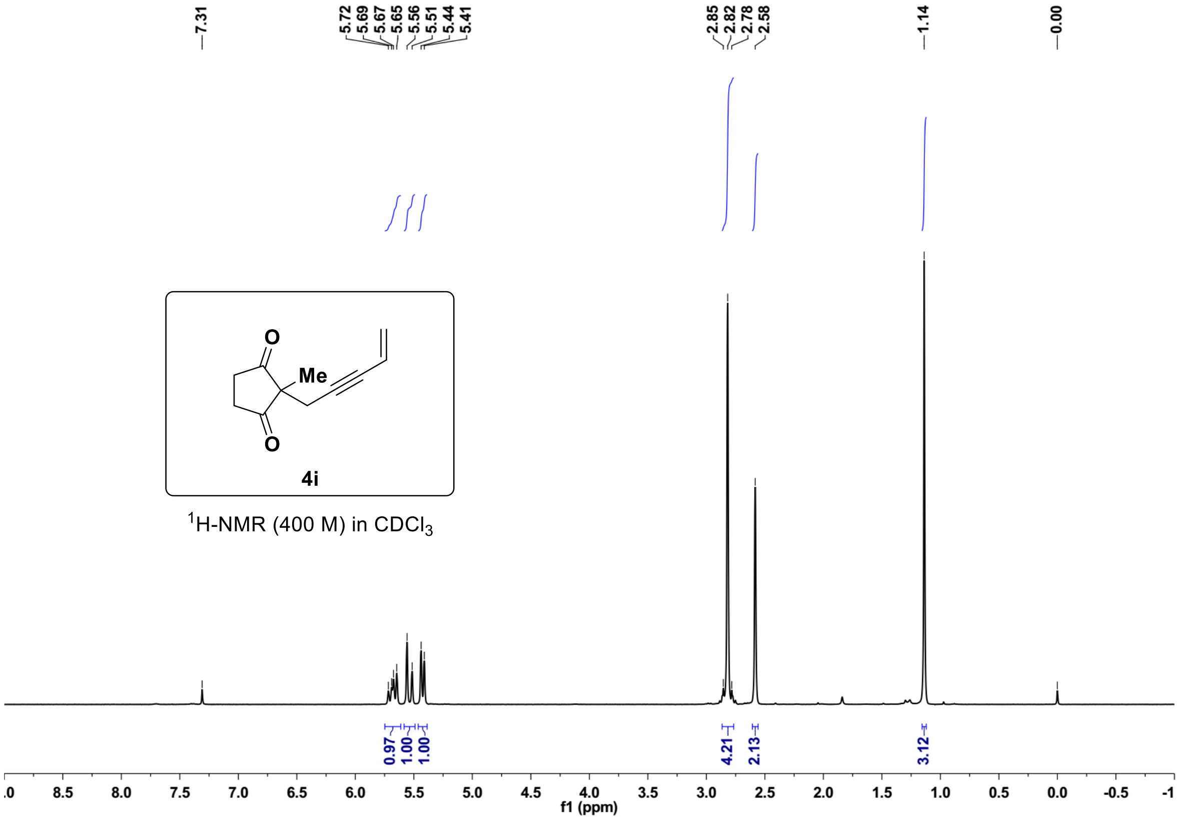

Supplementary Figure 68. ^{1}H NMR spectra for **4i**

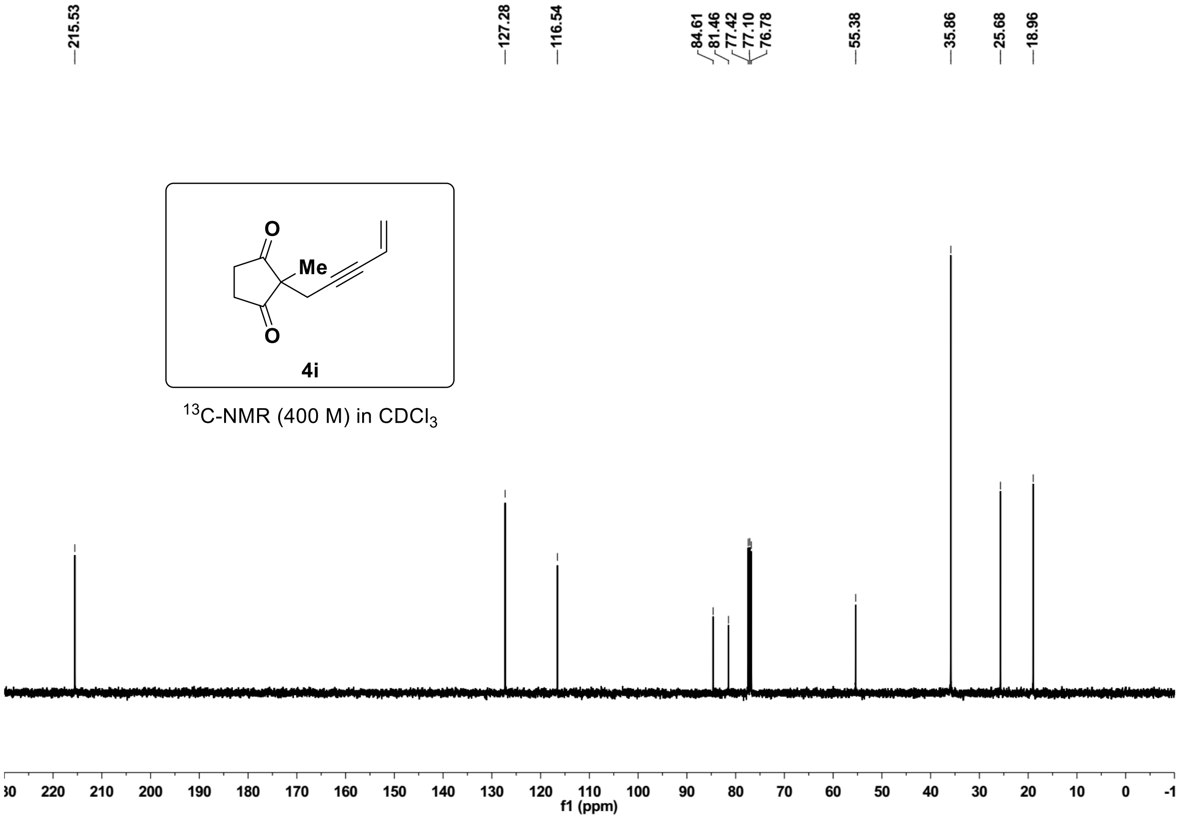

Supplementary Figure 69. ^{13}C NMR spectra for **4i**

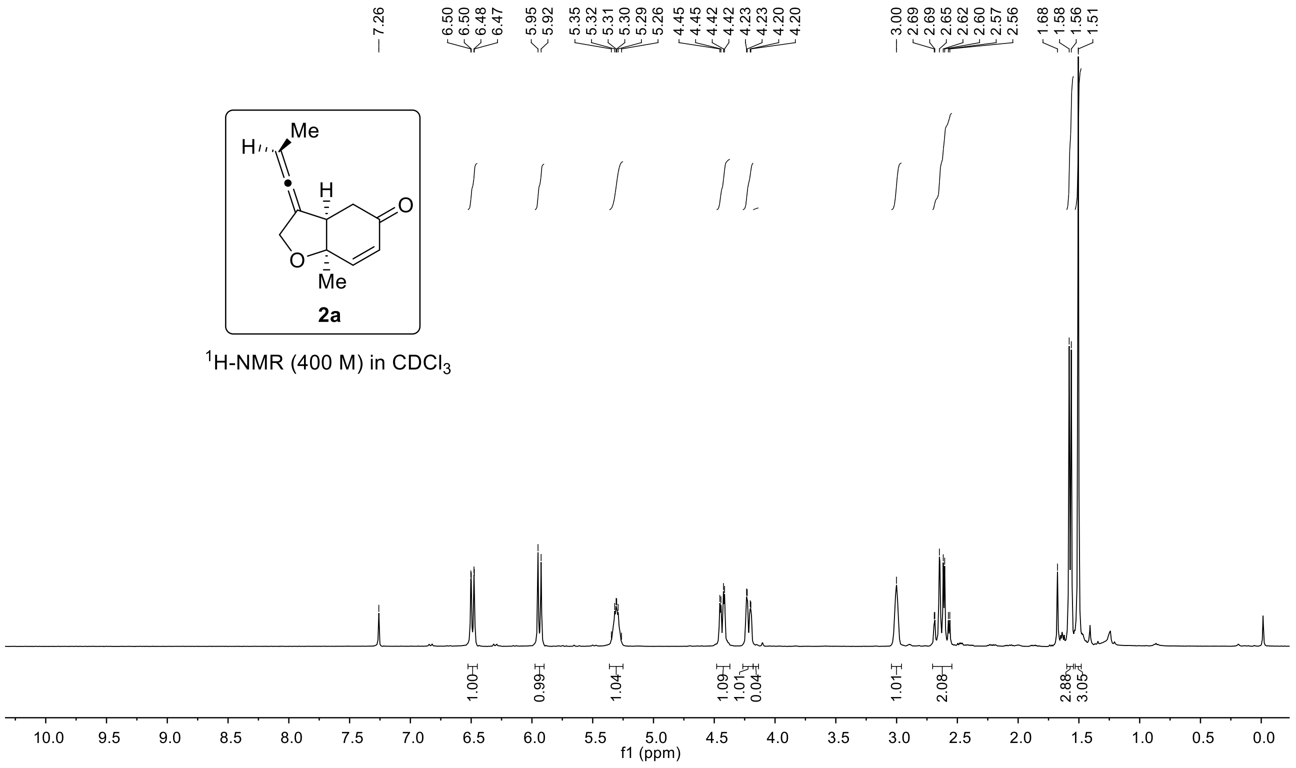

Supplementary Figure 70. ^{1}H NMR spectra for **2a**

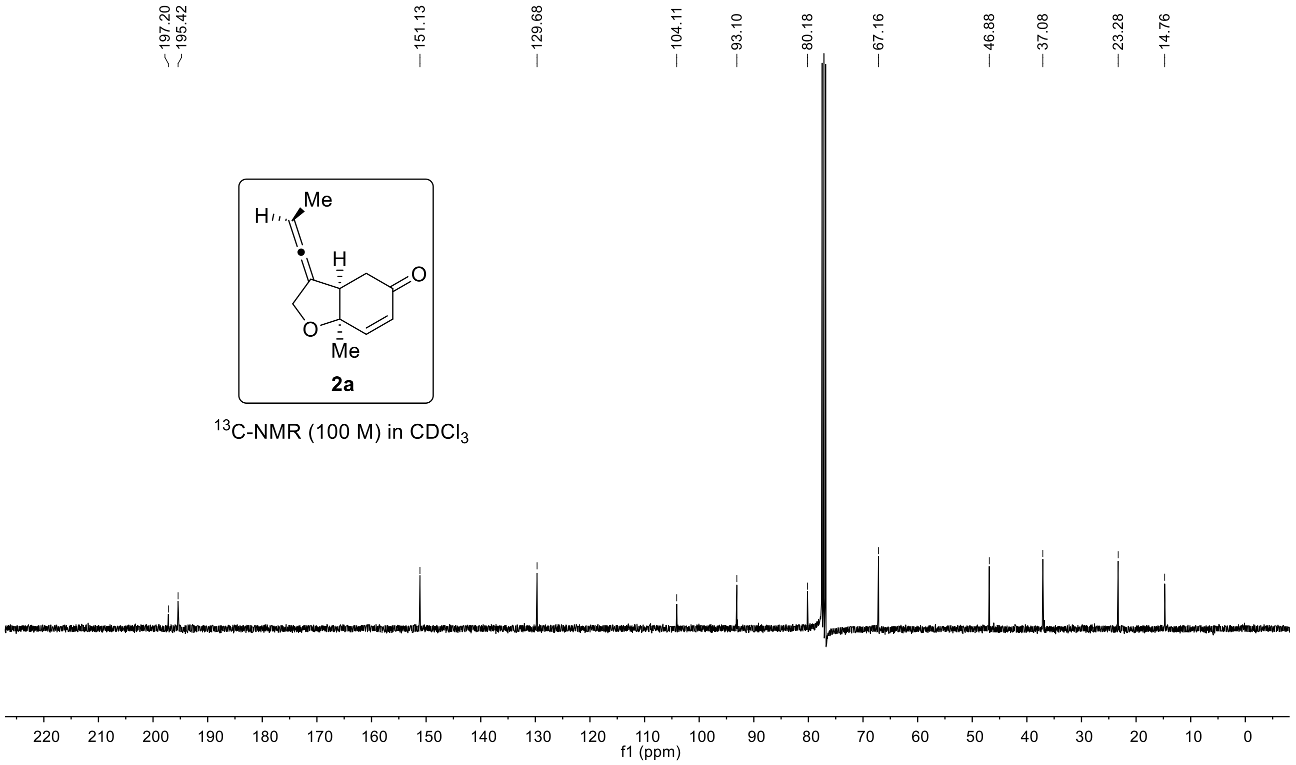

Supplementary Figure 71. ^{13}C NMR spectra for **2a**

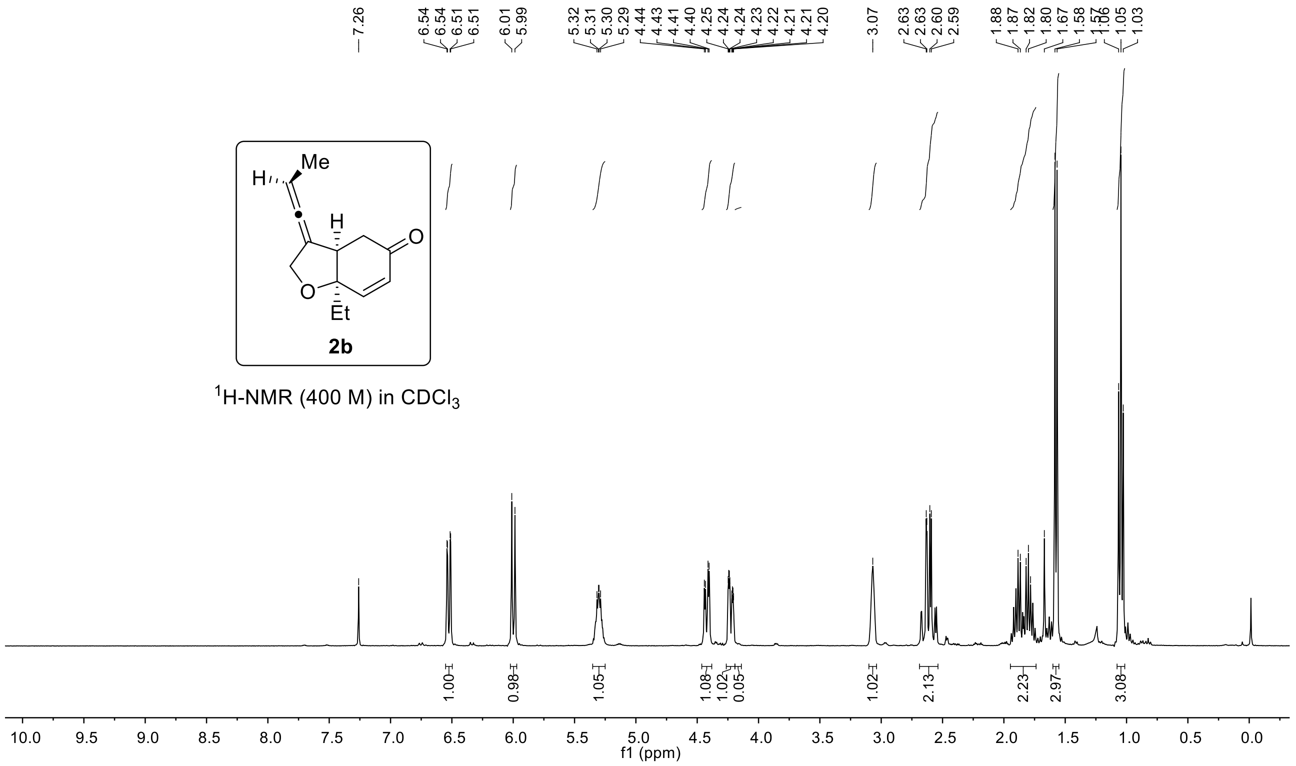

Supplementary Figure 72. ^{1}H NMR spectra for **2b**

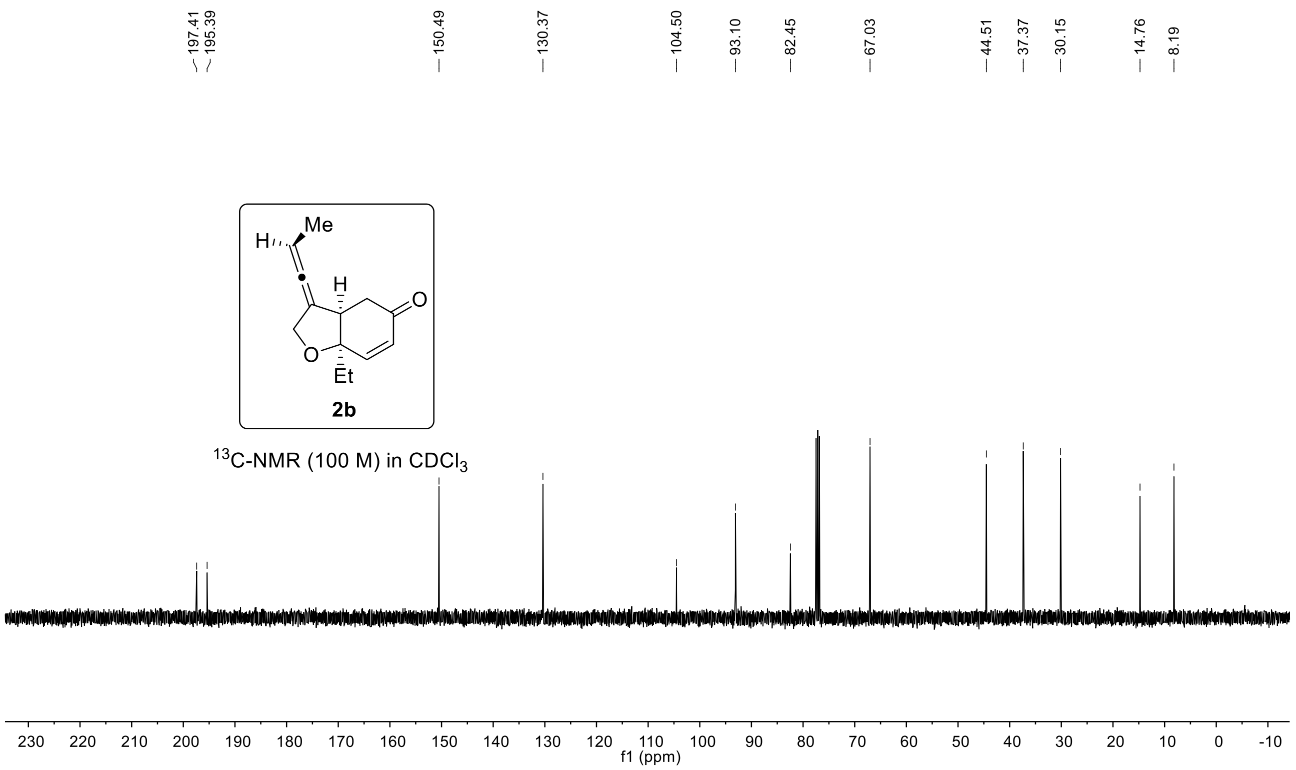

Supplementary Figure 73. ^{13}C NMR spectra for **2b**

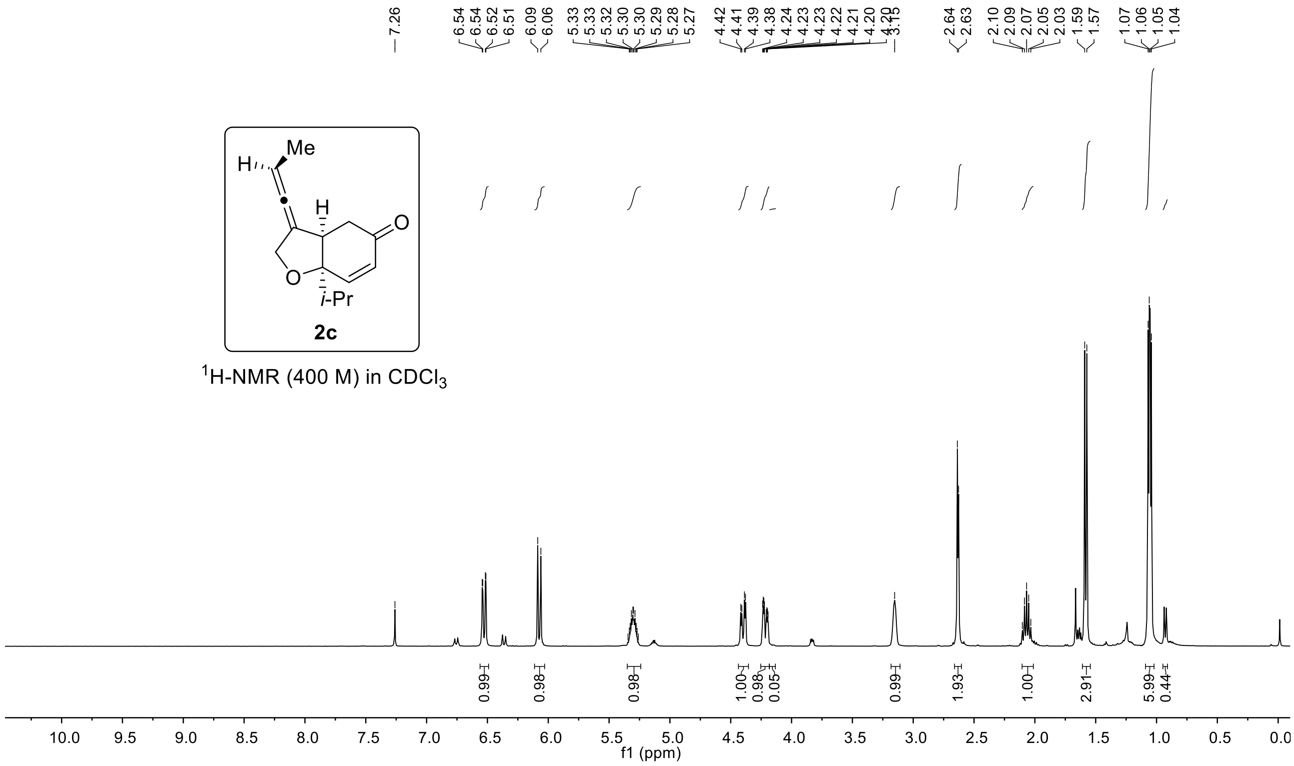

Supplementary Figure 74. ^{1}H NMR spectra for **2c**

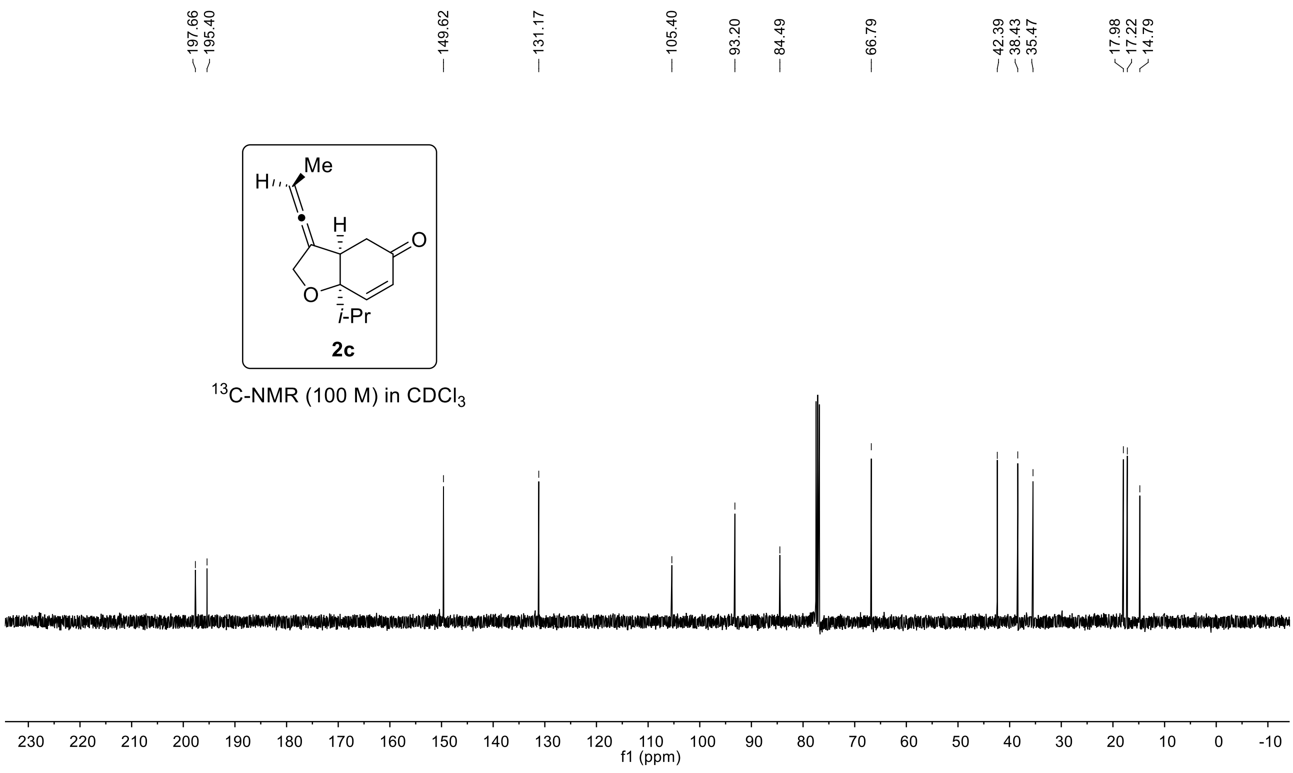

Supplementary Figure 75. ^{13}C NMR spectra for **2c**

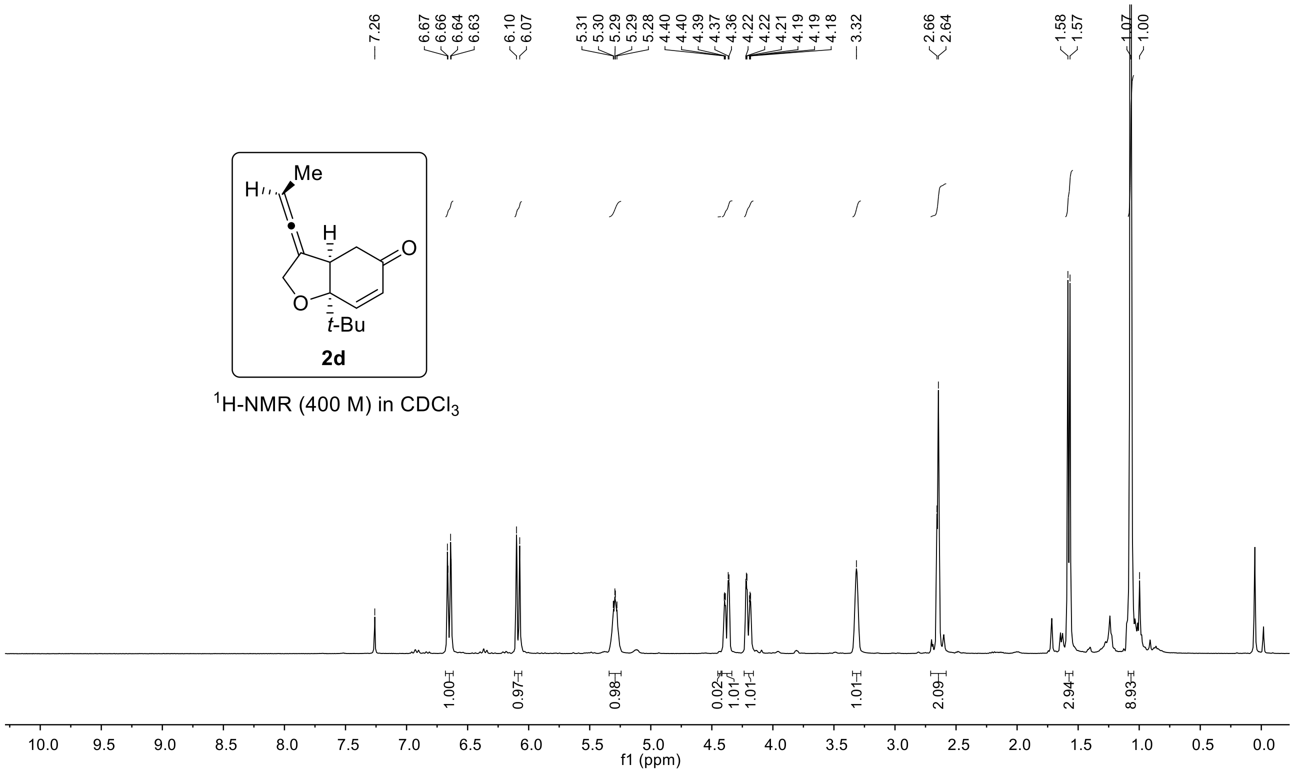

Supplementary Figure 76. ^{1}H NMR spectra for **2d**

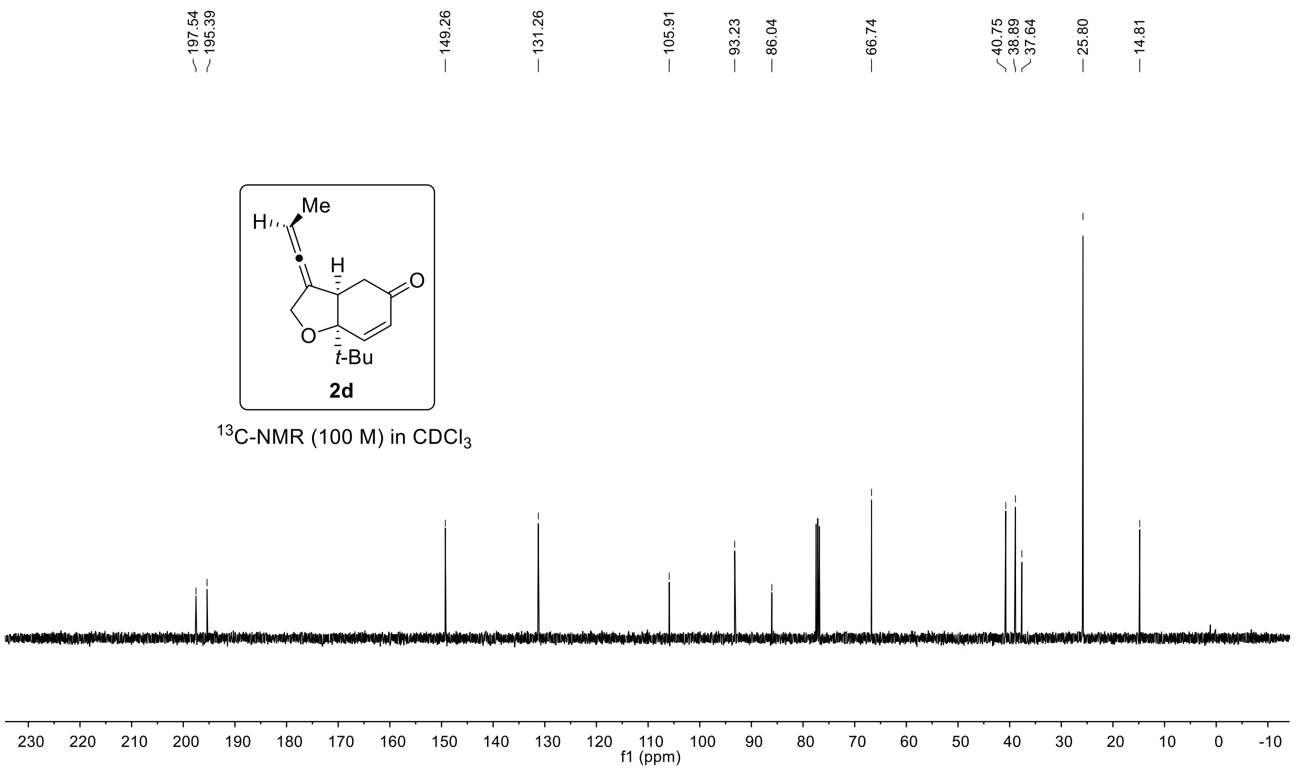

Supplementary Figure 77. ^{13}C NMR spectra for **2d**

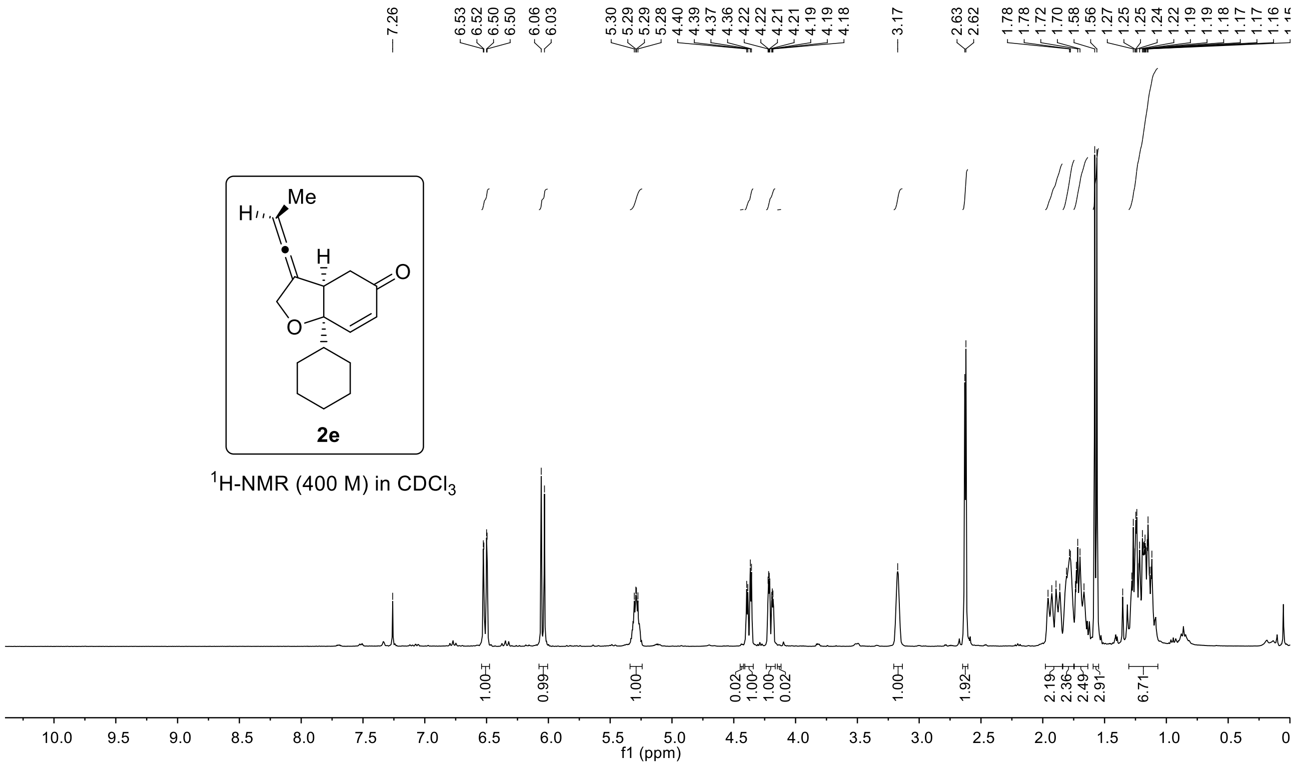

Supplementary Figure 78. ^{1}H NMR spectra for **2e**

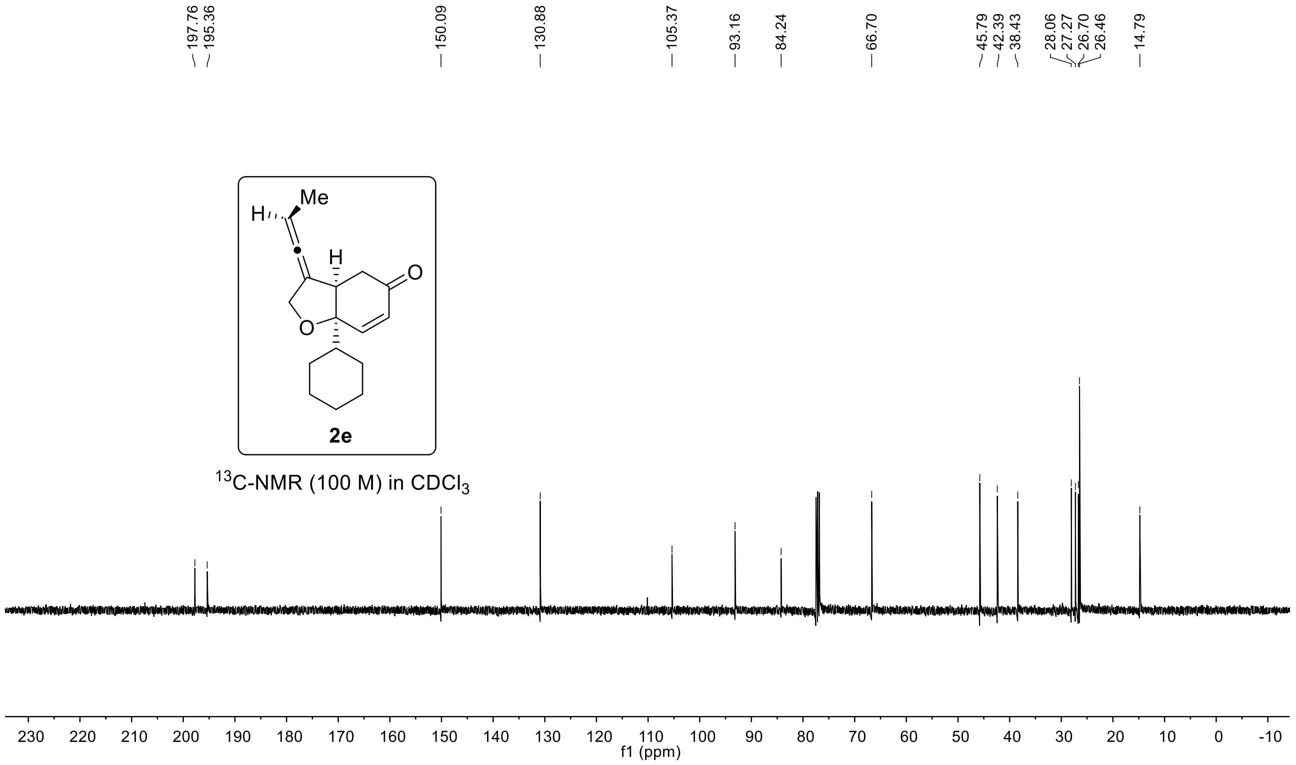

Supplementary Figure 79. ^{13}C NMR spectra for **2e**

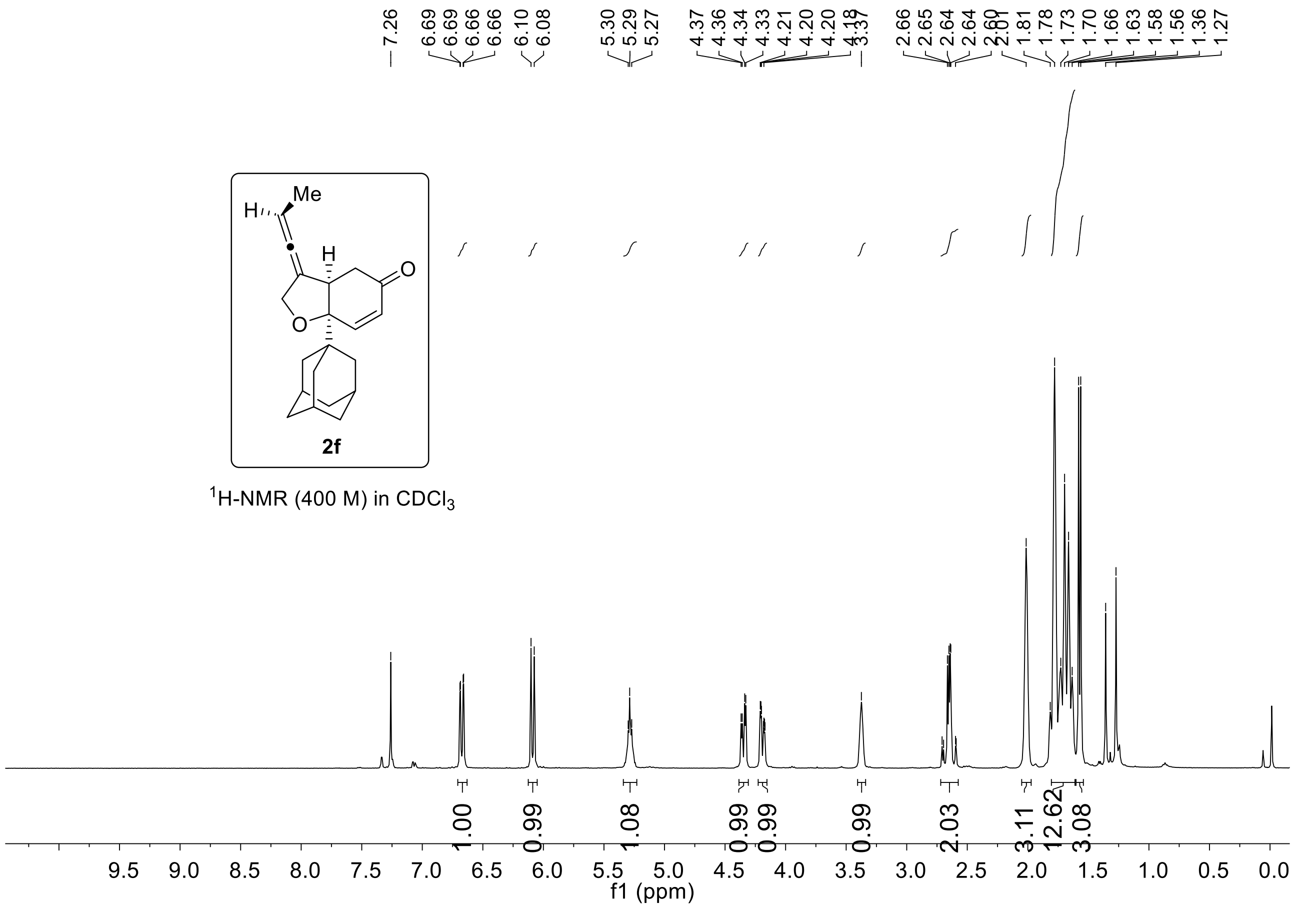

Supplementary Figure 80. ^{1}H NMR spectra for **2f**

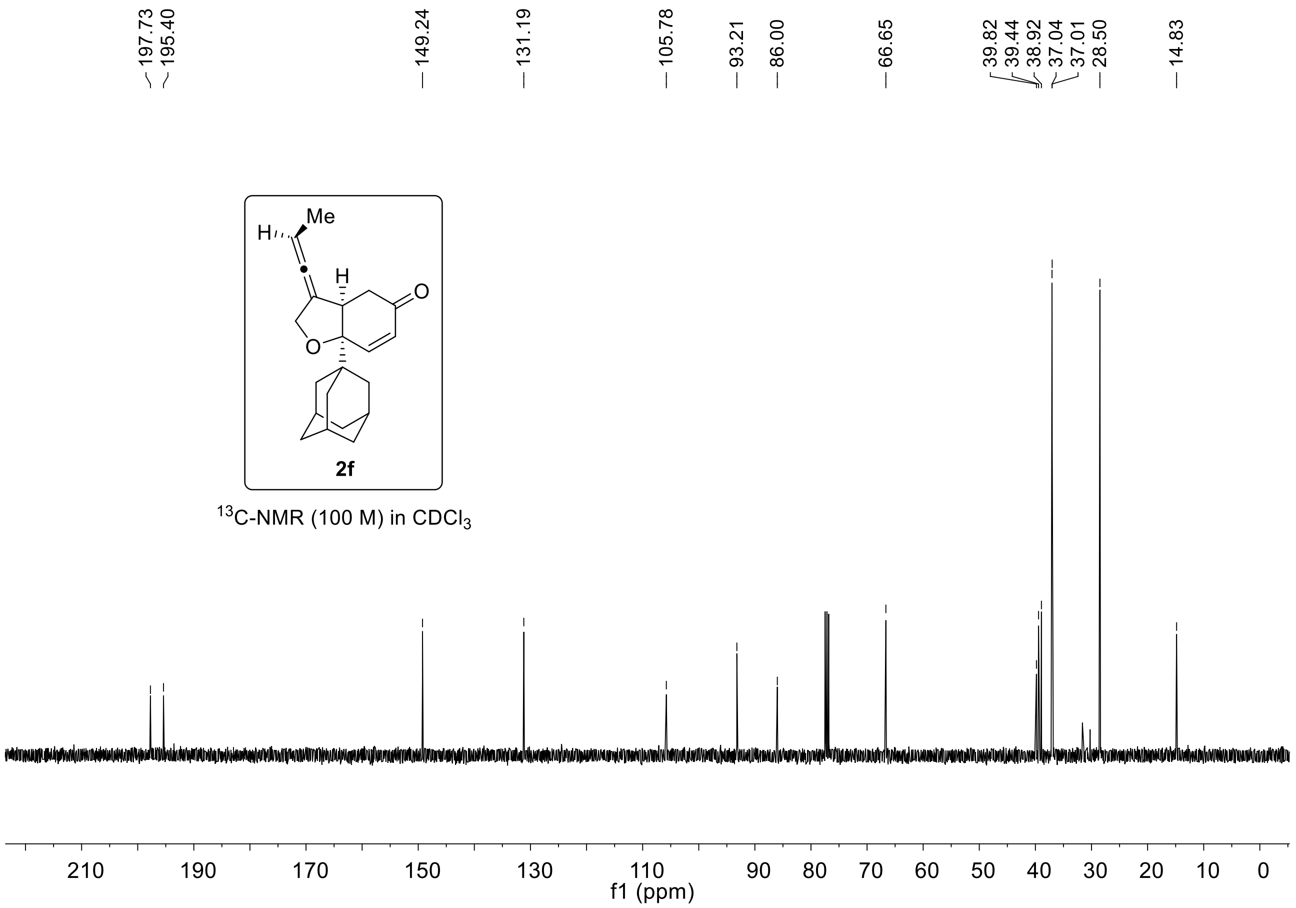

Supplementary Figure 81. ^{13}C NMR spectra for **2f**

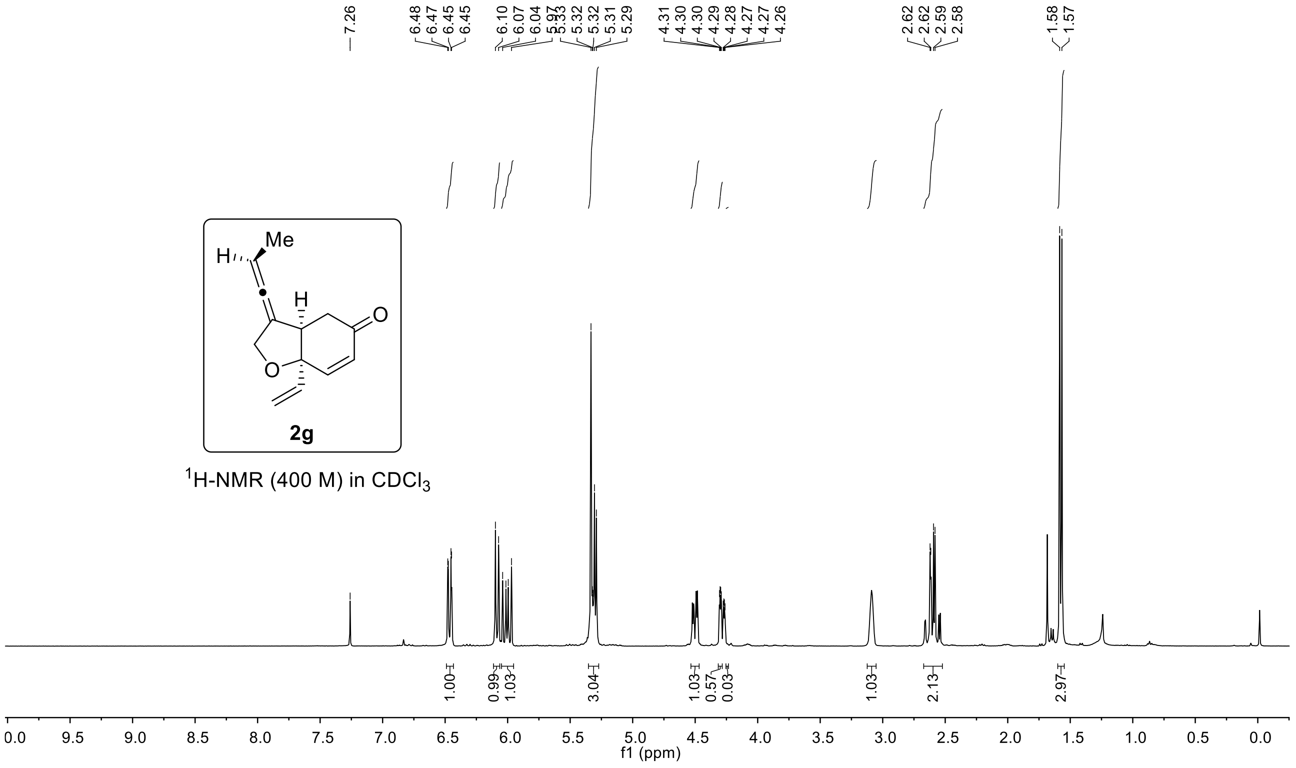

Supplementary Figure 82. ^{1}H NMR spectra for **2g**

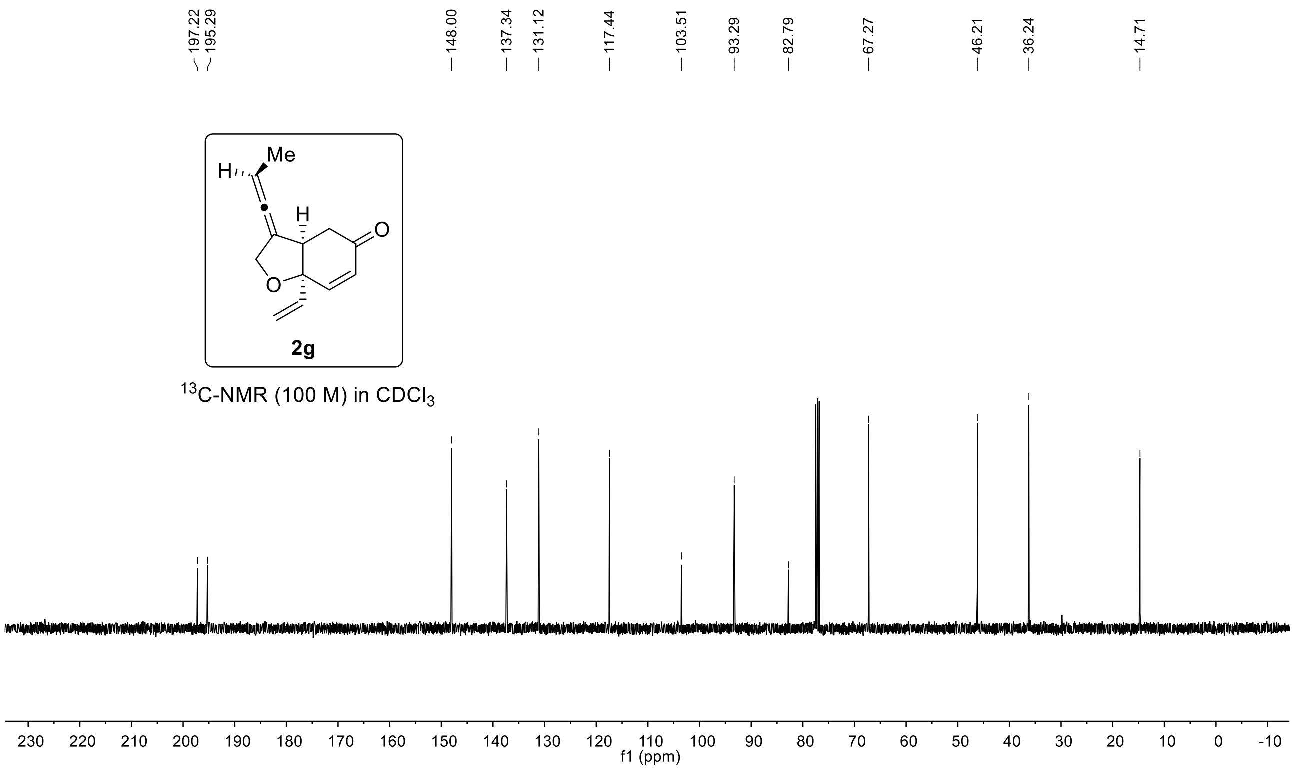

Supplementary Figure 83. ^{13}C NMR spectra for **2g**

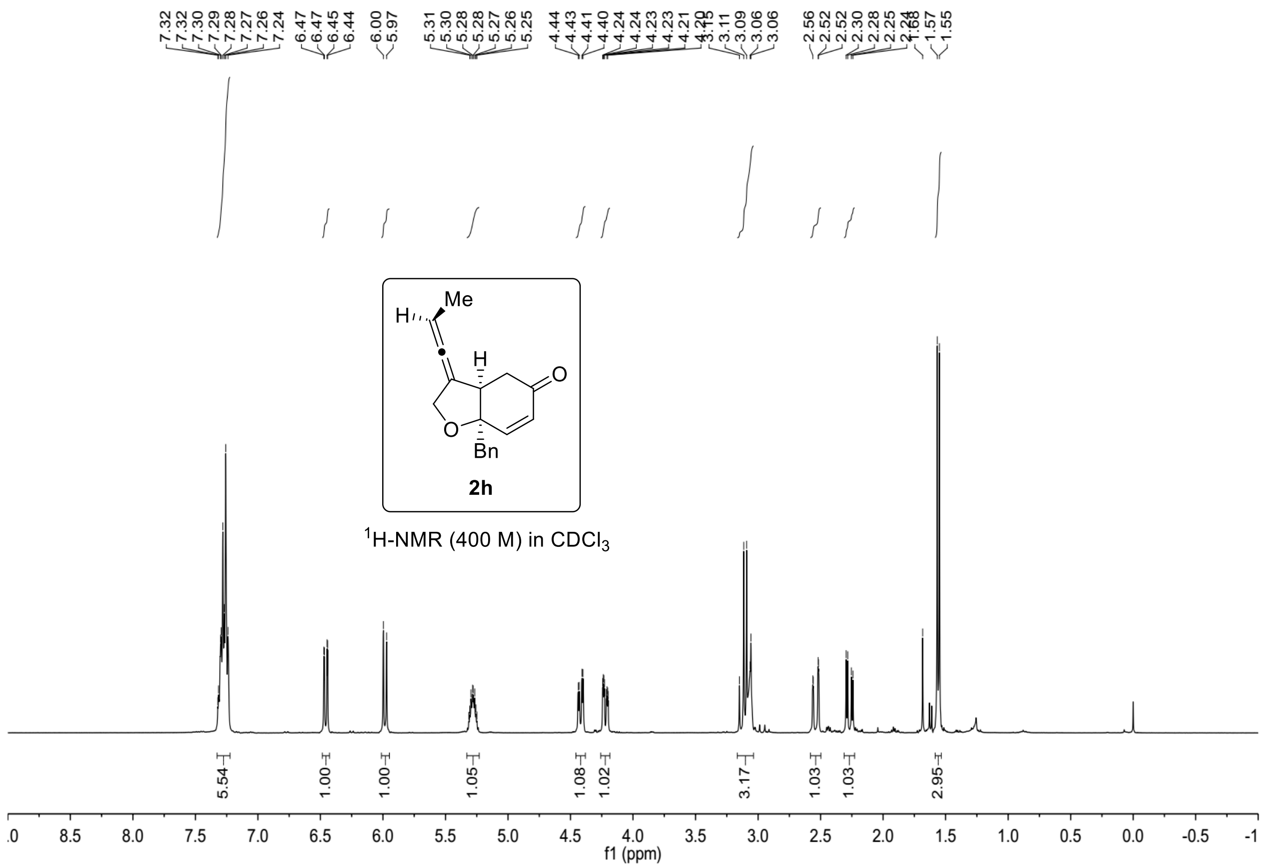

Supplementary Figure 84. ^{1}H NMR spectra for **2h**

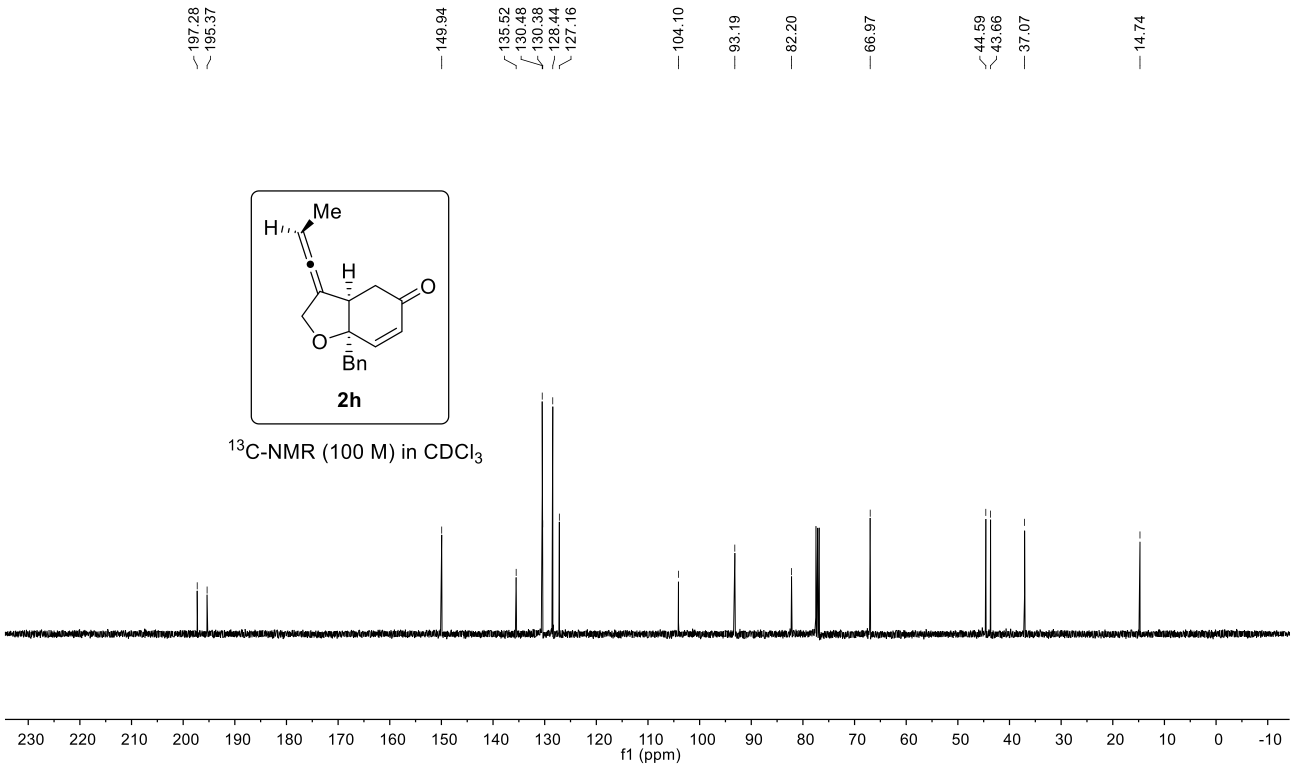

Supplementary Figure 85. ^{13}C NMR spectra for **2h**

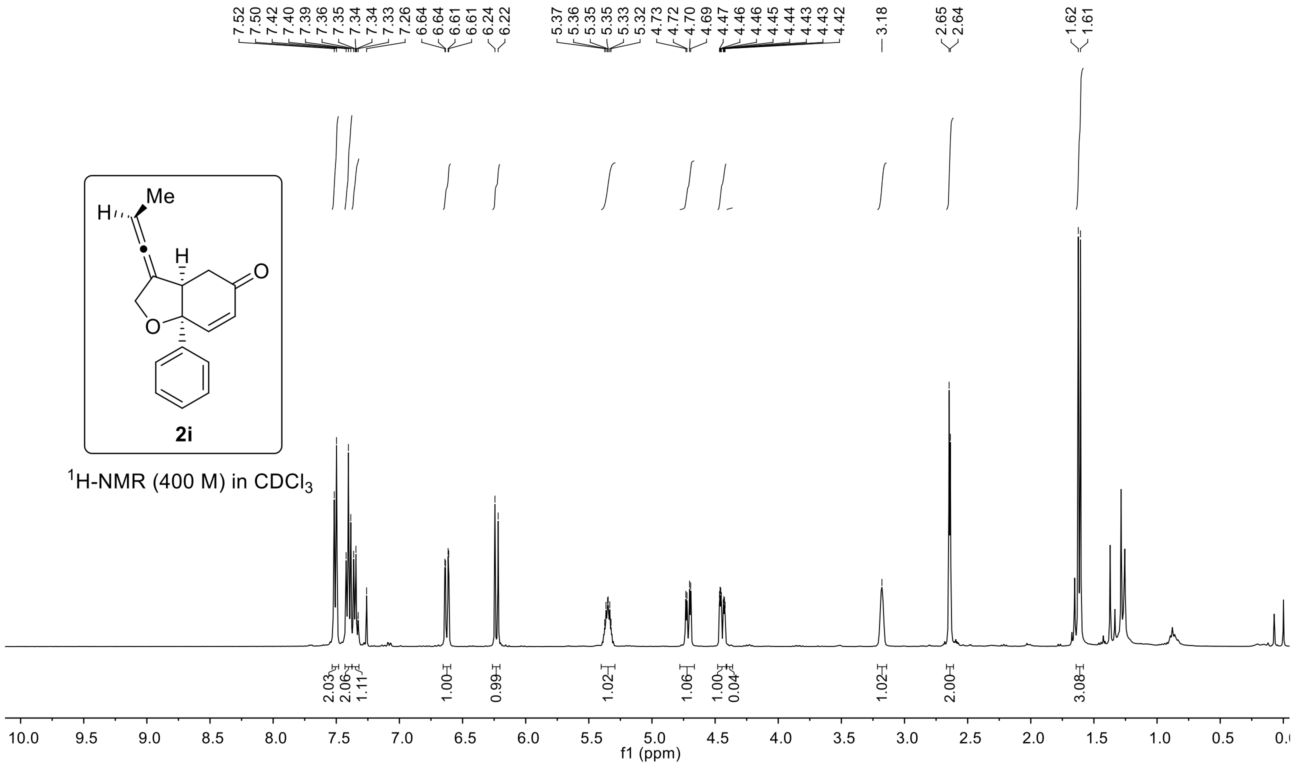

Supplementary Figure 86. ^{1}H NMR spectra for **2i**

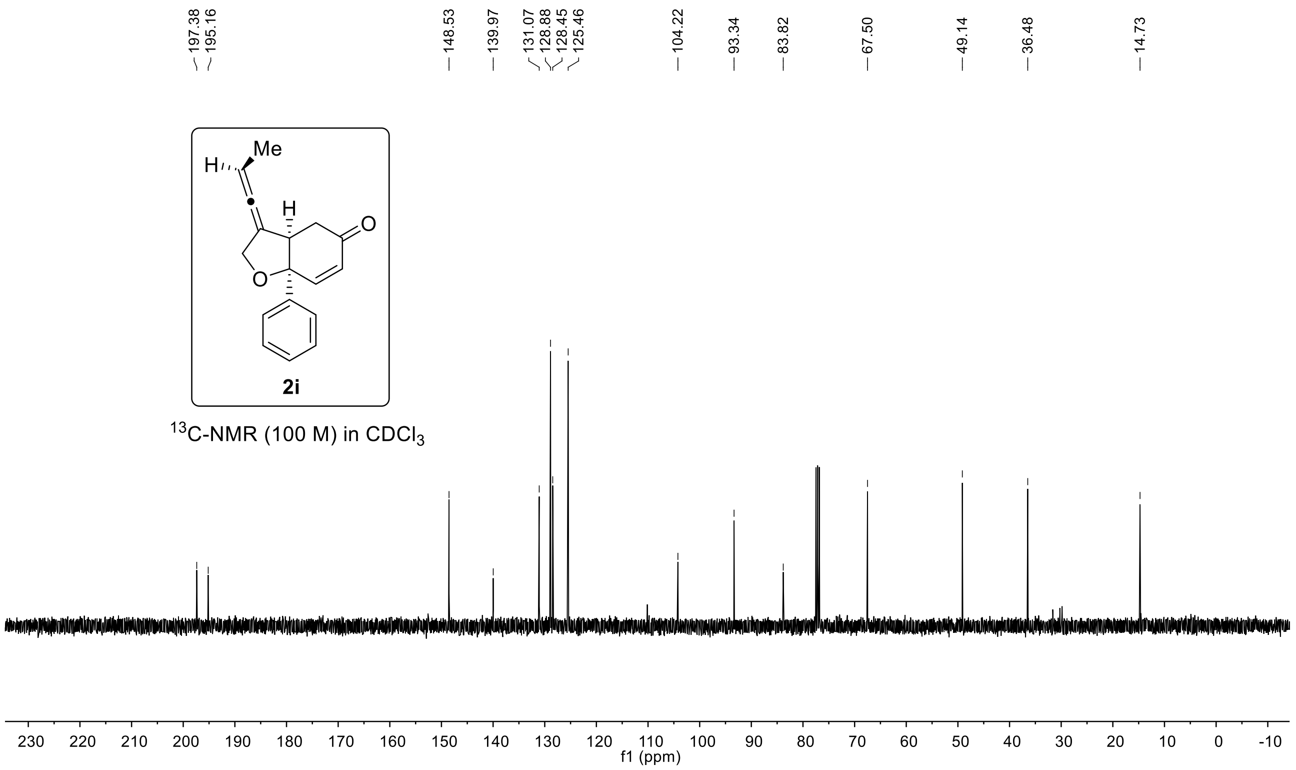

Supplementary Figure 87. ^{13}C NMR spectra for **2i**

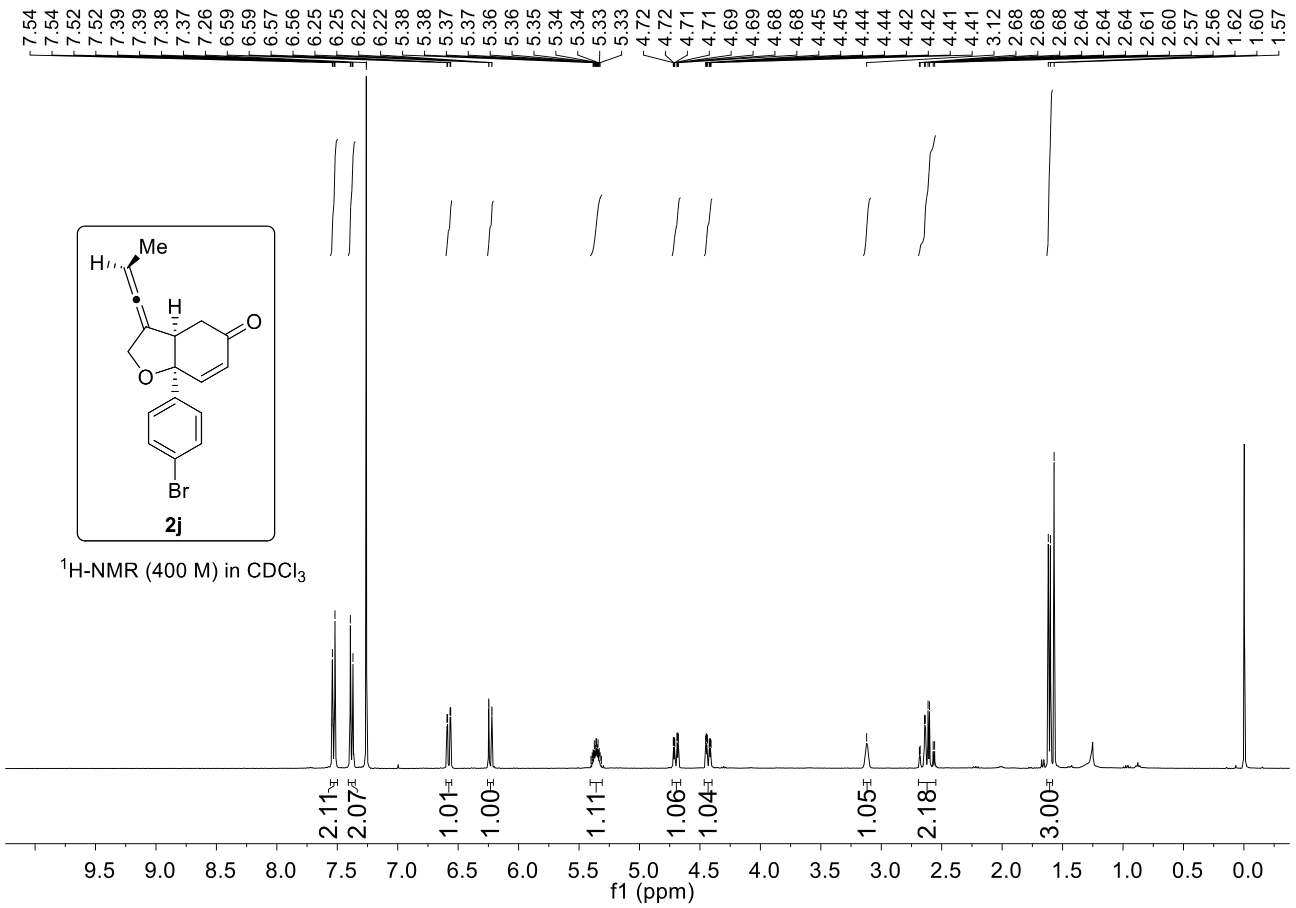

Supplementary Figure 88. ^{1}H NMR spectra for **2j**

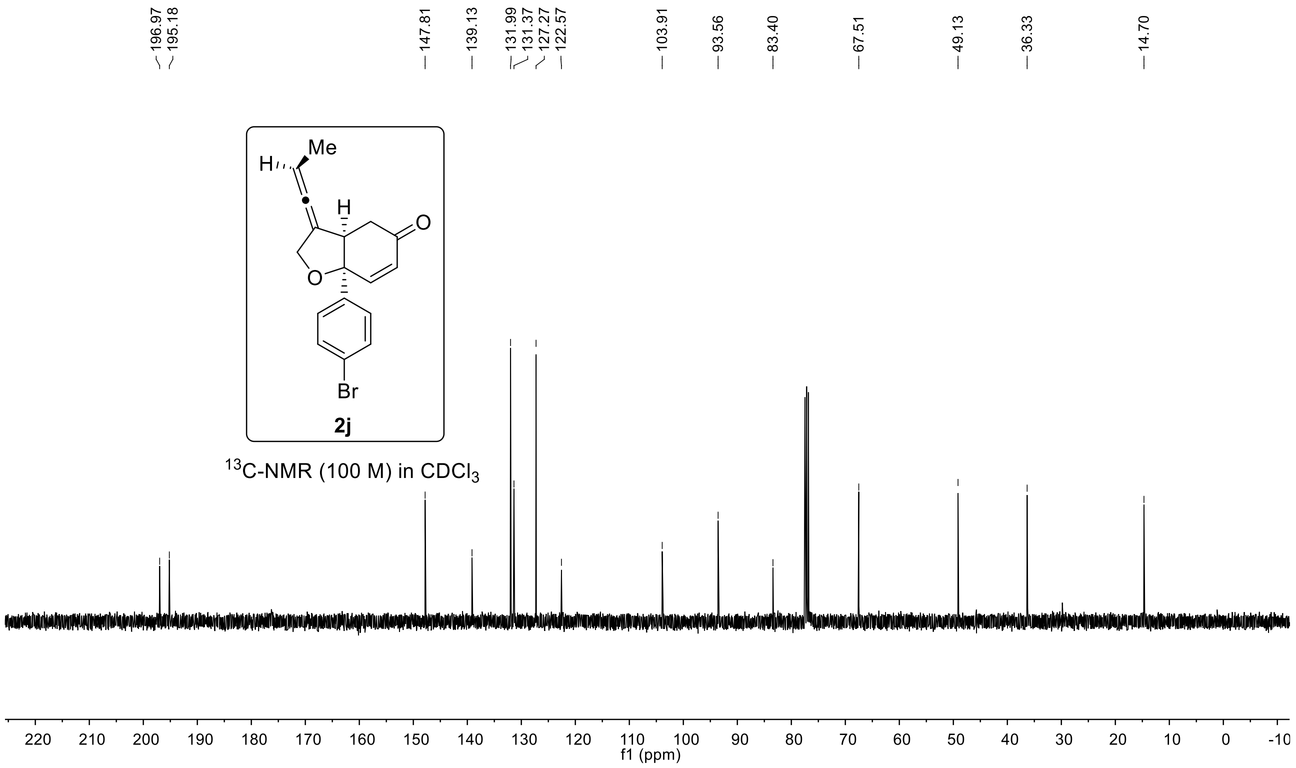

Supplementary Figure 89. ^{13}C NMR spectra for **2j**

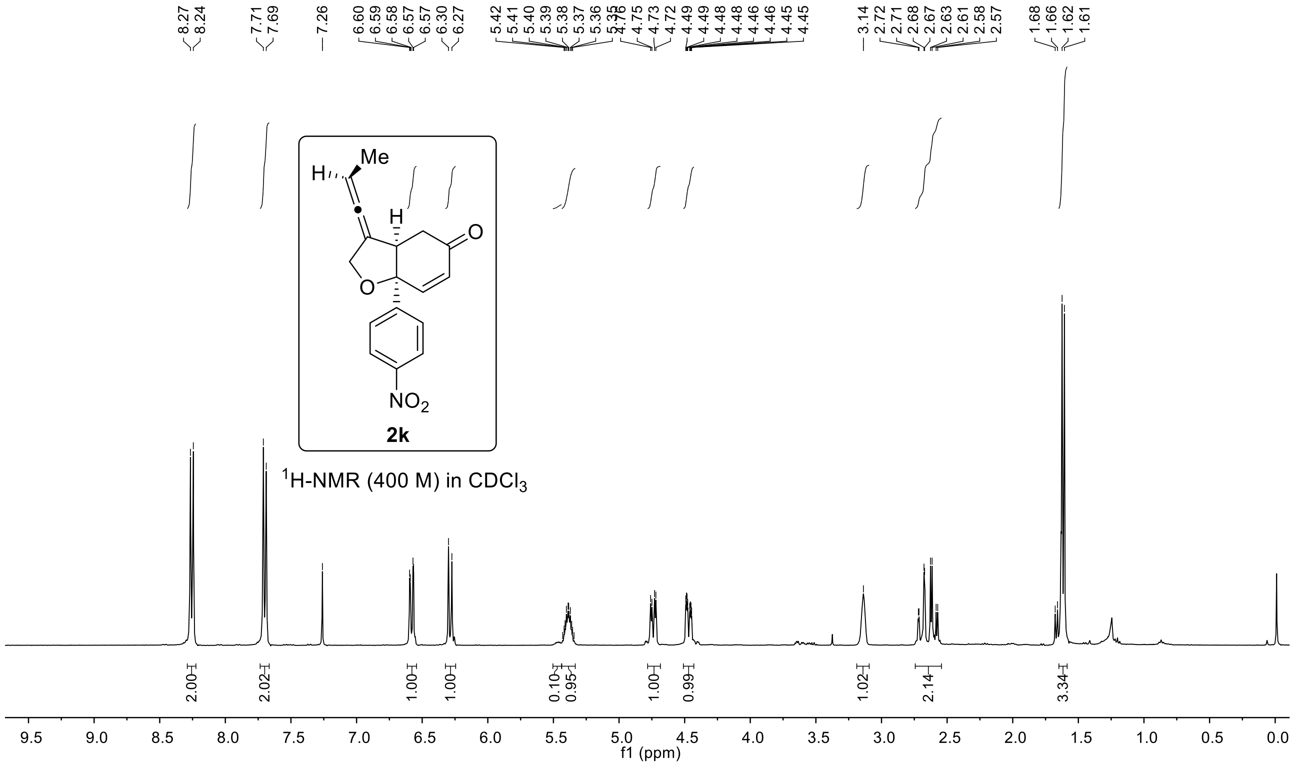

Supplementary Figure 90. ^{1}H NMR spectra for **2k**

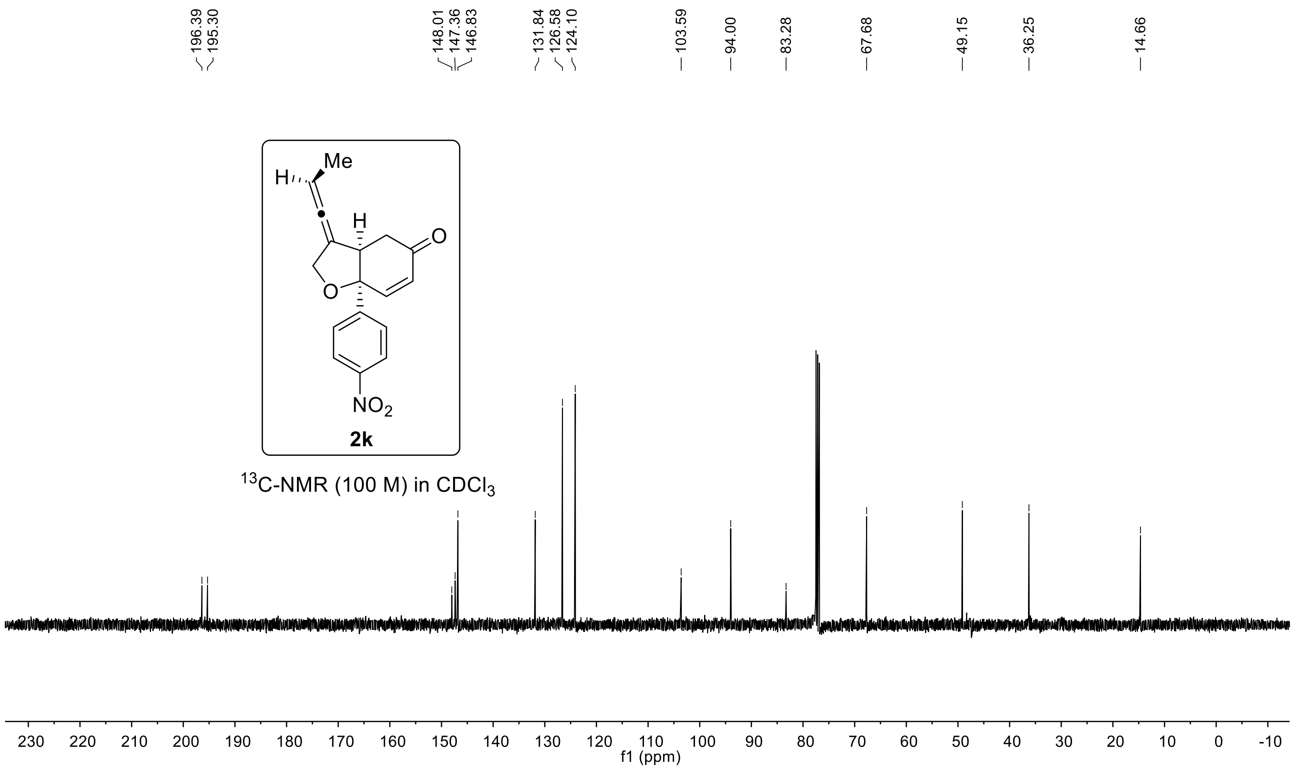

Supplementary Figure 91. ^{13}C NMR spectra for **2k**

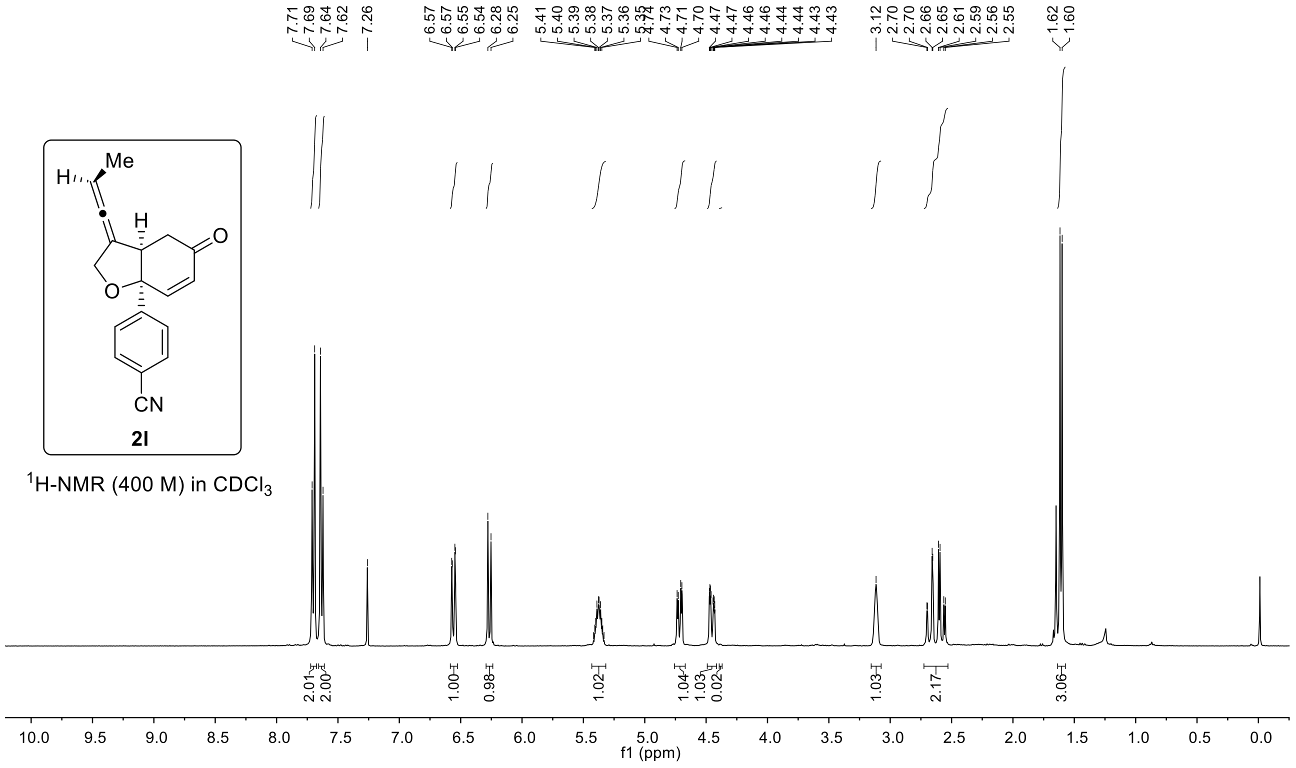

Supplementary Figure 92. ^{1}H NMR spectra for **2l**

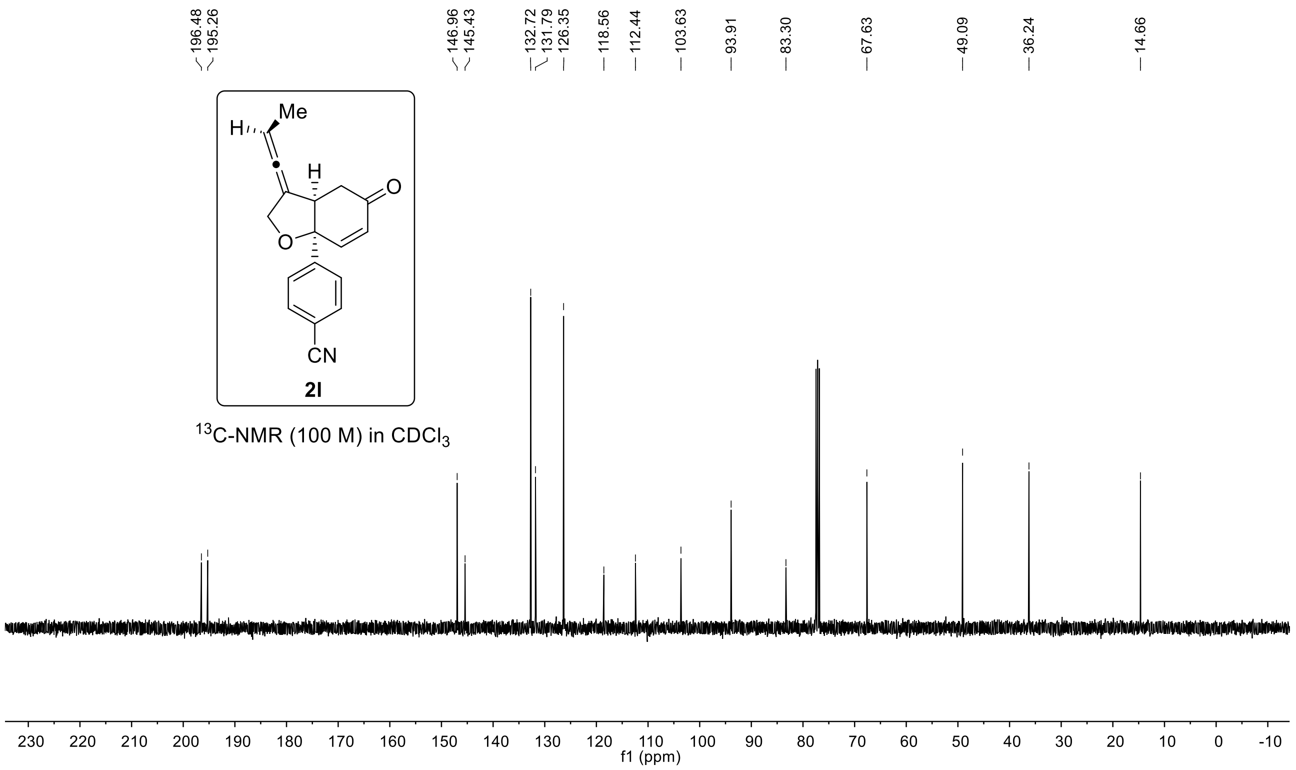

Supplementary Figure 93. ^{13}C NMR spectra for **2l**

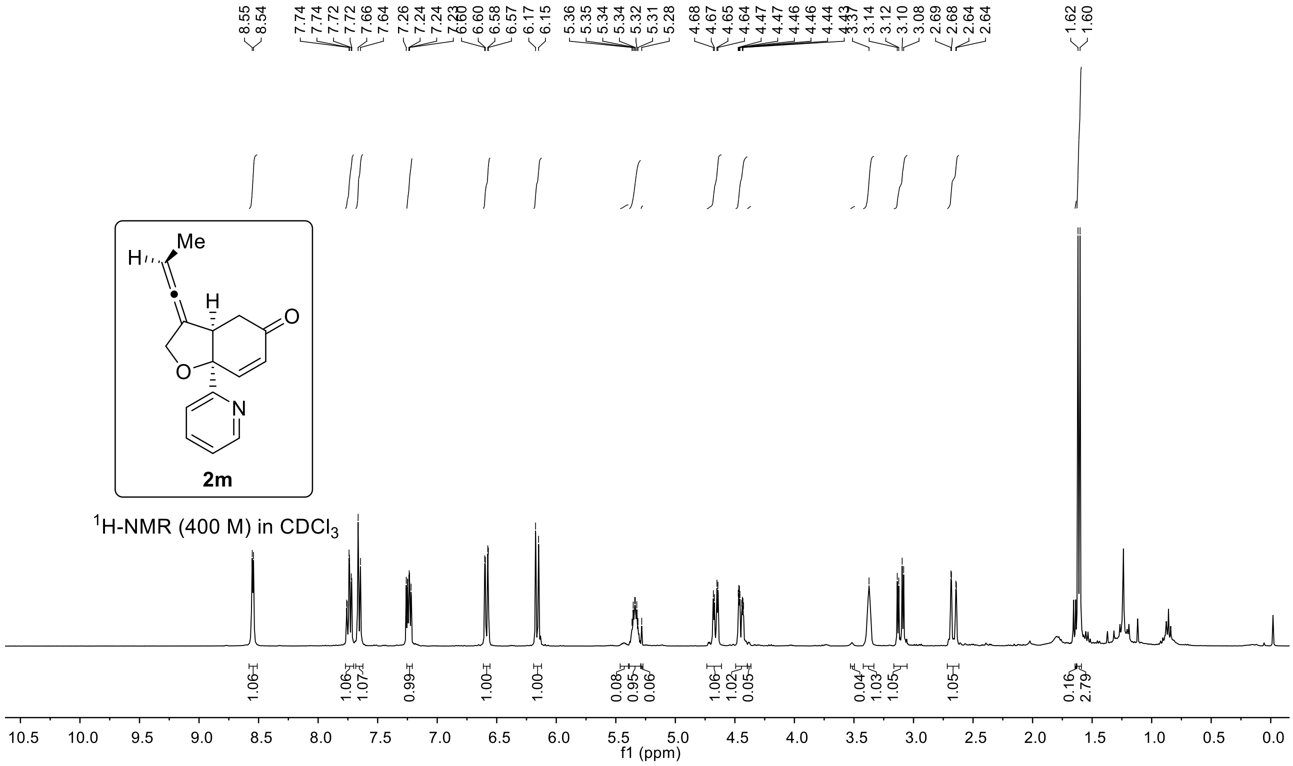

Supplementary Figure 94. ^{1}H NMR spectra for **2m**

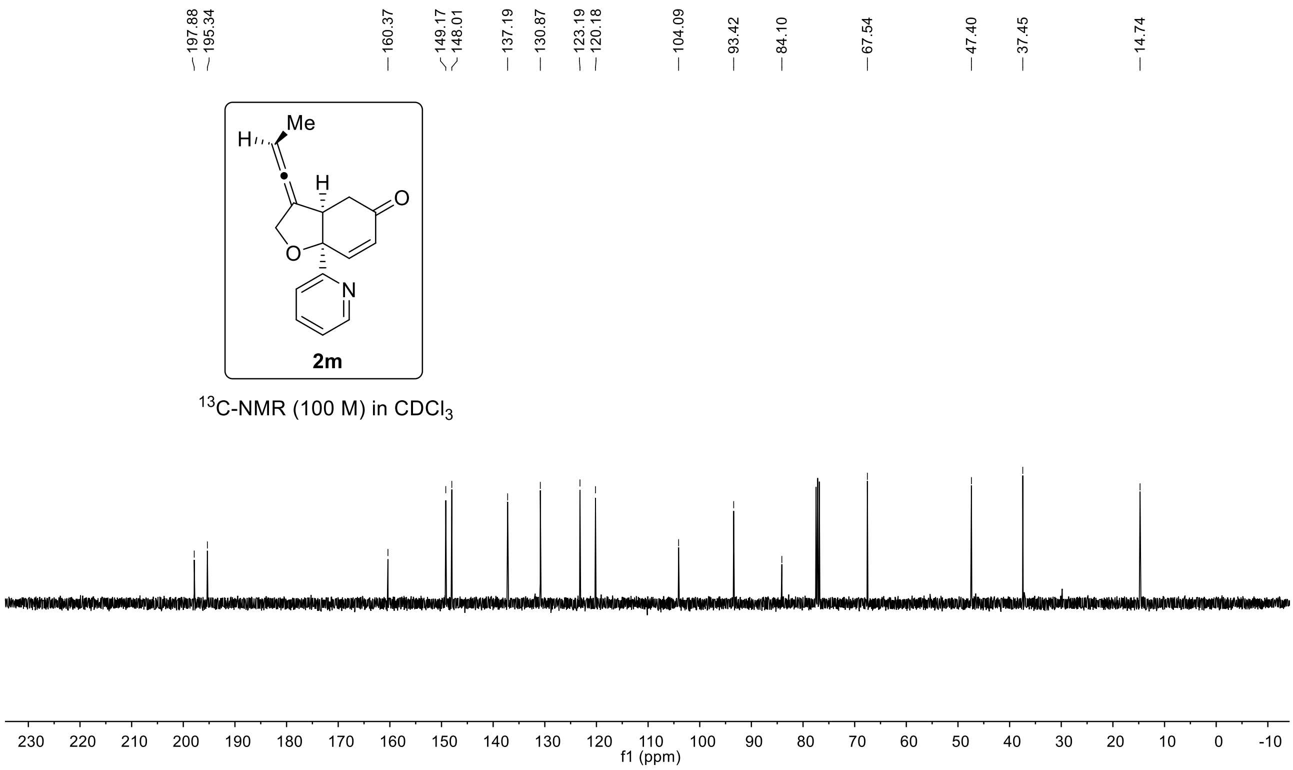

Supplementary Figure 95. ^{13}C NMR spectra for **2m**

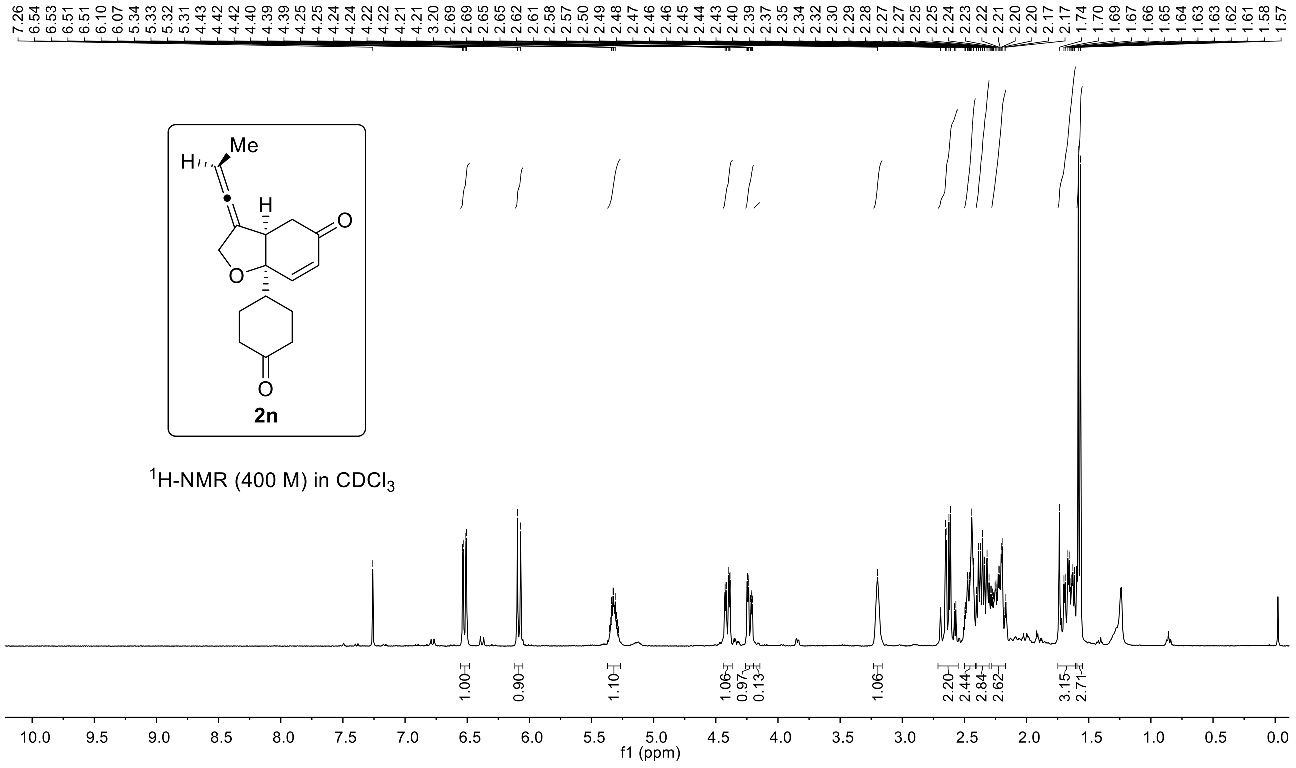

Supplementary Figure 96. ^{1}H NMR spectra for **2n**

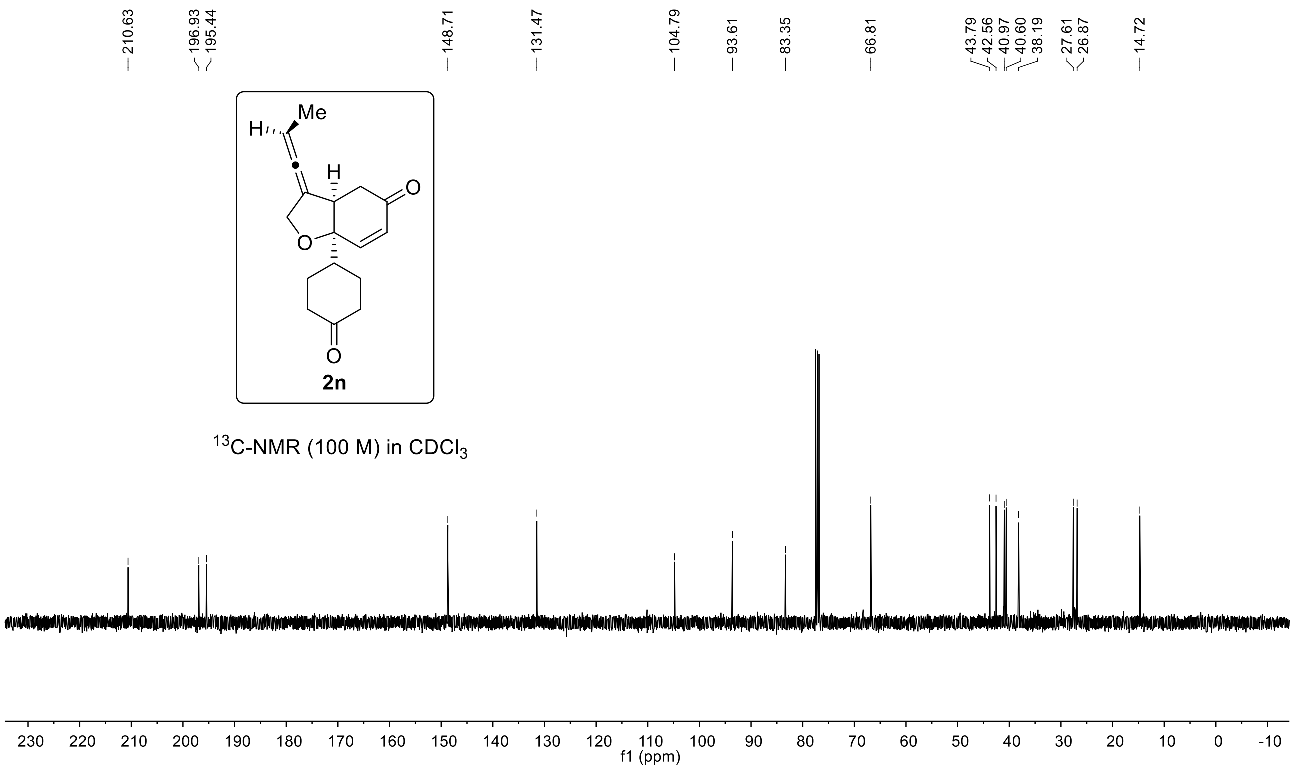

Supplementary Figure 97. ^{13}C NMR spectra for **2n**

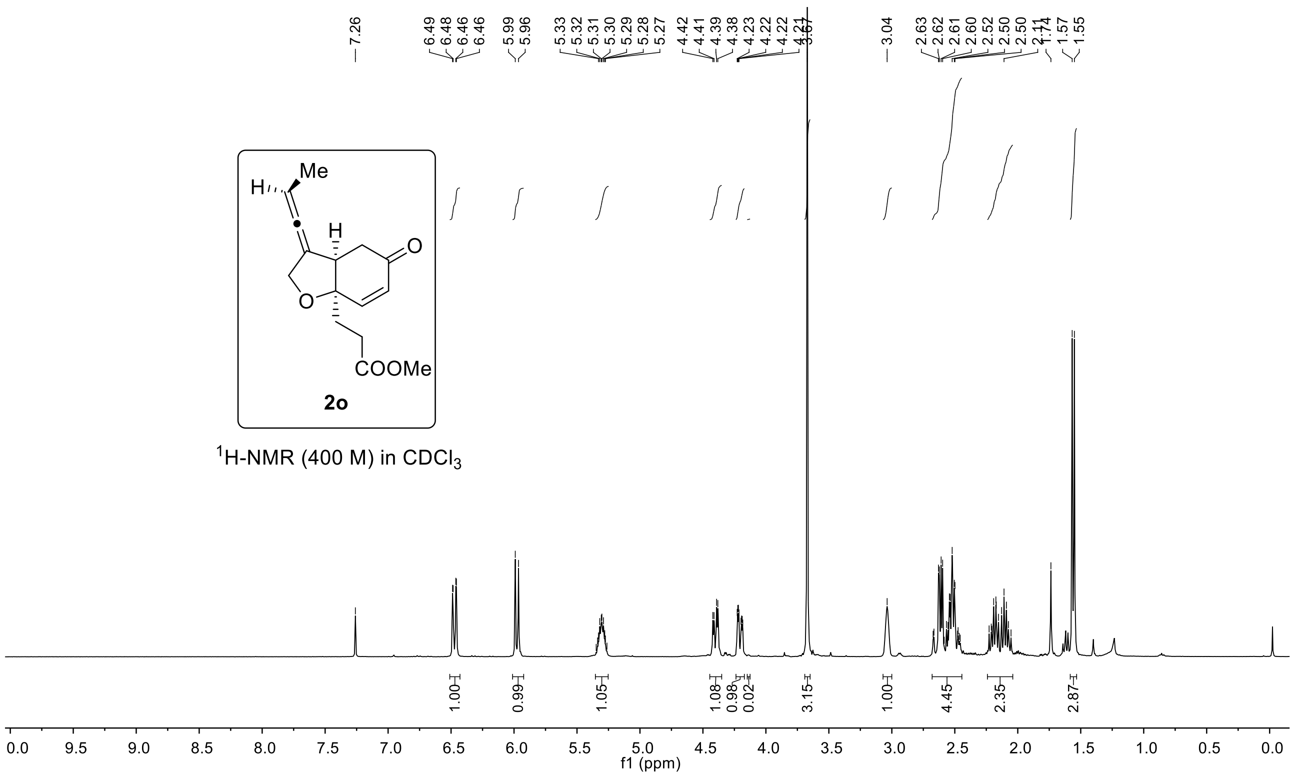

Supplementary Figure 98. ^{1}H NMR spectra for **2o**

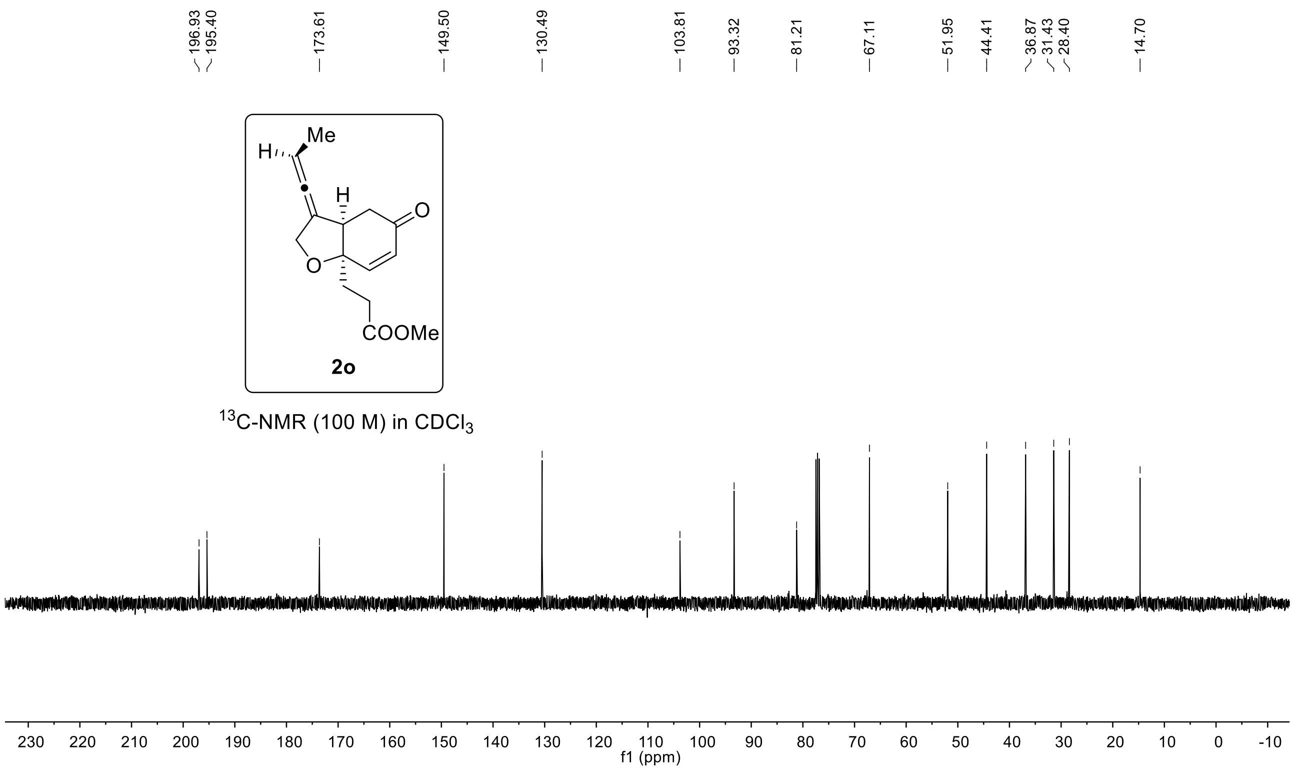

Supplementary Figure 99. ^{13}C NMR spectra for **2o**

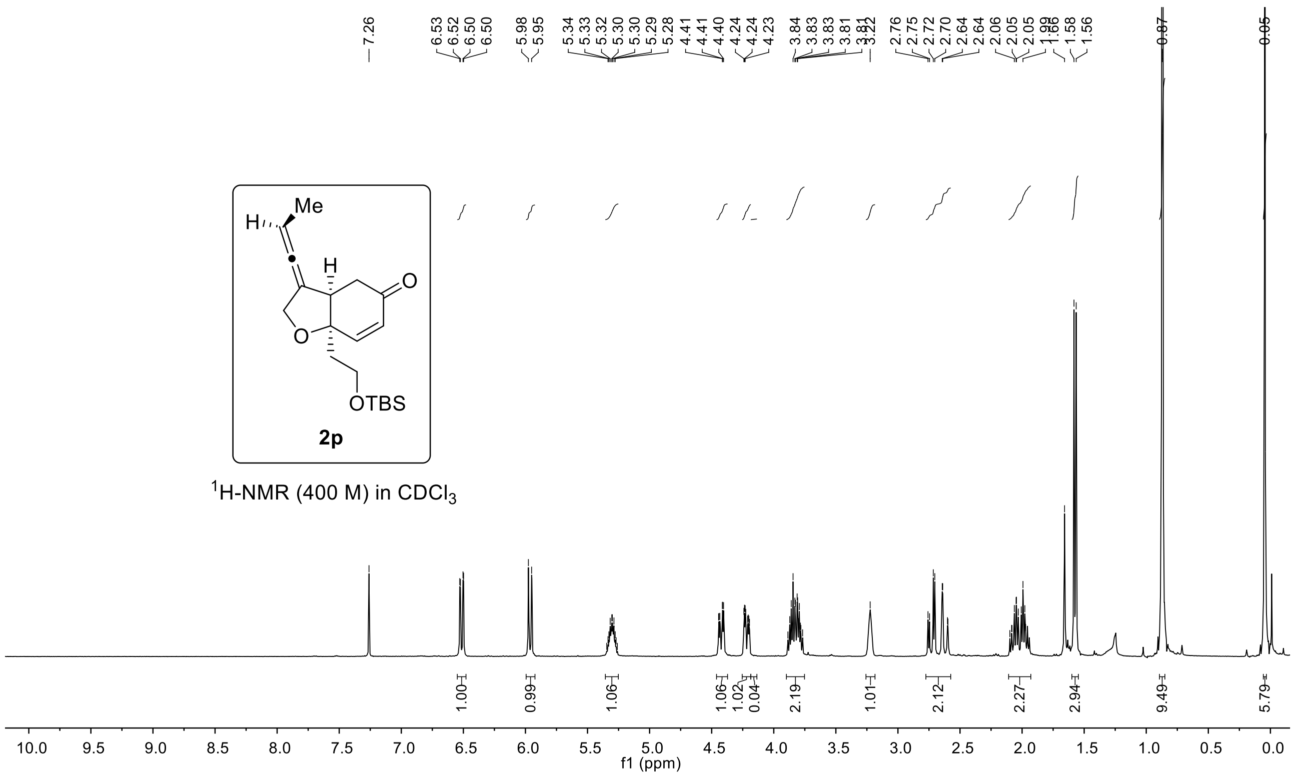

Supplementary Figure 100. ^{1}H NMR spectra for **2p**

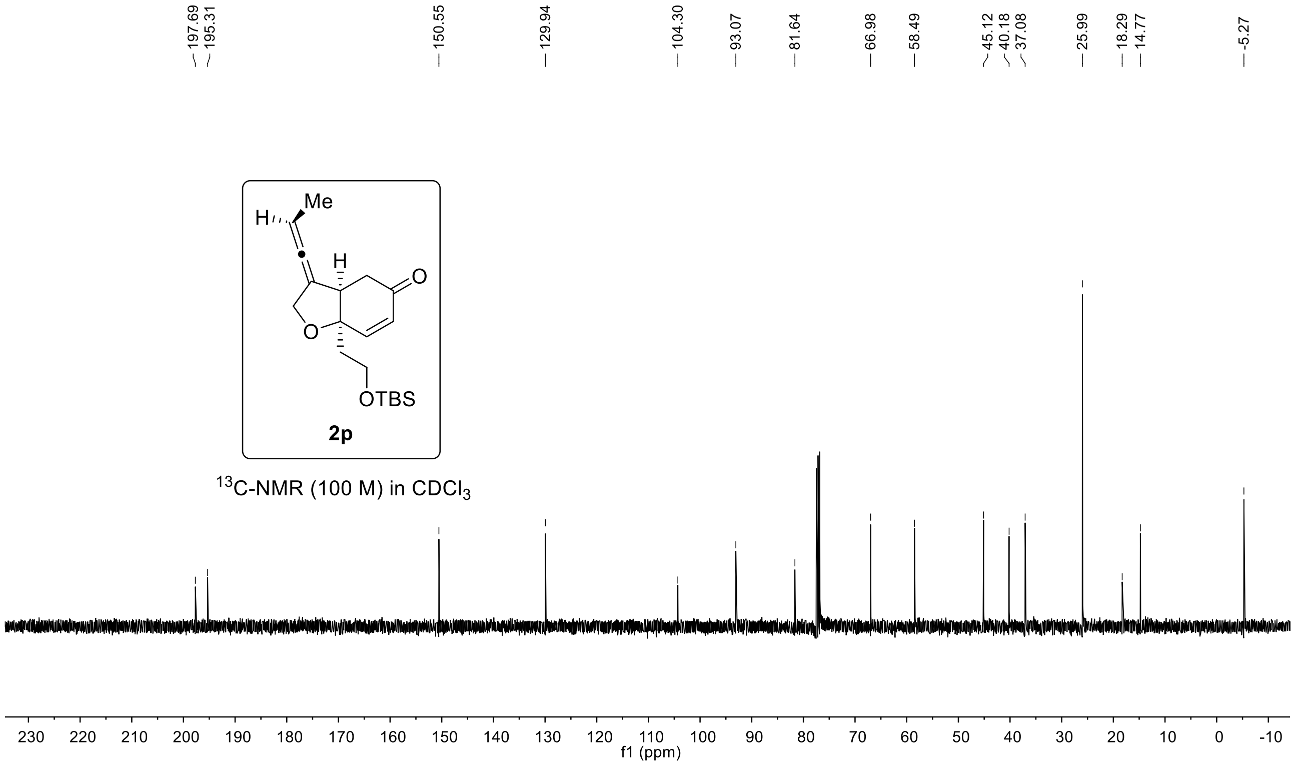

Supplementary Figure 101. ^{13}C NMR spectra for **2p**

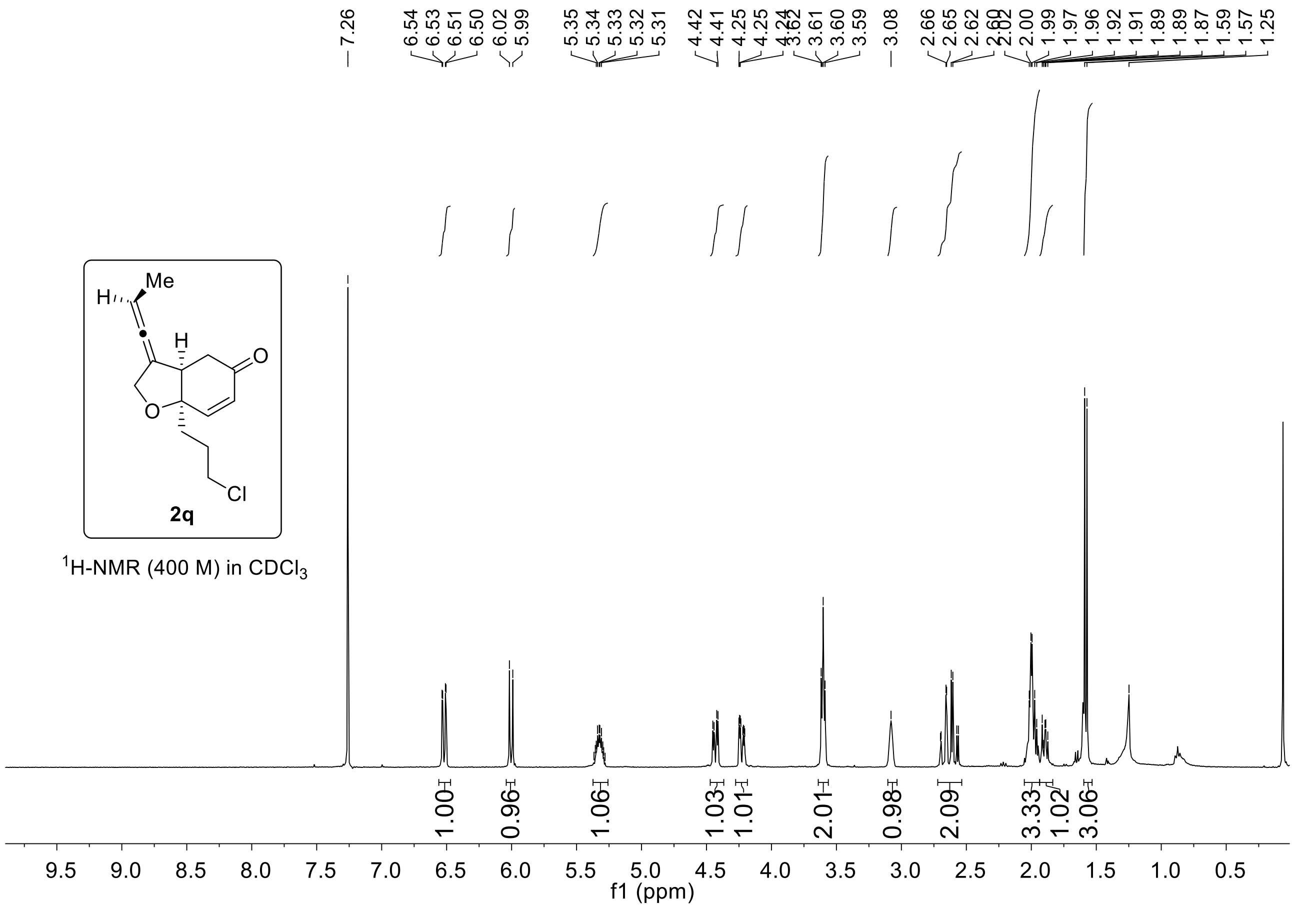

Supplementary Figure 102. ^{1}H NMR spectra for **2q**

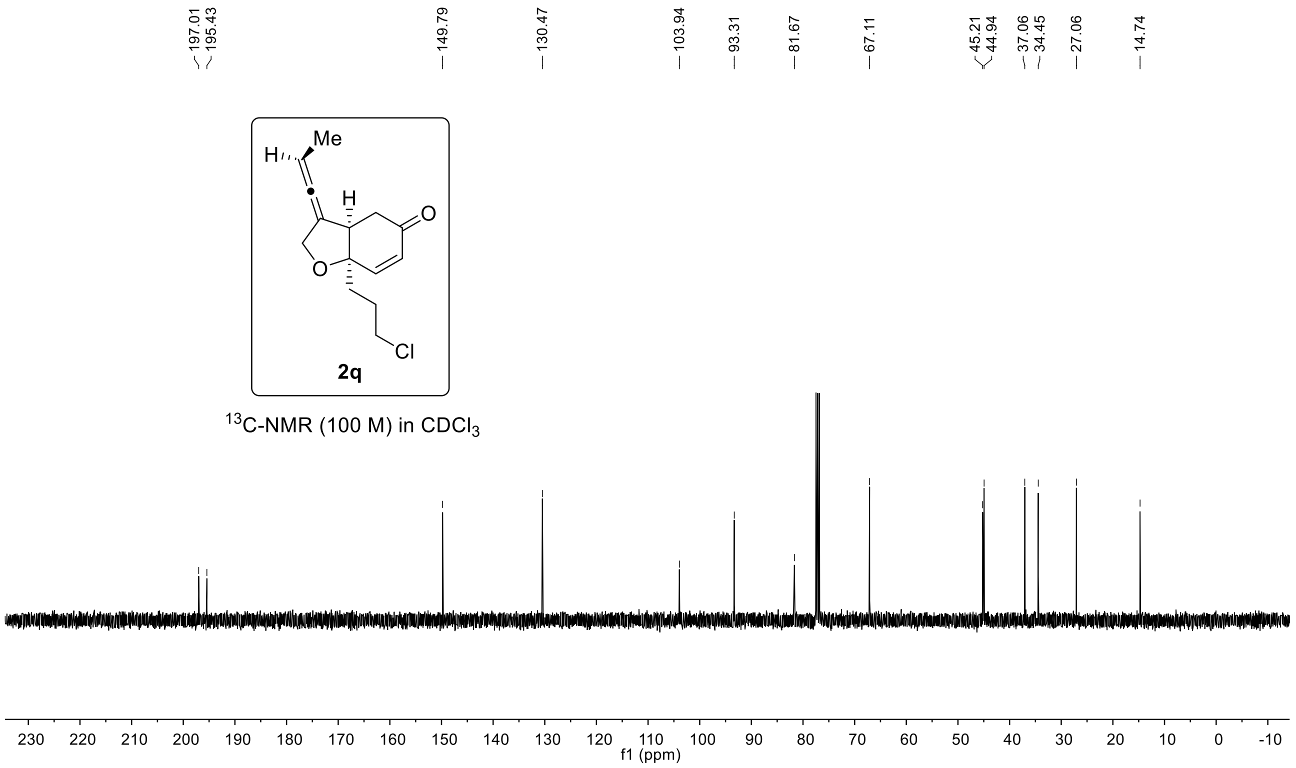

Supplementary Figure 103. ^{13}C NMR spectra for **2q**

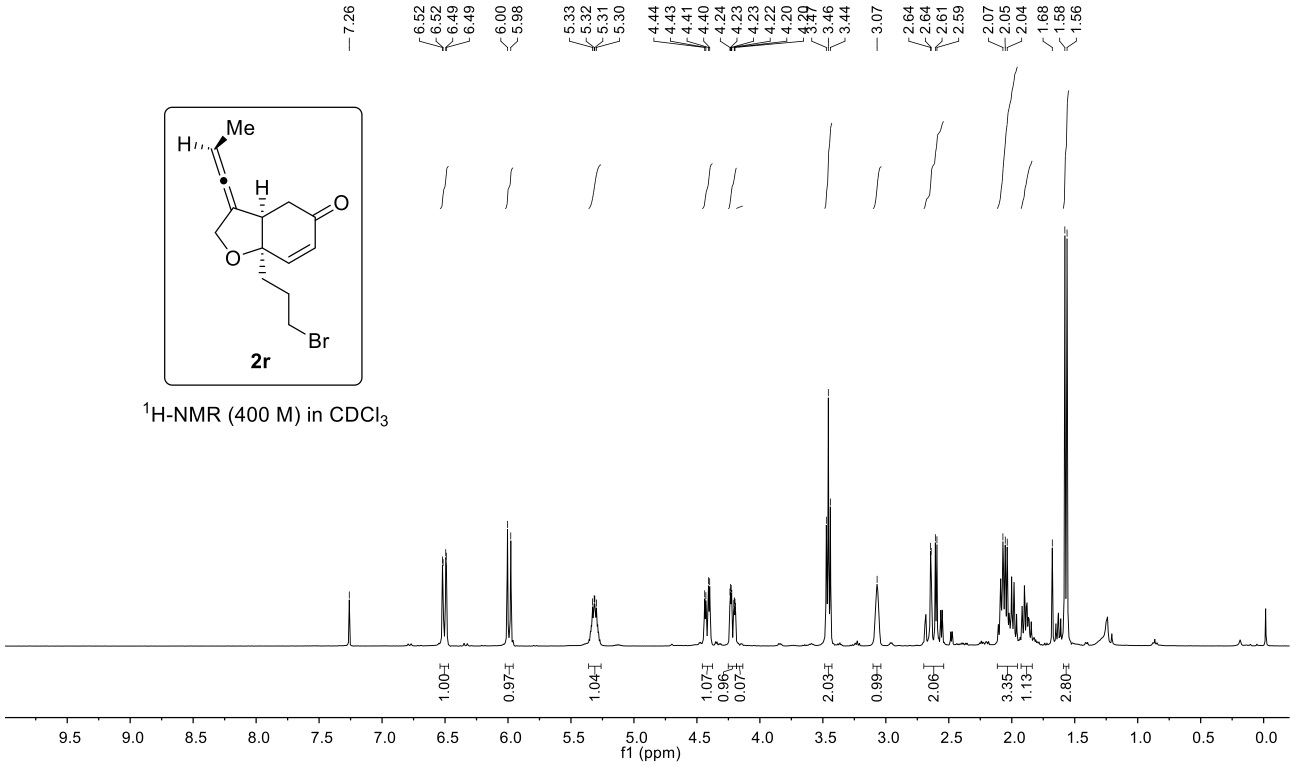

Supplementary Figure 104. ^{1}H NMR spectra for **2r**

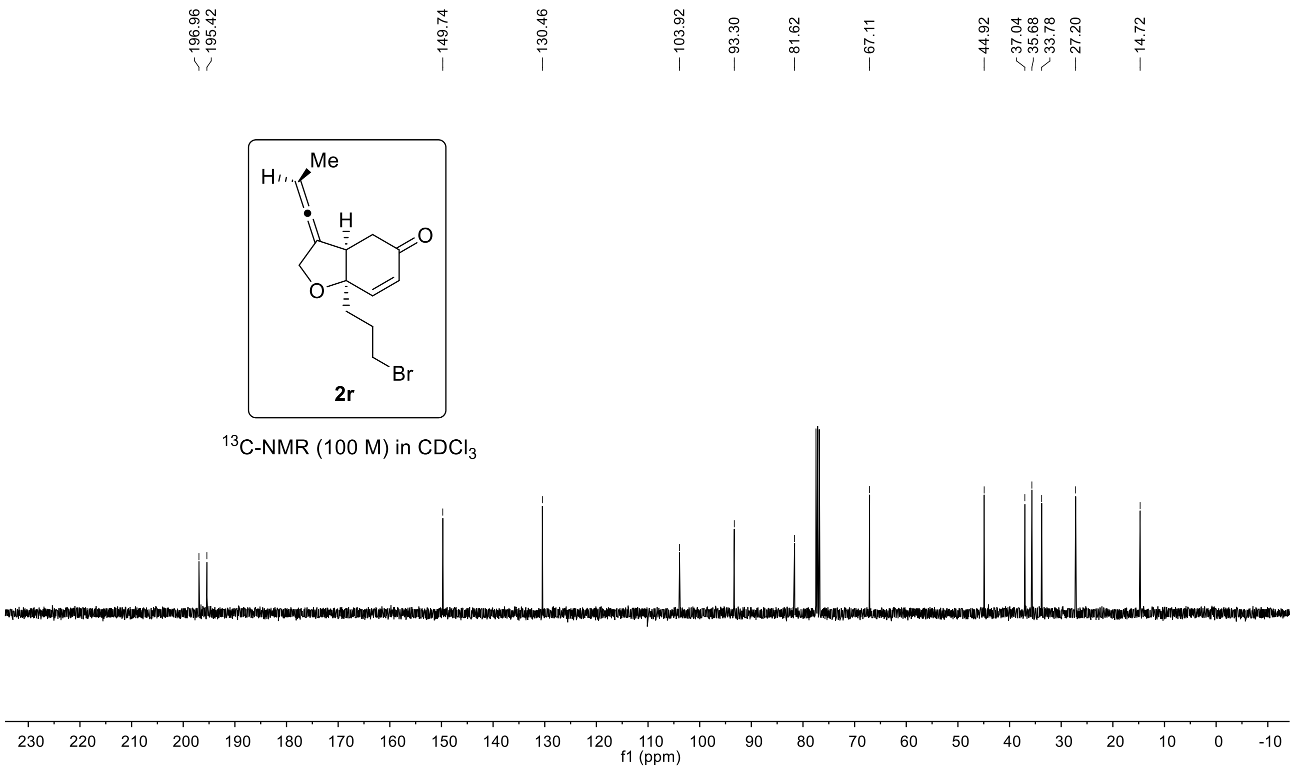

Supplementary Figure 105. ^{13}C NMR spectra for **2r**

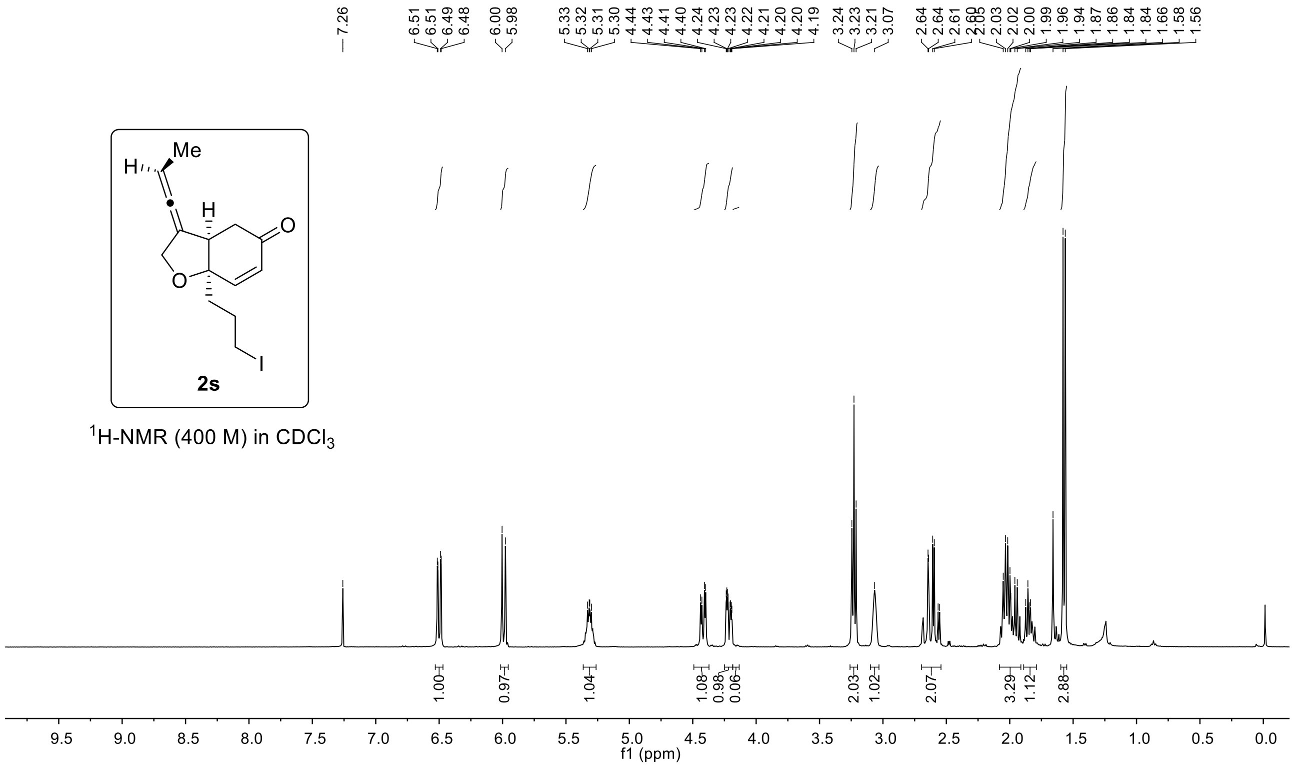

Supplementary Figure 106. ^{1}H NMR spectra for **2s**

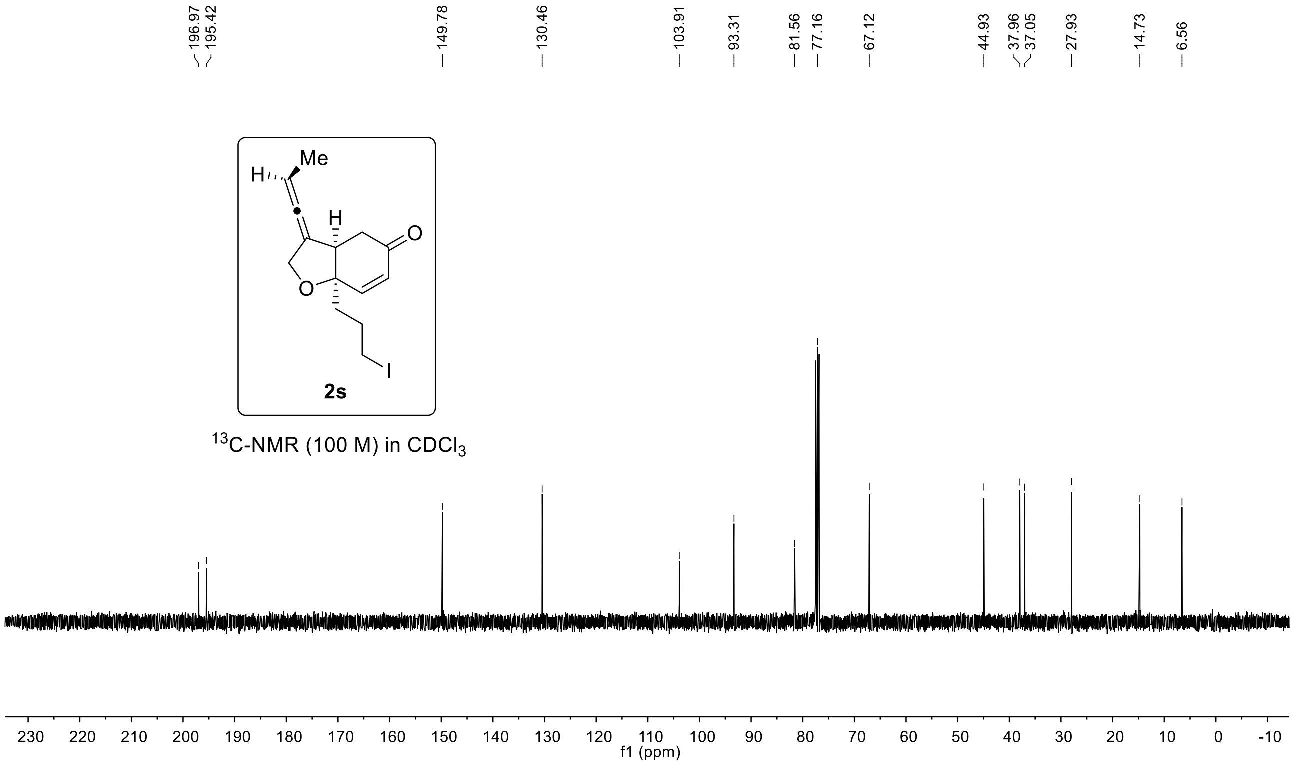

Supplementary Figure 107. ^{13}C NMR spectra for **2s**

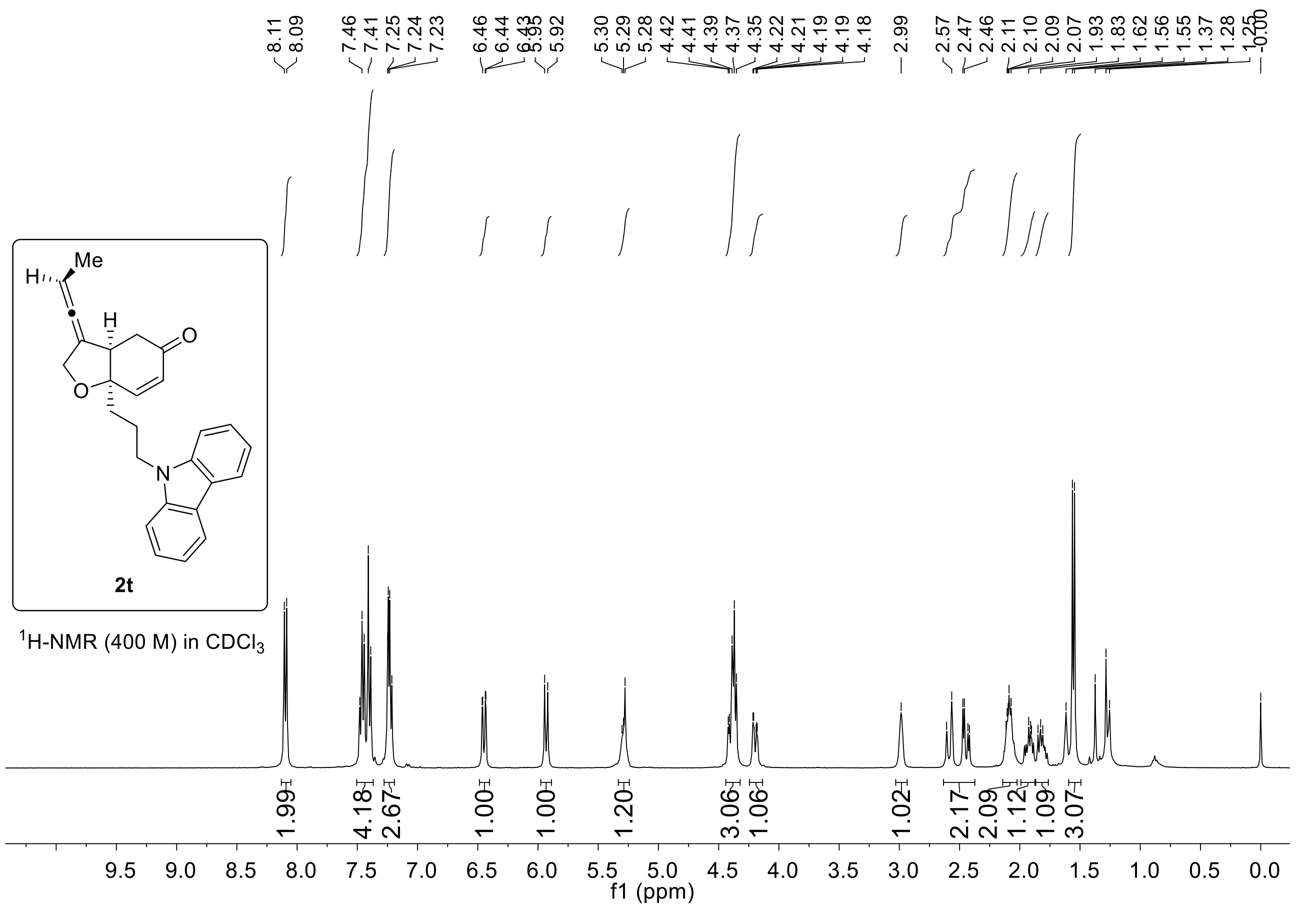

Supplementary Figure 108. ^{1}H NMR spectra for **2t**

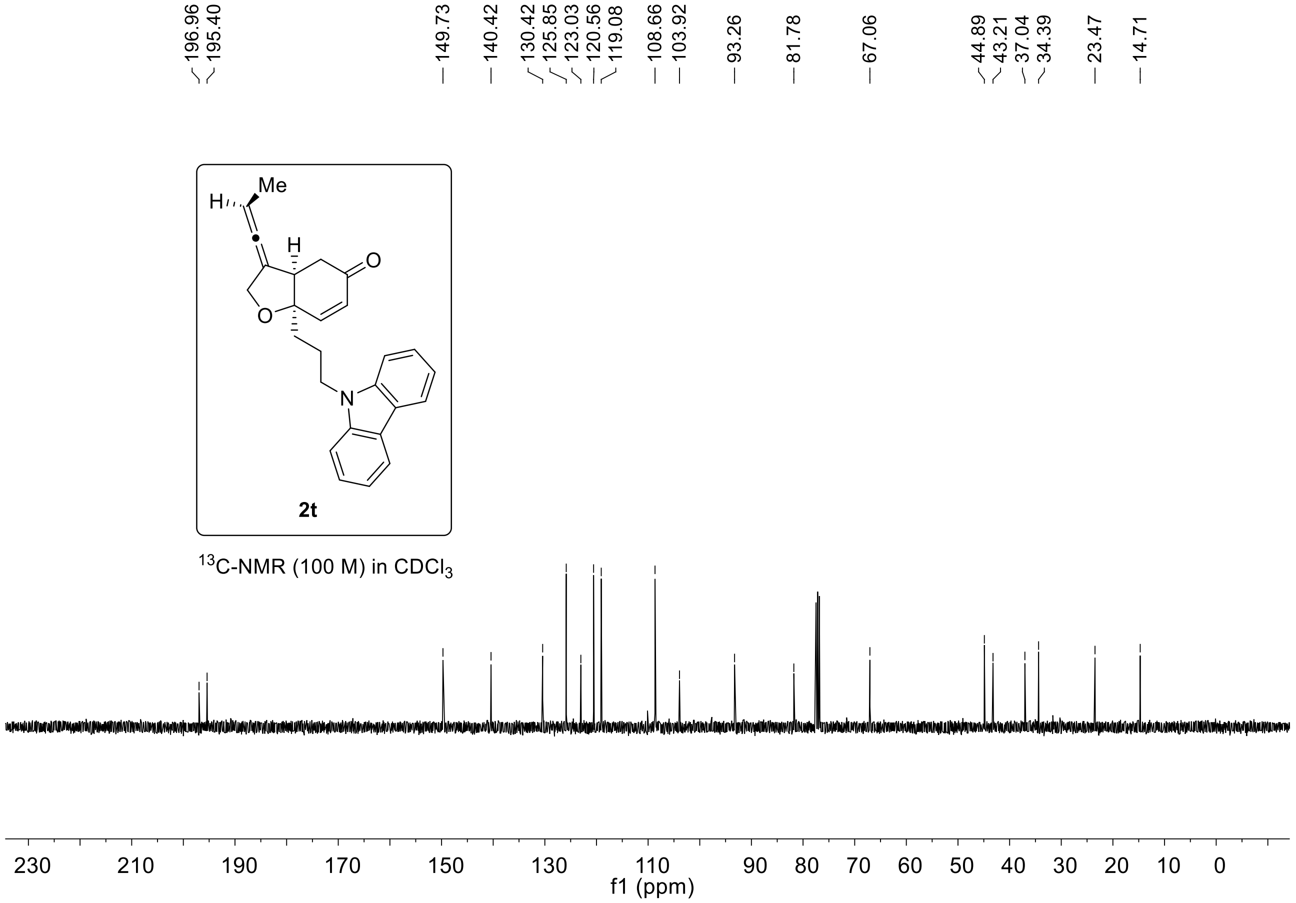

Supplementary Figure 109. ^{13}C NMR spectra for **2t**

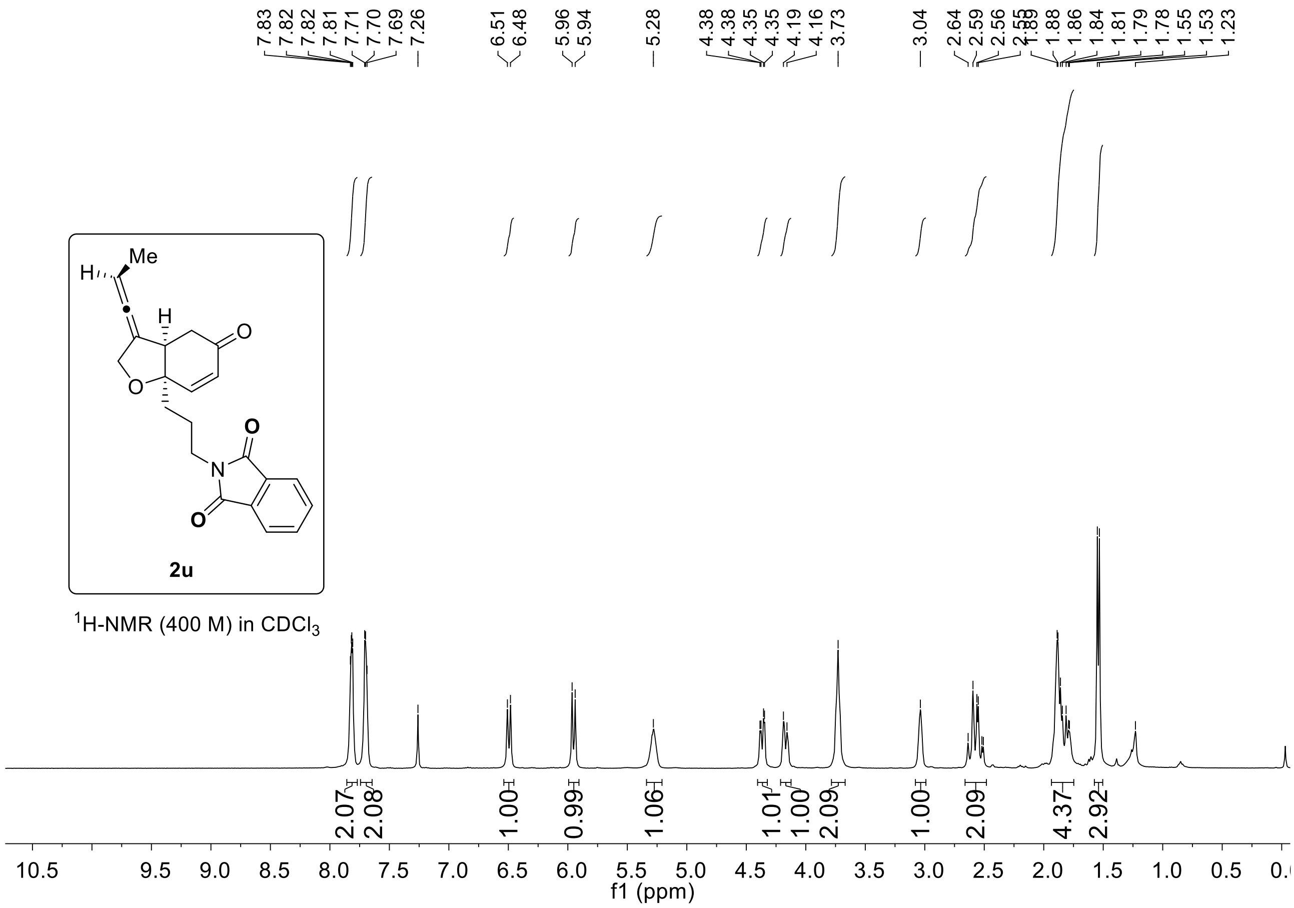

Supplementary Figure 110. ^{1}H NMR spectra for **2u**

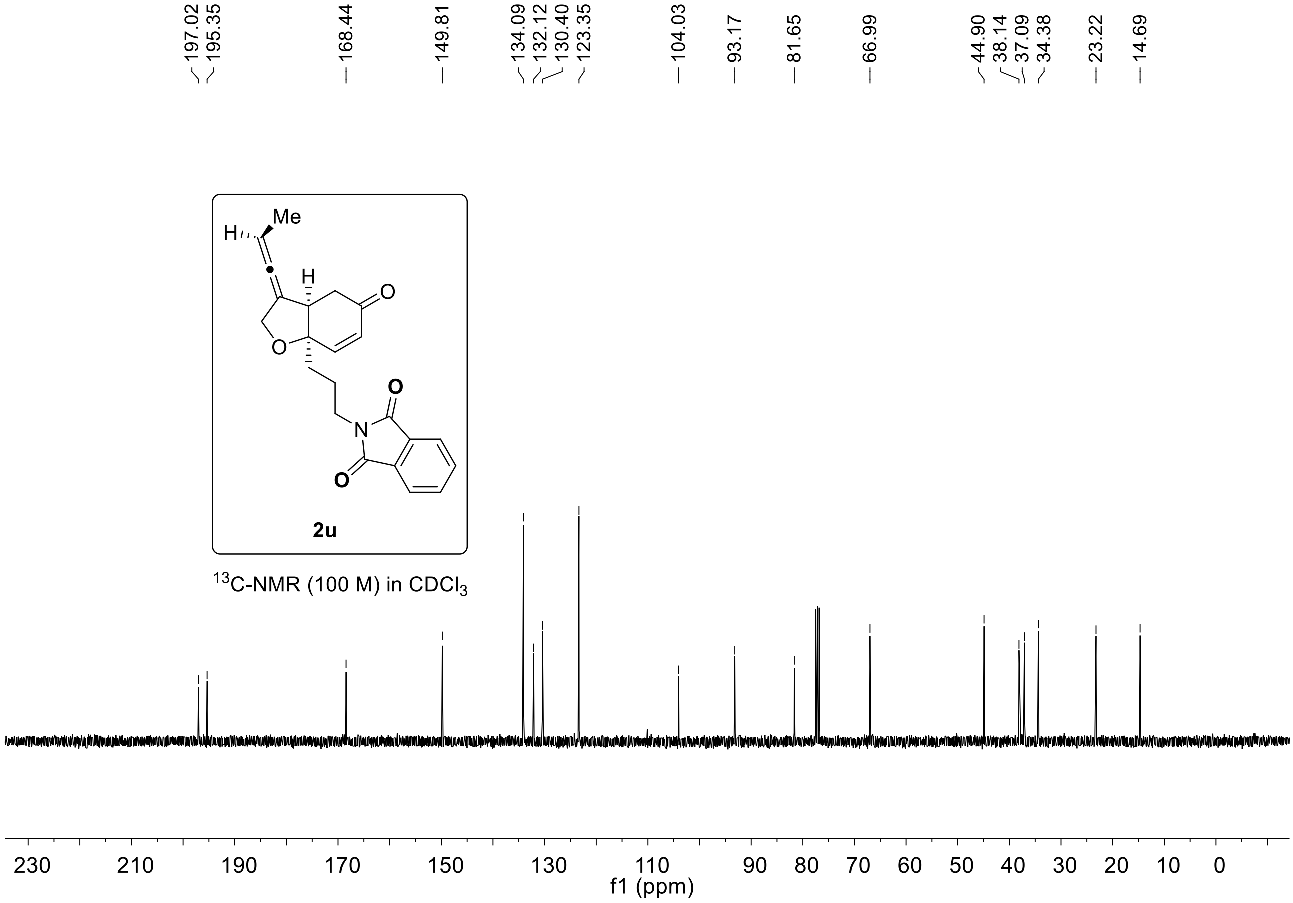

Supplementary Figure 111. ^{13}C NMR spectra for **2u**

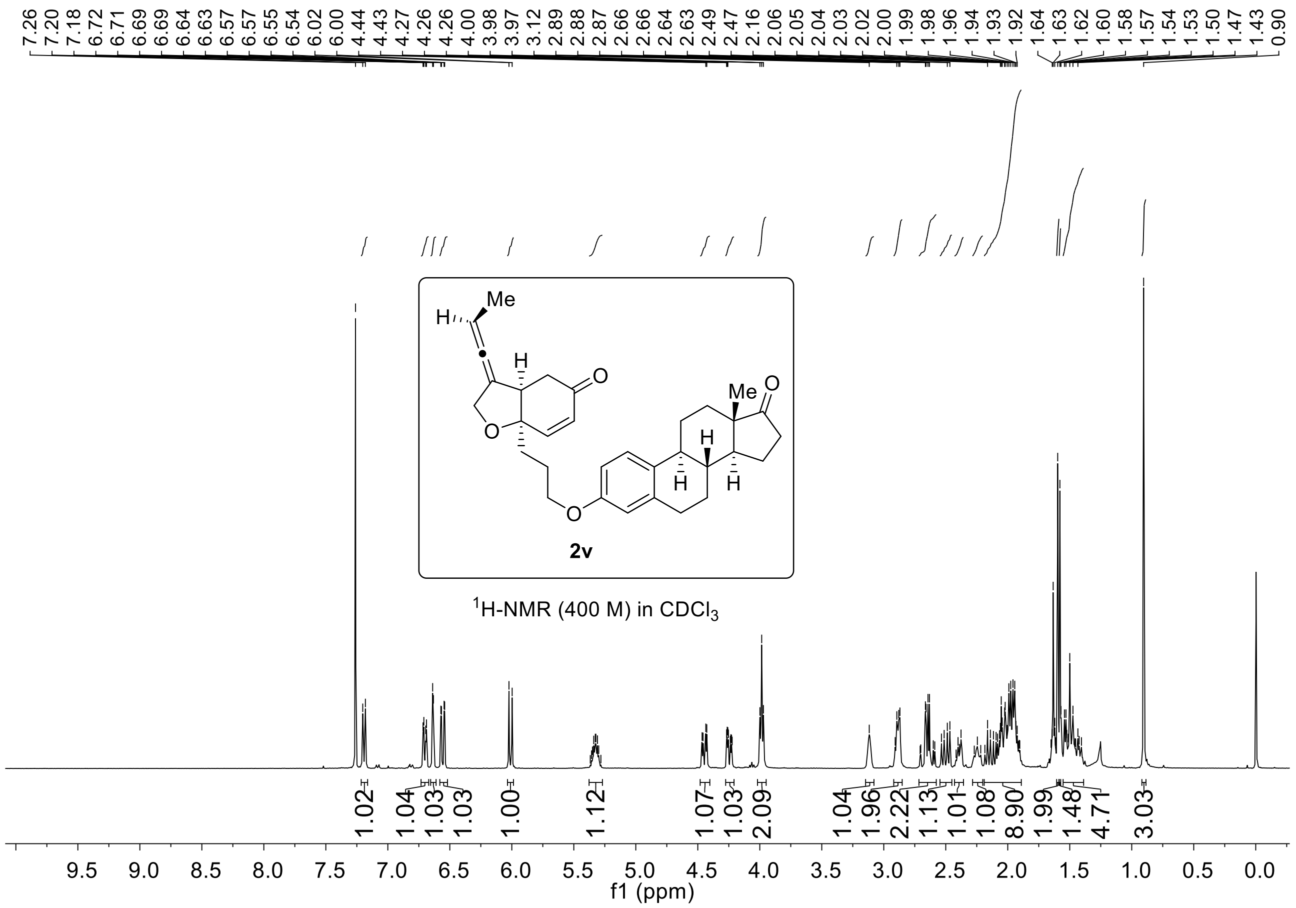

Supplementary Figure 112. ^{1}H NMR spectra for **2v**

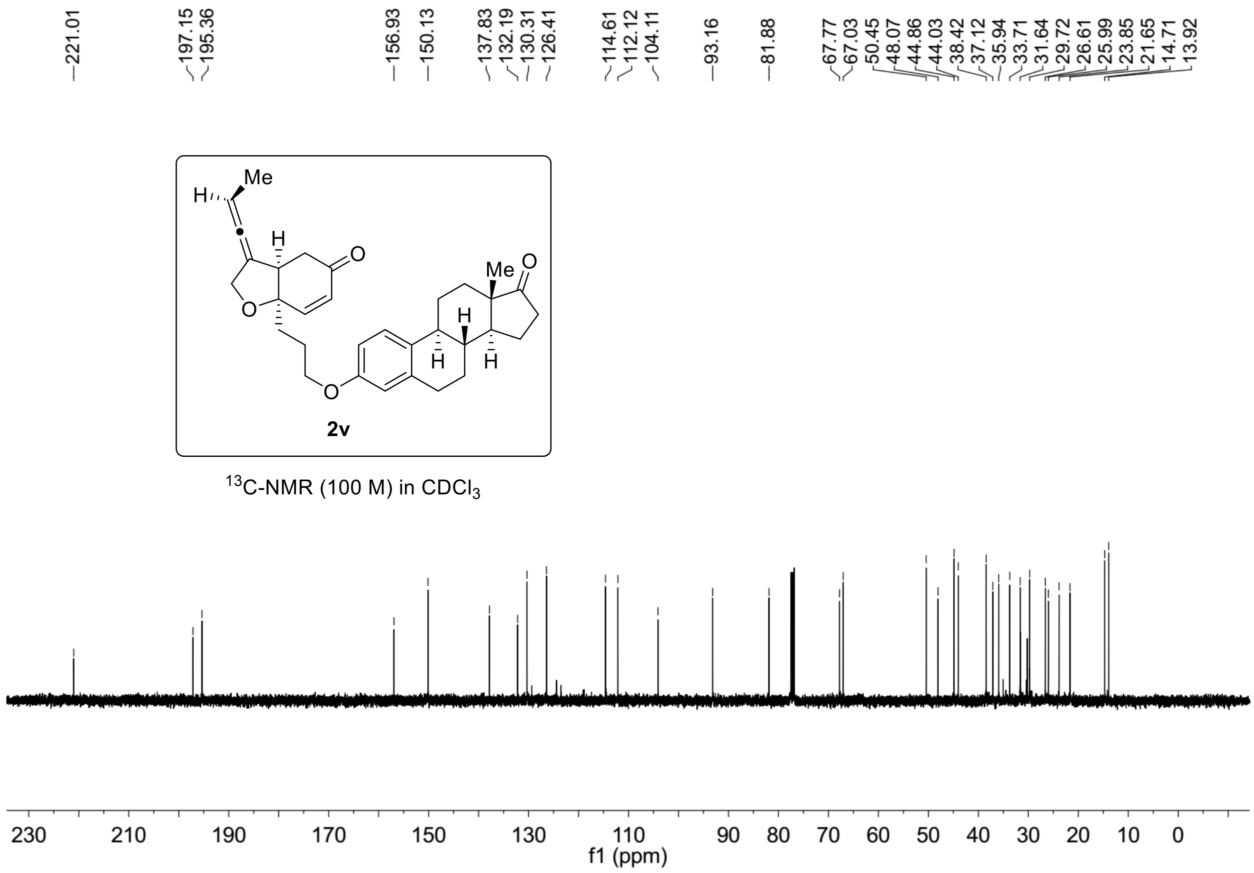

Supplementary Figure 113. ^{13}C NMR spectra for **2v**

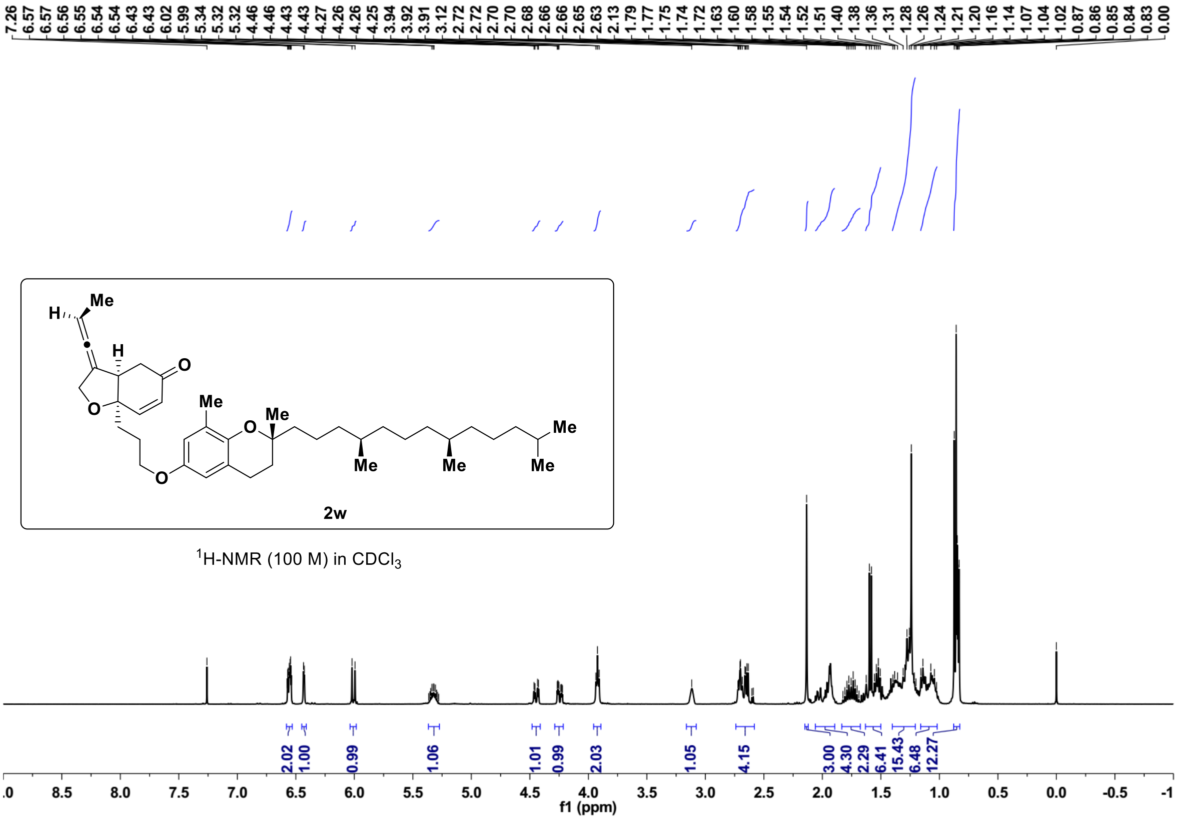

Supplementary Figure 114. ^{1}H NMR spectra for **2w**

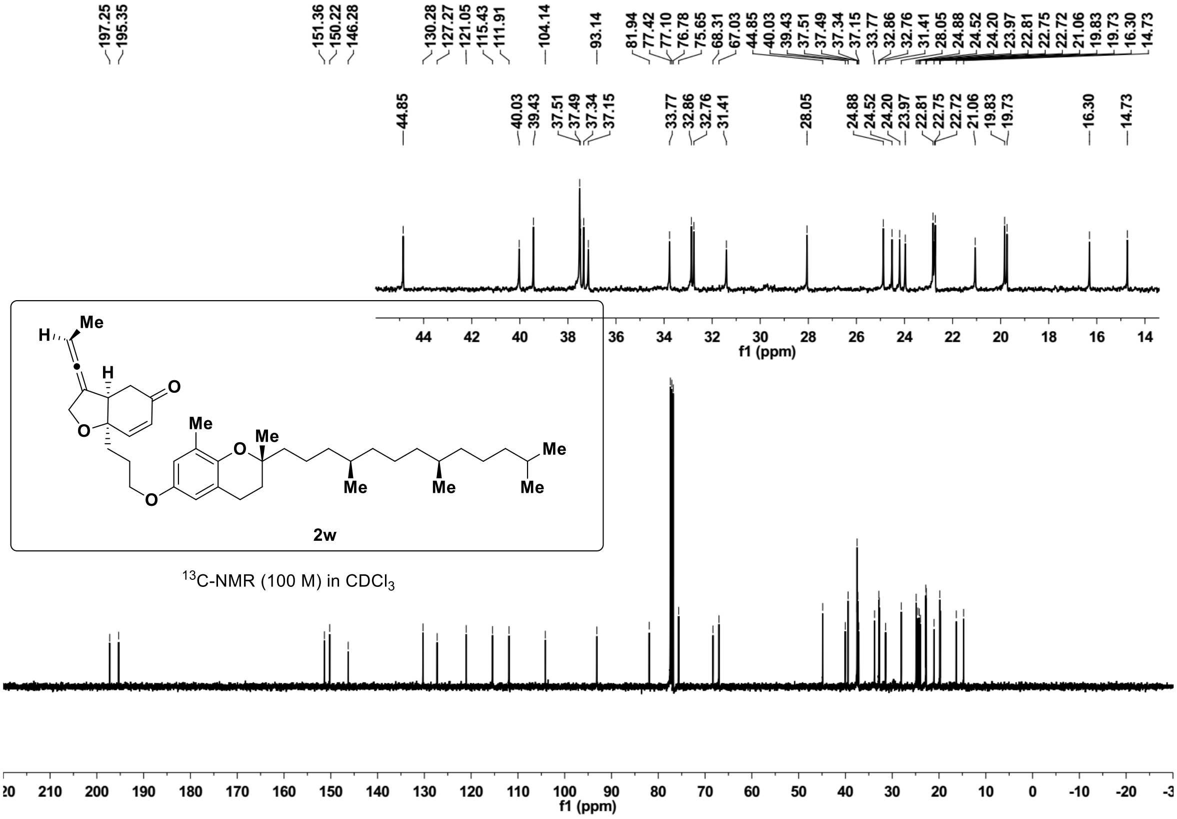

Supplementary Figure 115. ^{13}C NMR spectra for **2w**

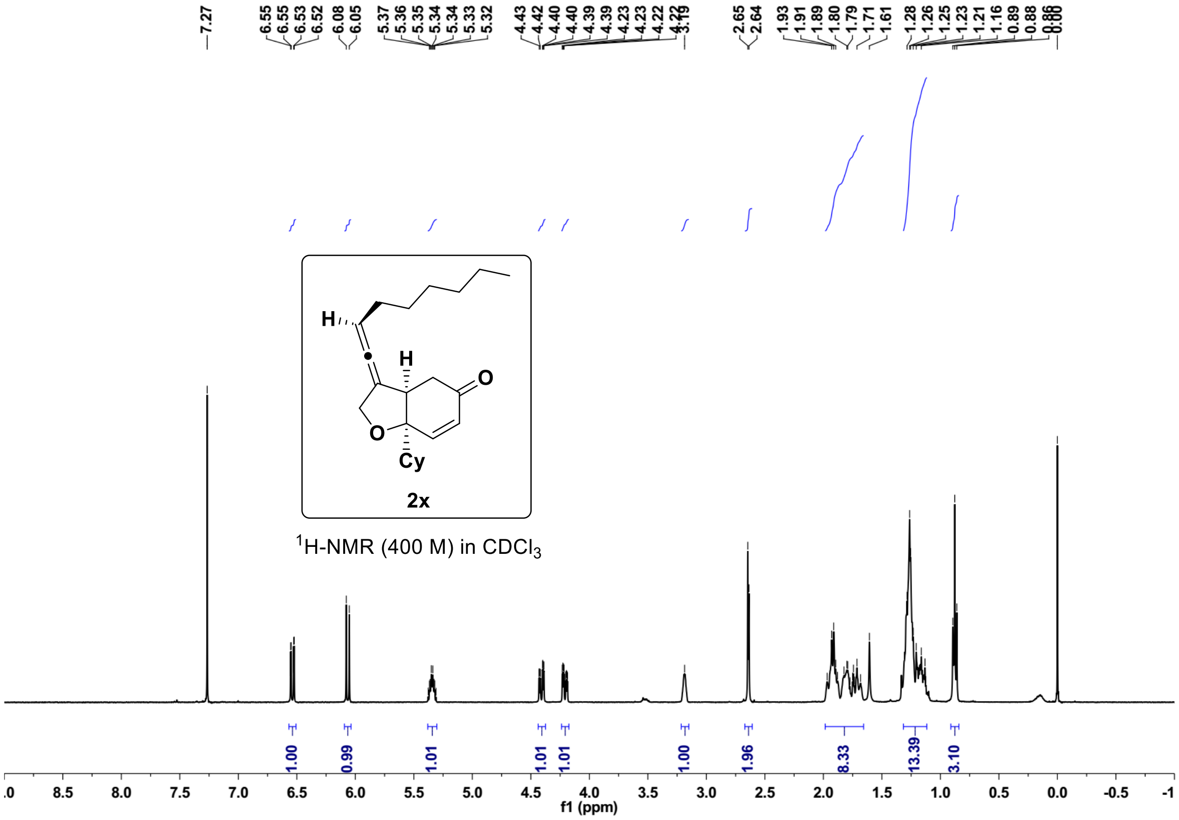

Supplementary Figure 116. ^{1}H NMR spectra for **2x**

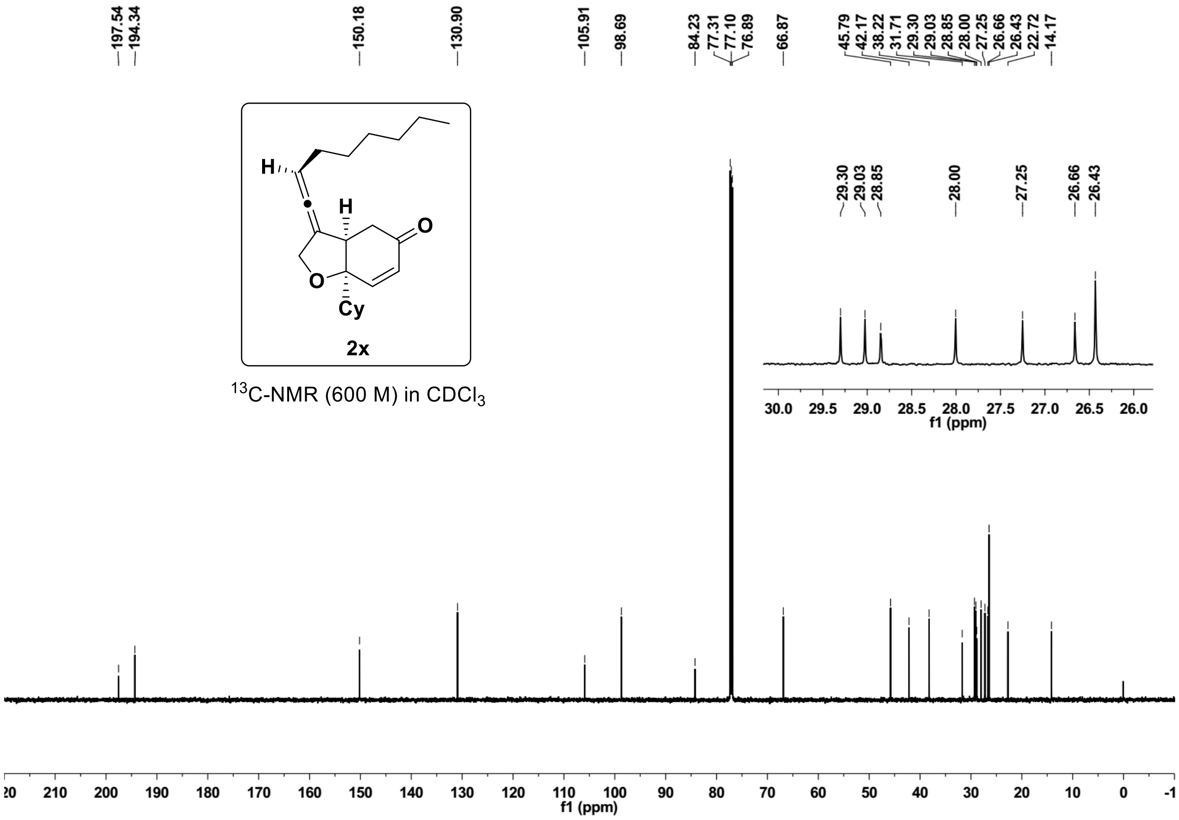

Supplementary Figure 117. ^{13}C NMR spectra for **2x**

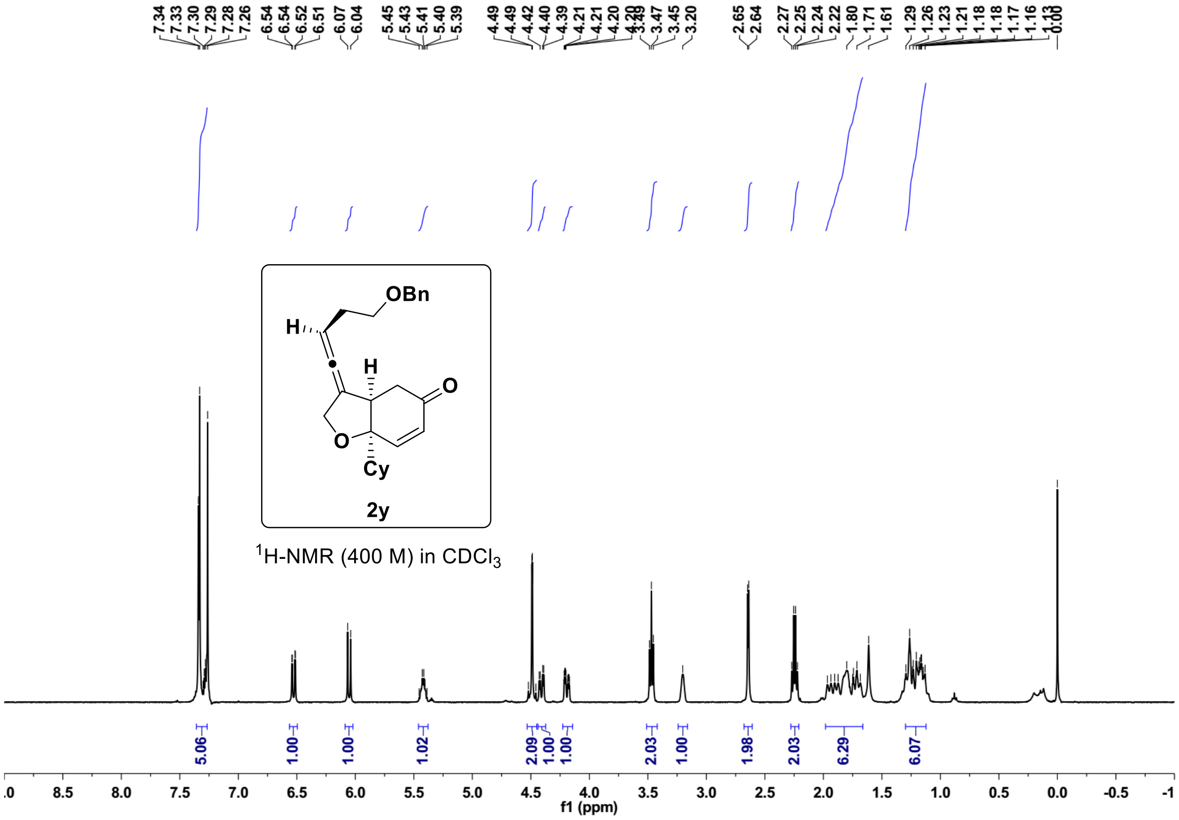

Supplementary Figure 118. ^{1}H NMR spectra for **2y**

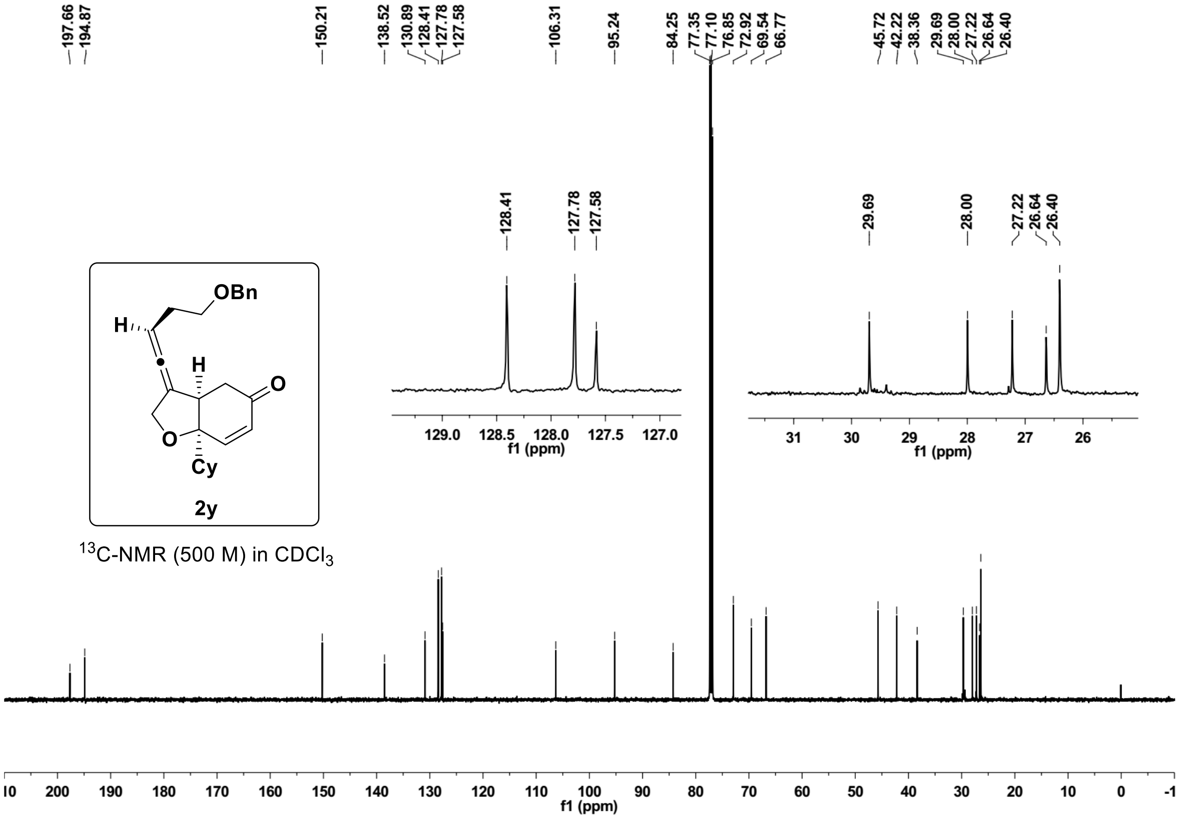

Supplementary Figure 119. ^{13}C NMR spectra for **2y**

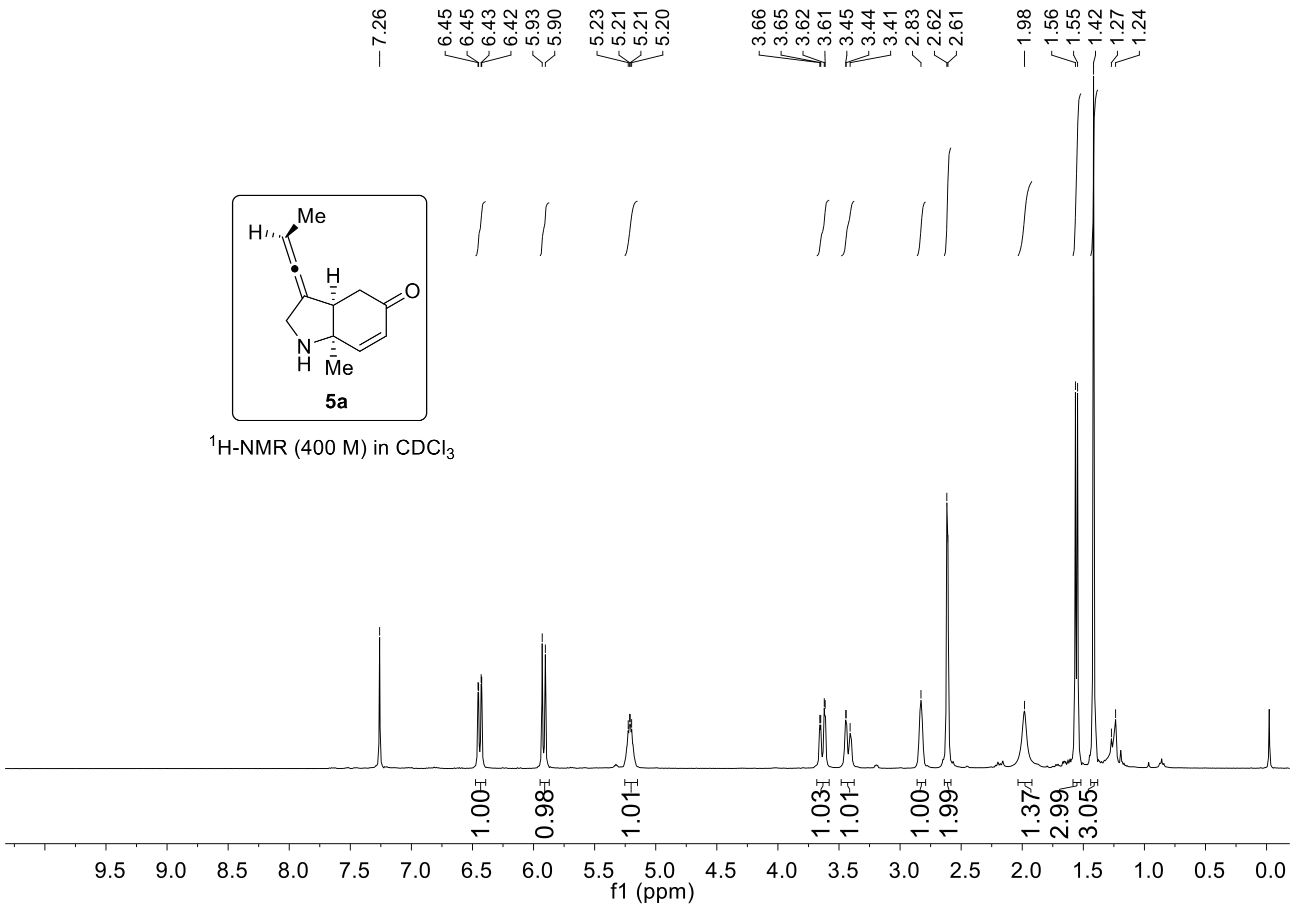

Supplementary Figure 120. ^{1}H NMR spectra for **5a**

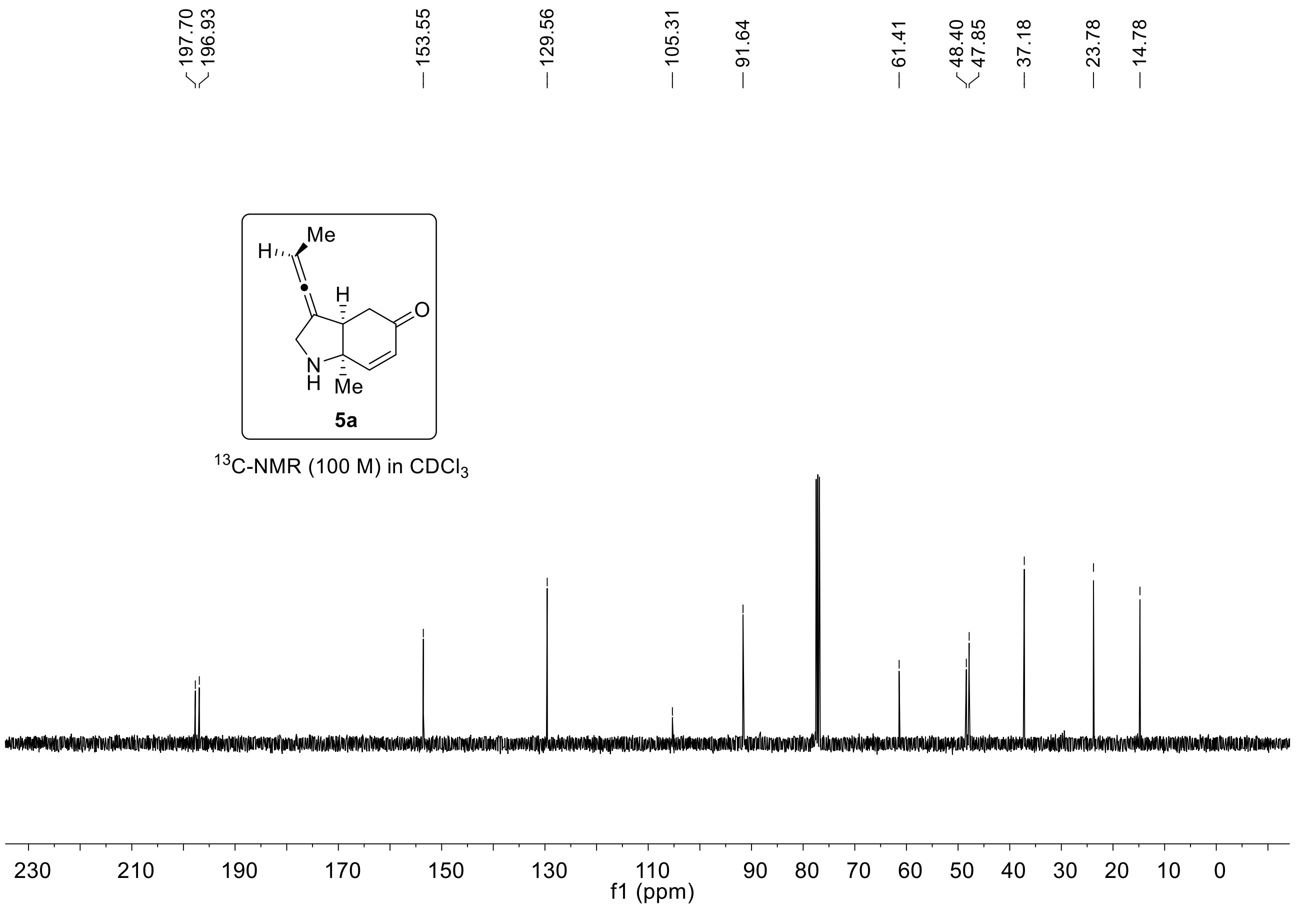

Supplementary Figure 121. ^{13}C NMR spectra for **5a**

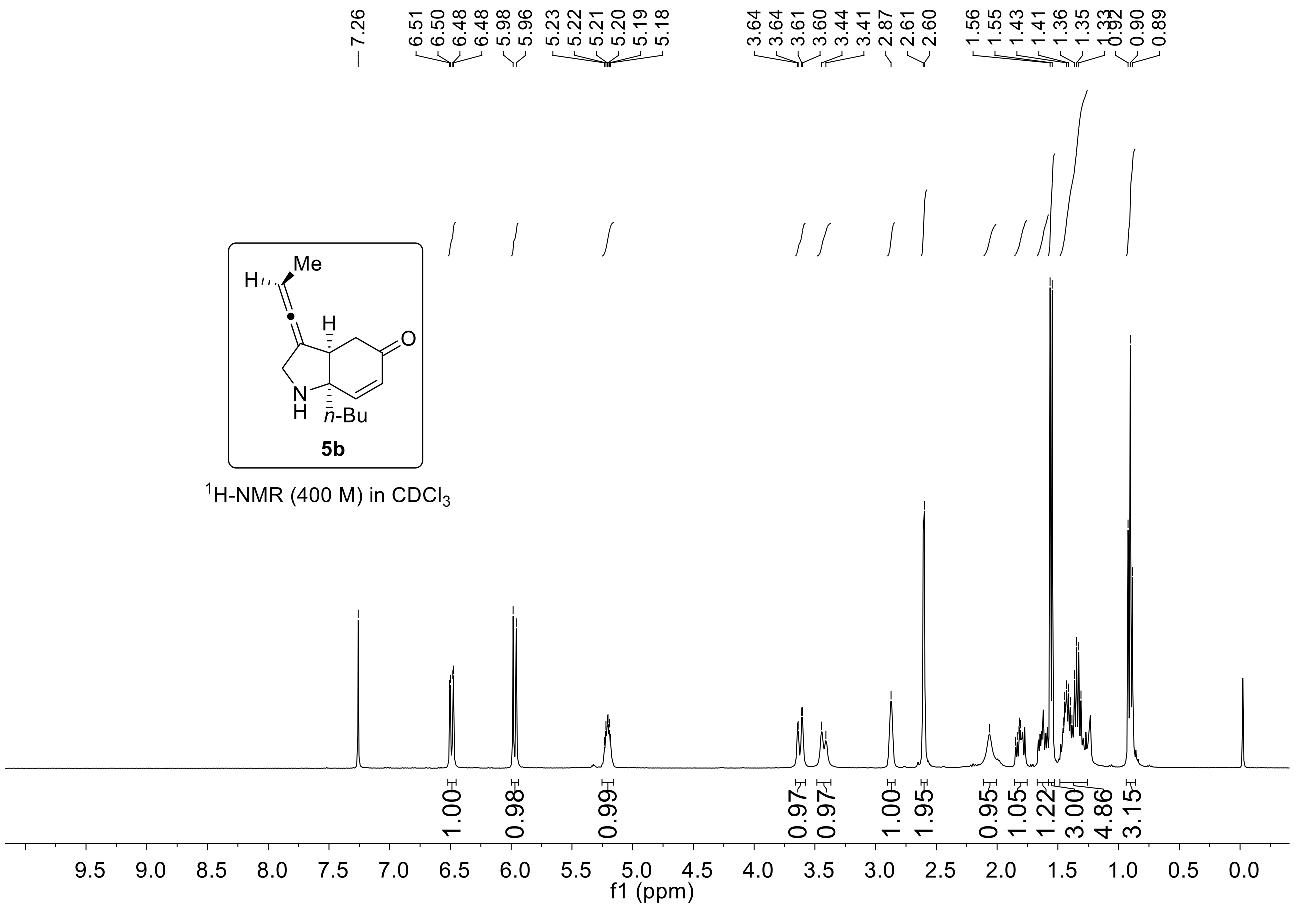

Supplementary Figure 122. ^{1}H NMR spectra for **5b**

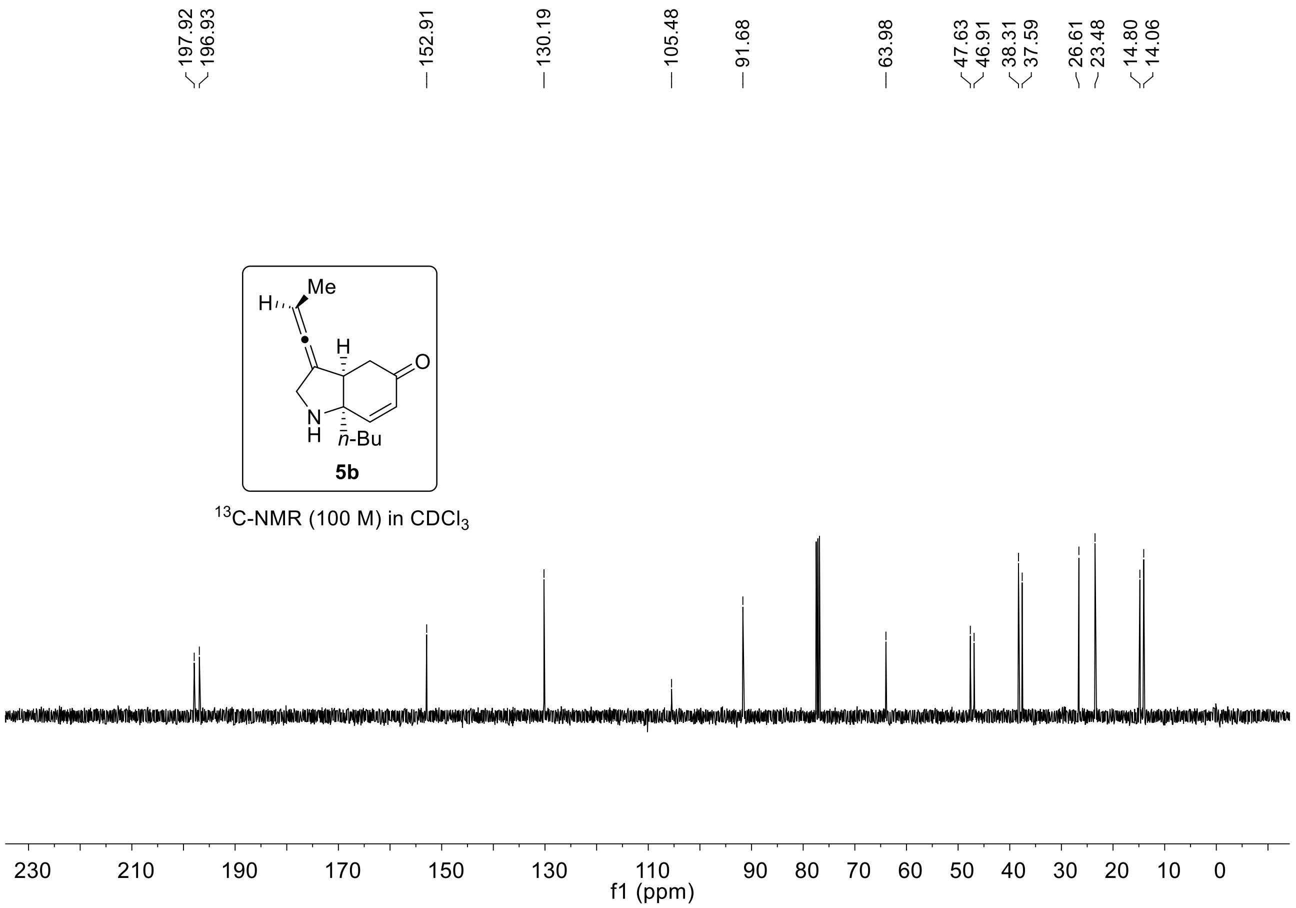

Supplementary Figure 123. ^{13}C NMR spectra for **5b**

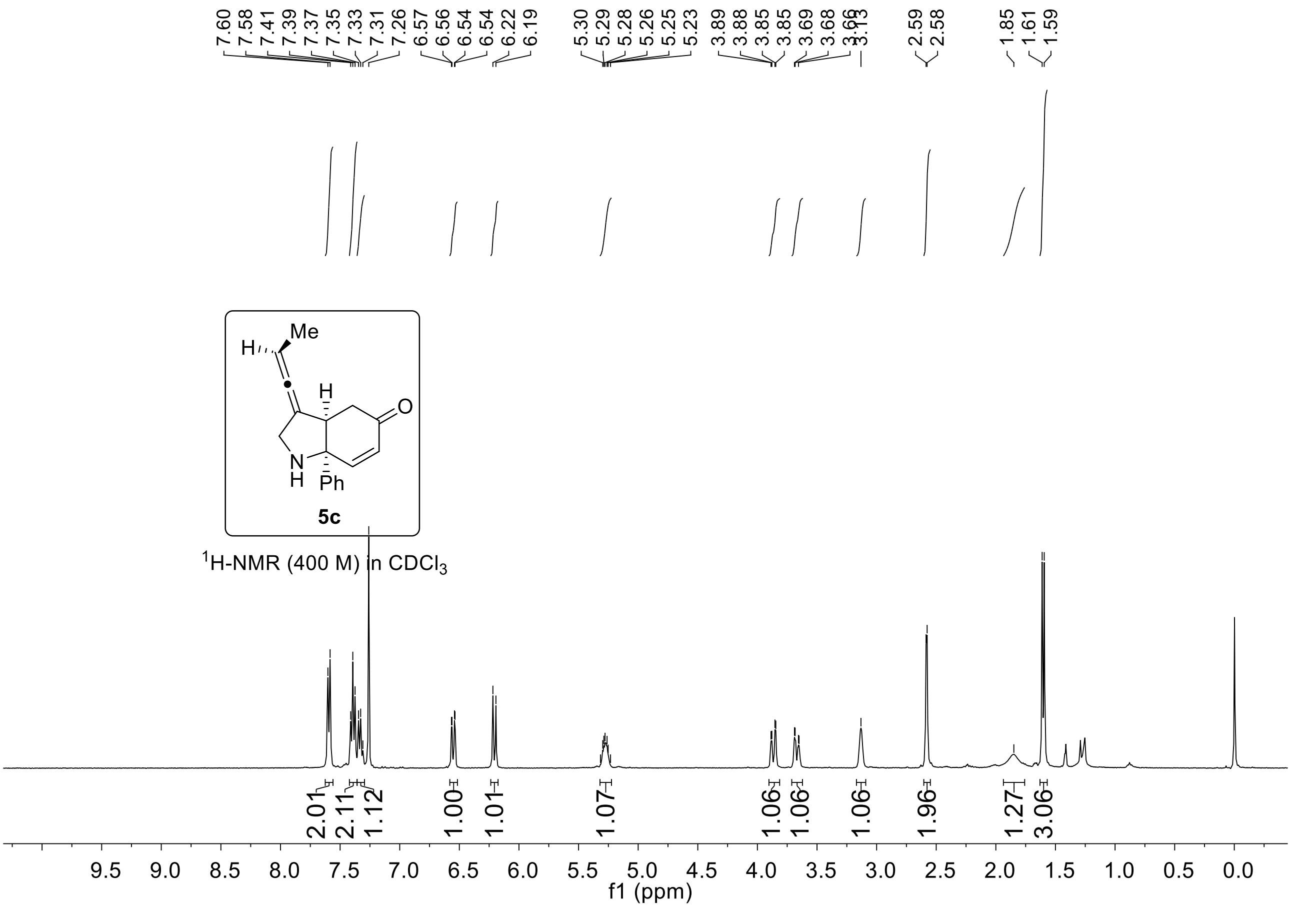

Supplementary Figure 124. ^{1}H NMR spectra for **5c**

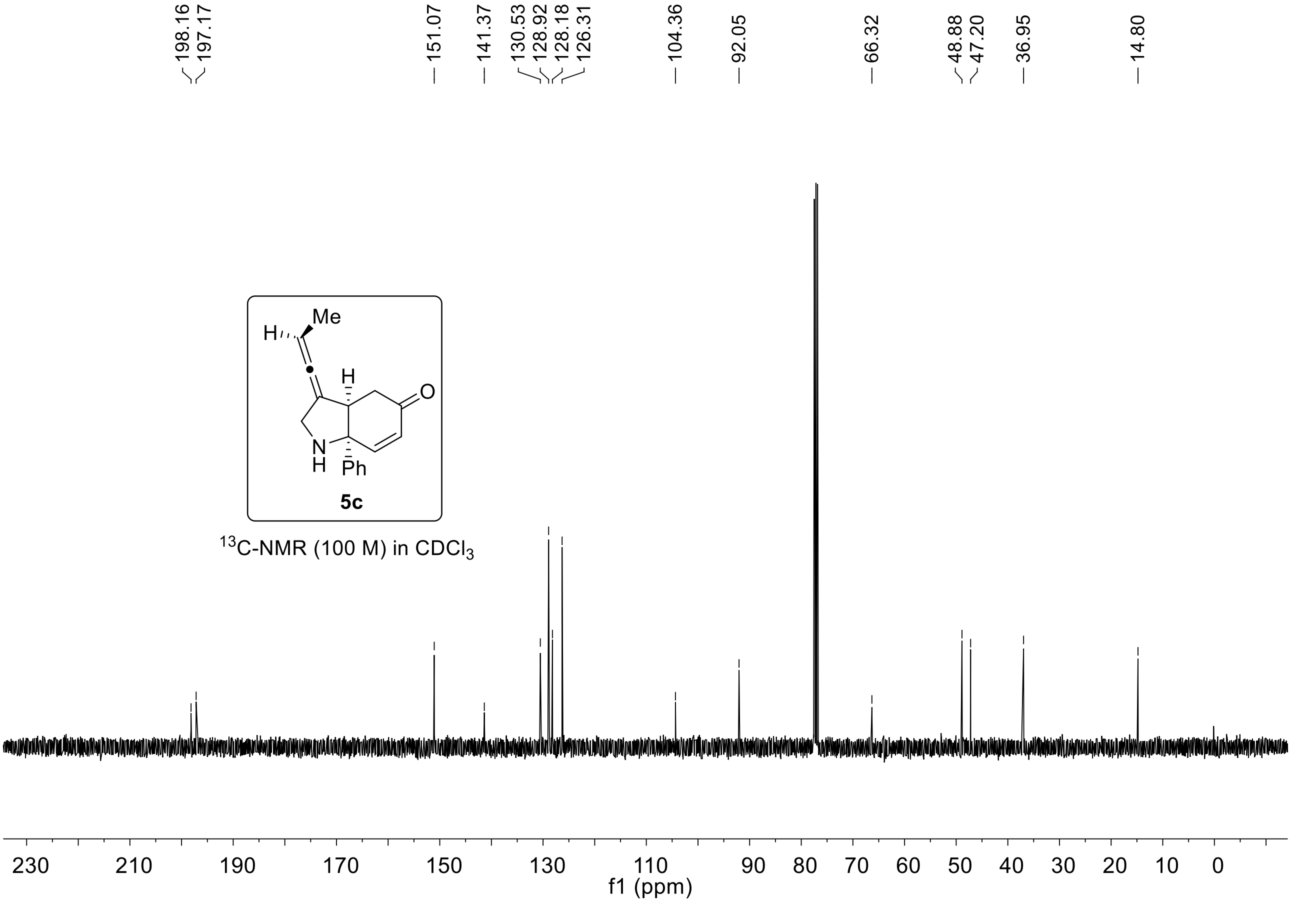

Supplementary Figure 125. ^{13}C NMR spectra for **5c**

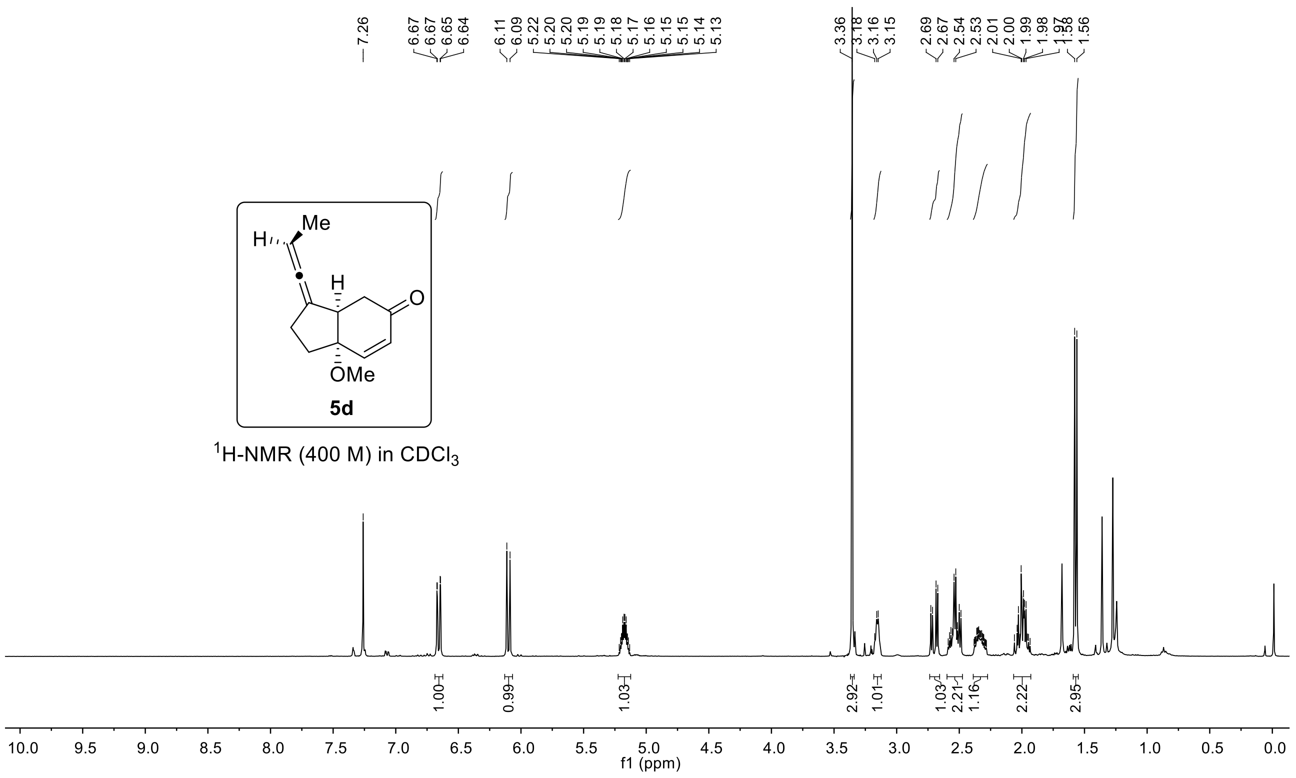

Supplementary Figure 126. ^{1}H NMR spectra for **5d**

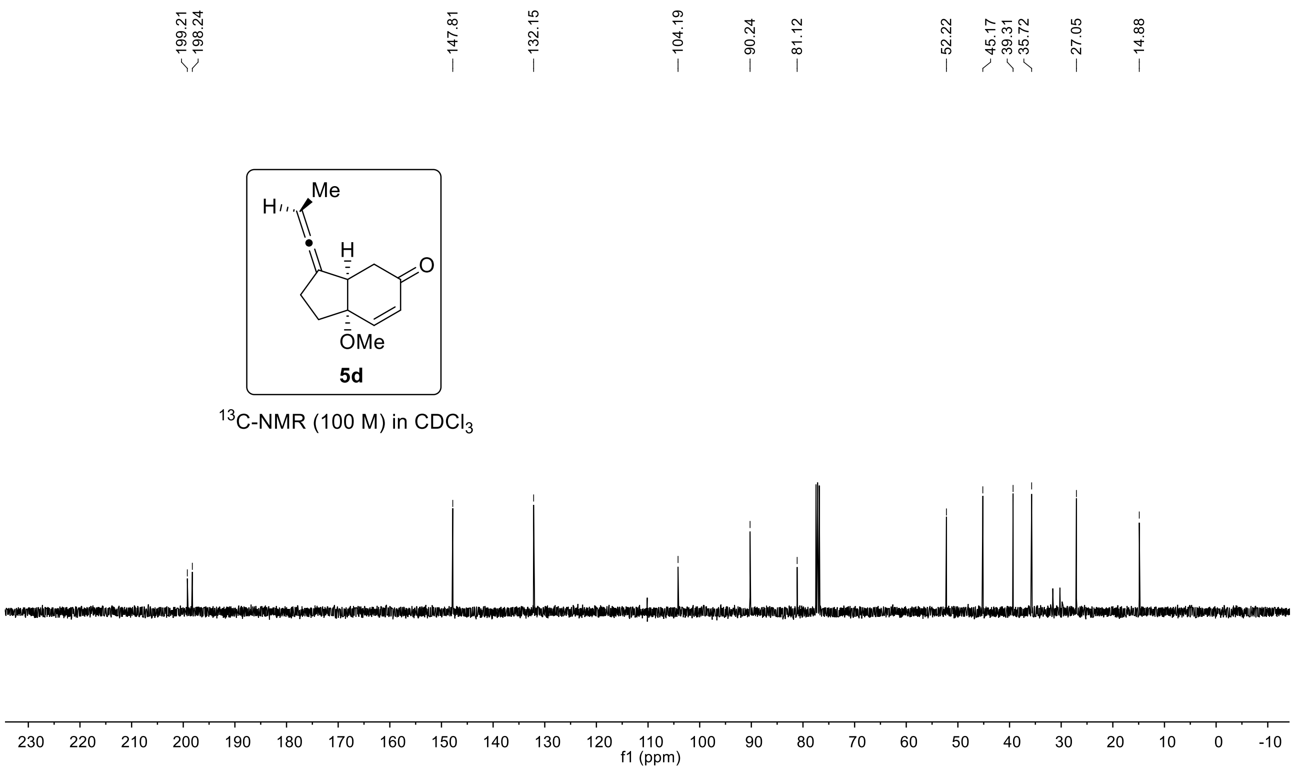

Supplementary Figure 127. ^{13}C NMR spectra for **5d**

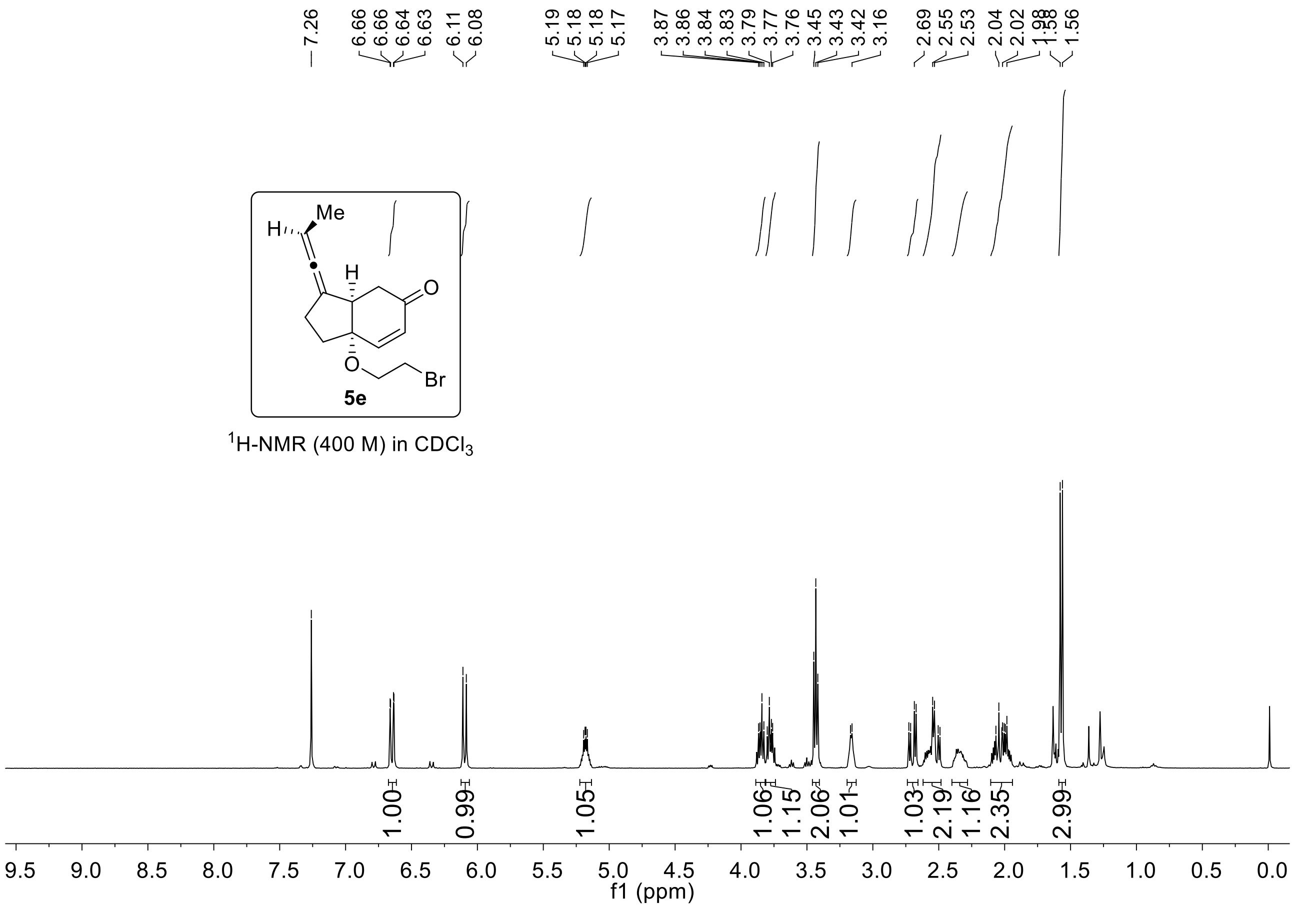

Supplementary Figure 128. ^{1}H NMR spectra for **5e**

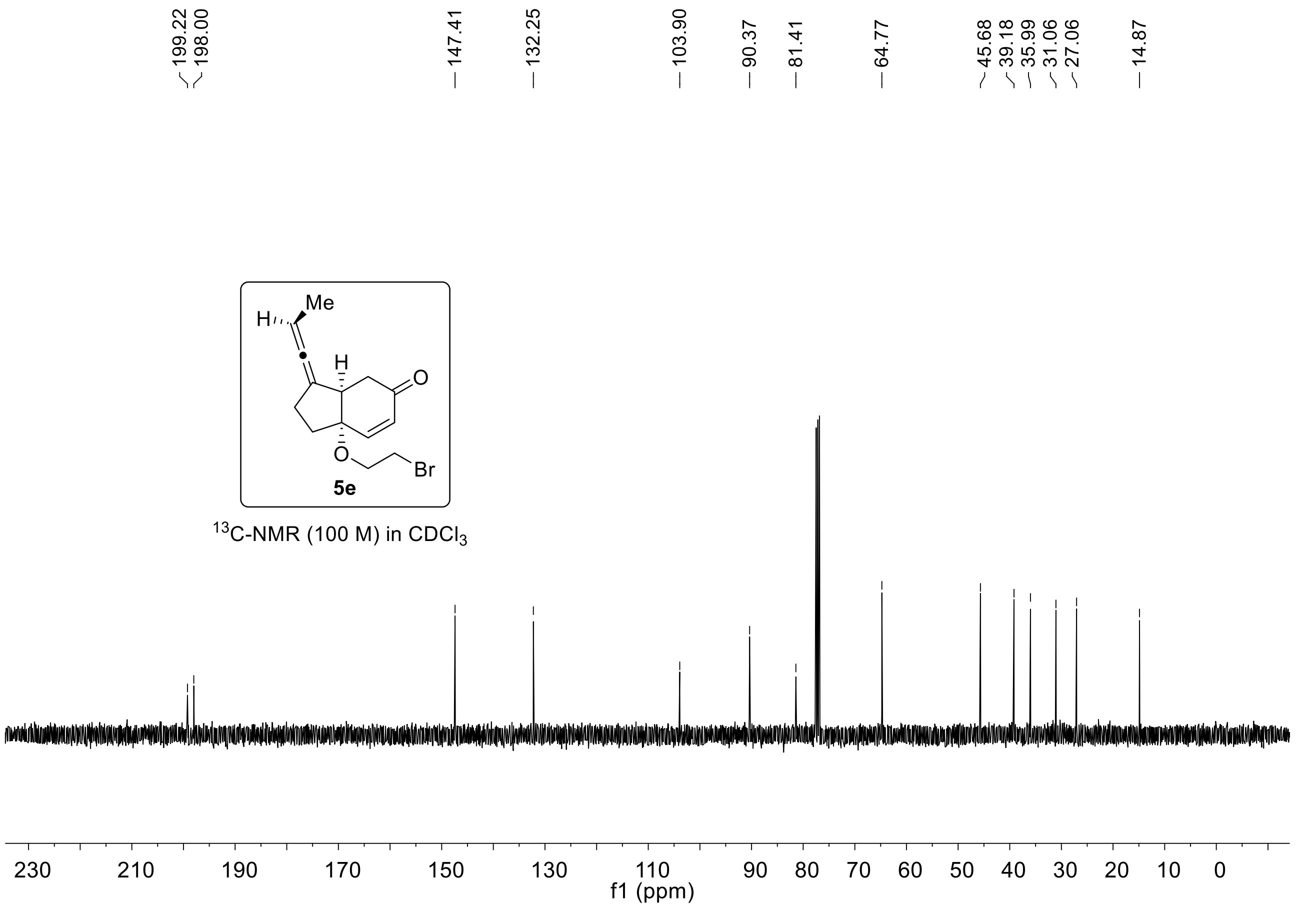

Supplementary Figure 129. ^{13}C NMR spectra for **5e**

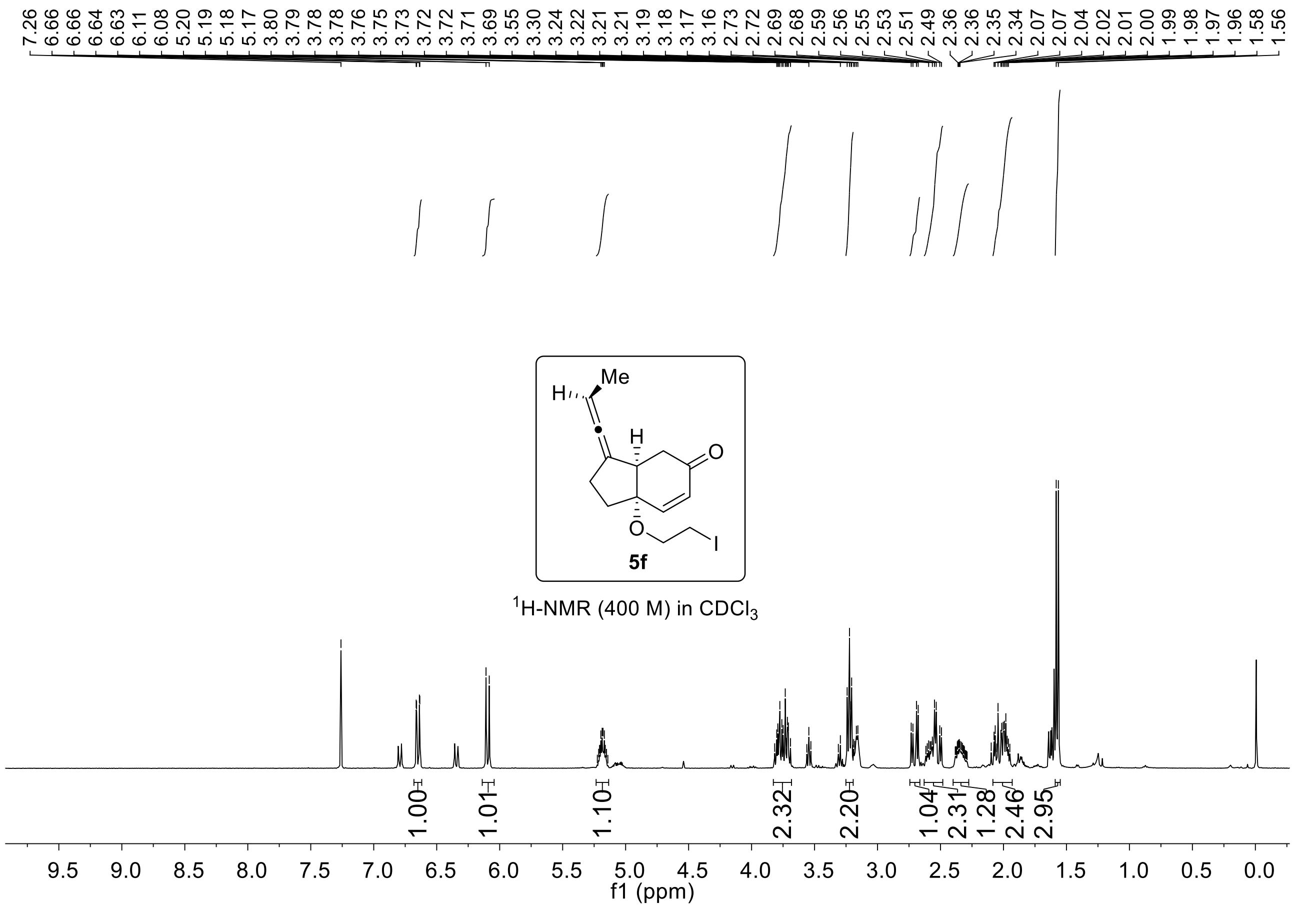

Supplementary Figure 130. ^{1}H NMR spectra for **5f**

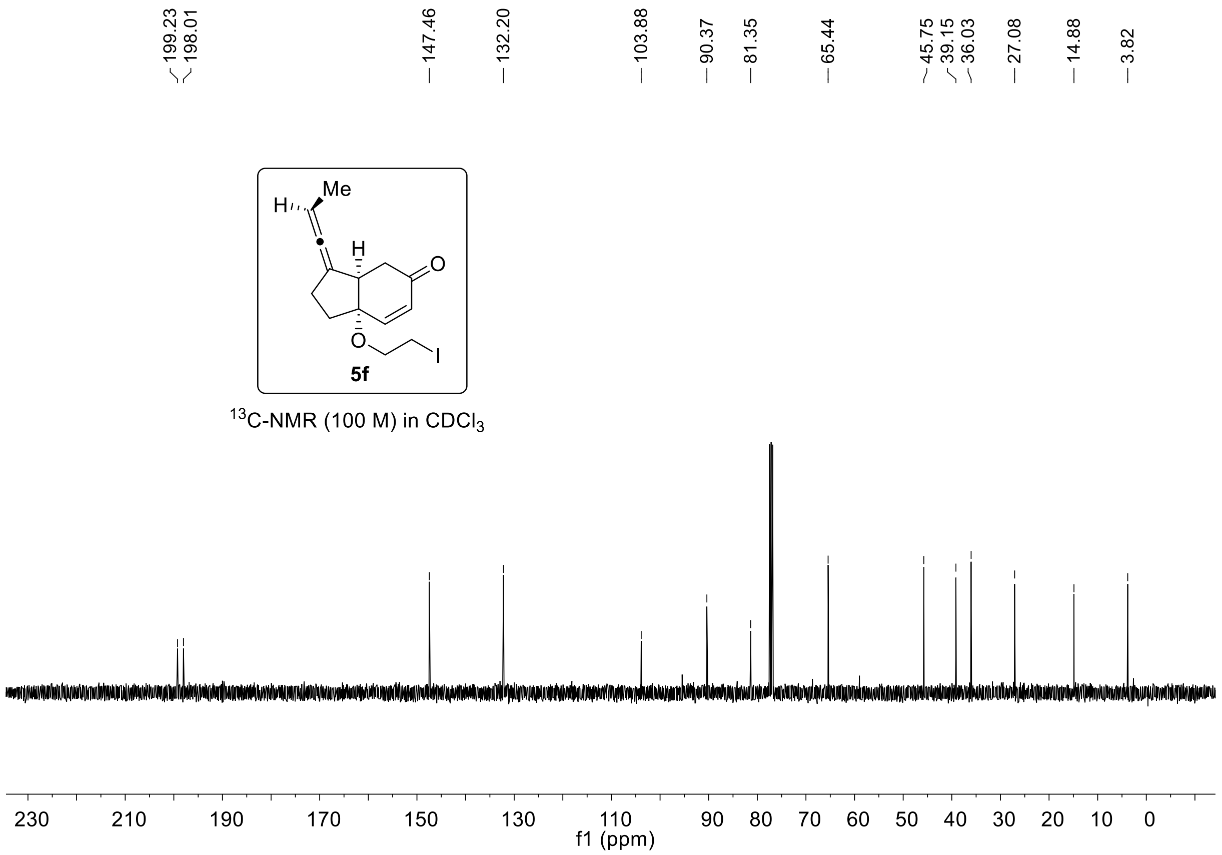

Supplementary Figure 131. ^{13}C NMR spectra for **5f**

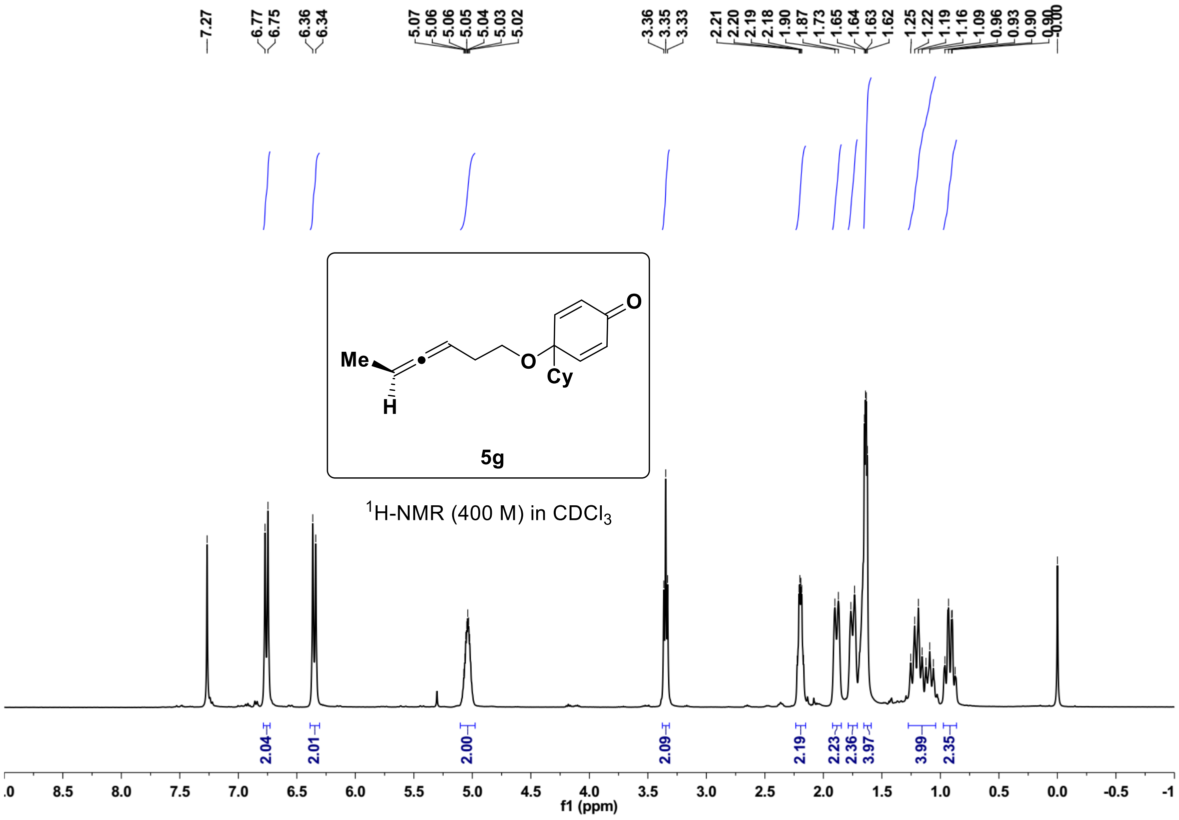

Supplementary Figure 132. ^{1}H NMR spectra for **5g**

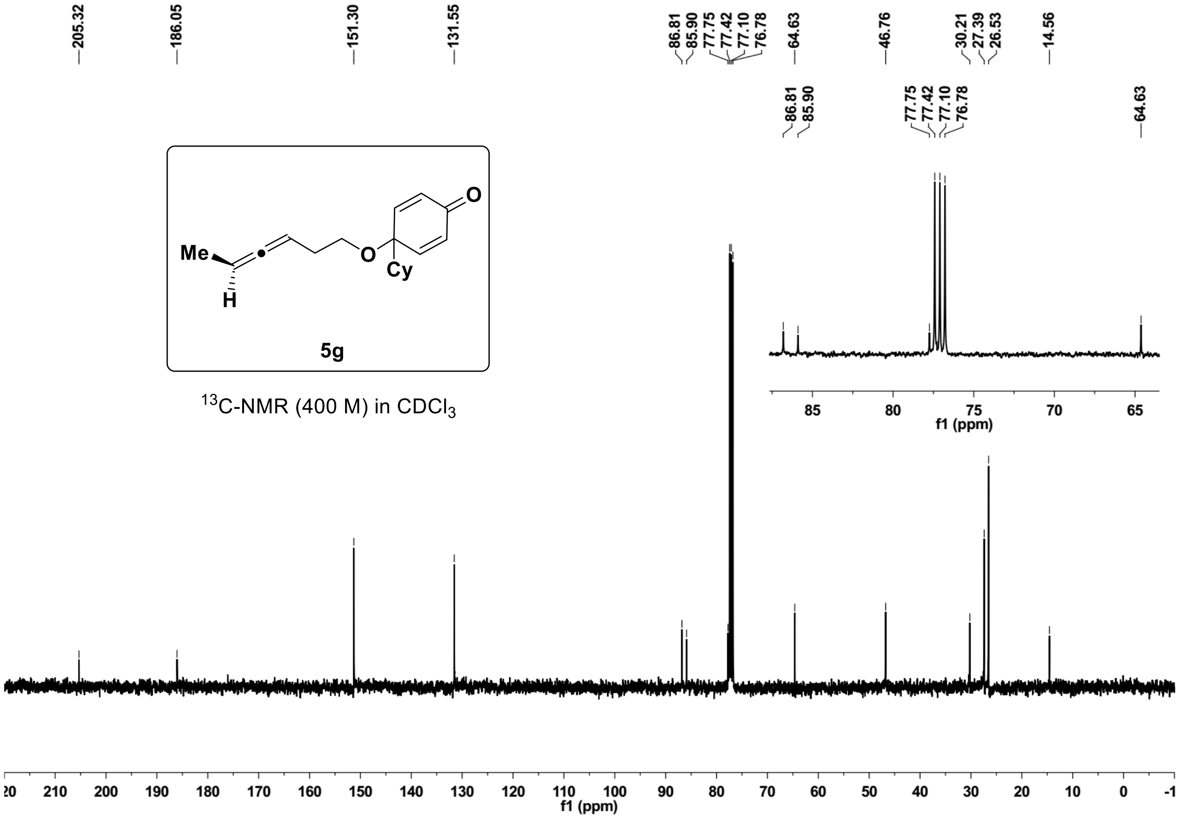

Supplementary Figure 133. ^{13}C NMR spectra for **5g**

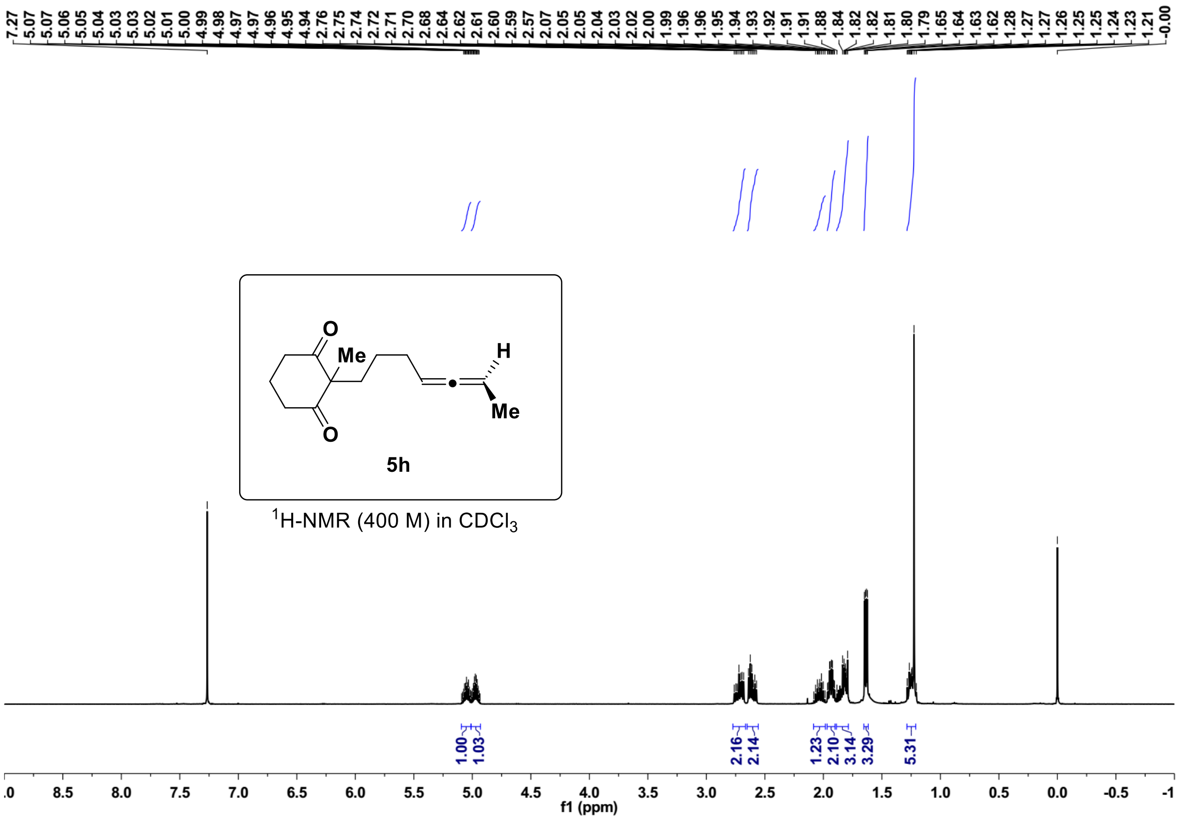

Supplementary Figure 134. ^{1}H NMR spectra for **5h**

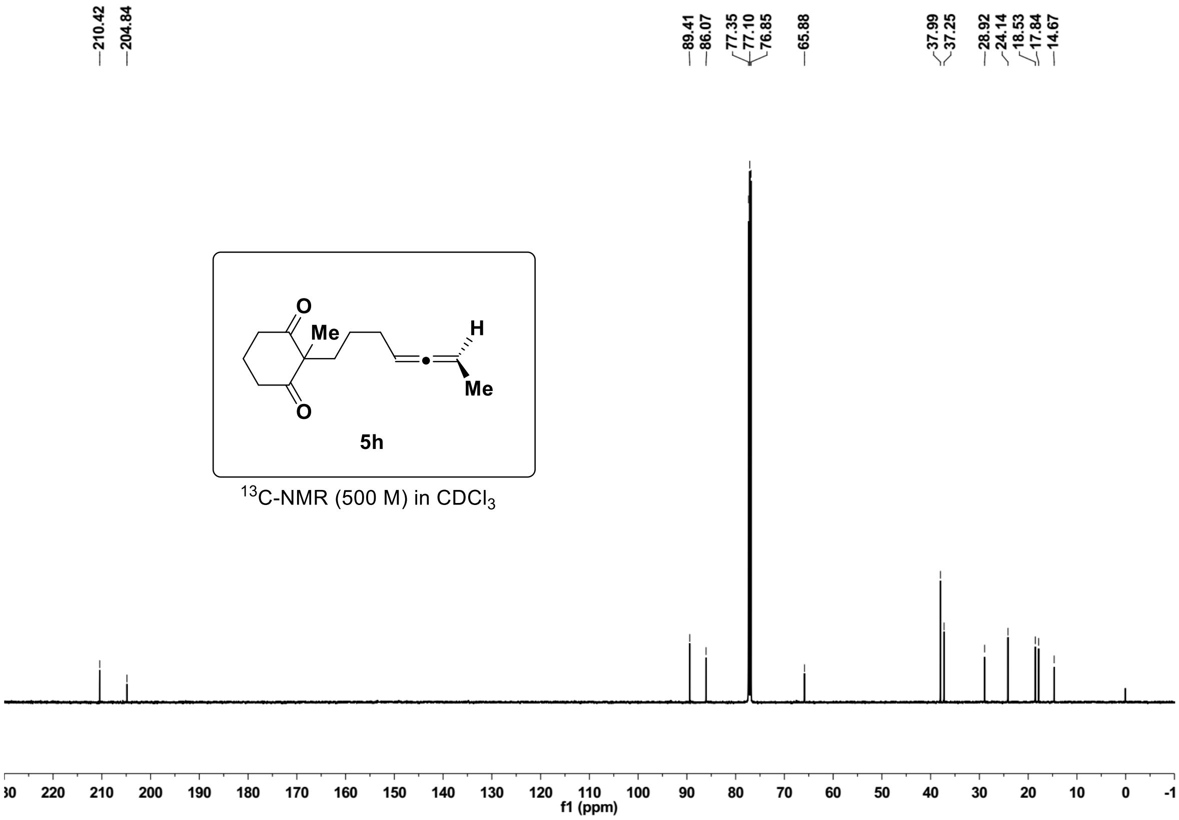

Supplementary Figure 135. ^{13}C NMR spectra for **5h**

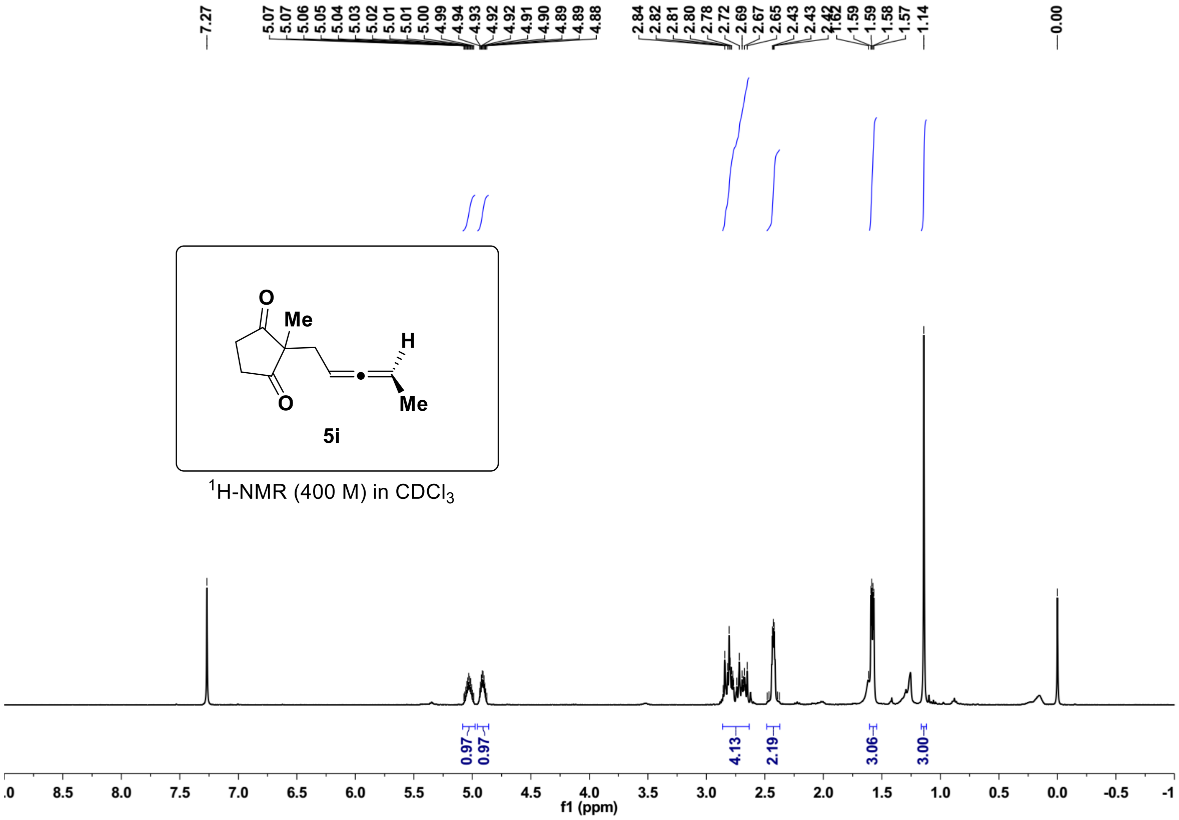

Supplementary Figure 136. ^{1}H NMR spectra for **5i**

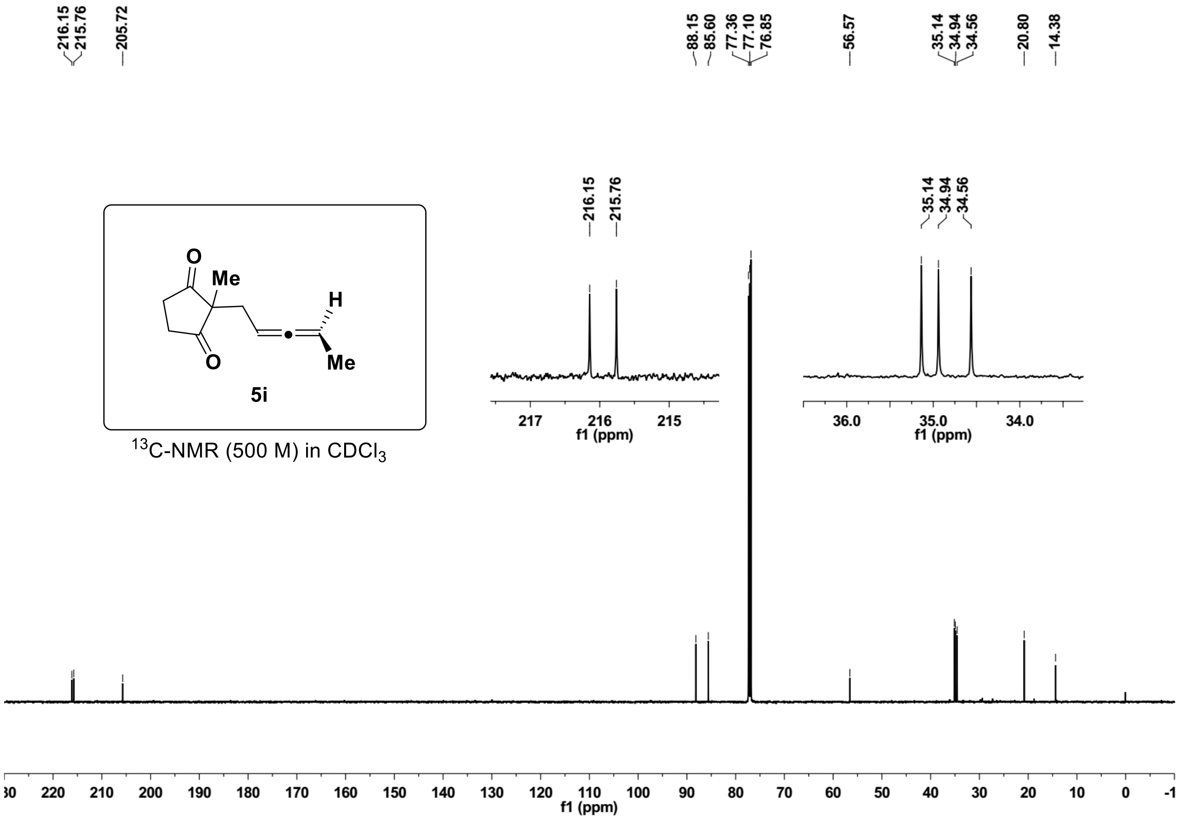

Supplementary Figure 137. ^{13}C NMR spectra for **5i**

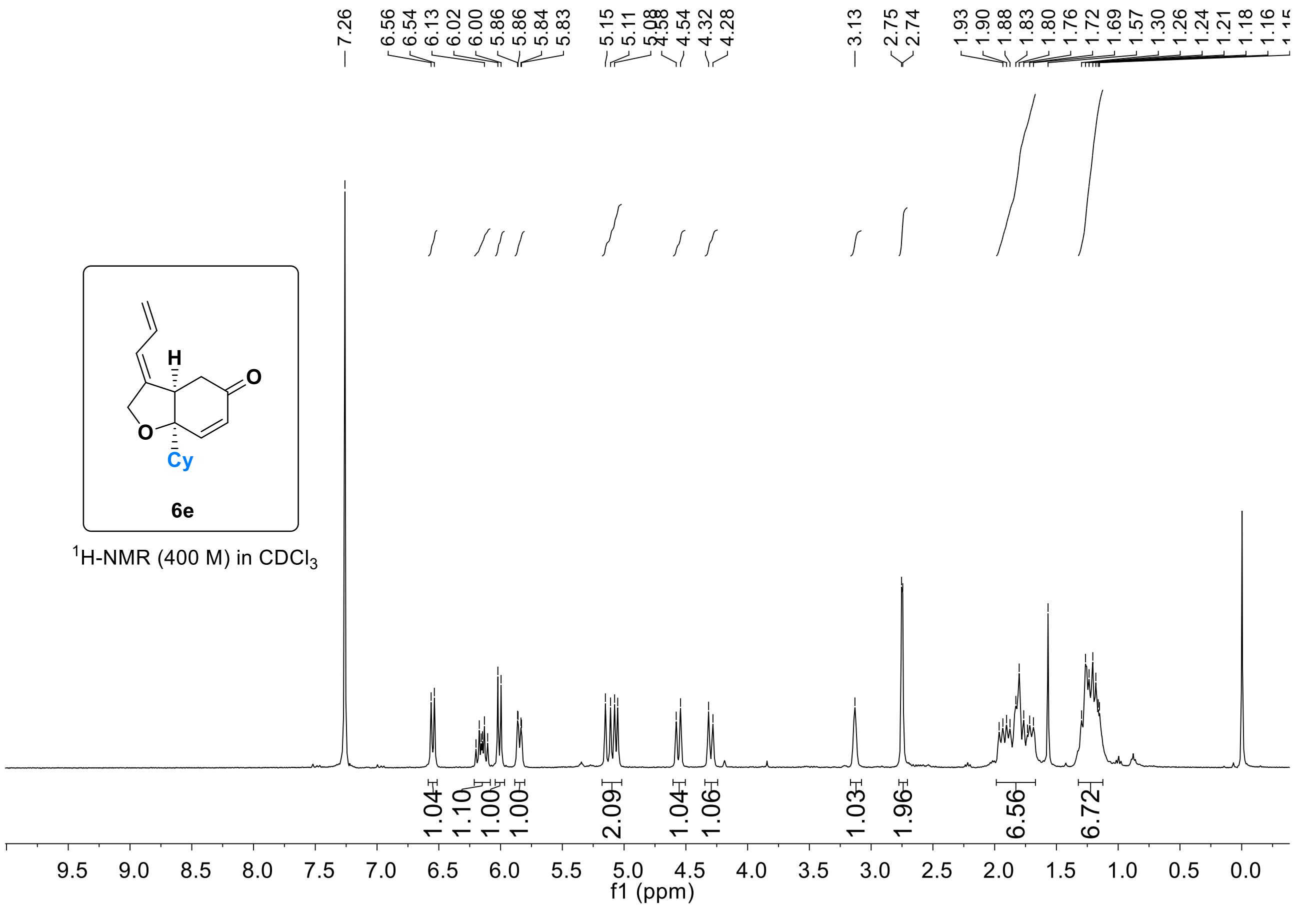

Supplementary Figure 138. ^{1}H NMR spectra for **6e**

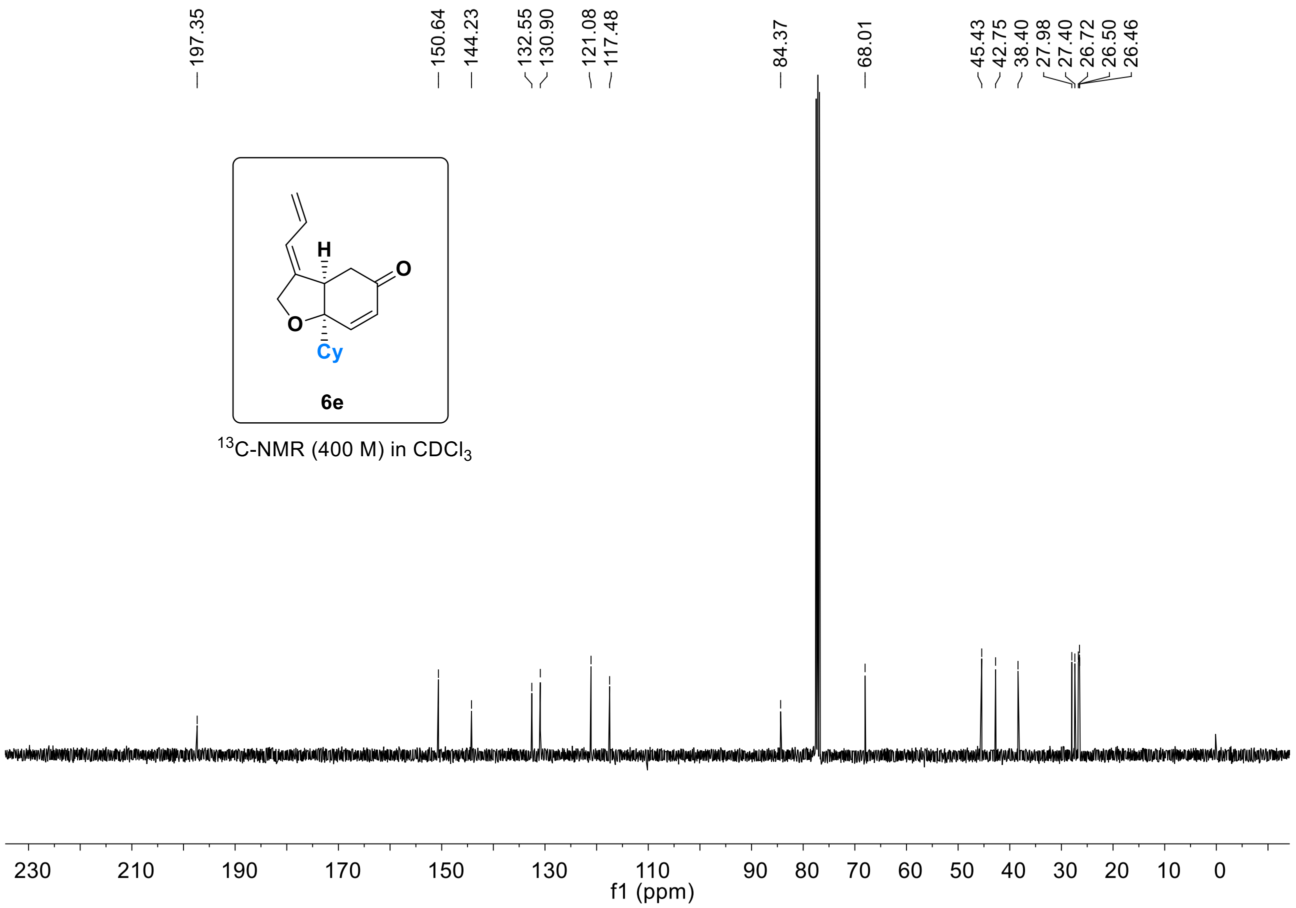

Supplementary Figure 139. ^{13}C NMR spectra for **6e**

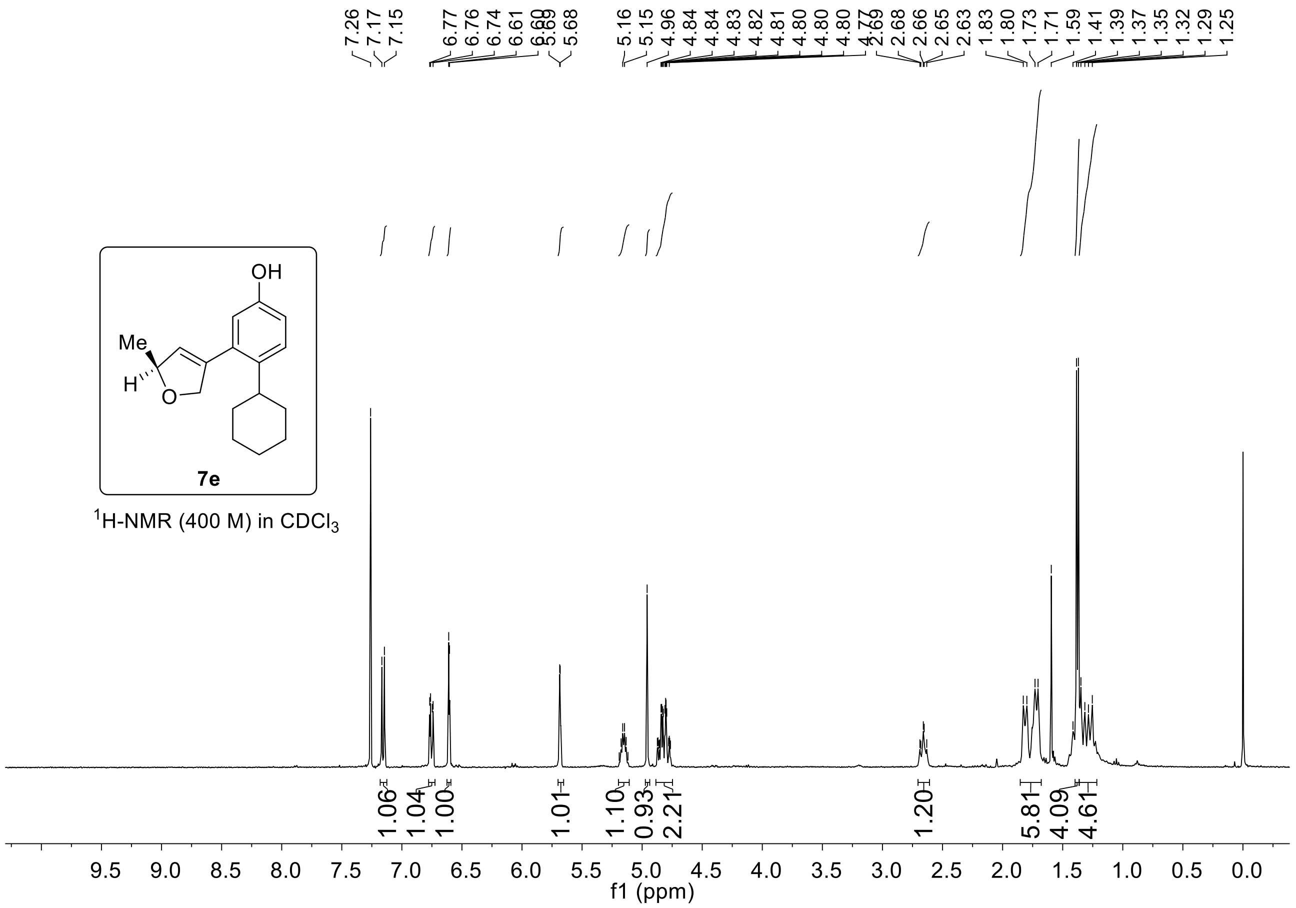

Supplementary Figure 140. ^{1}H NMR spectra for **7e**

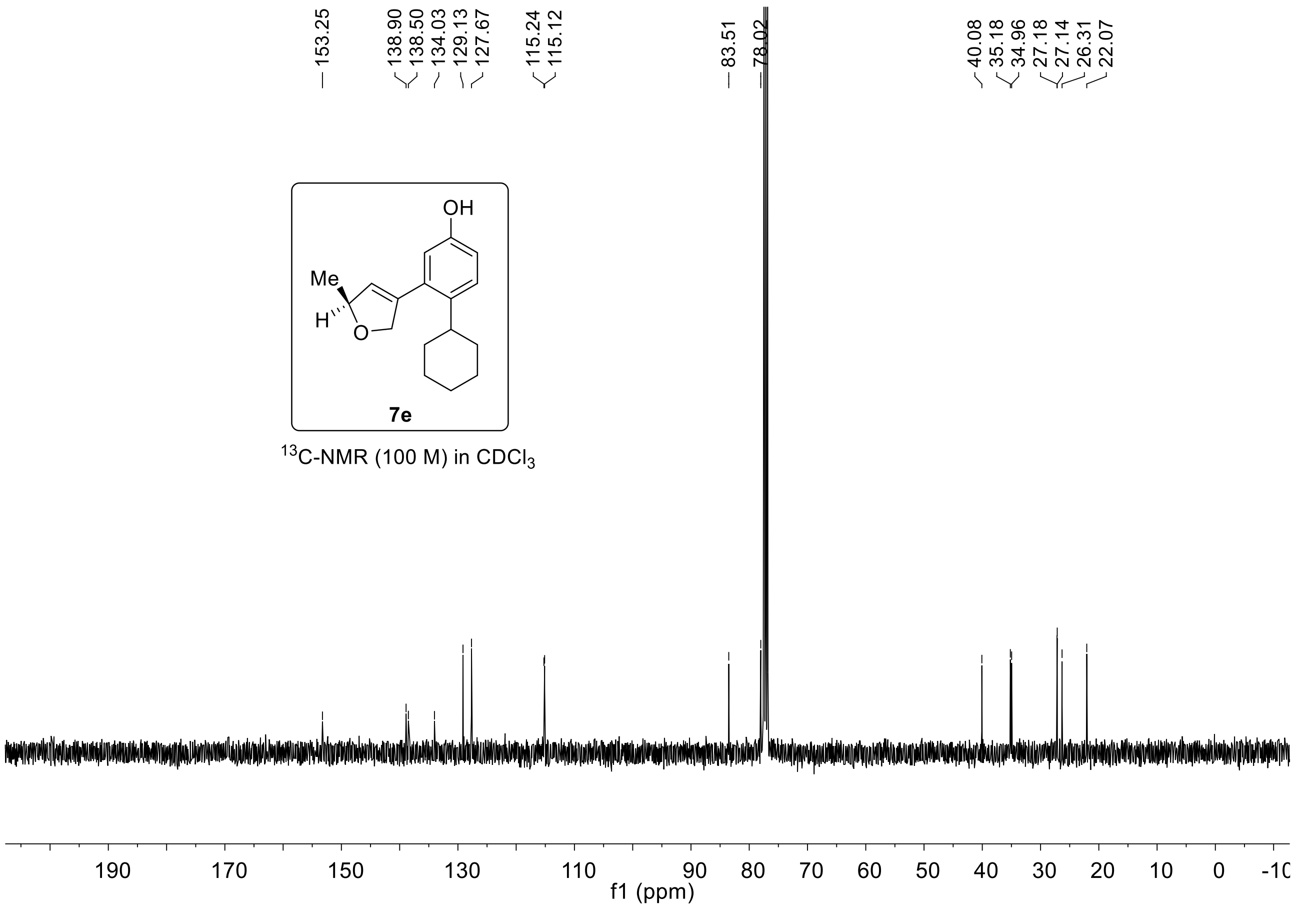

Supplementary Figure 141. ^{13}C NMR spectra for **7e**

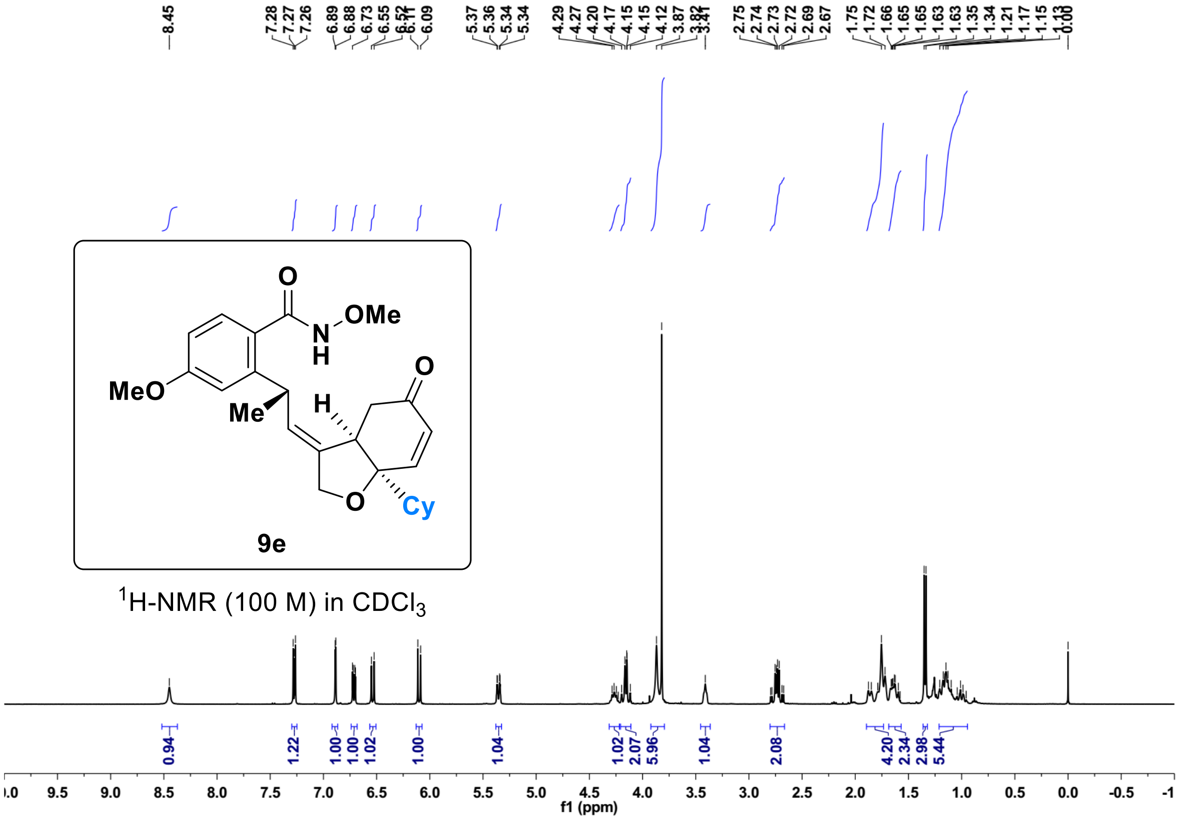

Supplementary Figure 142. ^{1}H NMR spectra for **9e**

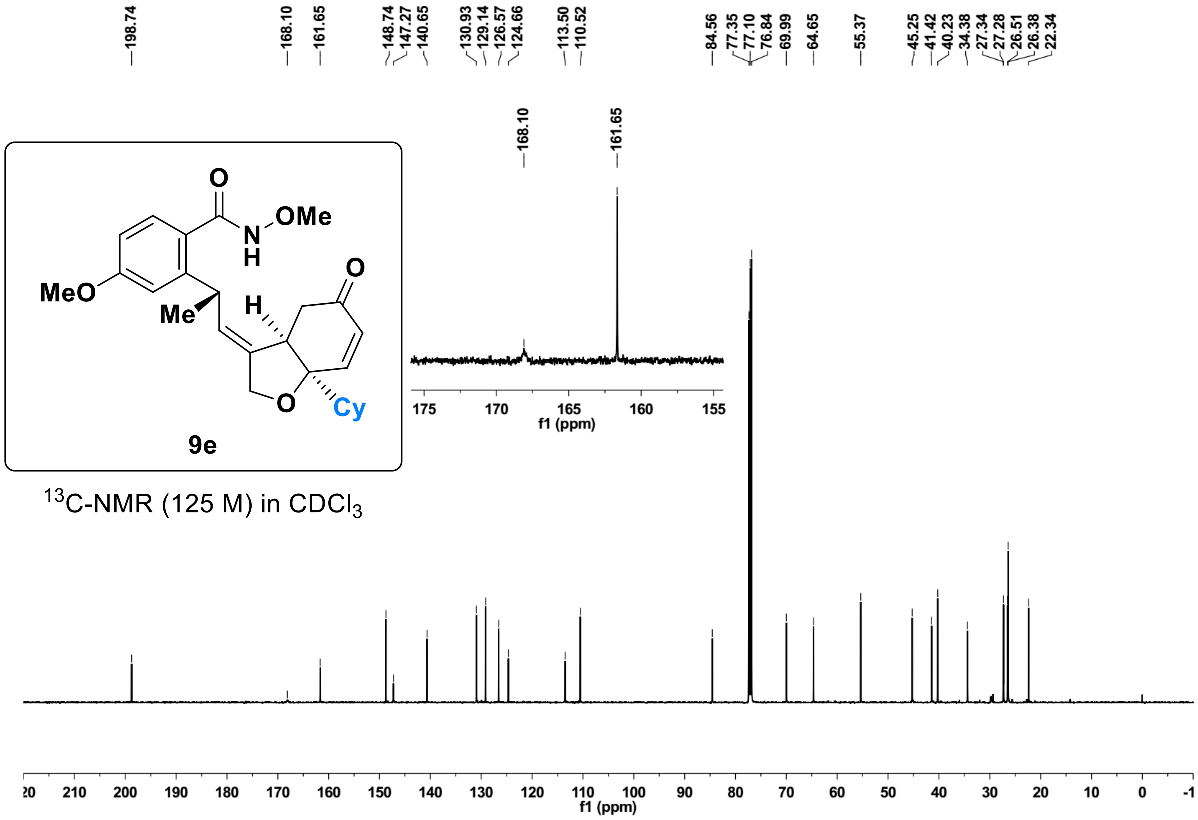

Supplementary Figure 143. ^{13}C NMR spectra for **9e**

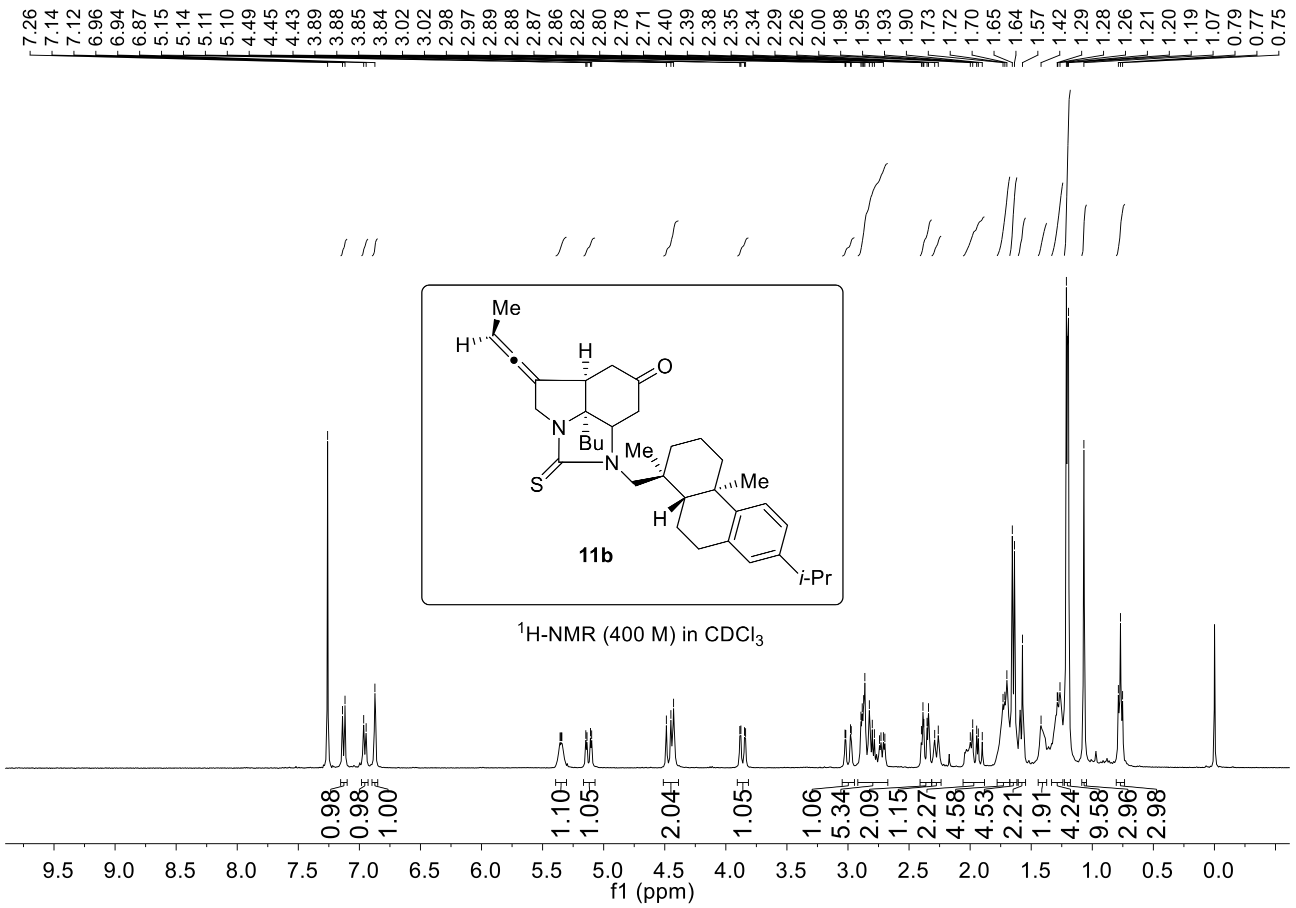

Supplementary Figure 144. ^{1}H NMR spectra for **11b**

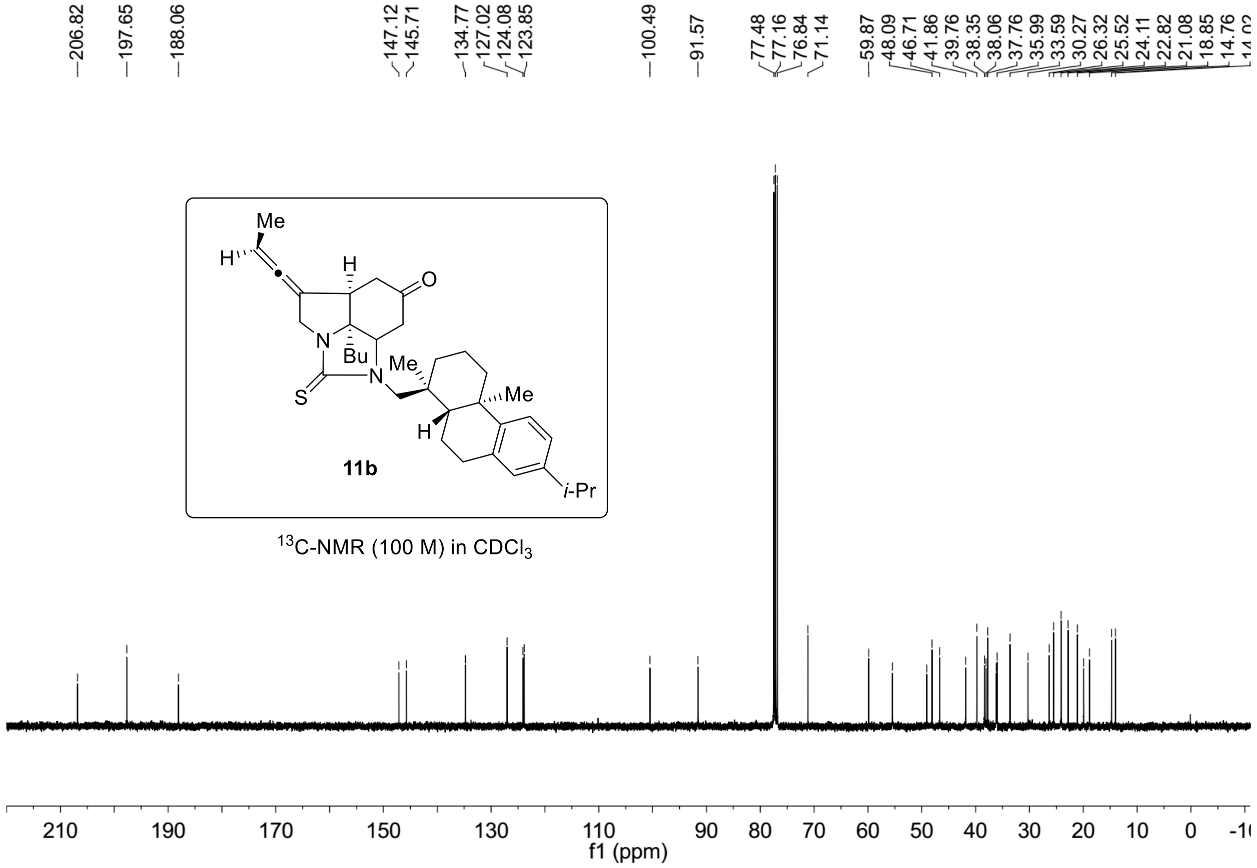

Supplementary Figure 145. ^{13}C NMR spectra for **11b**

Supplementary References

1. He, Z.-T. et al. Efficient access to bicyclo[4.3.0]nonanes: copper-catalyzed asymmetric silylative cyclization of cyclohexadienone-tethered allenes. *Angew. Chem. Int. Ed.* **54**, 14815-14818 (2015).
